# A multiscale cerebral neurochemical connectome of the rat brain

**DOI:** 10.1371/journal.pbio.2002612

**Published:** 2017-07-03

**Authors:** Hamid R. Noori, Judith Schöttler, Maria Ercsey-Ravasz, Alejandro Cosa-Linan, Melinda Varga, Zoltan Toroczkai, Rainer Spanagel

**Affiliations:** 1 Institute of Psychopharmacology, Central Institute of Mental Health, Medical Faculty Mannheim, University of Heidelberg, Mannheim, Germany; 2 Neuronal Convergence Group, Max Planck Institute for Biological Cybernetics, Tübingen, Germany; 3 Institut des Hautes Etudes Scientifiques, Bures-sur-Yvette, France; 4 Faculty of Physics, Babes-Bolyai University, Cluj-Napoca, Romania; 5 Romanian Institute of Science and Technology, Cluj-Napoca, Romania; 6 Physics Department and the Interdisciplinary Center for Network Science and Applications, University of Notre Dame, Notre Dame, Indiana, United States of America; Allen Institute for Brain Science, United States of America

## Abstract

Understanding the rat neurochemical connectome is fundamental for exploring neuronal information processing. By using advanced data mining, supervised machine learning, and network analysis, this study integrates over 5 decades of neuroanatomical investigations into a multiscale, multilayer neurochemical connectome of the rat brain. This neurochemical connectivity database (ChemNetDB) is supported by comprehensive systematically-determined receptor distribution maps. The rat connectome has an onion-type structural organization and shares a number of structural features with mesoscale connectomes of mouse and macaque. Furthermore, we demonstrate that extremal values of graph theoretical measures (e.g., degree and betweenness) are associated with evolutionary-conserved deep brain structures such as amygdala, bed nucleus of the stria terminalis, dorsal raphe, and lateral hypothalamus, which regulate primitive, yet fundamental functions, such as circadian rhythms, reward, aggression, anxiety, and fear. The ChemNetDB is a freely available resource for systems analysis of motor, sensory, emotional, and cognitive information processing.

## Author summary

The mammalian brain consists of a network of chemically diverse, multiscale, and multilayer neuronal wiring patterns that form the physical infrastructure underlying the processing of motoric, sensory, emotional, and cognitive information. Decades of histological track-tracing studies have generated a set of highly valuable yet disorganized data, which is very hard to retrieve in a hypothesis-free manner. We present an open-access database (ChemNetDB) that organizes over 50 years of neuroanatomical track-tracing and neurochemical measurements from 36,464 rats. This neurochemical connectome is terminologically consistent and shares several network invariants with mouse and macaque cortical networks, suggesting that the mammalian brain exhibits universal structural features. Furthermore, several network measures reflect and confirm the key functional roles of deep subcortical brain areas, which are known to be responsible for primitive, yet essential and evolutionary conserved functions related to survival. ChemNetDB provides the first whole-brain, multiscale, and consistently collated rat connectome database. ChemNetDB also includes neurochemical specificity and represents a powerful tool for in vitro, in vivo, and in silico investigations of brain function and disorders.

The central nervous system consists of a multiscale chemically multilayer network that represents the integration of macro-, meso-, and microconnectomes [1]. Groups of morphologically and physiologically diverse neurons are wired as connectivity patterns with a certain degree of universality across species and individual variability. This multiscale wiring diagram provides the topological foundation of motor, sensory, emotional, and cognitive information processing in the brain and has attracted the attention of neuroanatomists for decades. However, the majority of the existing data are derived from histological track tracing studies, which are qualitative, incomplete, variable, scattered, and hard to retrieve.

This disorganized big data were mostly treated in a knowledge-based, hypothesis-driven manner to characterize disease-related neurocircuitries [2–7]. In recent years and with the increase of computational power, this strategy is being replaced by empirical and evidence-based approaches. For rats, extensive data mining combined with systematic analysis has already led to the identification of microcircuits of specific brain regions such as the cerebral cortex, amygdala, and hippocampus [8–10]. At the mesoscale, Oh and colleagues [11] used a systematic and standardized approach to identify the brain-wide cellular level connectome of the mouse brain and thereby provided the first experimentally extended atlas for a single vertebrate animal. At the macroscale, dynamical databases for rats and primates such as BAMS (bams1.org), neurocircuitry for modeling drug effects [12], the connectome of basal ganglia [13–15], and CoCoMax (cocomac.g-node.org, [16]) currently provide valuable resources for the analysis of brain connectivity and the modeling of brain dynamics. Yet, the lack of integration of neurochemical data, the low density of these networks (e.g., BAMS2 database with a maximum coverage of 11.2%), as well as the separation of meso- (i.e., intraregional connections) and macroconnectomes (i.e., neuronal projections between distinct brain regions) limit the applicability of these databases. Thus, a cohesive and comprehensive understanding of the rat neurochemical connectome is still missing.

ChemNetDB aims to address this knowledge gap by integrating neuroanatomical track tracing and neurochemical data from 36,464 rats into a terminologically accurate database of the multiscale, neurochemical connectome of the rat brain. To achieve this goal, a methodological combination of neuroinformatics, machine learning, and network analytic techniques was utilized. Hence, by superimposing the connections, neurochemistry, and cell types in a terminologically and mathematically consistent manner on anatomical data, this database provides a comprehensive framework for the effective study of the normal and diseased brain and constitutes a robust reference for future in vitro, in vivo and in silico neuroscience studies.

## Results

### Data selection process and overall estimates

Automatic keyword-based search (1,750 keywords) and manual grey search on electronic databases revealed 124,694 abstracts, titles, or both identified as original publications. Out of these, 4,517 studies were relevant for data mining and data from 1,560 original research articles with 36,464 rats were selected for the connectome identification based on the inclusion criteria. A flow diagram of the study selection process is represented in supplementary information (S1 Fig).

According to our stringent inclusion and exclusion criteria, these selected studies represent the relevant published outcome of 55 years of neuroanatomical research. All selected studies were performed in outbred rats with no specific genotype or phenotype. Furthermore, animals did not receive any pharmacological treatment or behavioral training. On average, 86.08% ± 0.02% of the cases were male animals. The strain of the animals was inverse Gaussian distributed (μ = 0.25;λ = 1.24). Thereby, 54.03% ± 0.01% and 24.41% ± 0.01% of the animals were Sprague-Dawley and Wistar rats, respectively.

### Brain-wide connectivity database

In total, the systematic literature search identified 281 interconnected nuclei (4,585 axonal projections) of cerebral regions, cerebellum, and components of medulla oblongata. The connectivity matrices associated with this raw database are presented in the supplementary information (S7, S8 and S9 Tables) along with Bregma levels from rostral to caudal. The nomenclature-consistent, brain-wide (cerebral) core of the chemical connectivity database (ChemNetDB) comprises by 188 cortical and subcortical morphologically distinct regions with 3,712 connections. The database references are provided within S1 Text.

This database considerably differs from the existing macroscopic connectomes [17,18] of the rat central nervous system. In contrast to ChemNetDB, which is a multiscale connectome database, these databases are often restricted to a single spatial scale. Furthermore, with the exception of a few brain regions such as amygdala, the bed nucleus of stria terminalis (BNST), and cerebral cortex, they are only sparsely connected, and do not provide edge-complete connectivity. Databases lacking the edge-completeness property include brain regions that are either not connected to any other region, or receive only afferents from other regions, or send only efferents to other brain areas. None of these cases are biologically or physically feasible within the central nervous system. Thus, these databases effectively present cerebral subnetworks of the full connectome. In addition, concerns regarding nomenclature and data integration (such as combining data from neonatal and adult animals) and other inconsistencies make these platforms much more difficult to use. ChemNetDB overcomes these issues and shows significant advantages over the existing databases in that (1) it is currently the most comprehensive multiscale database that contains previous databases as subsystems and it is nearly edge-complete; (2) it integrates neurochemical information in a consistent and validated manner; and (3) it is consistent with respect to age of the animals and nomenclature (see S2 Text for more detail).

### A multiscale connectome

**Fig 1:**
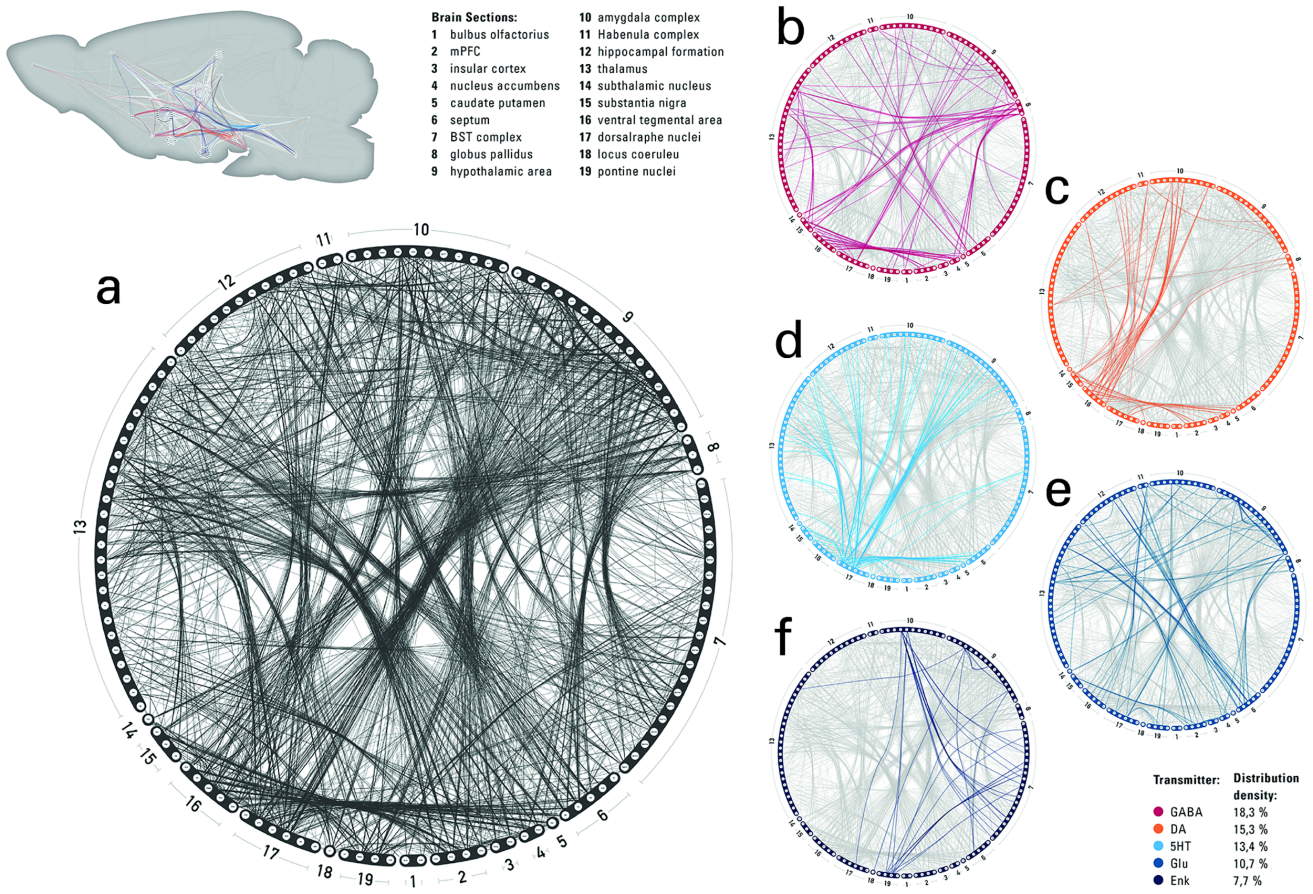
Schematic representation of the neurochemical connectome. (a) Connectogram of the 125-node cerebral connectome partitioned in 19 large-scale brain regions. With 18.36%, the gamma-aminobutyric acid-ergic (GABAergic) neuronal connections constitute the dominant chemical components (b) followed by dopaminergic subnetwork 15.37% (c), serotoninergic connections 13.47% (d), glutamate system 10.75% (e), and enkephalinergic connections 7.76% (f).

Divisive hierarchical algorithms with edge-completeness constraints further reduced the dimension of the network and integrated the extracted cerebral core into a multilayer cerebral neurochemical connectome of the rat brain. Neurochemical connections within and between brain regions were mapped into a 3-dimensional space using a standardized platform to generate a comprehensive and quantitative database of inter-areal and cell-type−specific projections. The connectome consists of 125 scale-consistent cerebral nuclei and 2,931 multiscale, multichemical connections (Fig 1a). Thereby, 632 bidirectional, 1,642 unidirectional connections, and 25 loops utilize 25 different neurochemical compounds, such as amino acids, monoamines, cannabinoids,- opioids, and several other neurotransmitters. Despite such diversity, the chemical coverage of the network is only 25.08% and the majority of the connections were treated as binary links. Gamma-aminobutyric acid-ergic (GABAergic) neuronal connections (Fig 1b) constitute the dominant chemical components at the short-range (18.36% of the chemically denoted connections). In contrast, dopamine (15.37%; Fig 1c), serotonin (13.47%; Fig 1d), glutamate (10.75%; Fig 1e), and enkephalin (7.76%; Fig 1f) represent the majority of chemical colocalizations of long-range axonal projections.

A comprehensive systematic review on receptor distribution according to their mRNA expression levels and receptor binding was used to improve the robustness of these observations by providing spatial colocalization patterns of the transmitter systems within the connectome (S10–S13 Tables). For this purpose data on glutamatergic (mGluR1-7, GluK4-5, GluA1-4, GluN1-2(A-D)), monoaminergic (D1-D5, 5-HT1-7(A-C), β1–2, α1-2(A-D)), cholinergic (M1-M5, nAChRα(2–6)β(2–3)), GABAergic (GABA_A_α(1–6)β(2–3)γ(1–3)δ, GABA_B1_(a,b,p), GABA_B2_), opioid (μ, δ, κ, ORL1), and cannabinoid receptors from 103 (out of 1,843) selected original research articles are qualitatively integrated into brain-wide receptor distribution density maps (S2–S12 Figs).

In the following, we provide examples of network analyses of the ChemNetDB.

### Structural network properties

First, we investigated the network properties of the whole-brain connectome at 2 levels of resolution, namely as a coarser 19-node network (see Methods) described by the *G*_19×19_ adjacency matrix [12] and then at full resolution, as a 125-node network (*G*_125×125_).

The *G*_19×19_ has a total of 236 directed links and thus a graph density of $\rho_{19}=\frac{236}{19\times 18}=0.69$, similar to the interareal networks in the macaque and the mouse [19,20]. The 125-node network *G*_125×125_ has 2,906 directed links and a graph density of $\rho_{125}=\frac{2906}{125\times 124}=0.19$, significantly lower than the coarse version.

**Fig 2:**
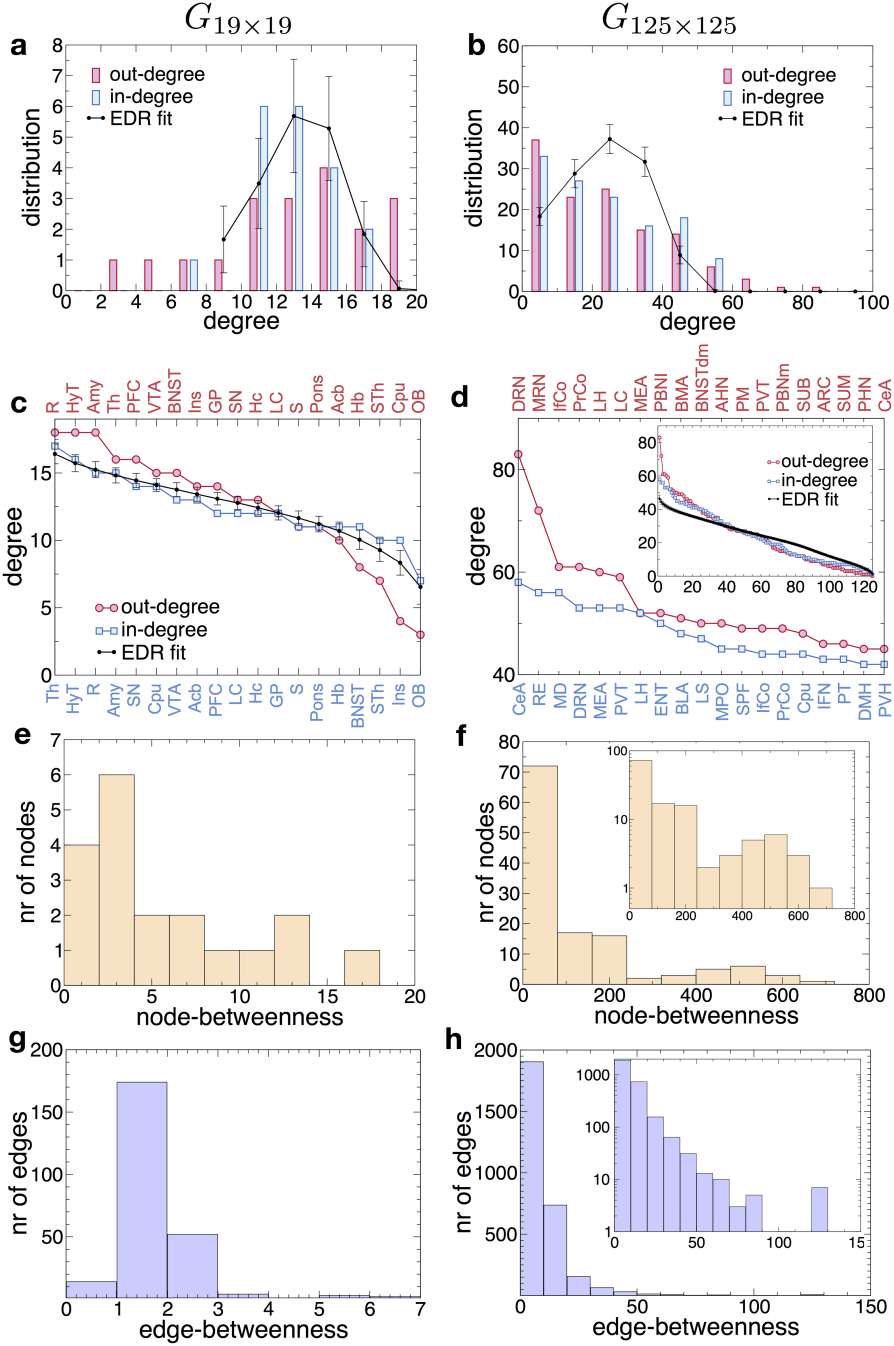
Graph properties at the two resolution levels, *G*_19×19_ and *G*_125×125_. (a,b) In- and out-degree distributions and the fit obtained through the exponential distance rule (EDR) model (being a random model provides the same distribution for in- and out-degree). (c,d) Degree ranking and its EDR fit. Nodes are listed as function of their in- (top, red) and out-degree ranking (bottom, blue). In (d) only the first 20 nodes are listed, the inset shows the whole ranking plot for all 125 nodes. (e,f) Histogram of node-betweenness values. (g,h) Histogram of edge-betweenness values. Insets in (f,h) show the histograms on log-linear scale.

Both graphs are relatively small and the degree distributions are noisy (Fig 2a and 2b), hence it is difficult to identify these distributions by simple fitting. However, below we present a model that generates predictions for these distributions (black lines in Fig 2a and 2b). Plotting the degree sequence in Fig 2c and 2d (rank ordering the nodes by their in- and out-degrees), we observe the existence of a small number of high degree nodes (hubs) receiving and/or sending many connections to the rest of the network. The BNST comprises 21 distinct brain areas, which are responsible for integration of limbic information and valence monitoring, processing threat reaction, fear, anxiety, and many other functions, collect 178 in-links, and project 365 out-links to the rest of the connectome. The dorsal raphe nucleus (DRN) has the largest number of outgoing projections (83) and the fourth largest of incoming links (53). This correlates with the fact that the DRN is the largest provider of serotonin innervation to the rest of the brain. Similarly, infra- and prelimbic cortices that comprise prefrontal cortex have high out-going projections (each 61) and a relatively high total degree of 105. This corresponds with the key role of prefrontal cortex in regulating cognitive functions. Moreover, another hub is the lateral hypothalamic area (with 52 in-degree and 60 out-degree), which is responsible for a significant array of functions, such as feeding behavior, wakefulness, thermoregulation, gastrointestinal functions, energy homeostasis, and visceral functions.

Supplementary tables show the top list of degrees (S1 and S2 Tables) and betweenness centralities (b.c.s; see Methods) for nodes (S3 and S5 Tables) and edges (S4 and S6 Tables) at both resolution levels, and Fig 2e–2h show the distributions of the betweenness values. According to these lists, DRN has the largest node betweenness, and that, expectedly, correlates with its high in- and out- degrees, a property that holds in general, although with some exceptions, such as the central nucleus of the amygdala (CeA). CeA, with the second highest b.c., has the largest in-degree, but it is only 19th in the out-degree list. The fact that it plays a key role in information transmission and processing resonates with the observation that it is a principal area for controlling emotional reactions.

**Fig 3:**
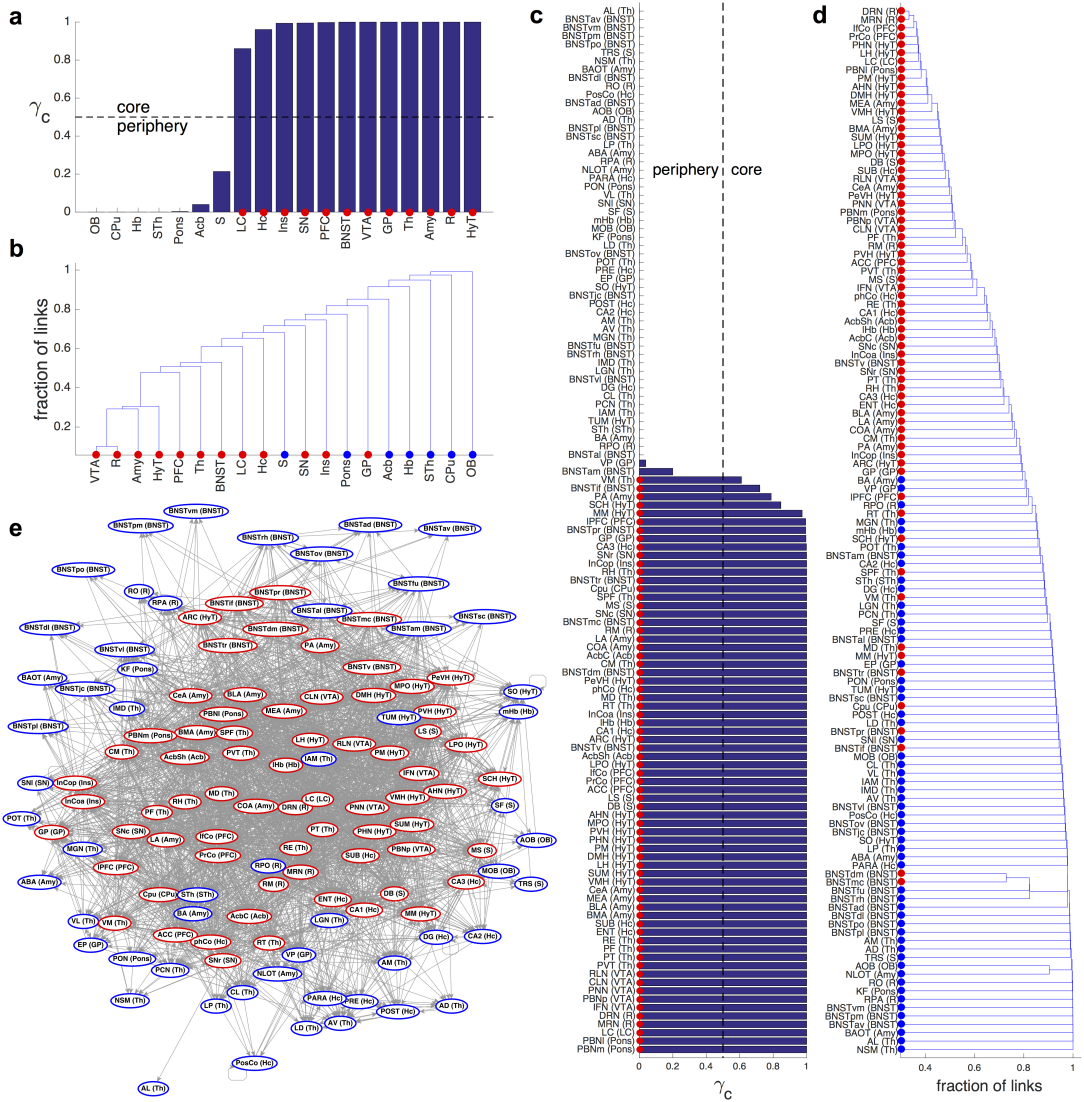
Core-periphery structure at the two resolution levels, *G*_19×19_ and *G*_125×125_. (a,c) Results found by the stochastic block model, where *γ*_*c*_ is the probability for a node to belong to the core. Core nodes, with *γ*_*c*_ > 0.5, are labeled with red dots. (b,d) Dendrograms showing the hierarchical decomposition of the networks based on the Girvan-Newman algorithm. The core nodes (labeled with red dots) are located towards the inside of the onion structure, while the periphery nodes (labeled with blue dots) are in the outer layers. (e) Graphical representation of the *G*_125×125_ network using a force directed layout. Core nodes are concentrated towards the center. For the lists of core areas at the two resolution levels, see S13 Fig.

The distributions of both node (Fig 2c and 2d) and edge betweenness (Fig 2e–2h) show a concentration at large values, suggesting a heterogeneous network structure; in particular, there are 8 edges with exceptionally high b.c. Given that for both macaque and mouse the interareal network is heterogeneous and shows a strong core-periphery structure [19–21], we have analyzed whether the same holds in the rat brain as well. By using a stochastic block modeling [22,23] method (see Methods section), we determined the probabilities of the individual nodes to belong either to the core or periphery (Fig 3a–3c). In Fig 3 the core nodes are colored red while the periphery nodes are blue. It shows a clear separation of the two classes in a similar fashion to the macaque and mouse. For *G*_19×19_ the core contains 12 nodes with an internal density of *ρ*_*cc*_ = 0.87, a periphery of 7 nodes of internal density *ρ*_*pp*_ = 0.24, and the subgraph of edges running between core and periphery nodes of density *ρ*_*cp*_ = 0.66. For *G*_125×125_ the core has 69 nodes with a high internal density of *ρ*_*cc*_ = 0.41, a periphery of 56 nodes of internal density *ρ*_*pp*_ = 0.03 and the subgraph of edges running between core and periphery nodes of density *ρ*_*cp*_ = 0.12. The list of core areas and the corresponding regions (in the 19-node segmentation) they belong to is shown in S13 Fig. Fig 3e shows the location of core nodes within *G*_125×125_, using a force-based layout representation. Note that while this analysis clearly indicates the core-periphery organization of the connectome, it does not reveal its internal structure, in particular, the internal network communities, if any. To explore this aspect, we performed a hierarchical decomposition of the network using the Girvan-Newman algorithm (see Methods), which outputs a hierarchical community dendrogram [24]. The results are shown in Fig 3b and 3d for both resolution levels. They indicate that in contrast with social networks, which have a clustering of communities over several levels, the rat connectome (at either resolution) has an onion-type structural organization [25], in which layers of areas are added on top of the previous ones. On the *x* axis, we colored the areas according to their core-periphery membership, which correlates well with depth in the dendrogram, with core nodes concentrating towards the inside of the onion structure. While this overall onion structure is a direct consequence of high-network density, the membership of the levels from the innermost to the outer layers is highly specific to the network.

Although the densities of *G*_125×125_ and its core are not drastically different (0.19 versus 0.41), at this resolution such difference implies significant specificity. Specificity can be estimated by computing an upper bound to the probability that an Erdos-Renyi random graph on 125 nodes of density *p* = 0.19 will have a core on 69 nodes of internal density *ρ*_*cc*_ = 0.41. The computation is shown in the Methods section, giving a very low probability of approximately 10^−228^, showing that the rat connectome core-periphery structure is, indeed, highly specific.

The raphe nuclei, DRN and medial raphe nucleus (MRN), constitute the deepest core of the dendrogram or the heart of the onion structure. These serotonergic systems play an important and generalized role in regulation of sleep-wake states and behavioral arousal. While MRN is mostly involved in stress- and anxiety-related processes [26], the DRN is critically involved in the neuronal regulation of circadian rhythms and sleep [27], which may support the hypothesis that DRN acts as a pacemaker of the network.

### Network control, driver sets

**Fig 4:**
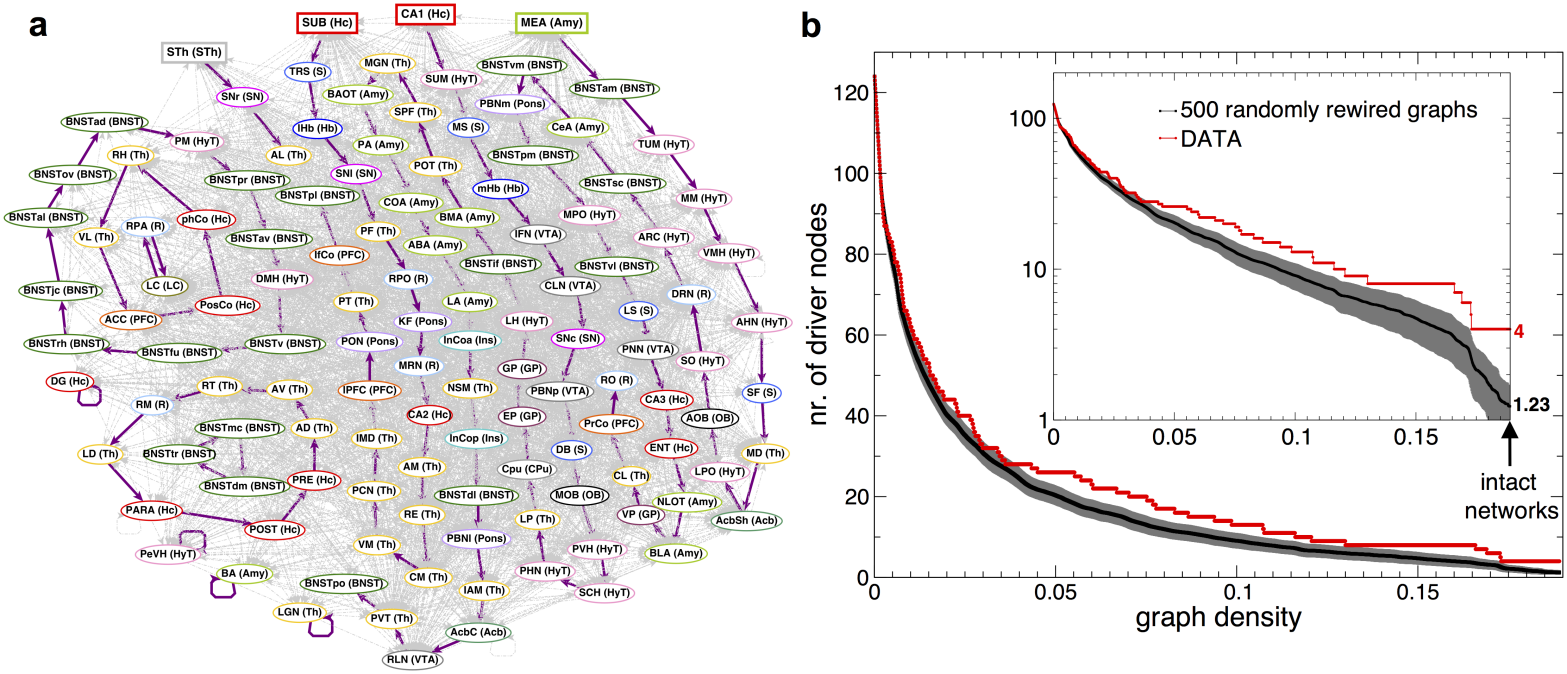
Driver nodes and maximum matching in the *G*_125×125_ network. (a) Purple edges show the links, which belong to the maximum matching and the driver nodes are highlighted as rectangles. The nodes are colored according to the brain regions they belong to. (b) The number of driver nodes as function of graph density, while sequentially removing the longest links from the *G*_125×125_ network (red curve), starting from the intact network. Degree-preserving randomizations/rewirings were done on the intact network, generating 500 randomized graphs. The edge removal process and driver node measurements were repeated on each of the randomized versions (average: black curve, SD: gray area). The inset shows the same figure but on a log-scale for the *y* axis.

A prerequisite of controlling a complex biological system is its structural controllability [28–30]. As another example analysis, here we identify the minimal set of driver nodes that could be used to control the system, through searching for a maximum matching in the graph (see definitions in Methods section). A realization of maximum matching is shown in Fig 4a with four driver nodes: subthalamic nucleus (STh), subiculum, CA1, and medial nucleus of amygdala. The interesting finding here is the role of STh in the structural controllability of the rat brain. Numerous deep brain stimulation studies have already shown the remarkable effects of activation of neurons within STh on global circuit dynamics [31] and in the treatment of Parkinson disease and other disorders [32]. This observation suggests an agreement between structural and functional controllability of neuronal networks and attracts our attention towards hippocampal and amygdaloid regions as potential targets for deep brain and other stimulation techniques.

While the number of driver nodes (4) is small compared to the total number of 125 nodes of the network, it is still significantly larger than in random networks with similar density. To show this we performed 500 randomizations of the network preserving the degree sequence and obtained an average of 1.23 driver nodes (SD: 0.45). Sequentially removing the longest (weakest) edge from the network, the number of driver nodes increases, as expected, exponentially (Fig 4b). However, the number of driver nodes for the original network, is consistently larger than for its degree preserved randomized versions. Thus, the original network structure is such as to allow for more points of control in the network, that is for a larger control diversity, than in a similar random graph with the same degree sequence.

### Modeling the rat connectome with an EDR network model

**Fig 5:**
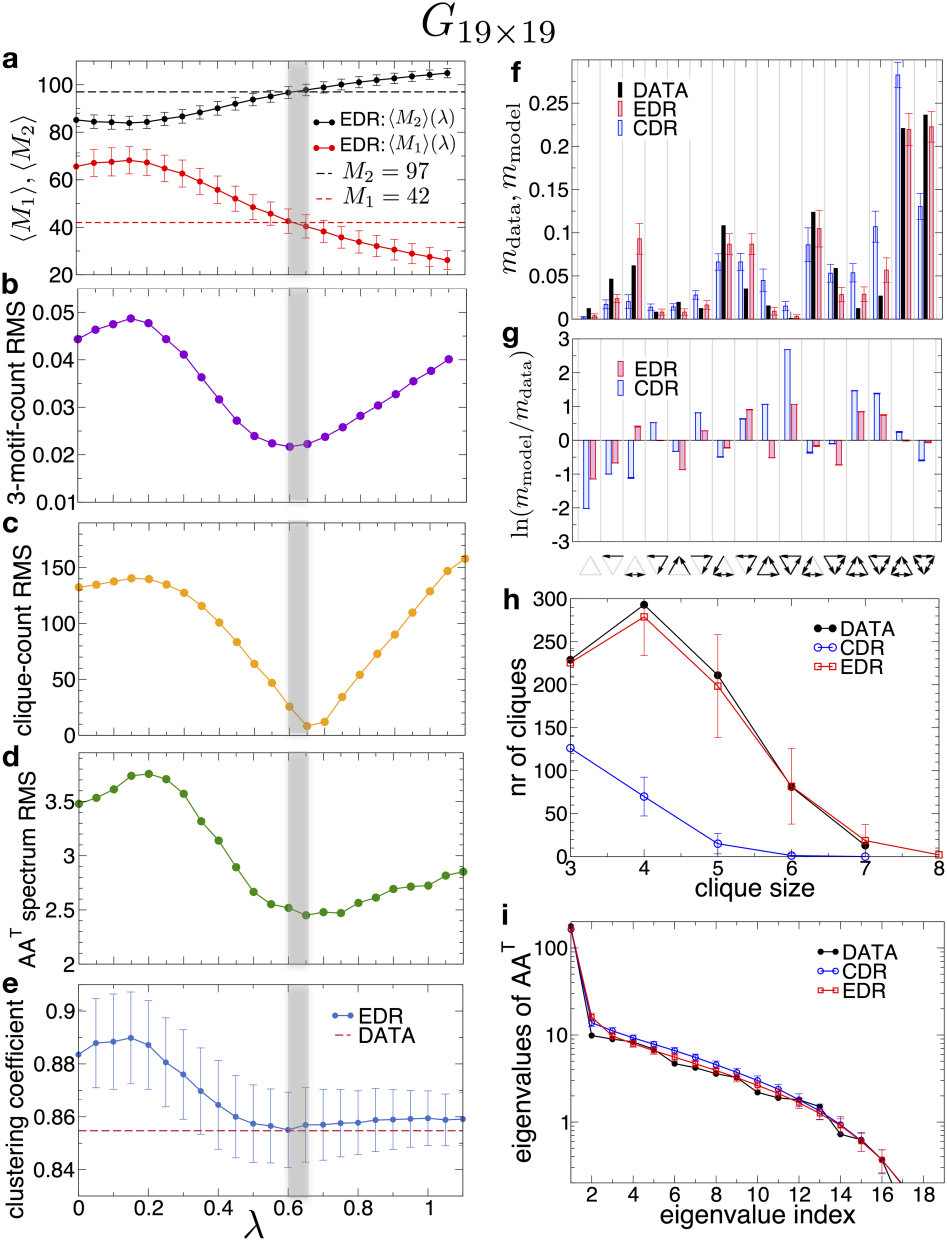
Modeling the *G*_19×19_ with an exponential distance rule (EDR) network. (a-e) Matching graph properties between model and data as function of the *λ* parameter. The vertical grey band indicates the range of optimal fit for: 0.60 − 0.65*mm*^−1^. Averages were taken over 1,000 model-generated networks for each value of *λ*. (a) The number of uni- and bidirectional links *M*_1_ and *M*_2_, respectively. (b) Root-mean-square (RMS) of deviations between the 3-motif counts of model and data. (c) RMS of deviations between the clique-counts of model and data. d) RMS of deviations between the eigenvalues of *AA*^*T*^ in the model and data, where *A* is the adjacency matrix. (e) The average local clustering coefficient in the undirected version of the graph. Dashed lines in (a) and (e) indicate values measured on the dataset. (f,g) Comparison of 3-motif counts between the dataset, EDR model with optimal *λ* = 0.6, and constant distant rule (CDR) model (*λ* = 0). (g) Log-ratios between motif counts. (h) Number of cliques with different sizes in data, EDR, and CDR models. (i) The eigenspectrum of the co-occurrence matrix *AA*^*T*^.

Consistent, cortex-wide retrograde tracer injections in several species (macaque [20,33]), mouse and microcebus [19] have shown that the distribution of the lengths of white matter (WM) axons follow an exponential decay, called exponential distance rule (EDR), with a species-dependent decay rate (λ). Naturally, 2 questions arise: Is the rat large-scale connectome also described well by the EDR network model? And (2) Does the agreement with the EDR model break with increasing resolution? The second question could not be answered for the other species, as there is no (nearly) edge-complete database available at higher resolutions for them, and thus ChemNetDB is especially valuable in this regard. By using ChemNetDB, we next attempt to answer both questions by modeling the connectome with the EDR network model at the *G*_19×19_ and *G*_125×125_ levels, respectively. Network comparison is based on parametric property matching described briefly in the Methods section and in detail in refs [19,20]. Fig 5 shows the results for the *G*_19×19_ using a set of commonly used graph measures: number of uni- and bidirectional edges (Fig 5a), the root-mean-square (RMS) of deviations for the 3-motif counts between model and data (Fig 5b, 5f and 5g), the RMS of clique-count deviations between model and data (Fig 5c and 5h), the RMS of the deviation of the eigenvalues of the co-occurrence matrix *AA*^*T*^ between model and data networks (Fig 5d and 5i) and finally the clustering coefficients (Fig 5e). These results show that all models generate the same value for the decay rate λ in the range 0.60 − 0.65*mm*^−1^ consistently, indicating that the EDR network model is a good model for the rat connectome at the large-scale level. Fits generated by and EDR with *λ* = 0.6*mm*^−1^ are shown in Fig 2a and 2c for the degree distributions and the degree sequence, respectively.

**Fig 6:**
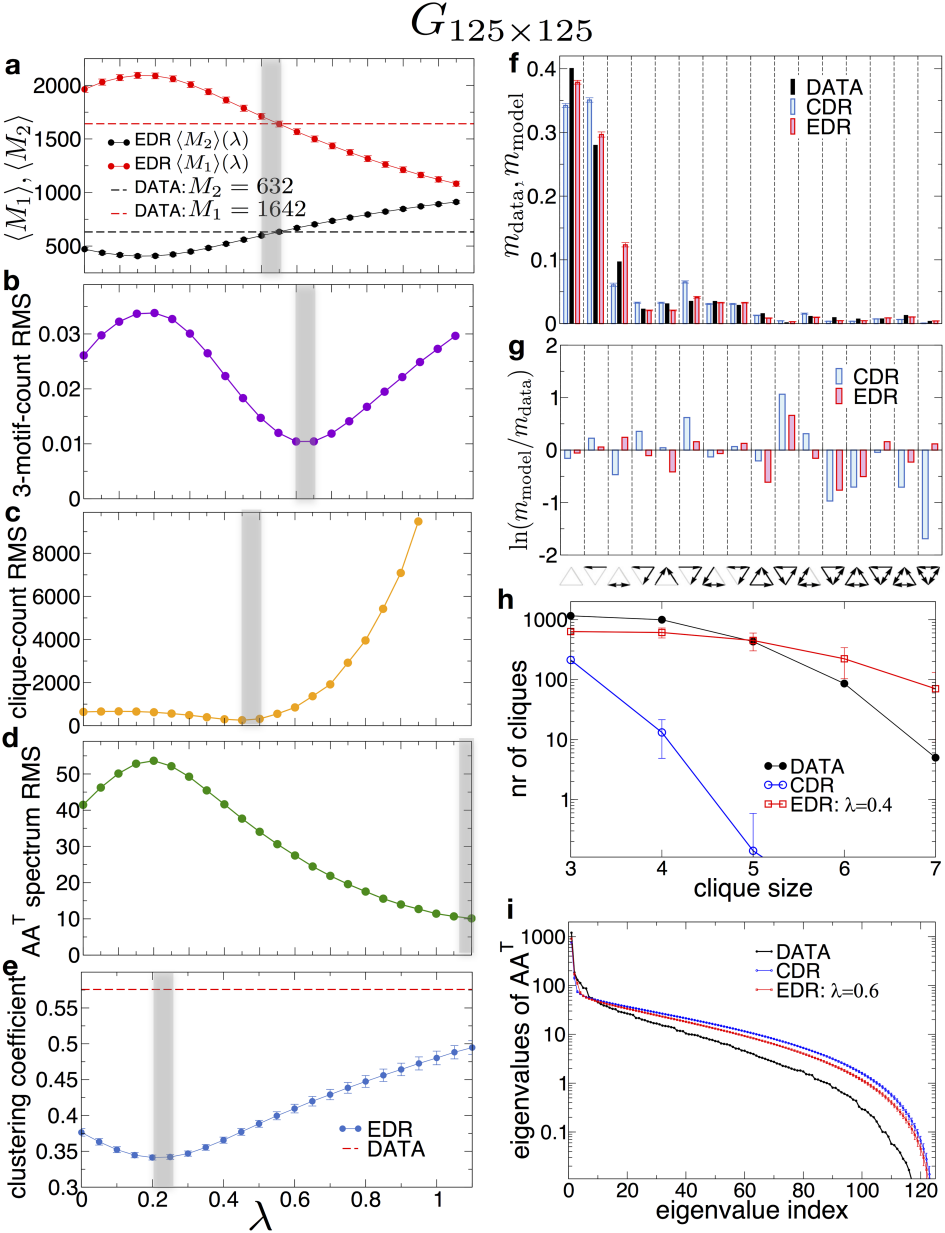
Modeling the *G*_125×125_ with an exponential distance rule (EDR) network. (a-e) Matching graph properties between model and data as function of the *λ* parameter. There is no narrow common band for *λ*, where all properties would match closely with the data. Averages were taken over 1,000 model-generated networks for each value of *λ*. (a) The number of uni- and bidirectional links: *M*_1_ and *M*_2_. (b) Root-mean-square (RMS) of deviations for the ratio of the 3-motif counts between model and data. (c) RMS of deviations between the clique counts for the model and data. (d) RMS of deviations between the eigenvalues of *AA*^*T*^ in the model and data. (e) The average local clustering coefficient in the undirected version of the graph. Dashed lines in (a) and (e) indicate values measured on the dataset. (f,g) Comparison of 3-motif counts between the dataset, EDR model with *λ* = 0.6, and CDR model (*λ* = 0). (g) Log-ratios between motif-counts. (h) Number of cliques with different sizes in the data and then the EDR and CDR network models. (i) The eigenspectrum of the co-occurrence matrix *AA*^*T*^.

Moving to the higher resolution connectome *G*_125×125_, the same procedure yields the comparisons shown in Fig 6. The figures now show a different picture: the best *λ* values determined from parameter matching are varying, from measure to measure, indicating that a single λ-parameter EDR model cannot describe the whole data network at this resolution. The reason lies with the fact that once we subdivide brain regions (as it was done in going from 19 areas to 125), there will be an increasing number of area pairs that are connected by gray matter (GM; nonmyelinated) connections, instead of WM connections. Note that at the G_19×19_ level, the connections are WM connections, just as for the cortical interareal networks in the mouse and macaque, where the EDR descriptions work well. As shown by experiments presented in Horvát et al. [19], the decay rate is sensitive to the nature of the medium in which the connections are running (WM versus GM). In particular, while local (GM) connections obey an EDR with almost the same decay rate (4.6 ÷ 4.9*mm*^−1^) in macaque, mouse and rat (see Fig 11B in [19]), for WM connections, the EDR decay rate decreases with increasing brain size (0.19*mm*^−1^ for macaque, 0.8*mm*^−1^ for mouse, and 0.6*mm*^−1^ for rat—this paper). The larger the brain, the smaller the decay rate for nonlocal (WM) connections. The EDR network model is defined via a single decay rate and it works well for all those brain networks for which the connections obey a single parameter EDR. Once the network contains both types of connections (GM and WM), as the rat brain at 125-area resolution, a single decay rate model will not work as shown in Fig 6. To develop a two-parameter EDR model, however, we also need to include information about the location of the GM connections with respect to the WM ones, resulting in a more involved model, which will be the subject of a forthcoming paper.

## Discussion

Most studies in neurobiology rely on a precise understanding of the neuronal connectivity and its neurochemical actors. Yet, investigators commonly face massive data that require enormous resources to be processed for their demands. By utilizing advanced neuroinformatics, our study resolves this problem and integrates over 50 years of neuroanatomy research on rat brains into a consistent multiscale, multilayer neurochemical cerebral connectome. Supervised machine learning was applied to resolve nomenclature issues resulting in an extensive standardized database, which combines the state-of-the-art knowledge of connectomics and neurochemistry. Establishing the neurochemical connectivity database (ChemNetDB) is a novel approach towards topological mapping of the brain that takes the neuroconnectomics well beyond the binary constructs and paves the way for more advanced and accurate investigations of healthy and disease states of rat brains.

We have investigated, as examples of analytic studies that could be done on the database, several network measures and their relationships with similar analyses in macaque and the mouse. Analysis of the structural properties of the resulting network reflect and confirm the key functional roles of deep subcortical brain areas such as lateral hypothalamus, BNST, and DRN. These regions are known to be responsible for primitive yet essential and evolutionary conserved functions, such as regulation of sleep and circadian rhythms, reward, anxiety, aggression, and fear. The importance of these brain areas for survival is also associated with an early formation in the developmental stages as reflected in the connectome by their extremal values of various graph connectivity measures such as b.c. and/or node degree.

Previous theoretical analysis demonstrated that the optimal core-periphery structures in networks that are stable against both random and targeted failures/attacks (targeting hubs) are onion-like structures [34], however, they have not been observed in real-world networks [25]. Our analysis shows that the onion structure occurs in large-scale, dense brain neuronal networks, where robustness against information transmission failures (of all types) is a critical requirement. The observed onion structure does not contradict the well-known hierarchically modular structure of brain networks. At this large-scale resolution, applying the Girvan-Newman methodology [24], the paths-based network modularity is not revealed due to the high density of the network. Instead, the onion organization is dominant and the community structure appears as core-periphery at this scale.

As another example of an analytical study on ChemNetDB, we tested whether the EDR property and the associated network model are consistent with the rat connectome structure, at different levels of resolution. The EDR, expressing economy of wiring has been found to hold in both macaque and mouse for both mesoscale WM connections and local GM connections. While direct measurements of the WM axon length distribution in the rat are currently lacking, the EDR network is highly consistent with the large-scale ChemNetDB connectome, suggesting that the whole-brain WM connectivity is also strongly determined by the EDR.

By using maximum matching algorithms, we have identified 4 nontrivial driver nodes of the network. In particular, STh appears as a feasible candidate to be involved in control mechanisms of the brain. However, the actual biological interpretation of driver nodes is not yet clear and the role of the identified brain regions in controlling brain activity requires further investigations and experimental validations.

Note that the outcomes of the example network analyses presented here might change with the addition of new data; however, the main observations, such as the strong core-periphery structure and network measures expressed as fractions (including density, the fraction of 3-motifs, etc.), are expected to be robust against changes due to the high density of the networks.

ChemNetDB provides the first, whole-brain, large-scale and consistently collated rat connectome database that also includes neurochemical specificity. This will provide researchers with a tool to gain insights into the fundamental relationships between connectome architecture, information processing, and brain function, with potential for advancing preclinical research and clinical applications such as those related to substance abuse and depression.

## Methods

### Standardized data mining and database creation

#### Search strategy

The online portal of the National Library of Medicine (http://www.ncbi.nlm.nih.gov/pubmed/) including PubMed, PubMed Central, and MEDLINE was used as the platform for literature research. A systematic screening of the original research articles published until February 2015 was performed based on 1,750 keywords (see S2 Text). In addition, the reference sections of identified papers as well as review and meta-analysis articles were screened for further relevant citations. For hippocampal formation, the search strategy was further extended to include the hippocampome [10].

#### Study selection

Reviewers, in pairs, independently screened titles and abstracts of articles and reviewed the full text of any title or abstract deemed potentially eligible by either reviewer. Reviewers resolved disagreements by discussion. Among these studies, only peer-reviewed original research articles in English language were chosen for data mining if they provided consistent information on chemoarchitecture, cytoarchitecture, cerebral intra- or interregional connectivities, mRNA expression levels, and/or receptor distribution densities. Thereby, only studies conducted on rats not associated with any disease-model, geno- or phenotype were included, which used anterograde tracing with radioactive amino acids, and autoradiographic fiber tracing methods, as well as anterograde and retrograde tracing with horseradish peroxidase, in situ hybridization, immunohistochemistry or immunocytochemistry as applied technique. Tracking tracing and immunohistochemical studies in animals other than rats or in cell cultures were excluded. Functional connectivity as measured by magnetic resonance imaging or electrical stimulation was excluded as the correspondence between anatomical projections and functional connectivity is still an open question. Optogenetics is most likely the right compromise. This modern technique, however, lacks standardized experimental and reporting protocols and has only rarely been used to investigate the rat neuroanatomy under basal conditions. Thus, there is an insufficient number of studies to conduct a robust systematic review of the literature and therefore observations based on this valuable technique are not included in the present version of the database. Abstracts and unpublished studies were not included. Authors were contacted if critical information was missing or only partially provided in their articles.

#### Outcomes and effect modifiers

The primary outcome was a set of multiscale connectivity matrices of brain regions containing intra- and interregional pathways. This set contains several adjacency matrices each associated with a specific transmitter system. The topological union of these matrices represents the unweighted adjacency matrix of the cerebral connectome. Secondary outcomes were neuron type, number of neurons, and distribution density of receptors within brain regions. Age, weight, gender, strain, number of rats, and the tracing markers were considered as effect modifiers (see Sensitivity Analysis).

#### Data extraction

Although the neuroanatomical literature is remarkably information-rich, neuron properties are often reported with incompletely defined and notoriously inconsistent terminology, creating a formidable challenge for data integration. In order to avoid artificial bias in data mining due to differences in nomenclature, the reviewers did not use any a priori parceling or data filtering techniques. If a research article fulfilled the inclusion criteria, then the 4 categories of variables, including the definition of the brain area as provided by the authors, were extracted and implemented into a standardized template.

Category I—Variables concerning cytoarchitecture and morphology: types of neurons; percentage share of different neurons; number of neurons; given nomenclature of brain region components.

Category II—Variables concerning topology: efferent connections; afferent connections.

Category III—Variables concerning neurotransmitter and receptors: local receptor distribution density; mRNA expression levels; neurotransmitter systems.

Category IV—Variables concerning utilized methods and animals: utilized tracer-technique; tracing markers; strain, age, weight, gender, and number of rats.

#### Quality assessment

Two factors may significantly influence the quality of the dataset: (1) differences in the nomenclature and parceling schemes and (2) the accuracy, reliability, and completeness of pathway tracing data. Neuroinformatics tools provide appropriate strategy to investigate inferences due to different parceling schemes and/or insufficient data. Here, we used divisive hierarchical algorithms with constraints for the preprocessing of the multivariate datasets to reduce the dimension of the network and compare the structural differences with respect to different parceling schemes. Edge-completeness of the network was the defining constraint. It excludes regions from clustering which only receive or only send neural projections. An exception to this rule is given if a network node belongs to a well-defined functional unit. The anterolateral nucleus of thalamus represents the only case that the exception was made.

There were 93 nomenclature inconsistencies. Here, we used a supervised machine learning technique to identify synonymous brain areas with respect to cytoarchitecture. Thereby, a given morphology is associated with one well-defined equivalence class for brain regions of different denotations. These equivalence classes were used as the input data for the structural preprocessing of the database. For algorithmic realization, the “LPBOOST” and “RUSBoost” (combined with “AdaBoostM2”) ensemble learning method “fitensemble” in MATLAB were used, which allow for the melding of results from many weak learners into one high-quality ensemble predictor, particularly appropriate for classification of skewed data (many more observations of one class).

If data on the existence of the pathways were not complete, then the related brain regions and connectivity patterns were treated as temporarily unreliable for network analysis and were excluded from the connectome. Nonetheless, all data are preserved within the database and each efferent and afferent matrix contains a section “other regions” that includes this additional connectivity data. This section not only contains the data from different parceling schemes but also data on cerebellum, nerve fibers, and components of medulla. Furthermore, it includes data on connectivity of cerebral areas for which not enough information is available at the current stage (auditory cortex, Barrington nucleus, caudal temporal cortex, central linear nucleus of raphe, [anterior and posterior] cingulate cortex, claustrum, cuneiform nucleus, dorsal peduncular cortex, dorsal/lateral and dorsolateral tegmental nucleus, ectorhinal cortex, Edinger Westphal Nucleus, endopiriform cortex, Forel fields, frontal cortex, frontal polar cortex, frontal motor cortex, gustatory cortex, infraradiata cortex, interpeduncular nucleus, lateral terminal nucleus of the accessory optic system, lateral/medial/ventral orbital cortex, lateral/vental occipital cortex, lateral precentral cortex, median eminence, mesencephalic reticular nucleus, nucleus ambiguus, motor trigeminal nucleus, nucleus basalis of meynert, nucleus incertus, nucleus of Darkschetwisch/Cajal, olfactory tubercle, orbitofrontal cortex, parasubthalamic nucleus, parastrial nucleus, parietal cortex, pedunculopontine nucleus, periamygdaloid cortex, periaqueductal gray, piriform cortex, pretectal area, primary motor cortex, primary somatosensory cortex, primary visual cortex, red nucleus, reticular formation, retrosplenial cortex, secondary somatosensory cortex, sensorimotor cortex, somatic motor cortex, subfornical organ, superior colliculus, temporal cortex, trigeminal motor nucleus, visual cortex, zona incerta). This procedure ensures the high quality of the network in a quantitative hypothesis-free manner.

#### Integration of data into a network structure

The remaining brain regions and their neurochemical efferents and afferents were compiled into a cerebral neurochemical connectome of the rat brain including 125 brain areas. Micro-, meso- and macroscale connections within and between brain regions were mapped into a 3-dimensional space using a standardized platform to generate a comprehensive and quantitative database of inter-areal and cell-type−specific projections. Weights of connections only by means of the transmitters they utilized. The length of each connection was calculated as the Euclidian distance between the geometric centers of the brain regions as defined by Paxinos atlas [35].

#### Recursive coarsening and network partitioning

Within the framework of traditional neuroanatomical studies, a large number of the connectome comprising brain regions can locally be compiled into functionally distinct brain regions. To simulate this complexity reduction and its consequences on the network properties, we performed a recursive coarsening of the connectome by rating its edges and contracting the edges prioritized by the rating [36]: (1) we defined weights of the edges in a binary manner such that the weight of intraregional connections were assumed 0 and 1; (2) a minimum weight spanning tree T^m^ was computed; and (3) the network edges were rated based on conductance values of T^m^ fundamental cuts. This computational approach results into a 19-node network congruent to the neurocircuitry for modeling drug effects [12].

#### Sensitivity analyses

To ensure the robustness of the connection matrix of the neurochemical connectome, OFAT (one-factor-at-a-time) sensitivity analyses were performed a posteriori and the impact of the effect modifiers (age, gender and strain) was investigated, if possible. Yet, a projection is in many cases only reported by a single yet reliable study and constitutes an entry of the connection matrix. Therefore, a systematic analysis is not possible at this stage. However, the skewness of distribution density of experimental parameters provides robust validity conditions for the representativeness of the network as a cerebral connectome of adult, male, Sprague-Dawley rat.

#### Network measures

The definitions below are given for completeness, the precise mathematical expressions can be found in the network literature [37].

#### Degree

The total degree, *k*_*i*_ of node *i* is the number of its connections/links. We call in-degree/out-degree (*k*_*i*_^in^ and *k*_*i*_^out^) the number of incoming/outgoing directed edges. From these definitions it follows that: *k*_*i*_ = *k*_*i*_^in^ + *k*_*i*_^out^.

#### Density

In a directed graph with *N* nodes/vertices and *M* links/edges, it is the ratio between the number of existing links (*M*) and the number of all possible connections, i.e.:

$$\rho =\frac{M}{N\left(N-1\right)}.$$

#### Number of unidirectional links

*M*_*1*_ in the graph is the number of node-pairs (*i* and *j*) that are connected only with a single directed link, from *i* → *j* or from *j* → *i*.

#### Number of bidirectional links

*M*_*2*_ is the number of node-pairs that are connected with a pair of oppositely oriented links. Thus, the total number of links *M = M*_*1*_ *+ 2M*_*2*_.

#### Undirected form of a directed graph

The directions of the links are discarded and the double links are replaced by single links.

#### Local clustering coefficient

The local clustering coefficient of a node in an undirected graph is defined as the fraction between the number of pairs of its connected neighbors and the total number of pairs of its neighbors. For a directed network we first generate its undirected version. The global clustering coefficient is defined as the average of local clustering coefficients over all nodes.

#### Cliques

Cliques are fully connected subgraphs, with all possible edges present between its nodes. A clique with k nodes in a directed graph has *k*(*k* − 1) directed links.

#### Motifs

These are all the possible link configurations between a set of *k* nodes, including absence of any link and link orientation. When *k* = 3 the total number of 3-motifs is 16. Their number, and more precisely, the over- or under-representation when compared to their abundance in a random graph is indicative of organizational and functional specificity in the network.

#### Directed shortest path length, average (directed) path length and diameter

The shortest path between 2 nodes in a network is a shortest sequence of consecutive, directed edges going from 1 node to the other following the directionality of the edges. The average path length is the average value of shortest paths for all ordered node pairs. The diameter of the network is the largest length of all the directed shortest paths in the network.

#### Betweenness centrality

The b.c. of a node (edge) is the fraction of all shortest directed paths that are running through that particular node (edge).

#### Eigenvalue spectrum

Relates to the eigenvalues of the adjacency matrix *A*. However, in a directed network the adjacency matrix is nonsymmetric and thus it can admit complex eigenvalues as well. Typically, for directed networks one looks at the so-called co-occurrence matrix *AA*^*T*^, which is symmetric and thus it has only real eigenvalues.

#### Core-periphery detection

We used stochastic block modeling [22,23] to assign nodes to either the core (c) class or periphery (p) class with a given probability, *γ*_*c*_ and *γ*_*p*_, respectively. It is a generative graph model in which the connection probabilities *p*_*rs*_, *r*, *s* ∈ {*c*, *p*} depend on the chosen group (core or periphery) membership of the nodes. The *p*_*rs*_ form a 2 × 2 affinity matrix ***p*** = {*p*_*rs*_}. The goal is to find the parameters *γ*_*c*_, *γ*_*p*_, {*p*_*rs*_} by fitting, which is performed using maximum likelihood with an expectation-maximization algorithm (EM). Given an adjacency matrix *A* = {*A*_*ij*_}, the likelihood is given by:

$$P\left(A|p,\vec{\gamma }\right)=\underset{g_{1}}{\sum }\ldots \underset{g_{N}}{\sum }\underset{i<j}{\prod }{\left(p_{g_{i}g_{j}}\right)}^{A_{ij}}{\left(1-p_{g_{i}g_{j}}\right)}^{1-A_{ij}}\underset{i}{\prod }\gamma_{i}$$

Thus, we want to find the values of the parameters such as to maximize the log-likelihood. However, computing this sum precisely is usually intractable computationally and one has to turn to approximations such as Markov-chain Monte Carlo, belief propagation or Bayesian inference.

We used the software package given in [23] to find the core-periphery structure. The algorithm described in this paper uses variational Bayesian inference to fit the model parameters *γ*_*c*_, *γ*_*p*_, {*p*_*rs*_}, which is extended to work on weighted networks also. It involves a parameter *α* which is related to the proportion in which the algorithm should use weights in the process. By setting *α* to 1, the algorithm can be used for unweighted networks. For more details on the variational Bayesian inference, we refer the reader to Aicher et al. [23]. An alternate but less sophisticated EM algorithm gave virtually the same answer, with the exception of one area that was classified as periphery, not core.

Due to their high-density networks, in the previous cases [19–21] we used a clique-analysis based method to distinguish between core and periphery, however, that works suboptimally in sparser graphs, motivating the stochastic block modeling approach used here and described above for *G*_125×125_. At the lower resolution of *G*_19×19_, the cliques distribution based method can, however, be used, producing a core-periphery structure that contains all the core areas found from the stochastic block modeling based method (see S14 Fig).

#### Hierarchical decomposition

We used the Girvan-Newman algorithm [24] to break the graph into increasingly smaller parts. This algorithm uses the edge betweenness measure (see above). An edge with high b.c. has a large influence on information transfer through the network, which indicates that it is “between” 2 important clusters of nodes. At each step of the algorithm the edge b.c. is calculated for all edges, then the edge with the highest b.c. is deleted from the graph and finally, the strongly connected components are measured. The last task is done in order to check whether the removal of the edge caused a break-up in the graph. A strongly connected component is one where each node, which belongs to this component, can be reached through paths along directed edges from all other nodes located in the same component. These steps are repeated until all the edges are deleted. This process can be visualized as a dendrogram, where we start at the point when everything is connected and proceed by creating a new branch at every step when a component splits until we reach the node level, where every node is its own component. The dendrogram shows how the components split during this process, providing a visualization of graph structural hierarchy.

#### Core-periphery specificity calculations

Consider Erdős-Rényi directed random graphs on *N* nodes, of link-density *p* (i.e., the probability that a node *i* will have an edge directed to node *j* is *p*). We want to provide an upper bound on the probability that such a random graph will have a subgraph of *K* nodes of link-density *q*. Let us first fix (arbitrarily) a set of *K* nodes {*i*_1_, ⋯, *i*_*K*_} and consider the vertex-induced subgraph within the larger ER graph. If *L* = *K*(*K* − 1), then a density *q* implies *qL* edges within this subgraph. As each edge is generated by the ER process in the larger graph, it comes with probability *p*. Thus the probability that this subgraph on the specific set of vertices has a density *q* is just: $\left(\begin{array}{c} L\\ qL \end{array}\right)p^{qL}{\left(1-p\right)}^{\left(1-q\right)L}$. Adding these probabilities for all possible sets {*i*_1_, ⋯, *i*_*K*_} would then provide an upper bound to the probability for the existence of a subgraph on *K* nodes with density *q*, because not all choices {*i*_1_, ⋯, *i*_*K*_} generate independent subgraphs (the upper bound nature comes from the fact that we would have to subtract the probability of the overlap, according to the fundamental relation *p*(*A*∪*B*) = *p*(*A*)+ *p*(*B*)-*p*(*A*∩*B*)). Thus, an upper bound is given by $\left(\begin{array}{c} N\\ K \end{array}\right)\left(\begin{array}{c} L\\ qL \end{array}\right)p^{qL}{\left(1-p\right)}^{(1-q)L}$. For *G*_125×125_ we have *N* = 125, *K* = 69, *p* = 0.19, *q* = 0.41, giving a value of 4.9 × 10^−228^, extremely small.

Specificity of a dense network can further be probed by looking at its clique distribution. The core is formed by a denser subgraph, populated by a number of large-order cliques previously observed in the mouse, macaque, and now in the rat. In particular, the largest cliques in *G*_125×125_ have 7 nodes and there are 5 of them involving a total of 20 areas. Considering only the 162 edges participating in these 7 cliques, the upper bound on the likelihood in an ER graph is on the order of 10^−4^. However, there are actually 287 edges between these 20 nodes generating (by the method above) an upper bound of 10^−104^ for the same likelihood in an ER graph. Thus, from several angles, the core-periphery of the rat connectome is highly specific.

#### EDR based network modeling

The EDR can be thought of as a rule expressing the cost of long wiring. The EDR seems to be a universal feature of the wiring in the brain as local, i.e., GM projections (out to less than 2 mm), were also found to obey an EDR, with a decay rate that appeared to be almost identical between mouse and macaque [19]. Additional evidence [19,38] indicates that this local decay rate is the same in the rat as well; however, the same measurements for WM connections in the rat are presently lacking. Here, we describe a generative network model based on the EDR as a constraint on wiring lengths. Note that the EDR is a fundamental statistic on individual axonal lengths and it does not involve brain segmentations or areal definitions. However, given 2 brain areas, using the EDR we can estimate their connection strength based on the distance between those areas, as if determined by wiring costs alone. Thus, given a segmentation of the brain into areas, we can build an EDR-based network model of the connectome. Here, we describe the computational procedure of generating EDR networks, which are random graphs based on the EDR rule and the matrix of interareal distances. There is no apriori reason for why the connectome should be describable by an EDR family of random graphs, because the latter is only based on wiring costs and geometry. However, as demonstrated in refs [19–21] the EDR based network model does a good job in matching the statistical properties of the large-scale connectome in both the macaque and mouse. The exponential distance rule itself *p*(*l*)~*e*^−*λl*^ for the distribution of the axonal lengths (*l*) is a global/average statistic, it does not define a network connectivity model. The true connectome is the result of many factors and processes, with the EDR being only 1 of the constraints. However, due to its fast decaying nature, this EDR constraint may strongly determine many of the connectome’s graph theoretical properties, as it was already demonstrated to be the case for the large-scale interareal network in the macaque and mouse brains. To assess the degree to which the EDR determines the structure of the connectome, one follows Jaynes’ maximum entropy principle [20,39,40]: the network generation mechanism includes only the constraints of the EDR, the distance matrix (geometry), the total number of nodes and the connectome’s density. Everything else is determined uniformly at random so that uncontrolled biases are avoided. The procedure has been described in several places [19,20], but we briefly summarize it here for completeness. The construction algorithm proceeds as follows: (1) A distance value *l* is generated from the EDR distribution; (2) from the distance matrix we determine the set of area pairs whose distance falls into the same distance bin with *l*; (3) We choose uniformly at random a distance pair from this set and then throw a connection between them oriented at random with equal probability (*i* → *j*or *j* → *i*). 4) We repeat (1) through (3) until we reach the same binary connectivity density as in the data network. This results in a directed, spatially embedded and weighted random network, an EDR network. By using this procedure, we create many realizations of these EDR networks and then we average the graph measures over these realizations (ensemble average). Given a graph measure, or property *P*, we compute its ensemble average as function of the decay rate *λ* and we set the value of *λ* such that the ensemble average of *P* is as close as possible to its value in the connectome. We then record this *λ*_*P*_ value obtained by graph property matching. If there is a direct way of measuring *λ*, e.g., from retrograde tracing, then its value can be compared with *λ*_*P*_. If the 2 are close, then the EDR is a strong determinant of that property in the connectome. For the more properties *P* holds, the stronger the EDR is, as an overall determinant of the connectome’s structure. Note that in this case it follows that the *λ*_*P*_−s are close to one another for many different properties *P*. As a matter of fact, this agreement can be used as an indication of the validity of the EDR as a model of the connectome, even if the direct measurement of *p*(*l*) is not available from tracer experiments (as in our case, here). Note that there is no a priori reason for why the *λ*–s matched from different properties should be equal, and indeed this seems to be the case for the higher resolution network; most likely because either it involves several different EDR–s at this higher resolution or that at this level of resolution other, e.g., function related factors start playing more significant roles, and thus the simple, EDR based description needs to be augmented with these factors.

#### Network control, maximum matching, driver nodes

In order to control a networked system, one needs to identify the minimum set of driver nodes that, if driven by different input signals, can offer full control over the network [28,29]. This problem can be mapped into finding the maximum matching in a directed network, a well-known graph theoretical problem. In a directed graph, a matching is defined to be a set of directed edges (shown in purple in Fig 4) that do not share common start or end vertices. A node is matched if there is a purple link ending in that node. Unmatched nodes are called driver nodes. In case of maximum matching the number of matched nodes is maximal, meaning the number of driver nodes is minimal. The matching is a set of directed paths or cycles.

For identifying the maximum matching shown in Fig 4, we used the algorithm from the igraph C++ library. Even if there might exist other realizations, we can be sure that the minimum number of driver nodes is 4.

## Supporting information

S1 Fig Flowchart of data-mining and processing. (TIF)

S2 Fig mRNA density distribution of GABA_A_ receptors. (TIF)

S3 Fig mRNA density distribution of GABA_B_ receptors. (TIF)

S4 Fig mRNA density distribution of glutamatergic receptors. (TIF)

S5 Fig mRNA density distribution of dopaminergic receptors. (TIF)

S6 Fig mRNA density distribution of muscarinic acetylcholine receptors. (TIF)

S7 Fig mRNA density distribution of nicotinic acetylcholine receptors. (TIF)

S8 Fig mRNA density distribution of adrenergic receptors. (TIF)

S9 Fig mRNA density distribution of 5-HT receptors. (TIF)

S10 Fig mRNA density distribution of CRH receptors. (TIF)

S11 Fig mRNA density distribution of opioid receptors. (TIF)

S12 Fig mRNA density distribution of endocannabinoid receptors. (TIF)

S13 Fig Network core areas in G_125x125_ and G_19x19_. (TIF)

S14 Fig Network core areas in G_19x19_ using the cliques list. There are 13 cliques of (the largest) size 7 sitting on 14 nodes. All the 12 core areas found with the stochastic block modeling method (shown in red) are also found among these 14.
(TIF)

S1 Table Degrees of the G_19x19_ network ranked from maximum to minimum. (XLSX)

S2 Table Degrees of the G_125x125_ network ranked from maximum to minimum. (XLSX)

S3 Table Node-betweenness values of the G_19x19_ network ranked from maximum to minimum. (XLSX)

S4 Table Edge-betweenness values of the G_19x19_ network ranked from maximum to minimum (top 30 connections). (XLSX)

S5 Table Node-betweenness values of the G_125x125_ network ranked from maximum to minimum (top 30 regions). (XLSX)

S6 Table Edge-betweenness values of the G_125x125_ network ranked from maximum to minimum (top 30 connections). (XLSX)

S7 Table Intraregional connectivity matrix. (XLSX)

S8 Table Interregional efferent projection matrix. (XLSX)

S9 Table Interregional afferent projection matrix. (XLSX)

S10 Table Systematic review of brain-wide distribution density of NMDA, AMPA, mGluR, dopaminergic and cannabinoid receptors based on mRNA expression levels and receptor binding studies. —not detected; +very low/slight/scarce/scattered; ++low/weak; +++ moderate; ++++dense/high/intense; +++++very dense/very intense/most; X means that the receptors were present but not quantified.
(XLSX)

S11 Table Systematic review of brain-wide distribution density of opioid, GABA, CRF, serotoninergic and adrenergic receptors based on mRNA expression levels and receptor binding studies. —not detected; +very low/slight/scarce/scattered; ++low/weak; +++ moderate; ++++dense/high/intense; +++++very dense/very intense/most; X means that the receptors were present but not quantified.
(XLSX)

S12 Table Detailed systematic review of brain-wide distribution density of GABAergic receptors based on mRNA expression levels and receptor binding studies. —not detected; +very low/slight/scarce/scattered; ++low/weak; +++ moderate; ++++dense/high/intense; +++++very dense/very intense/most; X means that the receptors were present but not quantified.
(XLSX)

S13 Table Detailed systematic review of brain-wide distribution density of cholinergic receptors based on mRNA expression levels and receptor binding studies. —not detected; +very low/slight/scarce/scattered; ++low/weak; +++ moderate; ++++dense/high/intense; +++++very dense/very intense/most; X means that the receptors were present but not quantified.
(XLSX)

S1 Text Database references. (DOCX)

S2 Text Methodological details. (DOCX)

## Abbreviations

b.c.: betweenness centrality
BNST: bed nucleus of the stria terminalis nuclei
CeA: central nucleus of the amaygdala
DRN: dorsal raphe nucleus
EDR: exponential distance rule
GABAergic: gamma-amniobutyric acid-ergic
GM: gray matter
MRN: medial raphe nucleus
RMS: root-mean-square
STh: subthalamic nucleus
WM: white matter
