# Supplementary figures and images for: A multiscale cerebral neurochemical connectome of the rat brain
Source: PLoS Biol. 2017 Jul 3;15(7):e2002612. doi: 10.1371/journal.pbio.2002612 (PMC5507471; doi:10.1371/journal.pbio.2002612)

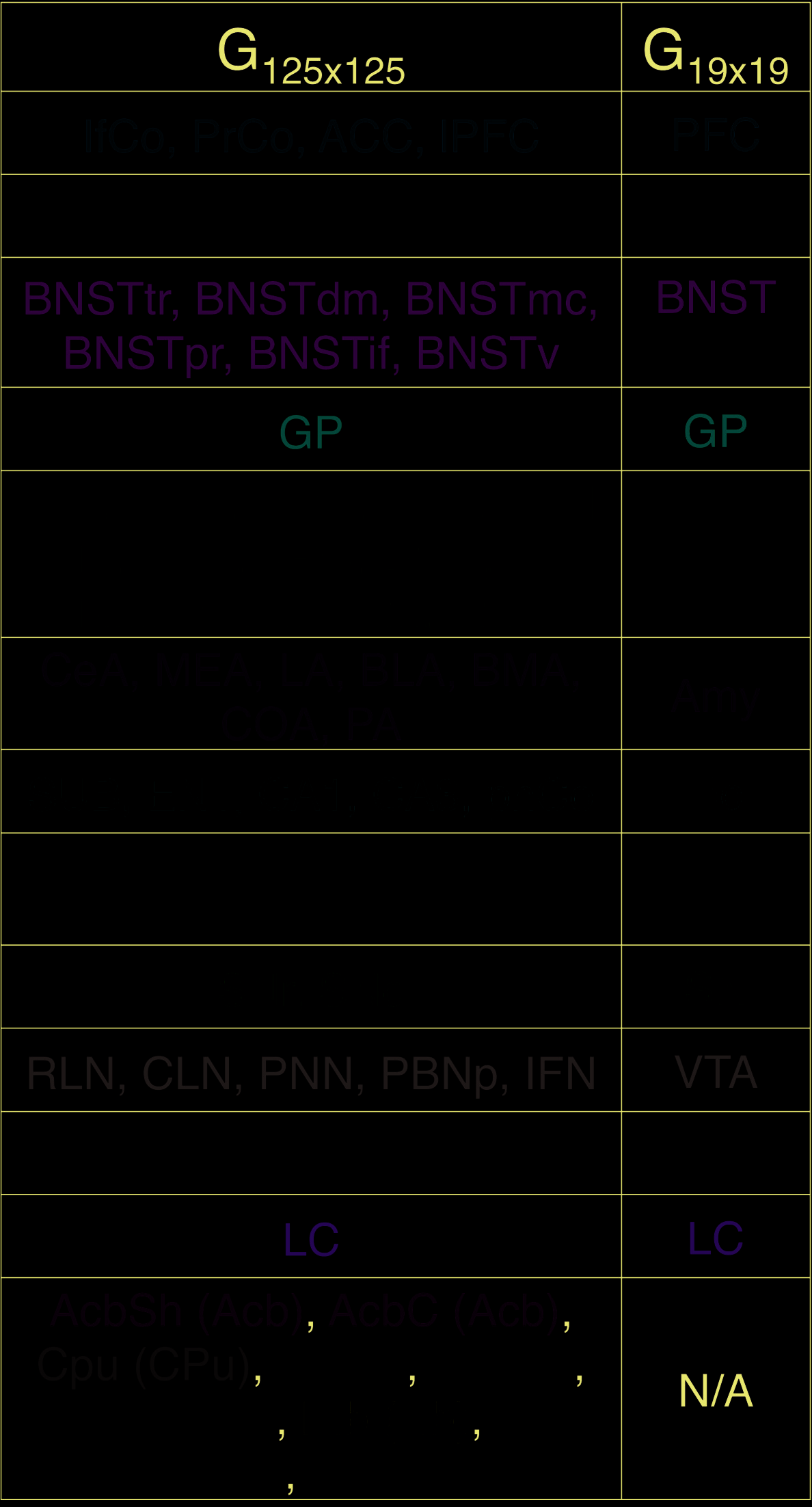

Supplement: S13 Fig — (TIF) [file pbio.2002612.s013.tif]

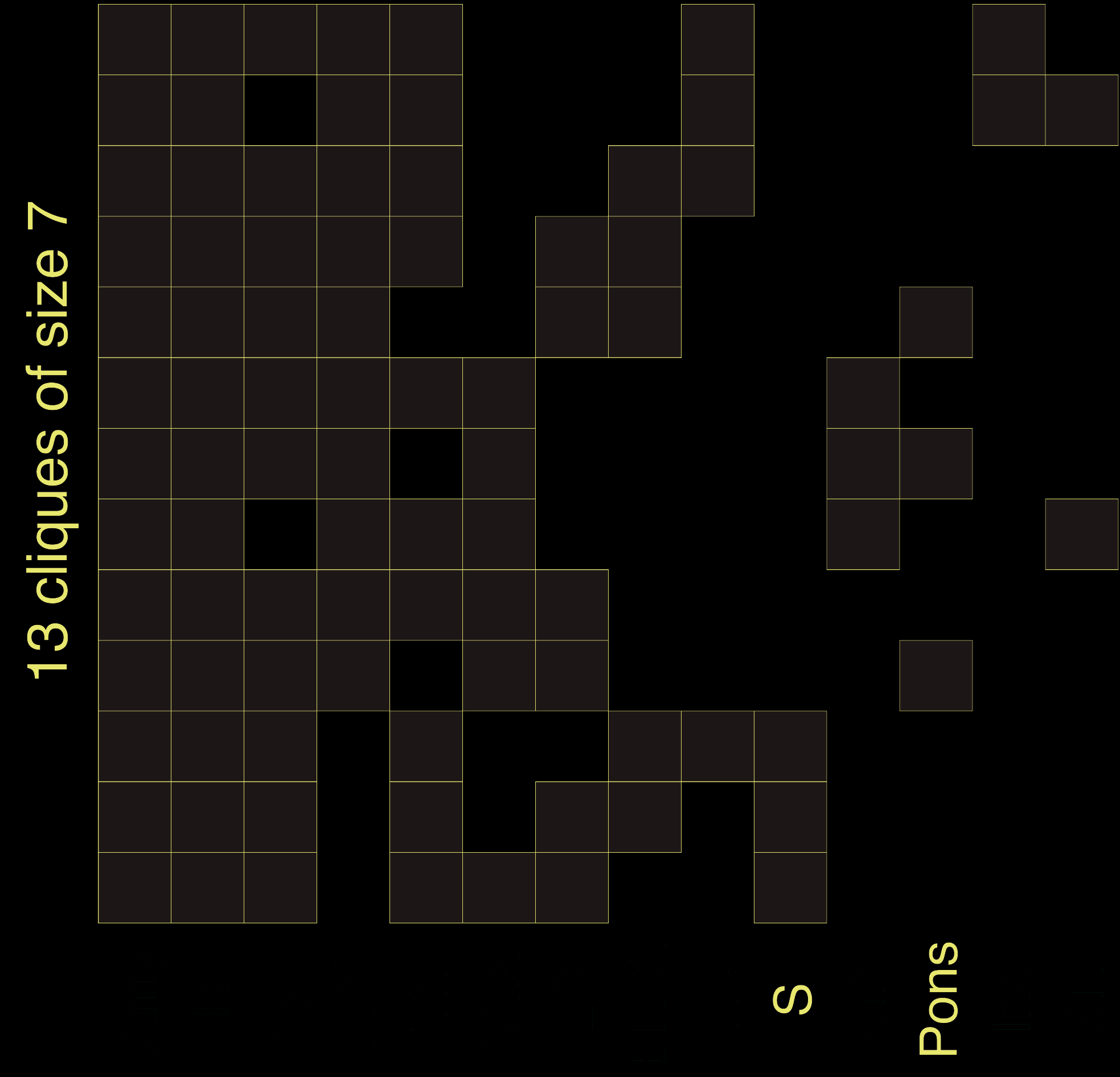

Supplement: S14 Fig — There are 13 cliques of (the largest) size 7 sitting on 14 nodes. All the 12 core areas found with the stochastic block modeling method (shown in red) are also found among these 14. (TIF) [file pbio.2002612.s014.tif]
