# Supplementary material for: A multiscale cerebral neurochemical connectome of the rat brain
Source: PLoS Biol. 2017 Jul 3;15(7):e2002612. doi: 10.1371/journal.pbio.2002612 (PMC5507471; doi:10.1371/journal.pbio.2002612)
Supplement: S1 Text — (DOCX) [file pbio.2002612.s028.docx]

**Database references:**

**Olfactory Bulb**

Intraregional connections

Main olfactory bulb (efferents and afferents)

1. Guevara-Aguilar, R., et al., Olfactory and visceral projections to the paraventricular nucleus. Brain Res Bull, 1988. 20(6): p. 799-801.

2. Smithson, K.G., M.L. Weiss, and G.I. Hatton, Supraoptic nucleus afferents from the main olfactory bulb--I. Anatomical evidence from anterograde and retrograde tracers in rat. Neuroscience, 1989. 31(2): p. 277-87.

3. Powell, T.P., W.M. Cowan, and G. Raisman, The central olfactory connexions. J Anat, 1965. 99(Pt 4): p. 791-813.

4. Kosel, K.C., G.W. Van Hoesen, and J.R. West, Olfactory bulb projections to the parahippocampal area of the rat. J Comp Neurol, 1981. 198(3): p. 467-82.

5. Meibach, R.C. and A. Siegel, Thalamic projections of the hippocampal formation: evidence for an alternate pathway involving the internal capsule. Brain Res, 1977. 134(1): p. 1-12.

6. Krettek, J.E. and J.L. Price, Projections from the amygdaloid complex and adjacent olfactory structures to the entorhinal cortex and to the subiculum in the rat and cat. J Comp Neurol, 1977. 172(4): p. 723-52.

7. Wouterlood, F.G., E. Mugnaini, and J. Nederlof, Projection of olfactory bulb efferents to layer I GABAergic neurons in the entorhinal area. Combination of anterograde degeneration and immunoelectron microscopy in rat. Brain Res, 1985. 343(2): p. 283-96.

8. Scott, J.W., R.L. McBride, and S.P. Schneider, The organization of projections from the olfactory bulb to the piriform cortex and olfactory tubercle in the rat. J Comp Neurol, 1980. 194(3): p. 519-34.

9. Raisman, G., An experimental study of the projection of the amygdala to the accessory olfactory bulb and its relationship to the concept of a dual olfactory system. Exp Brain Res, 1972. 14(4): p. 395-408.

10. Fuller, T.A. and J.L. Price, Putative glutamatergic and/or aspartatergic cells in the main and accessory olfactory bulbs of the rat. J Comp Neurol, 1988. 276(2): p. 209-18.

11. Zaborszky, L., et al., Cholinergic and GABAergic afferents to the olfactory bulb in the rat with special emphasis on the projection neurons in the nucleus of the horizontal limb of the diagonal band. J Comp Neurol, 1986. 243(4): p. 488-509.

12. Okoyama, S., et al., Cholinergic divergent projections from rat basal forebrain to the hippocampus and olfactory bulb. Neurosci Lett, 1987. 83(1-2): p. 77-81.

13. Gracia-Llanes, F.J., et al., GABAergic basal forebrain afferents innervate selectively GABAergic targets in the main olfactory bulb. Neuroscience, 2010. 170(3): p. 913-22.

14. de Olmos, J., H. Hardy, and L. Heimer, The afferent connections of the main and the accessory olfactory bulb formations in the rat: an experimental HRP-study. J Comp Neurol, 1978. 181(2): p. 213-44.

15. Gaykema, R.P., et al., Cortical projection patterns of the medial septum-diagonal band complex. J Comp Neurol, 1990. 293(1): p. 103-24.

16. Pedersen, P.E. and T.E. Benson, Projection of septal organ receptor neurons to the main olfactory bulb in rats. J Comp Neurol, 1986. 252(4): p. 555-62.

17. Swanson, L.W., An autoradiographic study of the efferent connections of the preoptic region in the rat. J Comp Neurol, 1976. 167(2): p. 227-56.

18. Kohler, C., et al., The cytoarchitecture, histochemistry and projections of the tuberomammillary nucleus in the rat. Neuroscience, 1985. 16(1): p. 85-110.

19. Araneda, S., K. Gysling, and A. Calas, Raphe serotonergic neurons projecting to the olfactory bulb contain galanin or somatostatin but not neurotensin. Brain Res Bull, 1999. 49(3): p. 209-14.

20. McLean, S., R.B. Rothman, and M. Herkenham, Autoradiographic localization of mu- and delta-opiate receptors in the forebrain of the rat. Brain Res, 1986. 378(1): p. 49-60.

21. Bobillier, P., et al., The efferent connections of the nucleus raphe centralis superior in the rat as revealed by radioautography. Brain Res, 1979. 166(1): p. 1-8.

22. Watanabe, K. and E. Kawana, Selective retrograde transport of tritiated D-aspartate from the olfactory bulb to the anterior olfactory nucleus, pyriform cortex and nucleus of the lateral olfactory tract in the rat. Brain Res, 1984. 296(1): p. 148-51.

23. van Groen, T. and J.M. Wyss, Extrinsic projections from area CA1 of the rat hippocampus: olfactory, cortical, subcortical, and bilateral hippocampal formation projections. J Comp Neurol, 1990. 302(3): p. 515-28.

24. McLean, J.H. and M.T. Shipley, Serotonergic afferents to the rat olfactory bulb: I. Origins and laminar specificity of serotonergic inputs in the adult rat. J Neurosci, 1987. 7(10): p. 3016-28.

25. McLean, J.H., et al., Chemoanatomical organization of the noradrenergic input from locus coeruleus to the olfactory bulb of the adult rat. J Comp Neurol, 1989. 285(3): p. 339-49.

26. Shipley, M.T., F.J. Halloran, and J. de la Torre, Surprisingly rich projection from locus coeruleus to the olfactory bulb in the rat. Brain Res, 1985. 329(1-2): p. 294-9.

27. Guevara-Aguilar, R., et al., Differential projections from locus coeruleus to olfactory bulb and olfactory tubercle: an HRP study. Brain Res Bull, 1982. 8(6): p. 711-9.

28. Cornwall, J. and O.T. Phillipson, Single neurones of the basal forebrain and laterodorsal tegmental nucleus project by collateral axons to the olfactory bulb and the mediodorsal nucleus in the rat. Brain Res, 1989. 491(1): p. 194-8.

29. Neafsey, E.J., K.M. Hurley-Gius, and D. Arvanitis, The topographical organization of neurons in the rat medial frontal, insular and olfactory cortex projecting to the solitary nucleus, olfactory bulb, periaqueductal gray and superior colliculus. Brain Res, 1986. 377(2): p. 261-70.

Accessory olfactory bulb (efferents and afferents)

1. Smithson, K.G., M.L. Weiss, and G.I. Hatton, Supraoptic nucleus afferents from the accessory olfactory bulb: evidence from anterograde and retrograde tract tracing in the rat. Brain Res Bull, 1992. 29(2): p. 209-20.

2. Raisman, G., An experimental study of the projection of the amygdala to the accessory olfactory bulb and its relationship to the concept of a dual olfactory system. Exp Brain Res, 1972. 14(4): p. 395-408.

3. Fuller, T.A. and J.L. Price, Putative glutamatergic and/or aspartatergic cells in the main and accessory olfactory bulbs of the rat. J Comp Neurol, 1988. 276(2): p. 209-18.

4. de Olmos, J., H. Hardy, and L. Heimer, The afferent connections of the main and the accessory olfactory bulb formations in the rat: an experimental HRP-study. J Comp Neurol, 1978. 181(2): p. 213-44.

5. Swanson, L.W., An autoradiographic study of the efferent connections of the preoptic region in the rat. J Comp Neurol, 1976. 167(2): p. 227-56.

6. Canteras, N.S., R.B. Simerly, and L.W. Swanson, Organization of projections from the medial nucleus of the amygdala: a PHAL study in the rat. J Comp Neurol, 1995. 360(2): p. 213-45.

7. de la Rosa-Prieto, C., et al., Subicular and CA1 hippocampal projections to the accessory olfactory bulb. Hippocampus, 2009. 19(2): p. 124-9.

8. McLean, J.H., et al., Chemoanatomical organization of the noradrenergic input from locus coeruleus to the olfactory bulb of the adult rat. J Comp Neurol, 1989. 285(3): p. 339-49.

9. Pedersen, P.E. and T.E. Benson, Projection of septal organ receptor neurons to the main olfactory bulb in rats. J Comp Neurol, 1986. 252(4): p. 555-62.

#### Prefrontal Cortex

Intraregional connections

1. Gaykema, R.P., et al., Prefrontal cortical projections to the cholinergic neurons in the basal forebrain. J Comp Neurol, 1991. 303(4): p. 563-83.

Anterior Cingulate Cortex (efferents and afferents)

1. Carter, C.J., Topographical distribution of possible glutamatergic pathways from the frontal cortex to the striatum and substantia nigra in rats. Neuropharmacology, 1982. 21(5): p. 379-83.

2. Montaron, M.F., et al., Prefrontal cortex inputs of the nucleus accumbens-nigro-thalamic circuit. Neuroscience, 1996. 71(2): p. 371-82.

3. Sesack, S.R., et al., Topographical organization of the efferent projections of the medial prefrontal cortex in the rat: an anterograde tract-tracing study with Phaseolus vulgaris leucoagglutinin. J Comp Neurol, 1989. 290(2): p. 213-42.

4. Wang, X., P. Zhong, and Z. Yan, Dopamine D4 receptors modulate GABAergic signaling in pyramidal neurons of prefrontal cortex. J Neurosci, 2002. 22(21): p. 9185-93.

5. Vertes, R.P., Analysis of projections from the medial prefrontal cortex to the thalamus in the rat, with emphasis on nucleus reuniens. J Comp Neurol, 2002. 442(2): p. 163-87.

6. Luiten, P.G. and P. Room, Interrelations between lateral, dorsomedial and ventromedial hypothalamic nuclei in the rat. An HRP study. Brain Res, 1980. 190(2): p. 321-32.

7. Allen, G.V. and D.A. Hopkins, Mamillary body in the rat: topography and synaptology of projections from the subicular complex, prefrontal cortex, and midbrain tegmentum. J Comp Neurol, 1989. 286(3): p. 311-36.

8. Abrahamson, E.E. and R.Y. Moore, The posterior hypothalamic area: chemoarchitecture and afferent connections. Brain Res, 2001. 889(1-2): p. 1-22.

9. McDonald, A.J., F. Mascagni, and L. Guo, Projections of the medial and lateral prefrontal cortices to the amygdala: a Phaseolus vulgaris leucoagglutinin study in the rat. Neuroscience, 1996. 71(1): p. 55-75.

10. Cassell, M.D. and D.J. Wright, Topography of projections from the medial prefrontal cortex to the amygdala in the rat. Brain Res Bull, 1986. 17(3): p. 321-33.

11. Ottersen, O.P., Connections of the amygdala of the rat. IV: Corticoamygdaloid and intraamygdaloid connections as studied with axonal transport of horseradish peroxidase. J Comp Neurol, 1982. 205(1): p. 30-48.

12. Greatrex, R.M. and O.T. Phillipson, Demonstration of synaptic input from prefrontal cortex to the habenula i the rat. Brain Res, 1982. 238(1): p. 192-7.

13. Jones, B.F. and M.P. Witter, Cingulate cortex projections to the parahippocampal region and hippocampal formation in the rat. Hippocampus, 2007. 17(10): p. 957-76.

14. Deacon, T.W., et al., Afferent connections of the perirhinal cortex in the rat. J Comp Neurol, 1983. 220(2): p. 168-90.

15. Kamishina, H., et al., Cortical connections of the rat lateral posterior thalamic nucleus. Brain Res, 2009. 1264: p. 39-56.

16. Vertes, R.P., et al., Nucleus reuniens of the midline thalamus: link between the medial prefrontal cortex and the hippocampus. Brain Res Bull, 2007. 71(6): p. 601-9.

17. Hardy, S.G., Projections to the midbrain from the medial versus lateral prefrontal cortices of the rat. Neurosci Lett, 1986. 63(2): p. 159-64.

18. Groenewegen, H.J., Organization of the afferent connections of the mediodorsal thalamic nucleus in the rat, related to the mediodorsal-prefrontal topography. Neuroscience, 1988. 24(2): p. 379-431.

19. Chen, S. and H.S. Su, Afferent connections of the thalamic paraventricular and parataenial nuclei in the rat--a retrograde tracing study with iontophoretic application of Fluoro-Gold. Brain Res, 1990. 522(1): p. 1-6.

20. Herkenham, M., The connections of the nucleus reuniens thalami: evidence for a direct thalamo-hippocampal pathway in the rat. J Comp Neurol, 1978. 177(4): p. 589-610.

21. Cornwall, J. and O.T. Phillipson, Mediodorsal and reticular thalamic nuclei receive collateral axons from prefrontal cortex and laterodorsal tegmental nucleus in the rat. Neurosci Lett, 1988. 88(2): p. 121-6.

22. Coolen, L.M., et al., Afferent connections of the parvocellular subparafascicular thalamic nucleus in the rat: evidence for functional subdivisions. J Comp Neurol, 2003. 463(2): p. 132-56.

23. Beckstead, R.M., Convergent prefrontal and nigral projections to the striatum of the rat. Neurosci Lett, 1979. 12(1): p. 59-64.

24. Geisler, S., et al., Glutamatergic afferents of the ventral tegmental area in the rat. J Neurosci, 2007. 27(21): p. 5730-43.

25. Hajos, M., et al., An electrophysiological and neuroanatomical study of the medial prefrontal cortical projection to the midbrain raphe nuclei in the rat. Neuroscience, 1998. 87(1): p. 95-108.

26. Goncalves, L., et al., Prefrontal afferents to the dorsal raphe nucleus in the rat. Brain Res Bull, 2009. 78(4-5): p. 240-7.

27. Vazquez-Borsetti, P., et al., Simultaneous projections from prefrontal cortex to dopaminergic and serotonergic nuclei. Int J Neuropsychopharmacol, 2011. 14(3): p. 289-302.

28. Saper, C.B., Reciprocal parabrachial-cortical connections in the rat. Brain Res, 1982. 242(1): p. 33-40.

29. van der Kooy, D., et al., Visceral cortex: a direct connection from prefrontal cortex to the solitary nucleus in rat. Neurosci Lett, 1982. 33(2): p. 123-7.

30. Christie, M.J., L.B. James, and P.M. Beart, An excitatory amino acid projection from rat prefrontal cortex to periaqueductal gray. Brain Res Bull, 1986. 16(1): p. 127-9.

31. McDonald, A.J., Organization of amygdaloid projections to the prefrontal cortex and associated striatum in the rat. Neuroscience, 1991. 44(1): p. 1-14.

32. Jasmin, L., et al., Rostral agranular insular cortex and pain areas of the central nervous system: a tract-tracing study in the rat. J Comp Neurol, 2004. 468(3): p. 425-40.

33. Mayo, W., et al., Cortical cholinergic projections from the basal forebrain of the rat, with special reference to the prefrontal cortex innervation. Neurosci Lett, 1984. 47(2): p. 149-54.

34. Sarter, M. and H.J. Markowitsch, Collateral innervation of the medial and lateral prefrontal cortex by amygdaloid, thalamic, and brain-stem neurons. J Comp Neurol, 1984. 224(3): p. 445-60.

35. Bacon, S.J., et al., Amygdala input to medial prefrontal cortex (mPFC) in the rat: a light and electron microscope study. Brain Res, 1996. 720(1-2): p. 211-9.

36. Gabbott, P.L., T.A. Warner, and S.J. Busby, Amygdala input monosynaptically innervates parvalbumin immunoreactive local circuit neurons in rat medial prefrontal cortex. Neuroscience, 2006. 139(3): p. 1039-48.

37. Cunningham, M.G., et al., Zincergic innervation of medial prefrontal cortex by basolateral projection neurons. Neuroreport, 2007. 18(6): p. 531-5.

38. Gabbott, P., et al., Amygdala afferents monosynaptically innervate corticospinal neurons in rat medial prefrontal cortex. J Comp Neurol, 2012. 520(11): p. 2440-58.

39. McDonald, A.J., Organization of amygdaloid projections to the mediodorsal thalamus and prefrontal cortex: a fluorescence retrograde transport study in the rat. J Comp Neurol, 1987. 262(1): p. 46-58.

40. Sripanidkulchai, K., B. Sripanidkulchai, and J.M. Wyss, The cortical projection of the basolateral amygdaloid nucleus in the rat: a retrograde fluorescent dye study. J Comp Neurol, 1984. 229(3): p. 419-31.

41. Carr, D.B. and S.R. Sesack, Hippocampal afferents to the rat prefrontal cortex: synaptic targets and relation to dopamine terminals. J Comp Neurol, 1996. 369(1): p. 1-15.

42. Conde, F., et al., Afferent connections of the medial frontal cortex of the rat. A study using retrograde transport of fluorescent dyes. I. Thalamic afferents. Brain Res Bull, 1990. 24(3): p. 341-54.

43. Vertes, R.P., W.B. Hoover, and J.J. Rodriguez, Projections of the central medial nucleus of the thalamus in the rat: node in cortical, striatal and limbic forebrain circuitry. Neuroscience, 2012. 219: p. 120-36.

44. Berendse, H.W. and H.J. Groenewegen, Restricted cortical termination fields of the midline and intralaminar thalamic nuclei in the rat. Neuroscience, 1991. 42(1): p. 73-102.

45. Andree, T.H., et al., Evidence for cholinergic muscarinic receptors on mediodorsal thalamic projections to the anterior cingulate cortex. Neurosci Lett, 1983. 40(2): p. 99-103.

46. Vertes, R.P. and W.B. Hoover, Projections of the paraventricular and paratenial nuclei of the dorsal midline thalamus in the rat. J Comp Neurol, 2008. 508(2): p. 212-37.

47. Bubser, M. and A.Y. Deutch, Thalamic paraventricular nucleus neurons collateralize to innervate the prefrontal cortex and nucleus accumbens. Brain Res, 1998. 787(2): p. 304-10.

48. Ohtake, T. and H. Yamada, Efferent connections of the nucleus reuniens and the rhomboid nucleus in the rat: an anterograde PHA-L tracing study. Neurosci Res, 1989. 6(6): p. 556-68.

49. Yoshida, M., et al., Dopaminergic neurons in the nucleus raphe dorsalis innervate the prefrontal cortex in the rat: a combined retrograde tracing and immunohistochemical study using anti-dopamine serum. Brain Res, 1989. 496(1-2): p. 373-6.

50. Cornwall, J. and O.T. Phillipson, Afferent projections to the parafascicular thalamic nucleus of the rat, as shown by the retrograde transport of wheat germ agglutinin. Brain Res Bull, 1988. 20(2): p. 139-50.

51. Loughlin, S.E. and J.H. Fallon, Substantia nigra and ventral tegmental area projections to cortex: topography and collateralization. Neuroscience, 1984. 11(2): p. 425-35.

52. Condes Lara, M., et al., Mesencephalic projections to the thalamic centralis lateralis and medial prefrontal cortex: a WGA-HRP study. Brain Res, 1990. 509(2): p. 321-4.

53. Margolis, E.B., et al., Kappa opioids selectively control dopaminergic neurons projecting to the prefrontal cortex. Proc Natl Acad Sci U S A, 2006. 103(8): p. 2938-42.

54. Gerfen, C.R. and R.M. Clavier, Neural inputs to the prefrontal agranular insular cortex in the rat: horseradish peroxidase study. Brain Res Bull, 1979. 4(3): p. 347-53.

55. Van Bockstaele, E.J., A. Biswas, and V.M. Pickel, Topography of serotonin neurons in the dorsal raphe nucleus that send axon collaterals to the rat prefrontal cortex and nucleus accumbens. Brain Res, 1993. 624(1-2): p. 188-98.

56. Del Cid-Pellitero, E. and M. Garzon, Medial prefrontal cortex receives input from dorsal raphe nucleus neurons targeted by hypocretin1/orexinA-containing axons. Neuroscience, 2011. 172: p. 30-43.

57. Soria-Fregozo, C., et al., Prefrontal serotonergic denervation induces increase in the density of 5-HT2A receptors in adult rat prefrontal cortex. Neurochem Res, 2008. 33(11): p. 2350-7.

58. Puig, M.V., et al., Serotonin modulates fast-spiking interneuron and synchronous activity in the rat prefrontal cortex through 5-HT1A and 5-HT2A receptors. J Neurosci, 2010. 30(6): p. 2211-22.

59. Vertes, R.P., A PHA-L analysis of ascending projections of the dorsal raphe nucleus in the rat. J Comp Neurol, 1991. 313(4): p. 643-68.

60. van Bockstaele, E.J., E.E. Colago, and V.M. Pickel, Enkephalin terminals form inhibitory-type synapses on neurons in the rat nucleus locus coeruleus that project to the medial prefrontal cortex. Neuroscience, 1996. 71(2): p. 429-42.

61. Van Eden, C.G., V.A. Lamme, and H.B. Uylings, Heterotopic Cortical Afferents to the Medial Prefrontal Cortex in the Rat. A Combined Retrograde and Anterograde Tracer Study. Eur J Neurosci, 1992. 4(1): p. 77-97.

62. Peinado, J.M., et al., Putative amino acid neurotransmitters and the nucleus dorsomedialis thalamus-prefrontal cortex pathway in the rat. Brain Res Bull, 1983. 10(4): p. 421-4.

63. Saper, C.B., Organization of cerebral cortical afferent systems in the rat. II. Hypothalamocortical projections. J Comp Neurol, 1985. 237(1): p. 21-46.

Infralimbic Cortex (efferents and afferents)

1. Hurley, K.M., et al., Efferent projections of the infralimbic cortex of the rat. J Comp Neurol, 1991. 308(2): p. 249-76.

2. Jasmin, L., et al., Rostral agranular insular cortex and pain areas of the central nervous system: a tract-tracing study in the rat. J Comp Neurol, 2004. 468(3): p. 425-40.

3. Shi, C.J. and M.D. Cassell, Cortical, thalamic, and amygdaloid connections of the anterior and posterior insular cortices. J Comp Neurol, 1998. 399(4): p. 440-68.

4. Vertes, R.P., Differential projections of the infralimbic and prelimbic cortex in the rat. Synapse, 2004. 51(1): p. 32-58.

5. Ding, D.C., P.L. Gabbott, and S. Totterdell, Differences in the laminar origin of projections from the medial prefrontal cortex to the nucleus accumbens shell and core regions in the rat. Brain Res, 2001. 917(1): p. 81-9.

6. Sesack, S.R. and V.M. Pickel, Prefrontal cortical efferents in the rat synapse on unlabeled neuronal targets of catecholamine terminals in the nucleus accumbens septi and on dopamine neurons in the ventral tegmental area. J Comp Neurol, 1992. 320(2): p. 145-60.

7. Carr, D.B., et al., Dopamine terminals in the rat prefrontal cortex synapse on pyramidal cells that project to the nucleus accumbens. J Neurosci, 1999. 19(24): p. 11049-60.

8. Sesack, S.R., et al., Topographical organization of the efferent projections of the medial prefrontal cortex in the rat: an anterograde tract-tracing study with Phaseolus vulgaris leucoagglutinin. J Comp Neurol, 1989. 290(2): p. 213-42.

9. Takagishi, M. and T. Chiba, Efferent projections of the infralimbic (area 25) region of the medial prefrontal cortex in the rat: an anterograde tracer PHA-L study. Brain Res, 1991. 566(1-2): p. 26-39.

10. Carter, C.J., Topographical distribution of possible glutamatergic pathways from the frontal cortex to the striatum and substantia nigra in rats. Neuropharmacology, 1982. 21(5): p. 379-83.

11. Brog, J.S., et al., The patterns of afferent innervation of the core and shell in the "accumbens" part of the rat ventral striatum: immunohistochemical detection of retrogradely transported fluoro-gold. J Comp Neurol, 1993. 338(2): p. 255-78.

12. Walaas, I. and F. Fonnum, Biochemical evidence for gamma-aminobutyrate containing fibres from the nucleus accumbens to the substantia nigra and ventral tegmental area in the rat. Neuroscience, 1980. 5(1): p. 63-72.

13. Hardy, S.G., Projections to the midbrain from the medial versus lateral prefrontal cortices of the rat. Neurosci Lett, 1986. 63(2): p. 159-64.

14. McDonald, A.J., Organization of amygdaloid projections to the mediodorsal thalamus and prefrontal cortex: a fluorescence retrograde transport study in the rat. J Comp Neurol, 1987. 262(1): p. 46-58.

15. McDonald, A.J., et al., Cortical afferents to the extended amygdala. Ann N Y Acad Sci, 1999. 877: p. 309-38.

16. Shin, J.W., J.C. Geerling, and A.D. Loewy, Inputs to the ventrolateral bed nucleus of the stria terminalis. J Comp Neurol, 2008. 511(5): p. 628-57.

17. Thompson, R.H. and L.W. Swanson, Organization of inputs to the dorsomedial nucleus of the hypothalamus: a reexamination with Fluorogold and PHAL in the rat. Brain Res Brain Res Rev, 1998. 27(2): p. 89-118.

18. Wouterlood, F.G., et al., Projection from the prefrontal cortex to histaminergic cell groups in the posterior hypothalamic region of the rat. Anterograde tracing with Phaseolus vulgaris leucoagglutinin combined with immunocytochemistry of histidine decarboxylase. Brain Res, 1987. 406(1-2): p. 330-6.

19. Allen, G.V. and D.A. Hopkins, Mamillary body in the rat: topography and synaptology of projections from the subicular complex, prefrontal cortex, and midbrain tegmentum. J Comp Neurol, 1989. 286(3): p. 311-36.

20. Gonzalo-Ruiz, A., et al., Afferent projections to the mammillary complex of the rat, with special reference to those from surrounding hypothalamic regions. J Comp Neurol, 1992. 321(2): p. 277-99.

21. Shibata, H., Descending projections to the mammillary nuclei in the rat, as studied by retrograde and anterograde transport of wheat germ agglutinin-horseradish peroxidase. J Comp Neurol, 1989. 285(4): p. 436-52.

22. Simerly, R.B. and L.W. Swanson, The organization of neural inputs to the medial preoptic nucleus of the rat. J Comp Neurol, 1986. 246(3): p. 312-42.

23. Comoli, E., E.R. Ribeiro-Barbosa, and N.S. Canteras, Afferent connections of the dorsal premammillary nucleus. J Comp Neurol, 2000. 423(1): p. 83-98.

24. Moga, M.M. and R.Y. Moore, Organization of neural inputs to the suprachiasmatic nucleus in the rat. J Comp Neurol, 1997. 389(3): p. 508-34.

25. Hayakawa, T., H. Ito, and K. Zyo, Neuroanatomical study of afferent projections to the supramammillary nucleus of the rat. Anat Embryol (Berl), 1993. 188(2): p. 139-48.

26. McDonald, A.J., F. Mascagni, and L. Guo, Projections of the medial and lateral prefrontal cortices to the amygdala: a Phaseolus vulgaris leucoagglutinin study in the rat. Neuroscience, 1996. 71(1): p. 55-75.

27. Cassell, M.D. and D.J. Wright, Topography of projections from the medial prefrontal cortex to the amygdala in the rat. Brain Res Bull, 1986. 17(3): p. 321-33.

28. Ottersen, O.P., Connections of the amygdala of the rat. IV: Corticoamygdaloid and intraamygdaloid connections as studied with axonal transport of horseradish peroxidase. J Comp Neurol, 1982. 205(1): p. 30-48.

29. Jones, B.F. and M.P. Witter, Cingulate cortex projections to the parahippocampal region and hippocampal formation in the rat. Hippocampus, 2007. 17(10): p. 957-76.

30. Vertes, R.P., Analysis of projections from the medial prefrontal cortex to the thalamus in the rat, with emphasis on nucleus reuniens. J Comp Neurol, 2002. 442(2): p. 163-87.

31. Vertes, R.P., et al., Nucleus reuniens of the midline thalamus: link between the medial prefrontal cortex and the hippocampus. Brain Res Bull, 2007. 71(6): p. 601-9.

32. Kuroda, M., et al., Synaptic relationships between axon terminals from the mediodorsal thalamic nucleus and gamma-aminobutyric acidergic cortical cells in the prelimbic cortex of the rat. J Comp Neurol, 2004. 477(2): p. 220-34.

33. Cornwall, J. and O.T. Phillipson, Mediodorsal and reticular thalamic nuclei receive collateral axons from prefrontal cortex and laterodorsal tegmental nucleus in the rat. Neurosci Lett, 1988. 88(2): p. 121-6.

34. Ray, J.P., et al., Sources of presumptive glutamatergic/aspartatergic afferents to the mediodorsal nucleus of the thalamus in the rat. J Comp Neurol, 1992. 320(4): p. 435-56.

35. Groenewegen, H.J., Organization of the afferent connections of the mediodorsal thalamic nucleus in the rat, related to the mediodorsal-prefrontal topography. Neuroscience, 1988. 24(2): p. 379-431.

36. Chen, S. and H.S. Su, Afferent connections of the thalamic paraventricular and parataenial nuclei in the rat--a retrograde tracing study with iontophoretic application of Fluoro-Gold. Brain Res, 1990. 522(1): p. 1-6.

37. Cornwall, J., J.D. Cooper, and O.T. Phillipson, Projections to the rostral reticular thalamic nucleus in the rat. Exp Brain Res, 1990. 80(1): p. 157-71.

38. Beckstead, R.M., Convergent prefrontal and nigral projections to the striatum of the rat. Neurosci Lett, 1979. 12(1): p. 59-64.

39. Geisler, S. and D.S. Zahm, Afferents of the ventral tegmental area in the rat-anatomical substratum for integrative functions. J Comp Neurol, 2005. 490(3): p. 270-94.

40. Wedzony, K., et al., Glutamatergic neurons of rat medial prefrontal cortex innervating the ventral tegmental area are positive for serotonin 5-HT1A receptor protein. J Physiol Pharmacol, 2007. 58(4): p. 611-24.

41. Carr, D.B. and S.R. Sesack, Terminals from the rat prefrontal cortex synapse on mesoaccumbens VTA neurons. Ann N Y Acad Sci, 1999. 877: p. 676-8.

42. Vazquez-Borsetti, P., et al., Simultaneous projections from prefrontal cortex to dopaminergic and serotonergic nuclei. Int J Neuropsychopharmacol, 2011. 14(3): p. 289-302.

43. Geisler, S., et al., Glutamatergic afferents of the ventral tegmental area in the rat. J Neurosci, 2007. 27(21): p. 5730-43.

44. Diaz-Mataix, L., F. Artigas, and P. Celada, Activation of pyramidal cells in rat medial prefrontal cortex projecting to ventral tegmental area by a 5-HT1A receptor agonist. Eur Neuropsychopharmacol, 2006. 16(4): p. 288-96.

45. Hajos, M., et al., An electrophysiological and neuroanatomical study of the medial prefrontal cortical projection to the midbrain raphe nuclei in the rat. Neuroscience, 1998. 87(1): p. 95-108.

46. Goncalves, L., et al., Prefrontal afferents to the dorsal raphe nucleus in the rat. Brain Res Bull, 2009. 78(4-5): p. 240-7.

47. Jankowski, M.P. and S.R. Sesack, Prefrontal cortical projections to the rat dorsal raphe nucleus: ultrastructural features and associations with serotonin and gamma-aminobutyric acid neurons. J Comp Neurol, 2004. 468(4): p. 518-29.

48. Hermann, D.M., et al., Afferent projections to the rat nuclei raphe magnus, raphe pallidus and reticularis gigantocellularis pars alpha demonstrated by iontophoretic application of choleratoxin (subunit b). J Chem Neuroanat, 1997. 13(1): p. 1-21.

49. Luppi, P.H., et al., Afferent projections to the rat locus coeruleus demonstrated by retrograde and anterograde tracing with cholera-toxin B subunit and Phaseolus vulgaris leucoagglutinin. Neuroscience, 1995. 65(1): p. 119-60.

50. Moga, M.M., C.B. Saper, and T.S. Gray, Neuropeptide organization of the hypothalamic projection to the parabrachial nucleus in the rat. J Comp Neurol, 1990. 295(4): p. 662-82.

51. Moga, M.M., et al., Organization of cortical, basal forebrain, and hypothalamic afferents to the parabrachial nucleus in the rat. J Comp Neurol, 1990. 295(4): p. 624-61.

52. Allen, G.V. and D.A. Hopkins, Convergent prefrontal cortex and mamillary body projections to the medial pontine nuclei: a light and electron microscopic study in the rat. J Comp Neurol, 1998. 398(3): p. 347-58.

53. van der Kooy, D., et al., Visceral cortex: a direct connection from prefrontal cortex to the solitary nucleus in rat. Neurosci Lett, 1982. 33(2): p. 123-7.

54. Christie, M.J., L.B. James, and P.M. Beart, An excitatory amino acid projection from rat prefrontal cortex to periaqueductal gray. Brain Res Bull, 1986. 16(1): p. 127-9.

55. Coolen, L.M., et al., Afferent connections of the parvocellular subparafascicular thalamic nucleus in the rat: evidence for functional subdivisions. J Comp Neurol, 2003. 463(2): p. 132-56.

56. Berendse, H.W., Y. Galis-de Graaf, and H.J. Groenewegen, Topographical organization and relationship with ventral striatal compartments of prefrontal corticostriatal projections in the rat. J Comp Neurol, 1992. 316(3): p. 314-47.

57. Condes Lara, M., et al., Mesencephalic projections to the thalamic centralis lateralis and medial prefrontal cortex: a WGA-HRP study. Brain Res, 1990. 509(2): p. 321-4.

58. Yasui, Y., et al., Autonomic responses and efferent pathways from the insular cortex in the rat. J Comp Neurol, 1991. 303(3): p. 355-74.

59. Ohara, P.T., et al., Dopaminergic input to GABAergic neurons in the rostral agranular insular cortex of the rat. J Neurocytol, 2003. 32(2): p. 131-41.

60. Dong, H.W. and L.W. Swanson, Projections from bed nuclei of the stria terminalis, dorsomedial nucleus: implications for cerebral hemisphere integration of neuroendocrine, autonomic, and drinking responses. J Comp Neurol, 2006. 494(1): p. 75-107.

61. Dong, H., G.D. Petrovich, and L.W. Swanson, Organization of projections from the juxtacapsular nucleus of the BST: a PHAL study in the rat. Brain Res, 2000. 859(1): p. 1-14.

62. Mayo, W., et al., Cortical cholinergic projections from the basal forebrain of the rat, with special reference to the prefrontal cortex innervation. Neurosci Lett, 1984. 47(2): p. 149-54.

63. Sarter, M. and H.J. Markowitsch, Collateral innervation of the medial and lateral prefrontal cortex by amygdaloid, thalamic, and brain-stem neurons. J Comp Neurol, 1984. 224(3): p. 445-60.

64. Canteras, N.S., R.B. Simerly, and L.W. Swanson, Projections of the ventral premammillary nucleus. J Comp Neurol, 1992. 324(2): p. 195-212.

65. Vertes, R.P., et al., Ascending projections of the posterior nucleus of the hypothalamus: PHA-L analysis in the rat. J Comp Neurol, 1995. 359(1): p. 90-116.

66. Sylvester, C.M., K.E. Krout, and A.D. Loewy, Suprachiasmatic nucleus projection to the medial prefrontal cortex: a viral transneuronal tracing study. Neuroscience, 2002. 114(4): p. 1071-80.

67. Bacon, S.J., et al., Amygdala input to medial prefrontal cortex (mPFC) in the rat: a light and electron microscope study. Brain Res, 1996. 720(1-2): p. 211-9.

68. Gabbott, P.L., T.A. Warner, and S.J. Busby, Amygdala input monosynaptically innervates parvalbumin immunoreactive local circuit neurons in rat medial prefrontal cortex. Neuroscience, 2006. 139(3): p. 1039-48.

69. Cunningham, M.G., et al., Zincergic innervation of medial prefrontal cortex by basolateral projection neurons. Neuroreport, 2007. 18(6): p. 531-5.

70. Ishikawa, A. and S. Nakamura, Convergence and interaction of hippocampal and amygdalar projections within the prefrontal cortex in the rat. J Neurosci, 2003. 23(31): p. 9987-95.

71. Krettek, J.E. and J.L. Price, Projections from the amygdaloid complex to the cerebral cortex and thalamus in the rat and cat. J Comp Neurol, 1977. 172(4): p. 687-722.

72. Petrovich, G.D., P.Y. Risold, and L.W. Swanson, Organization of projections from the basomedial nucleus of the amygdala: a PHAL study in the rat. J Comp Neurol, 1996. 374(3): p. 387-420.

73. Canteras, N.S., R.B. Simerly, and L.W. Swanson, Organization of projections from the medial nucleus of the amygdala: a PHAL study in the rat. J Comp Neurol, 1995. 360(2): p. 213-45.

74. Canteras, N.S., R.B. Simerly, and L.W. Swanson, Connections of the posterior nucleus of the amygdala. J Comp Neurol, 1992. 324(2): p. 143-79.

75. Swanson, L.W., A direct projection from Ammon's horn to prefrontal cortex in the rat. Brain Res, 1981. 217(1): p. 150-4.

76. Chiba, T., Collateral projection from the amygdalo--hippocampal transition area and CA1 to the hypothalamus and medial prefrontal cortex in the rat. Neurosci Res, 2000. 38(4): p. 373-83.

77. Gabbott, P., A. Headlam, and S. Busby, Morphological evidence that CA1 hippocampal afferents monosynaptically innervate PV-containing neurons and NADPH-diaphorase reactive cells in the medial prefrontal cortex (Areas 25/32) of the rat. Brain Res, 2002. 946(2): p. 314-22.

78. Ishikawa, A. and S. Nakamura, Ventral hippocampal neurons project axons simultaneously to the medial prefrontal cortex and amygdala in the rat. J Neurophysiol, 2006. 96(4): p. 2134-8.

79. Ferino, F., A.M. Thierry, and J. Glowinski, Anatomical and electrophysiological evidence for a direct projection from Ammon's horn to the medial prefrontal cortex in the rat. Exp Brain Res, 1987. 65(2): p. 421-6.

80. Delatour, B. and M.P. Witter, Projections from the parahippocampal region to the prefrontal cortex in the rat: evidence of multiple pathways. Eur J Neurosci, 2002. 15(8): p. 1400-7.

81. Carr, D.B. and S.R. Sesack, Hippocampal afferents to the rat prefrontal cortex: synaptic targets and relation to dopamine terminals. J Comp Neurol, 1996. 369(1): p. 1-15.

82. Van Eden, C.G., V.A. Lamme, and H.B. Uylings, Heterotopic Cortical Afferents to the Medial Prefrontal Cortex in the Rat. A Combined Retrograde and Anterograde Tracer Study. Eur J Neurosci, 1992. 4(1): p. 77-97.

83. Pirot, S., et al., Anatomical and electrophysiological evidence for an excitatory amino acid pathway from the thalamic mediodorsal nucleus to the prefrontal cortex in the rat. Eur J Neurosci, 1994. 6(7): p. 1225-34.

84. McIntyre, D.C., M.E. Kelly, and W.A. Staines, Efferent projections of the anterior perirhinal cortex in the rat. J Comp Neurol, 1996. 369(2): p. 302-18.

85. Witter, M.P., R.H. Ostendorf, and H.J. Groenewegen, Heterogeneity in the Dorsal Subiculum of the Rat. Distinct Neuronal Zones Project to Different Cortical and Subcortical Targets. Eur J Neurosci, 1990. 2(8): p. 718-725.

86. Groenewegen, H.J., et al., Organization of the projections from the subiculum to the ventral striatum in the rat. A study using anterograde transport of Phaseolus vulgaris leucoagglutinin. Neuroscience, 1987. 23(1): p. 103-20.

87. Conde, F., et al., Afferent connections of the medial frontal cortex of the rat. A study using retrograde transport of fluorescent dyes. I. Thalamic afferents. Brain Res Bull, 1990. 24(3): p. 341-54.

88. Vertes, R.P., W.B. Hoover, and J.J. Rodriguez, Projections of the central medial nucleus of the thalamus in the rat: node in cortical, striatal and limbic forebrain circuitry. Neuroscience, 2012. 219: p. 120-36.

89. Otake, K. and Y. Nakamura, Single midline thalamic neurons projecting to both the ventral striatum and the prefrontal cortex in the rat. Neuroscience, 1998. 86(2): p. 635-49.

90. Berendse, H.W. and H.J. Groenewegen, Restricted cortical termination fields of the midline and intralaminar thalamic nuclei in the rat. Neuroscience, 1991. 42(1): p. 73-102.

91. Vertes, R.P. and W.B. Hoover, Projections of the paraventricular and paratenial nuclei of the dorsal midline thalamus in the rat. J Comp Neurol, 2008. 508(2): p. 212-37.

92. Bubser, M. and A.Y. Deutch, Thalamic paraventricular nucleus neurons collateralize to innervate the prefrontal cortex and nucleus accumbens. Brain Res, 1998. 787(2): p. 304-10.

93. Barone, F.C., J.T. Cheng, and M.J. Wayner, GABA inhibition of lateral hypothalamic neurons: role of reticular thalamic afferents. Brain Res Bull, 1994. 33(6): p. 699-708.

94. Li, S. and G.J. Kirouac, Projections from the paraventricular nucleus of the thalamus to the forebrain, with special emphasis on the extended amygdala. J Comp Neurol, 2008. 506(2): p. 263-87.

95. Otake, K., Cholecystokinin and substance P immunoreactive projections to the paraventricular thalamic nucleus in the rat. Neurosci Res, 2005. 51(4): p. 383-94.

96. Ohtake, T. and H. Yamada, Efferent connections of the nucleus reuniens and the rhomboid nucleus in the rat: an anterograde PHA-L tracing study. Neurosci Res, 1989. 6(6): p. 556-68.

97. Yoshida, M., et al., Dopaminergic neurons in the nucleus raphe dorsalis innervate the prefrontal cortex in the rat: a combined retrograde tracing and immunohistochemical study using anti-dopamine serum. Brain Res, 1989. 496(1-2): p. 373-6.

98. Jaffe, E.H. and N. Hernandez, Release of [3H]dopamine from rat prefrontal cortex: modulation through presynaptic cholinergic heteroreceptors. Neurosci Lett, 1989. 105(1-2): p. 189-94.

99. Carr, D.B. and S.R. Sesack, GABA-containing neurons in the rat ventral tegmental area project to the prefrontal cortex. Synapse, 2000. 38(2): p. 114-23.

100. Margolis, E.B., et al., Kappa opioids selectively control dopaminergic neurons projecting to the prefrontal cortex. Proc Natl Acad Sci U S A, 2006. 103(8): p. 2938-42.

101. Yokofujita, J., et al., Synaptic characteristics between cortical cells in the rat prefrontal cortex and axon terminals from the ventral tegmental area that utilize different neurotransmitters. Int J Neurosci, 2008. 118(10): p. 1443-59.

102. Van Bockstaele, E.J., A. Biswas, and V.M. Pickel, Topography of serotonin neurons in the dorsal raphe nucleus that send axon collaterals to the rat prefrontal cortex and nucleus accumbens. Brain Res, 1993. 624(1-2): p. 188-98.

103. Del Cid-Pellitero, E. and M. Garzon, Medial prefrontal cortex receives input from dorsal raphe nucleus neurons targeted by hypocretin1/orexinA-containing axons. Neuroscience, 2011. 172: p. 30-43.

104. Puig, M.V., et al., Serotonin modulates fast-spiking interneuron and synchronous activity in the rat prefrontal cortex through 5-HT1A and 5-HT2A receptors. J Neurosci, 2010. 30(6): p. 2211-22.

105. Soria-Fregozo, C., et al., Prefrontal serotonergic denervation induces increase in the density of 5-HT2A receptors in adult rat prefrontal cortex. Neurochem Res, 2008. 33(11): p. 2350-7.

106. Vertes, R.P., A PHA-L analysis of ascending projections of the dorsal raphe nucleus in the rat. J Comp Neurol, 1991. 313(4): p. 643-68.

107. van Bockstaele, E.J., E.E. Colago, and V.M. Pickel, Enkephalin terminals form inhibitory-type synapses on neurons in the rat nucleus locus coeruleus that project to the medial prefrontal cortex. Neuroscience, 1996. 71(2): p. 429-42.

108. Saper, C.B. and A.D. Loewy, Efferent connections of the parabrachial nucleus in the rat. Brain Res, 1980. 197(2): p. 291-317.

109. Henny, P. and B.E. Jones, Projections from basal forebrain to prefrontal cortex comprise cholinergic, GABAergic and glutamatergic inputs to pyramidal cells or interneurons. Eur J Neurosci, 2008. 27(3): p. 654-70.

110. Peinado, J.M., et al., Putative amino acid neurotransmitters and the nucleus dorsomedialis thalamus-prefrontal cortex pathway in the rat. Brain Res Bull, 1983. 10(4): p. 421-4.

Prelimbic Cortex (efferents and afferents)

1. Sesack, S.R., et al., Topographical organization of the efferent projections of the medial prefrontal cortex in the rat: an anterograde tract-tracing study with Phaseolus vulgaris leucoagglutinin. J Comp Neurol, 1989. 290(2): p. 213-42.

2. Vertes, R.P., Differential projections of the infralimbic and prelimbic cortex in the rat. Synapse, 2004. 51(1): p. 32-58.

3. Carter, C.J., Topographical distribution of possible glutamatergic pathways from the frontal cortex to the striatum and substantia nigra in rats. Neuropharmacology, 1982. 21(5): p. 379-83.

4. Gorelova, N. and C.R. Yang, The course of neural projection from the prefrontal cortex to the nucleus accumbens in the rat. Neuroscience, 1997. 76(3): p. 689-706.

5. Sesack, S.R. and V.M. Pickel, Prefrontal cortical efferents in the rat synapse on unlabeled neuronal targets of catecholamine terminals in the nucleus accumbens septi and on dopamine neurons in the ventral tegmental area. J Comp Neurol, 1992. 320(2): p. 145-60.

6. Berendse, H.W., Y. Galis-de Graaf, and H.J. Groenewegen, Topographical organization and relationship with ventral striatal compartments of prefrontal corticostriatal projections in the rat. J Comp Neurol, 1992. 316(3): p. 314-47.

7. Carr, D.B., et al., Dopamine terminals in the rat prefrontal cortex synapse on pyramidal cells that project to the nucleus accumbens. J Neurosci, 1999. 19(24): p. 11049-60.

8. McDonald, A.J., Organization of amygdaloid projections to the prefrontal cortex and associated striatum in the rat. Neuroscience, 1991. 44(1): p. 1-14.

9. Montaron, M.F., et al., Prefrontal cortex inputs of the nucleus accumbens-nigro-thalamic circuit. Neuroscience, 1996. 71(2): p. 371-82.

10. Brog, J.S., et al., The patterns of afferent innervation of the core and shell in the "accumbens" part of the rat ventral striatum: immunohistochemical detection of retrogradely transported fluoro-gold. J Comp Neurol, 1993. 338(2): p. 255-78.

11. McDonald, A.J., Organization of amygdaloid projections to the mediodorsal thalamus and prefrontal cortex: a fluorescence retrograde transport study in the rat. J Comp Neurol, 1987. 262(1): p. 46-58.

12. Gaykema, R.P., et al., Prefrontal cortical projections to the cholinergic neurons in the basal forebrain. J Comp Neurol, 1991. 303(4): p. 563-83.

13. Donoghue, J.P. and M. Herkenham, Neostriatal projections from individual cortical fields conform to histochemically distinct striatal compartments in the rat. Brain Res, 1986. 365(2): p. 397-403.

14. McDonald, A.J., et al., Cortical afferents to the extended amygdala. Ann N Y Acad Sci, 1999. 877: p. 309-38.

15. Massi, L., et al., Cannabinoid receptors in the bed nucleus of the stria terminalis control cortical excitation of midbrain dopamine cells in vivo. J Neurosci, 2008. 28(42): p. 10496-508.

16. Thompson, R.H. and L.W. Swanson, Organization of inputs to the dorsomedial nucleus of the hypothalamus: a reexamination with Fluorogold and PHAL in the rat. Brain Res Brain Res Rev, 1998. 27(2): p. 89-118.

17. Allen, G.V. and D.A. Hopkins, Mamillary body in the rat: topography and synaptology of projections from the subicular complex, prefrontal cortex, and midbrain tegmentum. J Comp Neurol, 1989. 286(3): p. 311-36.

18. Shibata, H., Descending projections to the mammillary nuclei in the rat, as studied by retrograde and anterograde transport of wheat germ agglutinin-horseradish peroxidase. J Comp Neurol, 1989. 285(4): p. 436-52.

19. Gonzalo-Ruiz, A., et al., Afferent projections to the mammillary complex of the rat, with special reference to those from surrounding hypothalamic regions. J Comp Neurol, 1992. 321(2): p. 277-99.

20. Comoli, E., E.R. Ribeiro-Barbosa, and N.S. Canteras, Afferent connections of the dorsal premammillary nucleus. J Comp Neurol, 2000. 423(1): p. 83-98.

21. McDonald, A.J., F. Mascagni, and L. Guo, Projections of the medial and lateral prefrontal cortices to the amygdala: a Phaseolus vulgaris leucoagglutinin study in the rat. Neuroscience, 1996. 71(1): p. 55-75.

22. McDonald, A.J., F. Mascagni, and L. Guo, Projections of the medial and lateral prefrontal cortices to the amygdala: a Phaseolus vulgaris leucoagglutinin study in the rat. Neuroscience, 1996. 71(1): p. 55-75.

23. Cassell, M.D. and D.J. Wright, Topography of projections from the medial prefrontal cortex to the amygdala in the rat. Brain Res Bull, 1986. 17(3): p. 321-33.

24. Ottersen, O.P., Connections of the amygdala of the rat. IV: Corticoamygdaloid and intraamygdaloid connections as studied with axonal transport of horseradish peroxidase. J Comp Neurol, 1982. 205(1): p. 30-48.

25. Greatrex, R.M. and O.T. Phillipson, Demonstration of synaptic input from prefrontal cortex to the habenula i the rat. Brain Res, 1982. 238(1): p. 192-7.

26. Jones, B.F. and M.P. Witter, Cingulate cortex projections to the parahippocampal region and hippocampal formation in the rat. Hippocampus, 2007. 17(10): p. 957-76.

27. Deacon, T.W., et al., Afferent connections of the perirhinal cortex in the rat. J Comp Neurol, 1983. 220(2): p. 168-90.

28. Vertes, R.P., Analysis of projections from the medial prefrontal cortex to the thalamus in the rat, with emphasis on nucleus reuniens. J Comp Neurol, 2002. 442(2): p. 163-87.

29. Vertes, R.P., et al., Nucleus reuniens of the midline thalamus: link between the medial prefrontal cortex and the hippocampus. Brain Res Bull, 2007. 71(6): p. 601-9.

30. Kuroda, M., et al., Synaptic relationships between axon terminals from the mediodorsal thalamic nucleus and gamma-aminobutyric acidergic cortical cells in the prelimbic cortex of the rat. J Comp Neurol, 2004. 477(2): p. 220-34.

31. Hardy, S.G., Projections to the midbrain from the medial versus lateral prefrontal cortices of the rat. Neurosci Lett, 1986. 63(2): p. 159-64.

32. Cornwall, J. and O.T. Phillipson, Mediodorsal and reticular thalamic nuclei receive collateral axons from prefrontal cortex and laterodorsal tegmental nucleus in the rat. Neurosci Lett, 1988. 88(2): p. 121-6.

33. Groenewegen, H.J., Organization of the afferent connections of the mediodorsal thalamic nucleus in the rat, related to the mediodorsal-prefrontal topography. Neuroscience, 1988. 24(2): p. 379-431.

34. Ray, J.P., et al., Sources of presumptive glutamatergic/aspartatergic afferents to the mediodorsal nucleus of the thalamus in the rat. J Comp Neurol, 1992. 320(4): p. 435-56.

35. Kuroda, M. and J.L. Price, Synaptic organization of projections from basal forebrain structures to the mediodorsal thalamic nucleus of the rat. J Comp Neurol, 1991. 303(4): p. 513-33.

36. Beckstead, R.M., Convergent prefrontal and nigral projections to the striatum of the rat. Neurosci Lett, 1979. 12(1): p. 59-64.

37. Geisler, S. and D.S. Zahm, Afferents of the ventral tegmental area in the rat-anatomical substratum for integrative functions. J Comp Neurol, 2005. 490(3): p. 270-94.

38. Geisler, S., et al., Glutamatergic afferents of the ventral tegmental area in the rat. J Neurosci, 2007. 27(21): p. 5730-43.

39. Wedzony, K., et al., Glutamatergic neurons of rat medial prefrontal cortex innervating the ventral tegmental area are positive for serotonin 5-HT1A receptor protein. J Physiol Pharmacol, 2007. 58(4): p. 611-24.

40. Carr, D.B. and S.R. Sesack, Terminals from the rat prefrontal cortex synapse on mesoaccumbens VTA neurons. Ann N Y Acad Sci, 1999. 877: p. 676-8.

41. Vazquez-Borsetti, P., et al., Simultaneous projections from prefrontal cortex to dopaminergic and serotonergic nuclei. Int J Neuropsychopharmacol, 2011. 14(3): p. 289-302.

42. Diaz-Mataix, L., F. Artigas, and P. Celada, Activation of pyramidal cells in rat medial prefrontal cortex projecting to ventral tegmental area by a 5-HT1A receptor agonist. Eur Neuropsychopharmacol, 2006. 16(4): p. 288-96.

43. Goncalves, L., et al., Prefrontal afferents to the dorsal raphe nucleus in the rat. Brain Res Bull, 2009. 78(4-5): p. 240-7.

44. Jankowski, M.P. and S.R. Sesack, Prefrontal cortical projections to the rat dorsal raphe nucleus: ultrastructural features and associations with serotonin and gamma-aminobutyric acid neurons. J Comp Neurol, 2004. 468(4): p. 518-29.

45. Hermann, D.M., et al., Afferent projections to the rat nuclei raphe magnus, raphe pallidus and reticularis gigantocellularis pars alpha demonstrated by iontophoretic application of choleratoxin (subunit b). J Chem Neuroanat, 1997. 13(1): p. 1-21.

46. Lee, H.S., M.A. Kim, and B.D. Waterhouse, Retrograde double-labeling study of common afferent projections to the dorsal raphe and the nuclear core of the locus coeruleus in the rat. J Comp Neurol, 2005. 481(2): p. 179-93.

47. Allen, G.V. and D.A. Hopkins, Convergent prefrontal cortex and mamillary body projections to the medial pontine nuclei: a light and electron microscopic study in the rat. J Comp Neurol, 1998. 398(3): p. 347-58.

48. van der Kooy, D., et al., Visceral cortex: a direct connection from prefrontal cortex to the solitary nucleus in rat. Neurosci Lett, 1982. 33(2): p. 123-7.

49. Christie, M.J., L.B. James, and P.M. Beart, An excitatory amino acid projection from rat prefrontal cortex to periaqueductal gray. Brain Res Bull, 1986. 16(1): p. 127-9.

50. Condes Lara, M., et al., Mesencephalic projections to the thalamic centralis lateralis and medial prefrontal cortex: a WGA-HRP study. Brain Res, 1990. 509(2): p. 321-4.

51. Dong, H.W. and L.W. Swanson, Projections from bed nuclei of the stria terminalis, dorsomedial nucleus: implications for cerebral hemisphere integration of neuroendocrine, autonomic, and drinking responses. J Comp Neurol, 2006. 494(1): p. 75-107.

52. Dong, H., G.D. Petrovich, and L.W. Swanson, Organization of projections from the juxtacapsular nucleus of the BST: a PHAL study in the rat. Brain Res, 2000. 859(1): p. 1-14.

53. Dong, H.-W. and L.W. Swanson, Projections from bed nuclei of the stria terminalis, magnocellular nucleus: Implications for cerebral hemisphere regulation of micturition, defecation, and penile erection. The Journal of Comparative Neurology, 2006. 494(1): p. 108-141.

54. Mayo, W., et al., Cortical cholinergic projections from the basal forebrain of the rat, with special reference to the prefrontal cortex innervation. Neurosci Lett, 1984. 47(2): p. 149-54.

55. Vertes, R.P., et al., Ascending projections of the posterior nucleus of the hypothalamus: PHA-L analysis in the rat. J Comp Neurol, 1995. 359(1): p. 90-116.

56. Sarter, M. and H.J. Markowitsch, Collateral innervation of the medial and lateral prefrontal cortex by amygdaloid, thalamic, and brain-stem neurons. J Comp Neurol, 1984. 224(3): p. 445-60.

57. Canteras, N.S., R.B. Simerly, and L.W. Swanson, Projections of the ventral premammillary nucleus. J Comp Neurol, 1992. 324(2): p. 195-212.

58. Kohler, C., et al., The cytoarchitecture, histochemistry and projections of the tuberomammillary nucleus in the rat. Neuroscience, 1985. 16(1): p. 85-110.

59. Bacon, S.J., et al., Amygdala input to medial prefrontal cortex (mPFC) in the rat: a light and electron microscope study. Brain Res, 1996. 720(1-2): p. 211-9.

60. Gabbott, P.L., T.A. Warner, and S.J. Busby, Amygdala input monosynaptically innervates parvalbumin immunoreactive local circuit neurons in rat medial prefrontal cortex. Neuroscience, 2006. 139(3): p. 1039-48.

61. Cunningham, M.G., et al., Zincergic innervation of medial prefrontal cortex by basolateral projection neurons. Neuroreport, 2007. 18(6): p. 531-5.

62. Gabbott, P., et al., Amygdala afferents monosynaptically innervate corticospinal neurons in rat medial prefrontal cortex. J Comp Neurol, 2012. 520(11): p. 2440-58.

63. Krettek, J.E. and J.L. Price, Projections from the amygdaloid complex to the cerebral cortex and thalamus in the rat and cat. J Comp Neurol, 1977. 172(4): p. 687-722.

64. Ishikawa, A. and S. Nakamura, Convergence and interaction of hippocampal and amygdalar projections within the prefrontal cortex in the rat. J Neurosci, 2003. 23(31): p. 9987-95.

65. Petrovich, G.D., P.Y. Risold, and L.W. Swanson, Organization of projections from the basomedial nucleus of the amygdala: a PHAL study in the rat. J Comp Neurol, 1996. 374(3): p. 387-420.

66. Canteras, N.S., R.B. Simerly, and L.W. Swanson, Organization of projections from the medial nucleus of the amygdala: a PHAL study in the rat. J Comp Neurol, 1995. 360(2): p. 213-45.

67. Jay, T.M., J. Glowinski, and A.M. Thierry, Selectivity of the hippocampal projection to the prelimbic area of the prefrontal cortex in the rat. Brain Res, 1989. 505(2): p. 337-40.

68. Jay, T.M. and M.P. Witter, Distribution of hippocampal CA1 and subicular efferents in the prefrontal cortex of the rat studied by means of anterograde transport of Phaseolus vulgaris-leucoagglutinin. J Comp Neurol, 1991. 313(4): p. 574-86.

69. Ferino, F., A.M. Thierry, and J. Glowinski, Anatomical and electrophysiological evidence for a direct projection from Ammon's horn to the medial prefrontal cortex in the rat. Exp Brain Res, 1987. 65(2): p. 421-6.

70. Ishikawa, A. and S. Nakamura, Ventral hippocampal neurons project axons simultaneously to the medial prefrontal cortex and amygdala in the rat. J Neurophysiol, 2006. 96(4): p. 2134-8.

71. Gabbott, P., A. Headlam, and S. Busby, Morphological evidence that CA1 hippocampal afferents monosynaptically innervate PV-containing neurons and NADPH-diaphorase reactive cells in the medial prefrontal cortex (Areas 25/32) of the rat. Brain Res, 2002. 946(2): p. 314-22.

72. Chiba, T., Collateral projection from the amygdalo--hippocampal transition area and CA1 to the hypothalamus and medial prefrontal cortex in the rat. Neurosci Res, 2000. 38(4): p. 373-83.

73. Delatour, B. and M.P. Witter, Projections from the parahippocampal region to the prefrontal cortex in the rat: evidence of multiple pathways. Eur J Neurosci, 2002. 15(8): p. 1400-7.

74. Pirot, S., et al., Anatomical and electrophysiological evidence for an excitatory amino acid pathway from the thalamic mediodorsal nucleus to the prefrontal cortex in the rat. Eur J Neurosci, 1994. 6(7): p. 1225-34.

75. Carr, D.B. and S.R. Sesack, Hippocampal afferents to the rat prefrontal cortex: synaptic targets and relation to dopamine terminals. J Comp Neurol, 1996. 369(1): p. 1-15.

76. Van Eden, C.G., V.A. Lamme, and H.B. Uylings, Heterotopic Cortical Afferents to the Medial Prefrontal Cortex in the Rat. A Combined Retrograde and Anterograde Tracer Study. Eur J Neurosci, 1992. 4(1): p. 77-97.

77. Groenewegen, H.J., et al., Organization of the projections from the subiculum to the ventral striatum in the rat. A study using anterograde transport of Phaseolus vulgaris leucoagglutinin. Neuroscience, 1987. 23(1): p. 103-20.

78. Conde, F., et al., Afferent connections of the medial frontal cortex of the rat. A study using retrograde transport of fluorescent dyes. I. Thalamic afferents. Brain Res Bull, 1990. 24(3): p. 341-54.

79. Vertes, R.P., W.B. Hoover, and J.J. Rodriguez, Projections of the central medial nucleus of the thalamus in the rat: node in cortical, striatal and limbic forebrain circuitry. Neuroscience, 2012. 219: p. 120-36.

80. Berendse, H.W. and H.J. Groenewegen, Restricted cortical termination fields of the midline and intralaminar thalamic nuclei in the rat. Neuroscience, 1991. 42(1): p. 73-102.

81. Otake, K. and Y. Nakamura, Single midline thalamic neurons projecting to both the ventral striatum and the prefrontal cortex in the rat. Neuroscience, 1998. 86(2): p. 635-49.

82. Rotaru, D.C., G. Barrionuevo, and S.R. Sesack, Mediodorsal thalamic afferents to layer III of the rat prefrontal cortex: synaptic relationships to subclasses of interneurons. J Comp Neurol, 2005. 490(3): p. 220-38.

83. Vertes, R.P. and W.B. Hoover, Projections of the paraventricular and paratenial nuclei of the dorsal midline thalamus in the rat. J Comp Neurol, 2008. 508(2): p. 212-37.

84. Pinto, A., M. Jankowski, and S.R. Sesack, Projections from the paraventricular nucleus of the thalamus to the rat prefrontal cortex and nucleus accumbens shell: ultrastructural characteristics and spatial relationships with dopamine afferents. J Comp Neurol, 2003. 459(2): p. 142-55.

85. Bubser, M. and A.Y. Deutch, Thalamic paraventricular nucleus neurons collateralize to innervate the prefrontal cortex and nucleus accumbens. Brain Res, 1998. 787(2): p. 304-10.

86. Gerfen, C.R. and R.M. Clavier, Neural inputs to the prefrontal agranular insular cortex in the rat: horseradish peroxidase study. Brain Res Bull, 1979. 4(3): p. 347-53.

87. Barone, F.C., J.T. Cheng, and M.J. Wayner, GABA inhibition of lateral hypothalamic neurons: role of reticular thalamic afferents. Brain Res Bull, 1994. 33(6): p. 699-708.

88. Li, S. and G.J. Kirouac, Projections from the paraventricular nucleus of the thalamus to the forebrain, with special emphasis on the extended amygdala. J Comp Neurol, 2008. 506(2): p. 263-87.

89. Otake, K., Cholecystokinin and substance P immunoreactive projections to the paraventricular thalamic nucleus in the rat. Neurosci Res, 2005. 51(4): p. 383-94.

90. Yoshida, M., et al., Dopaminergic neurons in the nucleus raphe dorsalis innervate the prefrontal cortex in the rat: a combined retrograde tracing and immunohistochemical study using anti-dopamine serum. Brain Res, 1989. 496(1-2): p. 373-6.

91. Loughlin, S.E. and J.H. Fallon, Substantia nigra and ventral tegmental area projections to cortex: topography and collateralization. Neuroscience, 1984. 11(2): p. 425-35.

92. Del Cid-Pellitero, E. and M. Garzon, Medial prefrontal cortex receives input from dorsal raphe nucleus neurons targeted by hypocretin1/orexinA-containing axons. Neuroscience, 2011. 172: p. 30-43.

93. Margolis, E.B., et al., Kappa opioids selectively control dopaminergic neurons projecting to the prefrontal cortex. Proc Natl Acad Sci U S A, 2006. 103(8): p. 2938-42.

94. Carr, D.B. and S.R. Sesack, GABA-containing neurons in the rat ventral tegmental area project to the prefrontal cortex. Synapse, 2000. 38(2): p. 114-23.

95. Yokofujita, J., et al., Synaptic characteristics between cortical cells in the rat prefrontal cortex and axon terminals from the ventral tegmental area that utilize different neurotransmitters. Int J Neurosci, 2008. 118(10): p. 1443-59.

96. Van Bockstaele, E.J. and V.M. Pickel, Ultrastructure of serotonin-immunoreactive terminals in the core and shell of the rat nucleus accumbens: cellular substrates for interactions with catecholamine afferents. J Comp Neurol, 1993. 334(4): p. 603-17.

97. Van Bockstaele, E.J., A. Biswas, and V.M. Pickel, Topography of serotonin neurons in the dorsal raphe nucleus that send axon collaterals to the rat prefrontal cortex and nucleus accumbens. Brain Res, 1993. 624(1-2): p. 188-98.

98. Soria-Fregozo, C., et al., Prefrontal serotonergic denervation induces increase in the density of 5-HT2A receptors in adult rat prefrontal cortex. Neurochem Res, 2008. 33(11): p. 2350-7.

99. Puig, M.V., et al., Serotonin modulates fast-spiking interneuron and synchronous activity in the rat prefrontal cortex through 5-HT1A and 5-HT2A receptors. J Neurosci, 2010. 30(6): p. 2211-22.

100. van Bockstaele, E.J., E.E. Colago, and V.M. Pickel, Enkephalin terminals form inhibitory-type synapses on neurons in the rat nucleus locus coeruleus that project to the medial prefrontal cortex. Neuroscience, 1996. 71(2): p. 429-42.

101. Henny, P. and B.E. Jones, Projections from basal forebrain to prefrontal cortex comprise cholinergic, GABAergic and glutamatergic inputs to pyramidal cells or interneurons. Eur J Neurosci, 2008. 27(3): p. 654-70.

102. Peinado, J.M., et al., Putative amino acid neurotransmitters and the nucleus dorsomedialis thalamus-prefrontal cortex pathway in the rat. Brain Res Bull, 1983. 10(4): p. 421-4.

103. Saper, C.B., Organization of cerebral cortical afferent systems in the rat. II. Hypothalamocortical projections. J Comp Neurol, 1985. 237(1): p. 21-46.

Lateral Prefrontal Cortex (efferents and afferents)

1. Montaron, M.F., et al., Prefrontal cortex inputs of the nucleus accumbens-nigro-thalamic circuit. Neuroscience, 1996. 71(2): p. 371-82.

2. Hardy, S.G., Projections to the midbrain from the medial versus lateral prefrontal cortices of the rat. Neurosci Lett, 1986. 63(2): p. 159-64.

3. Gaykema, R.P., et al., Prefrontal cortical projections to the cholinergic neurons in the basal forebrain. J Comp Neurol, 1991. 303(4): p. 563-83.

4. McDonald, A.J., et al., Cortical afferents to the extended amygdala. Ann N Y Acad Sci, 1999. 877: p. 309-38.

5. McDonald, A.J., F. Mascagni, and L. Guo, Projections of the medial and lateral prefrontal cortices to the amygdala: a Phaseolus vulgaris leucoagglutinin study in the rat. Neuroscience, 1996. 71(1): p. 55-75.

6. Ottersen, O.P., Connections of the amygdala of the rat. IV: Corticoamygdaloid and intraamygdaloid connections as studied with axonal transport of horseradish peroxidase. J Comp Neurol, 1982. 205(1): p. 30-48.

7. Greatrex, R.M. and O.T. Phillipson, Demonstration of synaptic input from prefrontal cortex to the habenula i the rat. Brain Res, 1982. 238(1): p. 192-7.

8. Deacon, T.W., et al., Afferent connections of the perirhinal cortex in the rat. J Comp Neurol, 1983. 220(2): p. 168-90.

9. Groenewegen, H.J., Organization of the afferent connections of the mediodorsal thalamic nucleus in the rat, related to the mediodorsal-prefrontal topography. Neuroscience, 1988. 24(2): p. 379-431.

10. Ray, J.P., et al., Sources of presumptive glutamatergic/aspartatergic afferents to the mediodorsal nucleus of the thalamus in the rat. J Comp Neurol, 1992. 320(4): p. 435-56.

11. Geisler, S., et al., Glutamatergic afferents of the ventral tegmental area in the rat. J Neurosci, 2007. 27(21): p. 5730-43.

12. Goncalves, L., et al., Prefrontal afferents to the dorsal raphe nucleus in the rat. Brain Res Bull, 2009. 78(4-5): p. 240-7.

13. Vazquez-Borsetti, P., et al., Simultaneous projections from prefrontal cortex to dopaminergic and serotonergic nuclei. Int J Neuropsychopharmacol, 2011. 14(3): p. 289-302.

14. Saper, C.B., Reciprocal parabrachial-cortical connections in the rat. Brain Res, 1982. 242(1): p. 33-40.

15. Allen, G.V. and D.A. Hopkins, Convergent prefrontal cortex and mamillary body projections to the medial pontine nuclei: a light and electron microscopic study in the rat. J Comp Neurol, 1998. 398(3): p. 347-58.

16. van der Kooy, D., et al., Visceral cortex: a direct connection from prefrontal cortex to the solitary nucleus in rat. Neurosci Lett, 1982. 33(2): p. 123-7.

17. Christie, M.J., et al., Excitatory amino acid projections to the nucleus accumbens septi in the rat: a retrograde transport study utilizing D[3H]aspartate and [3H]GABA. Neuroscience, 1987. 22(2): p. 425-39.

18. McDonald, A.J., Organization of amygdaloid projections to the prefrontal cortex and associated striatum in the rat. Neuroscience, 1991. 44(1): p. 1-14.

19. Dong, H.W. and L.W. Swanson, Projections from bed nuclei of the stria terminalis, dorsomedial nucleus: implications for cerebral hemisphere integration of neuroendocrine, autonomic, and drinking responses. J Comp Neurol, 2006. 494(1): p. 75-107.

20. Gerfen, C.R. and R.M. Clavier, Neural inputs to the prefrontal agranular insular cortex in the rat: horseradish peroxidase study. Brain Res Bull, 1979. 4(3): p. 347-53.

21. Sarter, M. and H.J. Markowitsch, Collateral innervation of the medial and lateral prefrontal cortex by amygdaloid, thalamic, and brain-stem neurons. J Comp Neurol, 1984. 224(3): p. 445-60.

22. McDonald, A.J., Organization of amygdaloid projections to the mediodorsal thalamus and prefrontal cortex: a fluorescence retrograde transport study in the rat. J Comp Neurol, 1987. 262(1): p. 46-58.

23. Verwer, R.W., et al., Collateral projections from the rat hippocampal formation to the lateral and medial prefrontal cortex. Hippocampus, 1997. 7(4): p. 397-402.

24. Carr, D.B. and S.R. Sesack, Hippocampal afferents to the rat prefrontal cortex: synaptic targets and relation to dopamine terminals. J Comp Neurol, 1996. 369(1): p. 1-15.

25. Delatour, B. and M.P. Witter, Projections from the parahippocampal region to the prefrontal cortex in the rat: evidence of multiple pathways. Eur J Neurosci, 2002. 15(8): p. 1400-7.

26. Vertes, R.P., W.B. Hoover, and J.J. Rodriguez, Projections of the central medial nucleus of the thalamus in the rat: node in cortical, striatal and limbic forebrain circuitry. Neuroscience, 2012. 219: p. 120-36.

27. Berendse, H.W. and H.J. Groenewegen, Restricted cortical termination fields of the midline and intralaminar thalamic nuclei in the rat. Neuroscience, 1991. 42(1): p. 73-102.

28. Vertes, R.P. and W.B. Hoover, Projections of the paraventricular and paratenial nuclei of the dorsal midline thalamus in the rat. J Comp Neurol, 2008. 508(2): p. 212-37.

29. Beckstead, R.M., Convergent prefrontal and nigral projections to the striatum of the rat. Neurosci Lett, 1979. 12(1): p. 59-64.

30. Loughlin, S.E. and J.H. Fallon, Substantia nigra and ventral tegmental area projections to cortex: topography and collateralization. Neuroscience, 1984. 11(2): p. 425-35.

31. Puig, M.V., et al., Serotonin modulates fast-spiking interneuron and synchronous activity in the rat prefrontal cortex through 5-HT1A and 5-HT2A receptors. J Neurosci, 2010. 30(6): p. 2211-22.

**Insular Cortex**

Intraregional connections

1. Yasui, Y., et al., Autonomic responses and efferent pathways from the insular cortex in the rat. J Comp Neurol, 1991. 303(3): p. 355-74.

2. Wright, C.I. and H.J. Groenewegen, Patterns of overlap and segregation between insular cortical, intermediodorsal thalamic and basal amygdaloid afferents in the nucleus accumbens of the rat. Neuroscience, 1996. 73(2): p. 359-73.

3. Shi, C.J. and M.D. Cassell, Cascade projections from somatosensory cortex to the rat basolateral amygdala via the parietal insular cortex. J Comp Neurol, 1998. 399(4): p. 469-91.

#### Insular cortex - pars anterior (efferents and afferents)

1. Neafsey, E.J., K.M. Hurley-Gius, and D. Arvanitis, The topographical organization of neurons in the rat medial frontal, insular and olfactory cortex projecting to the solitary nucleus, olfactory bulb, periaqueductal gray and superior colliculus. Brain Res, 1986. 377(2): p. 261-70.

2. Jasmin, L., et al., Rostral agranular insular cortex and pain areas of the central nervous system: a tract-tracing study in the rat. J Comp Neurol, 2004. 468(3): p. 425-40.

3. Ohara, P.T., et al., Dopaminergic input to GABAergic neurons in the rostral agranular insular cortex of the rat. J Neurocytol, 2003. 32(2): p. 131-41.

4. Yasui, Y., et al., Autonomic responses and efferent pathways from the insular cortex in the rat. J Comp Neurol, 1991. 303(3): p. 355-74.

5. McDonald, A.J., et al., Cortical afferents to the extended amygdala. Ann N Y Acad Sci, 1999. 877: p. 309-38.

6. Ottersen, O.P., Connections of the amygdala of the rat. IV: Corticoamygdaloid and intraamygdaloid connections as studied with axonal transport of horseradish peroxidase. J Comp Neurol, 1982. 205(1): p. 30-48.

7. Shi, C.J. and M.D. Cassell, Cortical, thalamic, and amygdaloid connections of the anterior and posterior insular cortices. J Comp Neurol, 1998. 399(4): p. 440-68.

8. Kimura, A., H. Imbe, and T. Donishi, Efferent connections of an auditory area in the caudal insular cortex of the rat: anatomical nodes for cortical streams of auditory processing and cross-modal sensory interactions. Neuroscience, 2010. 166(4): p. 1140-57.

9. Sun, N., H. Yi, and M.D. Cassell, Evidence for a GABAergic interface between cortical afferents and brainstem projection neurons in the rat central extended amygdala. J Comp Neurol, 1994. 340(1): p. 43-64.

10. Kim, U. and T. Lee, Topography of descending projections from anterior insular and medial prefrontal regions to the lateral habenula of the epithalamus in the rat. Eur J Neurosci, 2012. 35(8): p. 1253-69.

11. Wang, J., et al., Afferent connections of the subparafascicular area in rat. Neuroscience, 2006. 138(1): p. 197-220.

12. Tsumori, T., et al., Insular cortical and amygdaloid fibers are in contact with posterolateral hypothalamic neurons projecting to the nucleus of the solitary tract in the rat. Brain Res, 2006. 1070(1): p. 139-44.

13. Cedarbaum, J.M. and G.K. Aghajanian, Afferent projections to the rat locus coeruleus as determined by a retrograde tracing technique. J Comp Neurol, 1978. 178(1): p. 1-16.

14. van der Kooy, D., et al., The organization of projections from the cortex, amygdala, and hypothalamus to the nucleus of the solitary tract in rat. J Comp Neurol, 1984. 224(1): p. 1-24.

15. Van Eden, C.G., V.A. Lamme, and H.B. Uylings, Heterotopic Cortical Afferents to the Medial Prefrontal Cortex in the Rat. A Combined Retrograde and Anterograde Tracer Study. Eur J Neurosci, 1992. 4(1): p. 77-97.

16. Reep, R.L., J.V. Corwin, and V. King, Neuronal connections of orbital cortex in rats: topography of cortical and thalamic afferents. Exp Brain Res, 1996. 111(2): p. 215-32.

17. Guldin, W.O. and H.J. Markowitsch, Cortical and thalamic afferent connections of the insular and adjacent cortex of the rat. J Comp Neurol, 1983. 215(2): p. 135-53.

18. Nakashima, M., et al., An anterograde and retrograde tract-tracing study on the projections from the thalamic gustatory area in the rat: distribution of neurons projecting to the insular cortex and amygdaloid complex. Neurosci Res, 2000. 36(4): p. 297-309.

19. Saper, C.B., Convergence of autonomic and limbic connections in the insular cortex of the rat. J Comp Neurol, 1982. 210(2): p. 163-73.

20. Krettek, J.E. and J.L. Price, Projections from the amygdaloid complex to the cerebral cortex and thalamus in the rat and cat. J Comp Neurol, 1977. 172(4): p. 687-722.

21. Berendse, H.W. and H.J. Groenewegen, Restricted cortical termination fields of the midline and intralaminar thalamic nuclei in the rat. Neuroscience, 1991. 42(1): p. 73-102.

22. Shimada, S., et al., Calcitonin gene-related peptide projection from the ventromedial thalamic nucleus to the insular cortex: a combined retrograde transport and immunocytochemical study. Brain Res, 1985. 344(1): p. 200-4.

23. Saper, C.B. and A.D. Loewy, Efferent connections of the parabrachial nucleus in the rat. Brain Res, 1980. 197(2): p. 291-317.

Insular Cortex - pars posterior (efferents and afferents)

1. Neafsey, E.J., K.M. Hurley-Gius, and D. Arvanitis, The topographical organization of neurons in the rat medial frontal, insular and olfactory cortex projecting to the solitary nucleus, olfactory bulb, periaqueductal gray and superior colliculus. Brain Res, 1986. 377(2): p. 261-70.

2. Ohara, P.T., et al., Dopaminergic input to GABAergic neurons in the rostral agranular insular cortex of the rat. J Neurocytol, 2003. 32(2): p. 131-41.

3. Yasui, Y., et al., Autonomic responses and efferent pathways from the insular cortex in the rat. J Comp Neurol, 1991. 303(3): p. 355-74.

4. Shi, C.J. and M.D. Cassell, Cortical, thalamic, and amygdaloid connections of the anterior and posterior insular cortices. J Comp Neurol, 1998. 399(4): p. 440-68.

5. McDonald, A.J., et al., Cortical afferents to the extended amygdala. Ann N Y Acad Sci, 1999. 877: p. 309-38.

6. Ottersen, O.P., Connections of the amygdala of the rat. IV: Corticoamygdaloid and intraamygdaloid connections as studied with axonal transport of horseradish peroxidase. J Comp Neurol, 1982. 205(1): p. 30-48.

7. Kimura, A., H. Imbe, and T. Donishi, Efferent connections of an auditory area in the caudal insular cortex of the rat: anatomical nodes for cortical streams of auditory processing and cross-modal sensory interactions. Neuroscience, 2010. 166(4): p. 1140-57.

8. Deacon, T.W., et al., Afferent connections of the perirhinal cortex in the rat. J Comp Neurol, 1983. 220(2): p. 168-90.

9. Wang, J., et al., Afferent connections of the subparafascicular area in rat. Neuroscience, 2006. 138(1): p. 197-220.

10. Tsumori, T., et al., Insular cortical and amygdaloid fibers are in contact with posterolateral hypothalamic neurons projecting to the nucleus of the solitary tract in the rat. Brain Res, 2006. 1070(1): p. 139-44.

11. Cedarbaum, J.M. and G.K. Aghajanian, Afferent projections to the rat locus coeruleus as determined by a retrograde tracing technique. J Comp Neurol, 1978. 178(1): p. 1-16.

12. Van Eden, C.G., V.A. Lamme, and H.B. Uylings, Heterotopic Cortical Afferents to the Medial Prefrontal Cortex in the Rat. A Combined Retrograde and Anterograde Tracer Study. Eur J Neurosci, 1992. 4(1): p. 77-97.

13. Torrealba, F. and C. Muller, Glutamate immunoreactivity of insular cortex afferents to the nucleus tractus solitarius in the rat: a quantitative electron microscopic study. Neuroscience, 1996. 71(1): p. 77-87.

14. Reep, R.L., J.V. Corwin, and V. King, Neuronal connections of orbital cortex in rats: topography of cortical and thalamic afferents. Exp Brain Res, 1996. 111(2): p. 215-32.

15. Vertes, R.P., Differential projections of the infralimbic and prelimbic cortex in the rat. Synapse, 2004. 51(1): p. 32-58.

16. Guldin, W.O. and H.J. Markowitsch, Cortical and thalamic afferent connections of the insular and adjacent cortex of the rat. J Comp Neurol, 1983. 215(2): p. 135-53.

17. Nakashima, M., et al., An anterograde and retrograde tract-tracing study on the projections from the thalamic gustatory area in the rat: distribution of neurons projecting to the insular cortex and amygdaloid complex. Neurosci Res, 2000. 36(4): p. 297-309.

18. Saper, C.B., Convergence of autonomic and limbic connections in the insular cortex of the rat. J Comp Neurol, 1982. 210(2): p. 163-73.

19. Vertes, R.P., PHA-L analysis of projections from the supramammillary nucleus in the rat. J Comp Neurol, 1992. 326(4): p. 595-622.

20. Krettek, J.E. and J.L. Price, Projections from the amygdaloid complex to the cerebral cortex and thalamus in the rat and cat. J Comp Neurol, 1977. 172(4): p. 687-722.

21. Jasmin, L., et al., Rostral agranular insular cortex and pain areas of the central nervous system: a tract-tracing study in the rat. J Comp Neurol, 2004. 468(3): p. 425-40.

22. Berendse, H.W. and H.J. Groenewegen, Restricted cortical termination fields of the midline and intralaminar thalamic nuclei in the rat. Neuroscience, 1991. 42(1): p. 73-102.

23. Shimada, S., et al., Calcitonin gene-related peptide projection from the ventromedial thalamic nucleus to the insular cortex: a combined retrograde transport and immunocytochemical study. Brain Res, 1985. 344(1): p. 200-4.

24. Shi, C.J. and M.D. Cassell, Cascade projections from somatosensory cortex to the rat basolateral amygdala via the parietal insular cortex. J Comp Neurol, 1998. 399(4): p. 469-91.

**Nucleus Accumbens**

Intraregional connections:

1. van Dongen, Y.C., et al., Anatomical evidence for direct connections between the shell and core subregions of the rat nucleus accumbens. Neuroscience, 2005. 136(4): p. 1049-71.

2. Taverna, S., et al., Direct physiological evidence for synaptic connectivity between medium-sized spiny neurons in rat nucleus accumbens in situ. J Neurophysiol, 2004. 91(3): p. 1111-21.

Nucleus Accumbens Core (efferents and afferents)

1. Usuda, I., K. Tanaka, and T. Chiba, Efferent projections of the nucleus accumbens in the rat with special reference to subdivision of the nucleus: biotinylated dextran amine study. Brain Res, 1998. 797(1): p. 73-93.

2. Conrad, L.C. and D.W. Pfaff, Autoradiographic tracing of nucleus accumbens efferents in the rat. Brain Res, 1976. 113(3): p. 589-96.

3. Heimer, L., et al., Specificity in the projection patterns of accumbal core and shell in the rat. Neuroscience, 1991. 41(1): p. 89-125.

4. Jones, D.L. and G.J. Mogenson, Nucleus accumbens to globus pallidus GABA projection subserving ambulatory activity. Am J Physiol, 1980. 238(1): p. R65-9.

5. Mogenson, G.J., L.W. Swanson, and M. Wu, Neural projections from nucleus accumbens to globus pallidus, substantia innominata, and lateral preoptic-lateral hypothalamic area: an anatomical and electrophysiological investigation in the rat. J Neurosci, 1983. 3(1): p. 189-202.

6. Dray, A. and N.R. Oakley, Projections from nucleus accumbens to globus pallidus and substantia nigra in the rat. Experientia, 1978. 34(1): p. 68-70.

7. Williams, D.J., A.R. Crossman, and P. Slater, The efferent projections of the nucleus accumbens in the rat. Brain Res, 1977. 130(2): p. 217-27.

8. Zahm, D.S. and L. Heimer, Specificity in the efferent projections of the nucleus accumbens in the rat: comparison of the rostral pole projection patterns with those of the core and shell. J Comp Neurol, 1993. 327(2): p. 220-32.

9. Zhou, L., T. Furuta, and T. Kaneko, Chemical organization of projection neurons in the rat accumbens nucleus and olfactory tubercle. Neuroscience, 2003. 120(3): p. 783-798.

10. Lu, X.Y., M.B. Ghasemzadeh, and P.W. Kalivas, Expression of D1 receptor, D2 receptor, substance P and enkephalin messenger RNAs in the neurons projecting from the nucleus accumbens. Neuroscience, 1998. 82(3): p. 767-80.

11. Lu, X.Y., M.B. Ghasemzadeh, and P.W. Kalivas, Expression of glutamate receptor subunit/subtype messenger RNAS for NMDAR1, GLuR1, GLuR2 and mGLuR5 by accumbal projection neurons. Brain Res Mol Brain Res, 1999. 63(2): p. 287-96.

12. Kuo, H. and H.T. Chang, Ventral pallido-striatal pathway in the rat brain: a light and electron microscopic study. J Comp Neurol, 1992. 321(4): p. 626-36.

13. Churchill, L. and P.W. Kalivas, A topographically organized gamma-aminobutyric acid projection from the ventral pallidum to the nucleus accumbens in the rat. J Comp Neurol, 1994. 345(4): p. 579-95.

14. Kim, E.M., et al., A bi-directional mu-opioid-opioid connection between the nucleus of the accumbens shell and the central nucleus of the amygdala in the rat. Brain Res, 2004. 1029(1): p. 135-9.

15. Groenewegen, H.J., Y. Galis-de Graaf, and W.J. Smeets, Integration and segregation of limbic cortico-striatal loops at the thalamic level: an experimental tracing study in rats. J Chem Neuroanat, 1999. 16(3): p. 167-85.

16. Carlsen, J. and L. Heimer, The projection from the parataenial thalamic nucleus, as demonstrated by the Phaseolus vulgaris-leucoagglutinin (PHA-L) method, identifies a subterritorial organization of the ventral striatum. Brain Res, 1986. 374(2): p. 375-9.

17. Otake, K. and Y. Nakamura, Single midline thalamic neurons projecting to both the ventral striatum and the prefrontal cortex in the rat. Neuroscience, 1998. 86(2): p. 635-49.

18. Kalivas, P.W., L. Churchill, and M.A. Klitenick, GABA and enkephalin projection from the nucleus accumbens and ventral pallidum to the ventral tegmental area. Neuroscience, 1993. 57(4): p. 1047-60.

19. Mansour, A., et al., A comparison of D1 receptor binding and mRNA in rat brain using receptor autoradiographic and in situ hybridization techniques. Neuroscience, 1991. 45(2): p. 359-71.

20. Walaas, I. and F. Fonnum, Biochemical evidence for gamma-aminobutyrate containing fibres from the nucleus accumbens to the substantia nigra and ventral tegmental area in the rat. Neuroscience, 1980. 5(1): p. 63-72.

21. Geisler, S. and D.S. Zahm, Afferents of the ventral tegmental area in the rat-anatomical substratum for integrative functions. J Comp Neurol, 2005. 490(3): p. 270-94.

22. Van Bockstaele, E.J. and V.M. Pickel, GABA-containing neurons in the ventral tegmental area project to the nucleus accumbens in rat brain. Brain Res, 1995. 682(1-2): p. 215-21.

23. Xia, Y., et al., Nucleus accumbens medium spiny neurons target non-dopaminergic neurons in the ventral tegmental area. J Neurosci, 2011. 31(21): p. 7811-6.

24. Carter, C.J., Topographical distribution of possible glutamatergic pathways from the frontal cortex to the striatum and substantia nigra in rats. Neuropharmacology, 1982. 21(5): p. 379-83.

25. Montaron, M.F., et al., Prefrontal cortex inputs of the nucleus accumbens-nigro-thalamic circuit. Neuroscience, 1996. 71(2): p. 371-82.

26. Hurley, K.M., et al., Efferent projections of the infralimbic cortex of the rat. J Comp Neurol, 1991. 308(2): p. 249-76.

27. Ding, D.C., P.L. Gabbott, and S. Totterdell, Differences in the laminar origin of projections from the medial prefrontal cortex to the nucleus accumbens shell and core regions in the rat. Brain Res, 2001. 917(1): p. 81-9.

28. Sesack, S.R. and V.M. Pickel, Prefrontal cortical efferents in the rat synapse on unlabeled neuronal targets of catecholamine terminals in the nucleus accumbens septi and on dopamine neurons in the ventral tegmental area. J Comp Neurol, 1992. 320(2): p. 145-60.

29. Carr, D.B., et al., Dopamine terminals in the rat prefrontal cortex synapse on pyramidal cells that project to the nucleus accumbens. J Neurosci, 1999. 19(24): p. 11049-60.

30. Sesack, S.R., et al., Topographical organization of the efferent projections of the medial prefrontal cortex in the rat: an anterograde tract-tracing study with Phaseolus vulgaris leucoagglutinin. J Comp Neurol, 1989. 290(2): p. 213-42.

31. Brog, J.S., et al., The patterns of afferent innervation of the core and shell in the "accumbens" part of the rat ventral striatum: immunohistochemical detection of retrogradely transported fluoro-gold. J Comp Neurol, 1993. 338(2): p. 255-78.

32. Gorelova, N. and C.R. Yang, The course of neural projection from the prefrontal cortex to the nucleus accumbens in the rat. Neuroscience, 1997. 76(3): p. 689-706.

33. Berendse, H.W., Y. Galis-de Graaf, and H.J. Groenewegen, Topographical organization and relationship with ventral striatal compartments of prefrontal corticostriatal projections in the rat. J Comp Neurol, 1992. 316(3): p. 314-47.

34. McDonald, A.J., Organization of amygdaloid projections to the prefrontal cortex and associated striatum in the rat. Neuroscience, 1991. 44(1): p. 1-14.

35. Jasmin, L., et al., Rostral agranular insular cortex and pain areas of the central nervous system: a tract-tracing study in the rat. J Comp Neurol, 2004. 468(3): p. 425-40.

36. Churchill, L., et al., GABAA receptors containing alpha 1 and beta 2 subunits are mainly localized on neurons in the ventral pallidum. Synapse, 1991. 8(2): p. 75-85.

37. Aylward, R.L. and S. Totterdell, Neurons in the ventral subiculum, amygdala and entorhinal cortex which project to the nucleus accumbens: their input from somatostatin-immunoreactive boutons. J Chem Neuroanat, 1993. 6(1): p. 31-42.

38. Johnson, L.R., et al., Input from the amygdala to the rat nucleus accumbens: its relationship with tyrosine hydroxylase immunoreactivity and identified neurons. Neuroscience, 1994. 61(4): p. 851-65.

39. French, S.J. and S. Totterdell, Individual nucleus accumbens-projection neurons receive both basolateral amygdala and ventral subicular afferents in rats. Neuroscience, 2003. 119(1): p. 19-31.

40. Christie, M.J., et al., Excitatory amino acid projections to the nucleus accumbens septi in the rat: a retrograde transport study utilizing D[3H]aspartate and [3H]GABA. Neuroscience, 1987. 22(2): p. 425-39.

41. McDonald, A.J., Topographical organization of amygdaloid projections to the caudatoputamen, nucleus accumbens, and related striatal-like areas of the rat brain. Neuroscience, 1991. 44(1): p. 15-33.

42. Ambroggi, F., et al., Basolateral amygdala neurons facilitate reward-seeking behavior by exciting nucleus accumbens neurons. Neuron, 2008. 59(4): p. 648-61.

43. Krettek, J.E. and J.L. Price, Amygdaloid projections to subcortical structures within the basal forebrain and brainstem in the rat and cat. J Comp Neurol, 1978. 178(2): p. 225-54.

44. Meredith, G.E., F.G. Wouterlood, and A. Pattiselanno, Hippocampal fibers make synaptic contacts with glutamate decarboxylase-immunoreactive neurons in the rat nucleus accumbens. Brain Res, 1990. 513(2): p. 329-34.

45. McIntyre, D.C., M.E. Kelly, and W.A. Staines, Efferent projections of the anterior perirhinal cortex in the rat. J Comp Neurol, 1996. 369(2): p. 302-18.

46. Vertes, R.P., W.B. Hoover, and J.J. Rodriguez, Projections of the central medial nucleus of the thalamus in the rat: node in cortical, striatal and limbic forebrain circuitry. Neuroscience, 2012. 219: p. 120-36.

47. Berendse, H.W. and H.J. Groenewegen, Organization of the thalamostriatal projections in the rat, with special emphasis on the ventral striatum. J Comp Neurol, 1990. 299(2): p. 187-228.

48. Wright, C.I. and H.J. Groenewegen, Patterns of overlap and segregation between insular cortical, intermediodorsal thalamic and basal amygdaloid afferents in the nucleus accumbens of the rat. Neuroscience, 1996. 73(2): p. 359-73.

49. Vertes, R.P. and W.B. Hoover, Projections of the paraventricular and paratenial nuclei of the dorsal midline thalamus in the rat. J Comp Neurol, 2008. 508(2): p. 212-37.

50. Barone, F.C., J.T. Cheng, and M.J. Wayner, GABA inhibition of lateral hypothalamic neurons: role of reticular thalamic afferents. Brain Res Bull, 1994. 33(6): p. 699-708.

51. Bubser, M. and A.Y. Deutch, Thalamic paraventricular nucleus neurons collateralize to innervate the prefrontal cortex and nucleus accumbens. Brain Res, 1998. 787(2): p. 304-10.

52. Li, S. and G.J. Kirouac, Projections from the paraventricular nucleus of the thalamus to the forebrain, with special emphasis on the extended amygdala. J Comp Neurol, 2008. 506(2): p. 263-87.

53. Otake, K., Cholecystokinin and substance P immunoreactive projections to the paraventricular thalamic nucleus in the rat. Neurosci Res, 2005. 51(4): p. 383-94.

54. Parsons, M.P., S. Li, and G.J. Kirouac, Functional and anatomical connection between the paraventricular nucleus of the thalamus and dopamine fibers of the nucleus accumbens. J Comp Neurol, 2007. 500(6): p. 1050-63.

55. Su, H.S. and M. Bentivoglio, Thalamic midline cell populations projecting to the nucleus accumbens, amygdala, and hippocampus in the rat. J Comp Neurol, 1990. 297(4): p. 582-93.

56. Kelley, A.E. and L. Stinus, The distribution of the projection from the parataenial nucleus of the thalamus to the nucleus accumbens in the rat: an autoradiographic study. Exp Brain Res, 1984. 54(3): p. 499-512.

57. Ohtake, T. and H. Yamada, Efferent connections of the nucleus reuniens and the rhomboid nucleus in the rat: an anterograde PHA-L tracing study. Neurosci Res, 1989. 6(6): p. 556-68.

58. Herkenham, M., The connections of the nucleus reuniens thalami: evidence for a direct thalamo-hippocampal pathway in the rat. J Comp Neurol, 1978. 177(4): p. 589-610.

59. Oleshko, N.N., et al., [Divergence of axon collaterals of substantia nigra neurons in the forebrain of the rat: double labeling with fluorochromes and horseradish peroxidase]. Neirofiziologiia, 1983. 15(5): p. 517-26.

60. Bubar, M.J., S.J. Stutz, and K.A. Cunningham, 5-HT(2C) receptors localize to dopamine and GABA neurons in the rat mesoaccumbens pathway. PLoS One, 2011. 6(6): p. e20508.

61. Margolis, E.B., et al., Kappa opioids selectively control dopaminergic neurons projecting to the prefrontal cortex. Proc Natl Acad Sci U S A, 2006. 103(8): p. 2938-42.

62. Dautan, D., et al., A major external source of cholinergic innervation of the striatum and nucleus accumbens originates in the brainstem. J Neurosci, 2014. 34(13): p. 4509-18.

63. Wang, Z.J., Z.R. Rao, and J.W. Shi, Tyrosine hydroxylase-, neurotensin-, or cholecystokinin-containing neurons in the nucleus tractus solitarii send projection fibers to the nucleus accumbens in the rat. Brain Res, 1992. 578(1-2): p. 347-50.

Nucleus Accumbens Shell (efferents and afferents)

1. Conrad, L.C. and D.W. Pfaff, Autoradiographic tracing of nucleus accumbens efferents in the rat. Brain Res, 1976. 113(3): p. 589-96.

2. Heimer, L., et al., Specificity in the projection patterns of accumbal core and shell in the rat. Neuroscience, 1991. 41(1): p. 89-125.

3. Jones, D.L. and G.J. Mogenson, Nucleus accumbens to globus pallidus GABA projection subserving ambulatory activity. Am J Physiol, 1980. 238(1): p. R65-9.

4. Mogenson, G.J., L.W. Swanson, and M. Wu, Neural projections from nucleus accumbens to globus pallidus, substantia innominata, and lateral preoptic-lateral hypothalamic area: an anatomical and electrophysiological investigation in the rat. J Neurosci, 1983. 3(1): p. 189-202.

5. Dray, A. and N.R. Oakley, Projections from nucleus accumbens to globus pallidus and substantia nigra in the rat. Experientia, 1978. 34(1): p. 68-70.

6. Williams, D.J., A.R. Crossman, and P. Slater, The efferent projections of the nucleus accumbens in the rat. Brain Res, 1977. 130(2): p. 217-27.

7. Usuda, I., K. Tanaka, and T. Chiba, Efferent projections of the nucleus accumbens in the rat with special reference to subdivision of the nucleus: biotinylated dextran amine study. Brain Res, 1998. 797(1): p. 73-93.

8. Zhou, L., T. Furuta, and T. Kaneko, Chemical organization of projection neurons in the rat accumbens nucleus and olfactory tubercle. Neuroscience, 2003. 120(3): p. 783-798.

9. Zahm, D.S. and L. Heimer, Specificity in the efferent projections of the nucleus accumbens in the rat: comparison of the rostral pole projection patterns with those of the core and shell. J Comp Neurol, 1993. 327(2): p. 220-32.

10. Lu, X.Y., M.B. Ghasemzadeh, and P.W. Kalivas, Expression of D1 receptor, D2 receptor, substance P and enkephalin messenger RNAs in the neurons projecting from the nucleus accumbens. Neuroscience, 1998. 82(3): p. 767-80.

11. Lu, X.Y., M.B. Ghasemzadeh, and P.W. Kalivas, Expression of glutamate receptor subunit/subtype messenger RNAS for NMDAR1, GLuR1, GLuR2 and mGLuR5 by accumbal projection neurons. Brain Res Mol Brain Res, 1999. 63(2): p. 287-96.

12. Churchill, L. and P.W. Kalivas, A topographically organized gamma-aminobutyric acid projection from the ventral pallidum to the nucleus accumbens in the rat. J Comp Neurol, 1994. 345(4): p. 579-95.

13. Kuo, H. and H.T. Chang, Ventral pallido-striatal pathway in the rat brain: a light and electron microscopic study. J Comp Neurol, 1992. 321(4): p. 626-36.

14. Groenewegen, H.J., Y. Galis-de Graaf, and W.J. Smeets, Integration and segregation of limbic cortico-striatal loops at the thalamic level: an experimental tracing study in rats. J Chem Neuroanat, 1999. 16(3): p. 167-85.

15. Groenewegen, H.J., Organization of the afferent connections of the mediodorsal thalamic nucleus in the rat, related to the mediodorsal-prefrontal topography. Neuroscience, 1988. 24(2): p. 379-431.

16. Coolen, L.M., et al., Afferent connections of the parvocellular subparafascicular thalamic nucleus in the rat: evidence for functional subdivisions. J Comp Neurol, 2003. 463(2): p. 132-56.

17. Walaas, I. and F. Fonnum, Biochemical evidence for gamma-aminobutyrate containing fibres from the nucleus accumbens to the substantia nigra and ventral tegmental area in the rat. Neuroscience, 1980. 5(1): p. 63-72.

18. Xia, Y., et al., Nucleus accumbens medium spiny neurons target non-dopaminergic neurons in the ventral tegmental area. J Neurosci, 2011. 31(21): p. 7811-6.

19. Van Bockstaele, E.J. and V.M. Pickel, GABA-containing neurons in the ventral tegmental area project to the nucleus accumbens in rat brain. Brain Res, 1995. 682(1-2): p. 215-21.

20. Geisler, S. and D.S. Zahm, Afferents of the ventral tegmental area in the rat-anatomical substratum for integrative functions. J Comp Neurol, 2005. 490(3): p. 270-94..

21. Geisler, S. and D.S. Zahm, Afferents of the ventral tegmental area in the rat-anatomical substratum for integrative functions. J Comp Neurol, 2005. 490(3): p. 270-94.

22. Kalivas, P.W., L. Churchill, and M.A. Klitenick, GABA and enkephalin projection from the nucleus accumbens and ventral pallidum to the ventral tegmental area. Neuroscience, 1993. 57(4): p. 1047-60.

23. Carter, C.J., Topographical distribution of possible glutamatergic pathways from the frontal cortex to the striatum and substantia nigra in rats. Neuropharmacology, 1982. 21(5): p. 379-83.

24. Brog, J.S., et al., The patterns of afferent innervation of the core and shell in the "accumbens" part of the rat ventral striatum: immunohistochemical detection of retrogradely transported fluoro-gold. J Comp Neurol, 1993. 338(2): p. 255-78.

25. Hurley, K.M., et al., Efferent projections of the infralimbic cortex of the rat. J Comp Neurol, 1991. 308(2): p. 249-76.

26. Ding, D.C., P.L. Gabbott, and S. Totterdell, Differences in the laminar origin of projections from the medial prefrontal cortex to the nucleus accumbens shell and core regions in the rat. Brain Res, 2001. 917(1): p. 81-9.

27. Sesack, S.R. and V.M. Pickel, Prefrontal cortical efferents in the rat synapse on unlabeled neuronal targets of catecholamine terminals in the nucleus accumbens septi and on dopamine neurons in the ventral tegmental area. J Comp Neurol, 1992. 320(2): p. 145-60.

28. Sesack, S.R., et al., Topographical organization of the efferent projections of the medial prefrontal cortex in the rat: an anterograde tract-tracing study with Phaseolus vulgaris leucoagglutinin. J Comp Neurol, 1989. 290(2): p. 213-42.

29. Takagishi, M. and T. Chiba, Efferent projections of the infralimbic (area 25) region of the medial prefrontal cortex in the rat: an anterograde tracer PHA-L study. Brain Res, 1991. 566(1-2): p. 26-39.

30. Gorelova, N. and C.R. Yang, The course of neural projection from the prefrontal cortex to the nucleus accumbens in the rat. Neuroscience, 1997. 76(3): p. 689-706.

31. Berendse, H.W., Y. Galis-de Graaf, and H.J. Groenewegen, Topographical organization and relationship with ventral striatal compartments of prefrontal corticostriatal projections in the rat. J Comp Neurol, 1992. 316(3): p. 314-47.

32. McDonald, A.J., Organization of amygdaloid projections to the prefrontal cortex and associated striatum in the rat. Neuroscience, 1991. 44(1): p. 1-14.

33. Jasmin, L., et al., Rostral agranular insular cortex and pain areas of the central nervous system: a tract-tracing study in the rat. J Comp Neurol, 2004. 468(3): p. 425-40.

34. Dong, H.W. and L.W. Swanson, Projections from bed nuclei of the stria terminalis, dorsomedial nucleus: implications for cerebral hemisphere integration of neuroendocrine, autonomic, and drinking responses. J Comp Neurol, 2006. 494(1): p. 75-107.

35. Dong, H.-W. and L.W. Swanson, Projections from bed nuclei of the stria terminalis, magnocellular nucleus: Implications for cerebral hemisphere regulation of micturition, defecation, and penile erection. The Journal of Comparative Neurology, 2006. 494(1): p. 108-141.

36. Dong, H.W. and L.W. Swanson, Projections from bed nuclei of the stria terminalis, anteromedial area: cerebral hemisphere integration of neuroendocrine, autonomic, and behavioral aspects of energy balance. J Comp Neurol, 2006. 494(1): p. 142-78.

37. Shin, J.W., J.C. Geerling, and A.D. Loewy, Inputs to the ventrolateral bed nucleus of the stria terminalis. J Comp Neurol, 2008. 511(5): p. 628-57.

38. Aylward, R.L. and S. Totterdell, Neurons in the ventral subiculum, amygdala and entorhinal cortex which project to the nucleus accumbens: their input from somatostatin-immunoreactive boutons. J Chem Neuroanat, 1993. 6(1): p. 31-42.

39. Johnson, L.R., et al., Input from the amygdala to the rat nucleus accumbens: its relationship with tyrosine hydroxylase immunoreactivity and identified neurons. Neuroscience, 1994. 61(4): p. 851-65.

40. French, S.J. and S. Totterdell, Individual nucleus accumbens-projection neurons receive both basolateral amygdala and ventral subicular afferents in rats. Neuroscience, 2003. 119(1): p. 19-31.

41. Meredith, G.E., F.G. Wouterlood, and A. Pattiselanno, Hippocampal fibers make synaptic contacts with glutamate decarboxylase-immunoreactive neurons in the rat nucleus accumbens. Brain Res, 1990. 513(2): p. 329-34.

42. Krayniak, P.F., R.C. Meibach, and A. Siegel, A projection from the entorhinal cortex to the nucleus accumbens in the rat. Brain Res, 1981. 209(2): p. 427-31.

43. Otake, K. and Y. Nakamura, Single midline thalamic neurons projecting to both the ventral striatum and the prefrontal cortex in the rat. Neuroscience, 1998. 86(2): p. 635-49.

44. Vertes, R.P., W.B. Hoover, and J.J. Rodriguez, Projections of the central medial nucleus of the thalamus in the rat: node in cortical, striatal and limbic forebrain circuitry. Neuroscience, 2012. 219: p. 120-36.

45. Wright, C.I. and H.J. Groenewegen, Patterns of overlap and segregation between insular cortical, intermediodorsal thalamic and basal amygdaloid afferents in the nucleus accumbens of the rat. Neuroscience, 1996. 73(2): p. 359-73.

46. Berendse, H.W. and H.J. Groenewegen, Organization of the thalamostriatal projections in the rat, with special emphasis on the ventral striatum. J Comp Neurol, 1990. 299(2): p. 187-228.

47. Carlsen, J. and L. Heimer, The projection from the parataenial thalamic nucleus, as demonstrated by the Phaseolus vulgaris-leucoagglutinin (PHA-L) method, identifies a subterritorial organization of the ventral striatum. Brain Res, 1986. 374(2): p. 375-9.

48. Kelley, A.E. and L. Stinus, The distribution of the projection from the parataenial nucleus of the thalamus to the nucleus accumbens in the rat: an autoradiographic study. Exp Brain Res, 1984. 54(3): p. 499-512.

49. Vertes, R.P. and W.B. Hoover, Projections of the paraventricular and paratenial nuclei of the dorsal midline thalamus in the rat. J Comp Neurol, 2008. 508(2): p. 212-37.

50. Barone, F.C., J.T. Cheng, and M.J. Wayner, GABA inhibition of lateral hypothalamic neurons: role of reticular thalamic afferents. Brain Res Bull, 1994. 33(6): p. 699-708.

51. Bubser, M. and A.Y. Deutch, Thalamic paraventricular nucleus neurons collateralize to innervate the prefrontal cortex and nucleus accumbens. Brain Res, 1998. 787(2): p. 304-10.

52. Moga, M.M., R.P. Weis, and R.Y. Moore, Efferent projections of the paraventricular thalamic nucleus in the rat. J Comp Neurol, 1995. 359(2): p. 221-38.

53. Li, S. and G.J. Kirouac, Projections from the paraventricular nucleus of the thalamus to the forebrain, with special emphasis on the extended amygdala. J Comp Neurol, 2008. 506(2): p. 263-87.

54. Otake, K., Cholecystokinin and substance P immunoreactive projections to the paraventricular thalamic nucleus in the rat. Neurosci Res, 2005. 51(4): p. 383-94.

55. Parsons, M.P., S. Li, and G.J. Kirouac, Functional and anatomical connection between the paraventricular nucleus of the thalamus and dopamine fibers of the nucleus accumbens. J Comp Neurol, 2007. 500(6): p. 1050-63.

56. Su, H.S. and M. Bentivoglio, Thalamic midline cell populations projecting to the nucleus accumbens, amygdala, and hippocampus in the rat. J Comp Neurol, 1990. 297(4): p. 582-93.

57. Hasue, R.H. and S.J. Shammah-Lagnado, Origin of the dopaminergic innervation of the central extended amygdala and accumbens shell: a combined retrograde tracing and immunohistochemical study in the rat. J Comp Neurol, 2002. 454(1): p. 15-33.

58. Bubar, M.J., S.J. Stutz, and K.A. Cunningham, 5-HT(2C) receptors localize to dopamine and GABA neurons in the rat mesoaccumbens pathway. PLoS One, 2011. 6(6): p. e20508.

59. Wang, Z.J., Z.R. Rao, and J.W. Shi, Tyrosine hydroxylase-, neurotensin-, or cholecystokinin-containing neurons in the nucleus tractus solitarii send projection fibers to the nucleus accumbens in the rat. Brain Res, 1992. 578(1-2): p. 347-50.

**Caudate Putamen**

1. Nakashima, M., et al., An anterograde and retrograde tract-tracing study on the projections from the thalamic gustatory area in the rat: distribution of neurons projecting to the insular cortex and amygdaloid complex. Neurosci Res, 2000. 36(4): p. 297-309.

2. Bishop, G.A., H.T. Chang, and S.T. Kitai, Morphological and physiological properties of neostriatal neurons: an intracellular horseradish peroxidase study in the rat. Neuroscience, 1982. 7(1): p. 179-91.

3. Wu, Y., S. Richard, and A. Parent, The organization of the striatal output system: a single-cell juxtacellular labeling study in the rat. Neurosci Res, 2000. 38(1): p. 49-62.

4. Fink-Jensen, A. and J.D. Mikkelsen, The striato-entopeduncular pathway in the rat. A retrograde transport study with wheatgerm-agglutinin-horseradish peroxidase. Brain Res, 1989. 476(1): p. 194-8.

5. Brann, M.R. and P.C. Emson, Microiontophoretic injection of fluorescent tracer combined with simultaneous immunofluorescent histochemistry for the demonstration of efferents from the caudate-putamen projecting to the globus pallidus. Neurosci Lett, 1980. 16(1): p. 61-5.

6. Tallaksen-Greene, S.J. and R.L. Albin, Localization of AMPA-selective excitatory amino acid receptor subunits in identified populations of striatal neurons. Neuroscience, 1994. 61(3): p. 509-19.

7. Chang, H.T., C.J. Wilson, and S.T. Kitai, Single neostriatal efferent axons in the globus pallidus: a light and electron microscopic study. Science, 1981. 213(4510): p. 915-8.

8. Staines, W.A. and H.C. Fibiger, Collateral projections of neurons of the rat globus pallidus to the striatum and substantia nigra. Exp Brain Res, 1984. 56(2): p. 217-20.

9. Del Fiacco, M., G. Paxinos, and A.C. Cuello, Neostriatal enkephalin-immunoreactive neurones project to the globus pallidus. Brain Res, 1982. 231(1): p. 1-17.

10. Kita, H., Parvalbumin-immunopositive neurons in rat globus pallidus: a light and electron microscopic study. Brain Res, 1994. 657(1-2): p. 31-41.

11. Kincaid, A.E., et al., The globus pallidus receives a projection from the parafascicular nucleus in the rat. Brain Res, 1991. 553(1): p. 18-26.

12. Cornwall, J. and O.T. Phillipson, Afferent projections to the parafascicular thalamic nucleus of the rat, as shown by the retrograde transport of wheat germ agglutinin. Brain Res Bull, 1988. 20(2): p. 139-50.

13. Coolen, L.M., et al., Afferent connections of the parvocellular subparafascicular thalamic nucleus in the rat: evidence for functional subdivisions. J Comp Neurol, 2003. 463(2): p. 132-56.

14. Bunney, B.S. and G.K. Aghajanian, The precise localization of nigral afferents in the rat as determined by a retrograde tracing technique. Brain Res, 1976. 117(3): p. 423-35.

15. Hattori, T., H.C. Fibiger, and P.L. McGeer, Demonstration of a pallido-nigral projection innervating dopaminergic neurons. J Comp Neurol, 1975. 162(4): p. 487-504.

16. Jessell, T.M., et al., Topographic projections of substance P and GABA pathways in the striato- and pallido-nigral system: a biochemical and immunohistochemical study. Brain Res, 1978. 152(3): p. 487-98.

17. Somogyi, P. and A.D. Smith, Projection of neostriatal spiny neurons to the substantia nigra. Application of a combined Golgi-staining and horseradish peroxidase transport procedure at both light and electron microscopic levels. Brain Res, 1979. 178(1): p. 3-15.

18. Hong, J.S., et al., Projections of substance P containing neurons from neostriatum to substantia nigra. Brain Res, 1977. 122(3): p. 541-4.

19. Fallon, J.H., Collateralization of monoamine neurons: mesotelencephalic dopamine projections to caudate, septum, and frontal cortex. J Neurosci, 1981. 1(12): p. 1361-8.

20. Araki, M., P.L. McGeer, and E.G. McGeer, Striatonigral and pallidonigral pathways studied by a combination of retrograde horseradish peroxidase tracing and a pharmacohistochemical method for gamma-aminobutyric acid transaminase. Brain Res, 1985. 331(1): p. 17-24.

21. Bolam, J.P., et al., A second type of striatonigral neuron: a comparison between retrogradely labelled and Golgi-stained neurons at the light and electron microscopic levels. Neuroscience, 1981. 6(11): p. 2141-57.

22. Williams, M.N. and R.L. Faull, The striatonigral projection and nigrotectal neurons in the rat. A correlated light and electron microscopic study demonstrating a monosynaptic striatal input to identified nigrotectal neurons using a combined degeneration and horseradish peroxidase procedure. Neuroscience, 1985. 14(4): p. 991-1010.

23. Gerfen, C.R., The neostriatal mosaic: compartmentalization of corticostriatal input and striatonigral output systems. Nature, 1984. 311(5985): p. 461-4.

24. Gerfen, C.R., K.G. Baimbridge, and J.J. Miller, The neostriatal mosaic: compartmental distribution of calcium-binding protein and parvalbumin in the basal ganglia of the rat and monkey. Proc Natl Acad Sci U S A, 1985. 82(24): p. 8780-4.

25. Nicola, S.M., J. Surmeier, and R.C. Malenka, Dopaminergic modulation of neuronal excitability in the striatum and nucleus accumbens. Annu Rev Neurosci, 2000. 23: p. 185-215.

26. Carter, C.J., Topographical distribution of possible glutamatergic pathways from the frontal cortex to the striatum and substantia nigra in rats. Neuropharmacology, 1982. 21(5): p. 379-83.

27. Wang, X., P. Zhong, and Z. Yan, Dopamine D4 receptors modulate GABAergic signaling in pyramidal neurons of prefrontal cortex. J Neurosci, 2002. 22(21): p. 9185-93.

28. Vertes, R.P., Analysis of projections from the medial prefrontal cortex to the thalamus in the rat, with emphasis on nucleus reuniens. J Comp Neurol, 2002. 442(2): p. 163-87.

29. Sesack, S.R. and V.M. Pickel, Prefrontal cortical efferents in the rat synapse on unlabeled neuronal targets of catecholamine terminals in the nucleus accumbens septi and on dopamine neurons in the ventral tegmental area. J Comp Neurol, 1992. 320(2): p. 145-60.

30. Takagishi, M. and T. Chiba, Efferent projections of the infralimbic (area 25) region of the medial prefrontal cortex in the rat: an anterograde tracer PHA-L study. Brain Res, 1991. 566(1-2): p. 26-39.

31. Hardy, S.G., Projections to the midbrain from the medial versus lateral prefrontal cortices of the rat. Neurosci Lett, 1986. 63(2): p. 159-64.

32. McDonald, A.J., Organization of amygdaloid projections to the mediodorsal thalamus and prefrontal cortex: a fluorescence retrograde transport study in the rat. J Comp Neurol, 1987. 262(1): p. 46-58.

33. Gaykema, R.P., et al., Prefrontal cortical projections to the cholinergic neurons in the basal forebrain. J Comp Neurol, 1991. 303(4): p. 563-83.

34. Feger, J., M. Bevan, and A.R. Crossman, The projections from the parafascicular thalamic nucleus to the subthalamic nucleus and the striatum arise from separate neuronal populations: a comparison with the corticostriatal and corticosubthalamic efferents in a retrograde fluorescent double-labelling study. Neuroscience, 1994. 60(1): p. 125-32.

35. Morino, P., et al., Cholecystokinin corticostriatal pathway in the rat: evidence for bilateral origin from medial prefrontal cortical areas. Neuroscience, 1994. 59(4): p. 939-52.

36. Donoghue, J.P. and M. Herkenham, Neostriatal projections from individual cortical fields conform to histochemically distinct striatal compartments in the rat. Brain Res, 1986. 365(2): p. 397-403.

37. Sesack, S.R., et al., Topographical organization of the efferent projections of the medial prefrontal cortex in the rat: an anterograde tract-tracing study with Phaseolus vulgaris leucoagglutinin. J Comp Neurol, 1989. 290(2): p. 213-42.

38. McDonald, A.J., Organization of amygdaloid projections to the prefrontal cortex and associated striatum in the rat. Neuroscience, 1991. 44(1): p. 1-14.

39. Webster, K.E., Cortico-striate interrelations in the albino rat. J Anat, 1961. 95: p. 532-44.

40. Neafsey, E.J., K.M. Hurley-Gius, and D. Arvanitis, The topographical organization of neurons in the rat medial frontal, insular and olfactory cortex projecting to the solitary nucleus, olfactory bulb, periaqueductal gray and superior colliculus. Brain Res, 1986. 377(2): p. 261-70.

41. Jasmin, L., et al., Rostral agranular insular cortex and pain areas of the central nervous system: a tract-tracing study in the rat. J Comp Neurol, 2004. 468(3): p. 425-40.

42. Ohara, P.T., et al., Dopaminergic input to GABAergic neurons in the rostral agranular insular cortex of the rat. J Neurocytol, 2003. 32(2): p. 131-41.

43. Yasui, Y., et al., Autonomic responses and efferent pathways from the insular cortex in the rat. J Comp Neurol, 1991. 303(3): p. 355-74.

44. Shi, C.J. and M.D. Cassell, Cortical, thalamic, and amygdaloid connections of the anterior and posterior insular cortices. J Comp Neurol, 1998. 399(4): p. 440-68.

45. Usuda, I., K. Tanaka, and T. Chiba, Efferent projections of the nucleus accumbens in the rat with special reference to subdivision of the nucleus: biotinylated dextran amine study. Brain Res, 1998. 797(1): p. 73-93.

46. Dong, H., G.D. Petrovich, and L.W. Swanson, Organization of projections from the juxtacapsular nucleus of the BST: a PHAL study in the rat. Brain Res, 2000. 859(1): p. 1-14.

47. Takada, M., et al., Direct projections from the entopeduncular nucleus to the lower brainstem in the rat. J Comp Neurol, 1994. 342(3): p. 409-29.

48. Bevan, M.D., et al., Selective innervation of neostriatal interneurons by a subclass of neuron in the globus pallidus of the rat. J Neurosci, 1998. 18(22): p. 9438-52.

49. Kincaid, A.E., et al., Evidence for a projection from the globus pallidus to the entopeduncular nucleus in the rat. Neurosci Lett, 1991. 128(1): p. 121-5.

50. Shammah-Lagnado, S.J., G.F. Alheid, and L. Heimer, Efferent connections of the caudal part of the globus pallidus in the rat. J Comp Neurol, 1996. 376(3): p. 489-507.

51. Kohler, C., et al., The cytoarchitecture, histochemistry and projections of the tuberomammillary nucleus in the rat. Neuroscience, 1985. 16(1): p. 85-110.

52. McDonald, A.J., Topographical organization of amygdaloid projections to the caudatoputamen, nucleus accumbens, and related striatal-like areas of the rat brain. Neuroscience, 1991. 44(1): p. 15-33.

53. Kitai, S.T., Anatomy and physiology of the neostriatum. Adv Biochem Psychopharmacol, 1981. 30: p. 1-21.

54. Bourgeais, L., C. Gauriau, and J.F. Bernard, Projections from the nociceptive area of the central nucleus of the amygdala to the forebrain: a PHA-L study in the rat. Eur J Neurosci, 2001. 14(2): p. 229-55.

55. Petrovich, G.D., P.Y. Risold, and L.W. Swanson, Organization of projections from the basomedial nucleus of the amygdala: a PHAL study in the rat. J Comp Neurol, 1996. 374(3): p. 387-420.

56. McIntyre, D.C., M.E. Kelly, and W.A. Staines, Efferent projections of the anterior perirhinal cortex in the rat. J Comp Neurol, 1996. 369(2): p. 302-18.

57. Groenewegen, H.J., et al., Organization of the projections from the subiculum to the ventral striatum in the rat. A study using anterograde transport of Phaseolus vulgaris leucoagglutinin. Neuroscience, 1987. 23(1): p. 103-20.

58. Deschenes, M., J. Bourassa, and A. Parent, Two different types of thalamic fibers innervate the rat striatum. Brain Res, 1995. 701(1-2): p. 288-92.

59. Kamishina, H., et al., Striatal projections from the rat lateral posterior thalamic nucleus. Brain Res, 2008. 1204: p. 24-39.

60. Erro, M.E., et al., Striatal input from the ventrobasal complex of the rat thalamus. Histochem Cell Biol, 2001. 115(6): p. 447-54.

61. Miller, J.J., et al., Anatomical and electrophysiological identification of a projection from the mesencephalic raphe to the caudate-putamen in the rat. Brain Res, 1975. 97(1): p. 133-6.

62. Barrington-Ward, S.J., et al., Evidence that thalamic efferent neurones are non-cholinergic: a study in the rat with special reference to the thalamostriatal pathway. Brain Res, 1984. 299(1): p. 146-51.

63. Berendse, H.W. and H.J. Groenewegen, Organization of the thalamostriatal projections in the rat, with special emphasis on the ventral striatum. J Comp Neurol, 1990. 299(2): p. 187-228.

64. Vertes, R.P., W.B. Hoover, and J.J. Rodriguez, Projections of the central medial nucleus of the thalamus in the rat: node in cortical, striatal and limbic forebrain circuitry. Neuroscience, 2012. 219: p. 120-36.

65. Wright, C.I. and H.J. Groenewegen, Patterns of overlap and segregation between insular cortical, intermediodorsal thalamic and basal amygdaloid afferents in the nucleus accumbens of the rat. Neuroscience, 1996. 73(2): p. 359-73.

66. Kobayashi, K., et al., A possible monosynaptic pathway links the pedunculopontine tegmental nucleus to thalamostriatal neurons in the hooded rat. Arch Histol Cytol, 2007. 70(3): p. 207-14.

67. LeDoux, J.E., C. Farb, and D.A. Ruggiero, Topographic organization of neurons in the acoustic thalamus that project to the amygdala. J Neurosci, 1990. 10(4): p. 1043-54.

68. LeDoux, J.E., D.A. Ruggiero, and D.J. Reis, Projections to the subcortical forebrain from anatomically defined regions of the medial geniculate body in the rat. J Comp Neurol, 1985. 242(2): p. 182-213.

69. Ohtake, T. and H. Yamada, Efferent connections of the nucleus reuniens and the rhomboid nucleus in the rat: an anterograde PHA-L tracing study. Neurosci Res, 1989. 6(6): p. 556-68.

70. Herkenham, M., The afferent and efferent connections of the ventromedial thalamic nucleus in the rat. J Comp Neurol, 1979. 183(3): p. 487-517.

71. Kita, H. and S.T. Kitai, Efferent projections of the subthalamic nucleus in the rat: light and electron microscopic analysis with the PHA-L method. J Comp Neurol, 1987. 260(3): p. 435-52.

72. Chang, H.T., C.J. Wilson, and S.T. Kitai, A Golgi study of rat neostriatal neurons: light microscopic analysis. J Comp Neurol, 1982. 208(2): p. 107-26.

73. Nakano, K., Neural circuits and topographic organization of the basal ganglia and related regions. Brain Dev, 2000. 22 Suppl 1: p. S5-16.

74. Ricardo, J.A., Efferent connections of the subthalamic region in the rat. I. The subthalamic nucleus of Luys. Brain Res, 1980. 202(2): p. 257-71.

75. Oleshko, N.N., et al., [Divergence of axon collaterals of substantia nigra neurons in the forebrain of the rat: double labeling with fluorochromes and horseradish peroxidase]. Neirofiziologiia, 1983. 15(5): p. 517-26.

76. Hattori, T., et al., Analysis of the fine structure of the dopaminergic nigrostriatal projection by electron microscopic autoradiography. Exp Neurol, 1973. 41(3): p. 599-611.

77. Kawai, Y., et al., Nigrostriatal dopamine neurons receive substance P-ergic inputs in the substantia nigra: application of the immunoelectron microscopic mirror technique to fluorescent double-staining for transmitter-specific projections. Brain Res, 1987. 401(2): p. 371-6.

78. Loughlin, S.E. and J.H. Fallon, Mesostriatal projections from ventral tegmentum and dorsal raphe: cells project ipsilaterally or contralaterally but not bilaterally. Neurosci Lett, 1982. 32(1): p. 11-6.

79. Kita, H. and T. Kita, Number, origins, and chemical types of rat pallidostriatal projection neurons. J Comp Neurol, 2001. 437(4): p. 438-48.

80. Li, Y.Q., T. Kaneko, and N. Mizuno, Collateral projections of nucleus raphe dorsalis neurones to the caudate-putamen and region around the nucleus raphe magnus and nucleus reticularis gigantocellularis pars alpha in the rat. Neurosci Lett, 2001. 299(1-2): p. 33-6.

81. van der Kooy, D. and T. Hattori, Dorsal raphe cells with collateral projections to the caudate-putamen and substantia nigra: a fluorescent retrograde double labeling study in the rat. Brain Res, 1980. 186(1): p. 1-7.

82. Steinbusch, H.W., et al., Serotonergic and non-serotonergic projections from the nucleus raphe dorsalis to the caudate-putamen complex in the rat, studied by a combined immunofluorescence and fluorescent retrograde axonal labeling technique. Neurosci Lett, 1980. 19(2): p. 137-42.

83. Azmitia, E.C. and M. Segal, An autoradiographic analysis of the differential ascending projections of the dorsal and median raphe nuclei in the rat. J Comp Neurol, 1978. 179(3): p. 641-67.

84. van de Kar, L.D. and S.A. Lorens, Differential serotonergic innervation of individual hypothalamic nuclei and other forebrain regions by the dorsal and median midbrain raphe nuclei. Brain Res, 1979. 162(1): p. 45-54.

85. Descarries, L., et al., Dopaminergic projection from nucleus raphe dorsalis to neostriatum in the rat. J Comp Neurol, 1986. 249(4): p. 511-20, 484-5.

86. Mason, S.T. and H.C. Fibiger, Regional topography within noradrenergic locus coeruleus as revealed by retrograde transport of horseradish peroxidase. J Comp Neurol, 1979. 187(4): p. 703-24.

87. Schilman, E.A., et al., The orbital cortex in rats topographically projects to central parts of the caudate-putamen complex. Neurosci Lett, 2008. 432(1): p. 40-5.

88. Bennett, B.D. and J.P. Bolam, Synaptic input and output of parvalbumin-immunoreactive neurons in the neostriatum of the rat. Neuroscience, 1994. 62(3): p. 707-19.

89. Walaas, I., Biochemical evidence for overlapping neocortical and allocortical glutamate projections to the nucleus accumbens and rostral caudatoputamen in the rat brain. Neuroscience, 1981. 6(3): p. 399-405.

**Septal Region**

Intraregional connections

1. Leranth, C., T. Deller, and G. Buzsaki, Intraseptal connections redefined: lack of a lateral septum to medial septum path. Brain Res, 1992. 583(1-2): p. 1-11.

2. Onteniente, B., et al., Dopamine-GABA interactions in the nucleus accumbens and lateral septum of the rat. Brain Res, 1987. 421(1-2): p. 391-6.

Lateral Septum (efferents and afferents)

1. Brog, J.S., et al., The patterns of afferent innervation of the core and shell in the "accumbens" part of the rat ventral striatum: immunohistochemical detection of retrogradely transported fluoro-gold. J Comp Neurol, 1993. 338(2): p. 255-78.

2. Varoqueaux, F. and P. Poulain, Projections of the mediolateral part of the lateral septum to the hypothalamus, revealed by Fos expression and axonal tracing in rats. Anat Embryol (Berl), 1999. 199(3): p. 249-63.

3. Leranth, C., T. Deller, and G. Buzsaki, Intraseptal connections redefined: lack of a lateral septum to medial septum path. Brain Res, 1992. 583(1-2): p. 1-11.

4. Meibach, R.C. and A. Siegel, Efferent connections of the septal area in the rat: an analysis utilizing retrograde and anterograde transport methods. Brain Res, 1977. 119(1): p. 1-20.

5. Swanson, L.W., An autoradiographic study of the efferent connections of the preoptic region in the rat. J Comp Neurol, 1976. 167(2): p. 227-56.

6. Shibata, H., Descending projections to the mammillary nuclei in the rat, as studied by retrograde and anterograde transport of wheat germ agglutinin-horseradish peroxidase. J Comp Neurol, 1989. 285(4): p. 436-52.

7. Simerly, R.B. and L.W. Swanson, The organization of neural inputs to the medial preoptic nucleus of the rat. J Comp Neurol, 1986. 246(3): p. 312-42.

8. Abrahamson, E.E. and R.Y. Moore, The posterior hypothalamic area: chemoarchitecture and afferent connections. Brain Res, 2001. 889(1-2): p. 1-22.

9. Comoli, E., E.R. Ribeiro-Barbosa, and N.S. Canteras, Afferent connections of the dorsal premammillary nucleus. J Comp Neurol, 2000. 423(1): p. 83-98.

10. Staiger, J.F. and F.G. Wouterlood, Efferent projections from the lateral septal nucleus to the anterior hypothalamus in the rat: a study combining Phaseolus vulgaris-leucoagglutinin tracing with vasopressin immunocytochemistry. Cell Tissue Res, 1990. 261(1): p. 17-23.

11. Beaulieu, J., D. Champagne, and G. Drolet, Enkephalin innervation of the paraventricular nucleus of the hypothalamus: distribution of fibers and origins of input. J Chem Neuroanat, 1996. 10(2): p. 79-92.

12. Csaki, A., et al., Localization of glutamatergic/aspartatergic neurons projecting to the hypothalamic paraventricular nucleus studied by retrograde transport of [3H]D-aspartate autoradiography. Neuroscience, 2000. 101(3): p. 637-55.

13. Rutherford, J.G., An investigation of a possible direct projection from the medial nucleus of the cerebellum to the paraventricular nucleus of the hypothalamus in the rat: a study using retrograde WGA-HRP and Fluoro-Gold tracing techniques. Anat Embryol (Berl), 1995. 192(3): p. 229-38.

14. Moga, M.M. and R.Y. Moore, Organization of neural inputs to the suprachiasmatic nucleus in the rat. J Comp Neurol, 1997. 389(3): p. 508-34.

15. Borhegyi, Z. and T.F. Freund, Dual projection from the medial septum to the supramammillary nucleus in the rat. Brain Res Bull, 1998. 46(5): p. 453-9.

16. Kiss, J., et al., Possible glutamatergic/aspartatergic projections to the supramammillary nucleus and their origins in the rat studied by selective [(3)H]D-aspartate labelling and immunocytochemistry. Neuroscience, 2002. 111(3): p. 671-91.

17. Hayakawa, T., H. Ito, and K. Zyo, Neuroanatomical study of afferent projections to the supramammillary nucleus of the rat. Anat Embryol (Berl), 1993. 188(2): p. 139-48.

18. Fahrbach, S.E., J.I. Morrell, and D.W. Pfaff, Studies of ventromedial hypothalamic afferents in the rat using three methods of HRP application. Exp Brain Res, 1989. 77(2): p. 221-33.

19. Luiten, P.G. and P. Room, Interrelations between lateral, dorsomedial and ventromedial hypothalamic nuclei in the rat. An HRP study. Brain Res, 1980. 190(2): p. 321-32.

20. Canteras, N.S., R.B. Simerly, and L.W. Swanson, Connections of the posterior nucleus of the amygdala. J Comp Neurol, 1992. 324(2): p. 143-79.

21. Li, Y.Q., et al., The sites of origin of dopaminergic afferent fibers to the lateral habenular nucleus in the rat. J Comp Neurol, 1993. 333(1): p. 118-33.

22. Freund, T.F. and M. Antal, GABA-containing neurons in the septum control inhibitory interneurons in the hippocampus. Nature, 1988. 336(6195): p. 170-3.

23. Swanson, L.W., P.E. Sawchenko, and W.M. Cowan, Evidence for collateral projections by neurons in Ammon's horn, the dentate gyrus, and the subiculum: a multiple retrograde labeling study in the rat. J Neurosci, 1981. 1(5): p. 548-59.

24. Cornwall, J. and O.T. Phillipson, Afferent projections to the dorsal thalamus of the rat as shown by retrograde lectin transport. II. The midline nuclei. Brain Res Bull, 1988. 21(2): p. 147-61.

25. Chen, S. and H.S. Su, Afferent connections of the thalamic paraventricular and parataenial nuclei in the rat--a retrograde tracing study with iontophoretic application of Fluoro-Gold. Brain Res, 1990. 522(1): p. 1-6.

26. Cornwall, J. and O.T. Phillipson, Afferent projections to the parafascicular thalamic nucleus of the rat, as shown by the retrograde transport of wheat germ agglutinin. Brain Res Bull, 1988. 20(2): p. 139-50.

27. Herkenham, M., The connections of the nucleus reuniens thalami: evidence for a direct thalamo-hippocampal pathway in the rat. J Comp Neurol, 1978. 177(4): p. 589-610.

28. McKenna, J.T. and R.P. Vertes, Afferent projections to nucleus reuniens of the thalamus. J Comp Neurol, 2004. 480(2): p. 115-42.

29. Coolen, L.M., et al., Afferent connections of the parvocellular subparafascicular thalamic nucleus in the rat: evidence for functional subdivisions. J Comp Neurol, 2003. 463(2): p. 132-56.

30. Geisler, S. and D.S. Zahm, Afferents of the ventral tegmental area in the rat-anatomical substratum for integrative functions. J Comp Neurol, 2005. 490(3): p. 270-94.

31. Gaykema, R.P., et al., Prefrontal cortical projections to the cholinergic neurons in the basal forebrain. J Comp Neurol, 1991. 303(4): p. 563-83.

32. Sesack, S.R., et al., Topographical organization of the efferent projections of the medial prefrontal cortex in the rat: an anterograde tract-tracing study with Phaseolus vulgaris leucoagglutinin. J Comp Neurol, 1989. 290(2): p. 213-42.

33. Caffe, A.R., F.W. van Leeuwen, and P.G. Luiten, Vasopressin cells in the medial amygdala of the rat project to the lateral septum and ventral hippocampus. J Comp Neurol, 1987. 261(2): p. 237-52.

34. Dong, H.W. and L.W. Swanson, Organization of axonal projections from the anterolateral area of the bed nuclei of the stria terminalis. J Comp Neurol, 2004. 468(2): p. 277-98.

35. Dong, H.W. and L.W. Swanson, Projections from bed nuclei of the stria terminalis, dorsomedial nucleus: implications for cerebral hemisphere integration of neuroendocrine, autonomic, and drinking responses. J Comp Neurol, 2006. 494(1): p. 75-107.

36. Dong, H.-W. and L.W. Swanson, Projections from bed nuclei of the stria terminalis, magnocellular nucleus: Implications for cerebral hemisphere regulation of micturition, defecation, and penile erection. The Journal of Comparative Neurology, 2006. 494(1): p. 108-141.

37. Dong, H.W. and L.W. Swanson, Projections from bed nuclei of the stria terminalis, anteromedial area: cerebral hemisphere integration of neuroendocrine, autonomic, and behavioral aspects of energy balance. J Comp Neurol, 2006. 494(1): p. 142-78.

38. Dong, H.W., et al., Basic organization of projections from the oval and fusiform nuclei of the bed nuclei of the stria terminalis in adult rat brain. J Comp Neurol, 2001. 436(4): p. 430-55.

39. Dong, H.W. and L.W. Swanson, Projections from bed nuclei of the stria terminalis, posterior division: implications for cerebral hemisphere regulation of defensive and reproductive behaviors. J Comp Neurol, 2004. 471(4): p. 396-433.

40. Gu, G., A. Cornea, and R.B. Simerly, Sexual differentiation of projections from the principal nucleus of the bed nuclei of the stria terminalis. J Comp Neurol, 2003. 460(4): p. 542-62.

41. Shin, J.W., J.C. Geerling, and A.D. Loewy, Inputs to the ventrolateral bed nucleus of the stria terminalis. J Comp Neurol, 2008. 511(5): p. 628-57.

42. Sakanaka, M., et al., Corticotropin releasing factor-containing afferents to the lateral septum of the rat brain. J Comp Neurol, 1988. 270(3): p. 404-15, 396-7.

43. Risold, P.Y., N.S. Canteras, and L.W. Swanson, Organization of projections from the anterior hypothalamic nucleus: a Phaseolus vulgaris-leucoagglutinin study in the rat. J Comp Neurol, 1994. 348(1): p. 1-40.

44. Watts, A.G. and L.W. Swanson, Efferent projections of the suprachiasmatic nucleus: II. Studies using retrograde transport of fluorescent dyes and simultaneous peptide immunohistochemistry in the rat. J Comp Neurol, 1987. 258(2): p. 230-52.

45. Sim, L.J. and S.A. Joseph, Arcuate nucleus projections to brainstem regions which modulate nociception. J Chem Neuroanat, 1991. 4(2): p. 97-109.

46. Aarnisalo, A.A. and P. Panula, Neuropeptide FF-containing efferent projections from the medial hypothalamus of rat: a Phaseolus vulgaris leucoagglutinin study. Neuroscience, 1995. 65(1): p. 175-92.

47. ter Horst, G.J. and P.G. Luiten, The projections of the dorsomedial hypothalamic nucleus in the rat. Brain Res Bull, 1986. 16(2): p. 231-48.

48. Raisman, G., The connexions of the septum. Brain, 1966. 89(2): p. 317-48.

49. Berk, M.L. and J.A. Finkelstein, Efferent connections of the lateral hypothalamic area of the rat: an autoradiographic investigation. Brain Res Bull, 1982. 8(5): p. 511-26.

50. Goto, M., et al., Projections from the subfornical region of the lateral hypothalamic area. J Comp Neurol, 2005. 493(3): p. 412-38.

51. Simerly, R.B. and L.W. Swanson, Projections of the medial preoptic nucleus: a Phaseolus vulgaris leucoagglutinin anterograde tract-tracing study in the rat. J Comp Neurol, 1988. 270(2): p. 209-42.

52. Gu, G.B. and R.B. Simerly, Projections of the sexually dimorphic anteroventral periventricular nucleus in the female rat. J Comp Neurol, 1997. 384(1): p. 142-64.

53. Vertes, R.P., et al., Ascending projections of the posterior nucleus of the hypothalamus: PHA-L analysis in the rat. J Comp Neurol, 1995. 359(1): p. 90-116.

54. Canteras, N.S., R.B. Simerly, and L.W. Swanson, Projections of the ventral premammillary nucleus. J Comp Neurol, 1992. 324(2): p. 195-212.

55. Sakanaka, M. and S. Magari, Reassessment of enkephalin (ENK)-containing afferents to the rat lateral septum with reference to the fine structures of septal ENK fibers. Brain Res, 1989. 479(2): p. 205-16.

56. Leak, R.K. and R.Y. Moore, Topographic organization of suprachiasmatic nucleus projection neurons. J Comp Neurol, 2001. 433(3): p. 312-34.

57. Vertes, R.P., PHA-L analysis of projections from the supramammillary nucleus in the rat. J Comp Neurol, 1992. 326(4): p. 595-622.

58. Kiss, J., et al., The supramammillo-hippocampal and supramammillo-septal glutamatergic/aspartatergic projections in the rat: a combined [3H]D-aspartate autoradiographic and immunohistochemical study. Neuroscience, 2000. 97(4): p. 657-69.

59. Saper, C.B., L.W. Swanson, and W.M. Cowan, The efferent connections of the ventromedial nucleus of the hypothalamus of the rat. J Comp Neurol, 1976. 169(4): p. 409-42.

60. Hoffman, G.E., et al., The hypothalamic ventromedial nucleus sends a met-enkephalin projection to the preoptic area's periventricular zone in the female rat. Brain Res Mol Brain Res, 1996. 36(2): p. 201-10.

61. Petrovich, G.D., P.Y. Risold, and L.W. Swanson, Organization of projections from the basomedial nucleus of the amygdala: a PHAL study in the rat. J Comp Neurol, 1996. 374(3): p. 387-420.

62. Canteras, N.S., R.B. Simerly, and L.W. Swanson, Organization of projections from the medial nucleus of the amygdala: a PHAL study in the rat. J Comp Neurol, 1995. 360(2): p. 213-45.

63. Herkenham, M. and W.J. Nauta, Efferent connections of the habenular nuclei in the rat. J Comp Neurol, 1979. 187(1): p. 19-47.

64. van Groen, T. and J.M. Wyss, Extrinsic projections from area CA1 of the rat hippocampus: olfactory, cortical, subcortical, and bilateral hippocampal formation projections. J Comp Neurol, 1990. 302(3): p. 515-28.

65. Rose, A.M., T. Hattori, and H.C. Fibiger, Analysis of the septo-hippocampal pathway by light and electron microscopic autoradiography. Brain Res, 1976. 108(1): p. 170-4.

66. Schwerdtfeger, W.K. and E. Buhl, Various types of non-pyramidal hippocampal neurons project to the septum and contralateral hippocampus. Brain Res, 1986. 386(1-2): p. 146-54.

67. Fonnum, F. and I. Walaas, The effect of intrahippocampal kainic acid injections and surgical lesions on neurotransmitters in hippocampus and septum. J Neurochem, 1978. 31(5): p. 1173-81.

68. Shinoda, K., M. Tohyama, and Y. Shiotani, Hippocampofugal gamma-aminobutyric acid (GABA)-containing neuron system in the rat: a study using a double-labeling method that combines retrograde tracing and immunocytochemistry. Brain Res, 1987. 409(1): p. 181-6.

69. Raisman, G., W.M. Cowan, and T.P. Powell, An experimental analysis of the efferent projection of the hippocampus. Brain, 1966. 89(1): p. 83-108.

70. Swanson, L.W. and W.M. Cowan, Hippocampo-hypothalamic connections: origin in subicular cortex, not ammon's horn. Science, 1975. 189(4199): p. 303-4.

71. Calderazzo, L., et al., Branched connections to the septum and to the entorhinal cortex from the hippocampus, amygdala, and diencephalon in the rat. Brain Res Bull, 1996. 40(4): p. 245-51.

72. Alonso, A. and C. Kohler, A study of the reciprocal connections between the septum and the entorhinal area using anterograde and retrograde axonal transport methods in the rat brain. J Comp Neurol, 1984. 225(3): p. 327-43.

73. Alonso, J.R. and M. Frotscher, Hippocampo-septal fibers terminate on identified spiny neurons in the lateral septum: a combined Golgi/electron-microscopic and degeneration study in the rat. Cell Tissue Res, 1989. 258(2): p. 243-6.

74. Jakab, R.L. and C. Leranth, Catecholaminergic, GABAergic, and hippocamposeptal innervation of GABAergic "somatospiny" neurons in the rat lateral septal area. J Comp Neurol, 1990. 302(2): p. 305-21.

75. Leranth, C. and M. Frotscher, Organization of the septal region in the rat brain: cholinergic-GABAergic interconnections and the termination of hippocampo-septal fibers. J Comp Neurol, 1989. 289(2): p. 304-14.

76. Swanson, L.W. and W.M. Cowan, The connections of the septal region in the rat. J Comp Neurol, 1979. 186(4): p. 621-55.

77. Canteras, N.S. and L.W. Swanson, Projections of the ventral subiculum to the amygdala, septum, and hypothalamus: a PHAL anterograde tract-tracing study in the rat. J Comp Neurol, 1992. 324(2): p. 180-94.

78. Groenewegen, H.J., et al., Organization of the projections from the subiculum to the ventral striatum in the rat. A study using anterograde transport of Phaseolus vulgaris leucoagglutinin. Neuroscience, 1987. 23(1): p. 103-20.

79. Witter, M.P., R.H. Ostendorf, and H.J. Groenewegen, Heterogeneity in the Dorsal Subiculum of the Rat. Distinct Neuronal Zones Project to Different Cortical and Subcortical Targets. Eur J Neurosci, 1990. 2(8): p. 718-725.

80. Handelmann, G.E., et al., Extra-hippocampal projections of CCK neurons of the hippocampus and subiculum. Peptides, 1983. 4(3): p. 331-4.

81. Kelley, A.E. and L. Stinus, The distribution of the projection from the parataenial nucleus of the thalamus to the nucleus accumbens in the rat: an autoradiographic study. Exp Brain Res, 1984. 54(3): p. 499-512.

82. Vertes, R.P. and W.B. Hoover, Projections of the paraventricular and paratenial nuclei of the dorsal midline thalamus in the rat. J Comp Neurol, 2008. 508(2): p. 212-37.

83. Moga, M.M., R.P. Weis, and R.Y. Moore, Efferent projections of the paraventricular thalamic nucleus in the rat. J Comp Neurol, 1995. 359(2): p. 221-38.

84. Beckstead, R.M., V.B. Domesick, and W.J. Nauta, Efferent connections of the substantia nigra and ventral tegmental area in the rat. Brain Res, 1979. 175(2): p. 191-217.

85. Swanson, L.W., The projections of the ventral tegmental area and adjacent regions: a combined fluorescent retrograde tracer and immunofluorescence study in the rat. Brain Res Bull, 1982. 9(1-6): p. 321-53.

86. Onteniente, B., et al., Dopamine-GABA interactions in the nucleus accumbens and lateral septum of the rat. Brain Res, 1987. 421(1-2): p. 391-6.

87. Kohler, C., V. Chan-Palay, and H. Steinbusch, The distribution and origin of serotonin-containing fibers in the septal area: a combined immunohistochemical and fluorescent retrograde tracing study in the rat. J Comp Neurol, 1982. 209(1): p. 91-111.

88. Aznar, S., Z.X. Qian, and G.M. Knudsen, non-serotonergic dorsal and median raphe projection onto parvalbumin- and calbindin-containing neurons in hippocampus and septum. Neuroscience, 2004. 124(3): p. 573-81.

89. Vertes, R.P., A PHA-L analysis of ascending projections of the dorsal raphe nucleus in the rat. J Comp Neurol, 1991. 313(4): p. 643-68.

90. Stratford, T.R. and D. Wirtshafter, Ascending dopaminergic projections from the dorsal raphe nucleus in the rat. Brain Res, 1990. 511(1): p. 173-6.

91. Azmitia, E.C. and M. Segal, An autoradiographic analysis of the differential ascending projections of the dorsal and median raphe nuclei in the rat. J Comp Neurol, 1978. 179(3): p. 641-67.

92. Waselus, M., et al., Differential projections of dorsal raphe nucleus neurons to the lateral septum and striatum. J Chem Neuroanat, 2006. 31(4): p. 233-42.

93. Vertes, R.P. and G.F. Martin, Autoradiographic analysis of ascending projections from the pontine and mesencephalic reticular formation and the median raphe nucleus in the rat. J Comp Neurol, 1988. 275(4): p. 511-41.

94. Lindvall, O. and U. Stenevi, Dopamine and noradrenaline neurons projecting to the septal area in the rat. Cell Tissue Res, 1978. 190(3): p. 383-407.

95. Saper, C.B., Reciprocal parabrachial-cortical connections in the rat. Brain Res, 1982. 242(1): p. 33-40.

Medial Septum (efferents and afferents)

1. Swanson, L.W. and W.M. Cowan, The connections of the septal region in the rat. J Comp Neurol, 1979. 186(4): p. 621-55.

2. Moga, M.M. and R.Y. Moore, Organization of neural inputs to the suprachiasmatic nucleus in the rat. J Comp Neurol, 1997. 389(3): p. 508-34.

3. Borhegyi, Z. and T.F. Freund, Dual projection from the medial septum to the supramammillary nucleus in the rat. Brain Res Bull, 1998. 46(5): p. 453-9.

4. Kiss, J., et al., Possible glutamatergic/aspartatergic projections to the supramammillary nucleus and their origins in the rat studied by selective [(3)H]D-aspartate labelling and immunocytochemistry. Neuroscience, 2002. 111(3): p. 671-91.

5. Luiten, P.G. and P. Room, Interrelations between lateral, dorsomedial and ventromedial hypothalamic nuclei in the rat. An HRP study. Brain Res, 1980. 190(2): p. 321-32.

6. Fahrbach, S.E., J.I. Morrell, and D.W. Pfaff, Studies of ventromedial hypothalamic afferents in the rat using three methods of HRP application. Exp Brain Res, 1989. 77(2): p. 221-33.

7. Li, Y.Q., et al., The sites of origin of dopaminergic afferent fibers to the lateral habenular nucleus in the rat. J Comp Neurol, 1993. 333(1): p. 118-33.

8. Kawaja, M.D., B.A. Flumerfelt, and A.W. Hrycyshyn, Synaptic organization of septal projections in the rat medial habenula: a wheat germ agglutinin-horseradish peroxidase and immunohistochemical study. Synapse, 1990. 6(1): p. 45-54.

9. Yoshida, K. and H. Oka, Topographical projections from the medial septum-diagonal band complex to the hippocampus: a retrograde tracing study with multiple fluorescent dyes in rats. Neurosci Res, 1995. 21(3): p. 199-209.

10. Colom, L.V., et al., Characterization of medial septal glutamatergic neurons and their projection to the hippocampus. Synapse, 2005. 58(3): p. 151-64.

11. Nyakas, C., et al., Detailed projection patterns of septal and diagonal band efferents to the hippocampus in the rat with emphasis on innervation of CA1 and dentate gyrus. Brain Res Bull, 1987. 18(4): p. 533-45.

12. Pasquier, D.A. and F. Reinoso-Suarez, The topographic organization of hypothalamic and brain stem projections to the hippocampus. Brain Res Bull, 1978. 3(4): p. 373-89.

13. Pasquier, D.A. and F. Reinoso-Suarez, Direct projections from hypothalamus to hippocampus in the rat demonstrated by retrograde transport of horseradish peroxidase. Brain Res, 1976. 108(1): p. 165-9.

14. Miettinen, R. and T.F. Freund, Neuropeptide Y-containing interneurons in the hippocampus receive synaptic input from median raphe and GABAergic septal afferents. Neuropeptides, 1992. 22(3): p. 185-93.

15. Peterson, G.M. and C.L. Shurlow, Morphological evidence for a substance P projection from medial septum to hippocampus. Peptides, 1992. 13(3): p. 509-17.

16. Robinson, G.B. and R.J. Racine, Interactions between septal and entorhinal inputs to the rat dentate gyrus: facilitation effects. Brain Res, 1986. 379(1): p. 63-7.

17. Robinson, S.E., et al., Dopaminergic control of the septal-hippocampal cholinergic pathway. J Pharmacol Exp Ther, 1979. 208(3): p. 476-9.

18. Amaral, D.G. and J. Kurz, An analysis of the origins of the cholinergic and noncholinergic septal projections to the hippocampal formation of the rat. J Comp Neurol, 1985. 240(1): p. 37-59.

19. Alonso, A. and C. Kohler, Evidence for separate projections of hippocampal pyramidal and non-pyramidal neurons to different parts of the septum in the rat brain. Neurosci Lett, 1982. 31(3): p. 209-14.

20. Fonnum, F. and I. Walaas, The effect of intrahippocampal kainic acid injections and surgical lesions on neurotransmitters in hippocampus and septum. J Neurochem, 1978. 31(5): p. 1173-81.

21. Swanson, L.W. and W.M. Cowan, Hippocampo-hypothalamic connections: origin in subicular cortex, not ammon's horn. Science, 1975. 189(4199): p. 303-4.

22. Schwerdtfeger, W.K. and E. Buhl, Various types of non-pyramidal hippocampal neurons project to the septum and contralateral hippocampus. Brain Res, 1986. 386(1-2): p. 146-54.

23. Raisman, G., W.M. Cowan, and T.P. Powell, An experimental analysis of the efferent projection of the hippocampus. Brain, 1966. 89(1): p. 83-108.

24. Kohler, C., V. Chan-Palay, and J.Y. Wu, Septal neurons containing glutamic acid decarboxylase immunoreactivity project to the hippocampal region in the rat brain. Anat Embryol (Berl), 1984. 169(1): p. 41-4.

25. Alonso, A. and C. Kohler, A study of the reciprocal connections between the septum and the entorhinal area using anterograde and retrograde axonal transport methods in the rat brain. J Comp Neurol, 1984. 225(3): p. 327-43.

26. Beckstead, R.M., Afferent connections of the entorhinal area in the rat as demonstrated by retrograde cell-labeling with horseradish peroxidase. Brain Res, 1978. 152(2): p. 249-64.

27. Segal, M., Afferents to the entorhinal cortex of the rat studied by the method of retrograde transport of horseradish peroxidase. Exp Neurol, 1977. 57(3): p. 750-65.

28. Meibach, R.C. and A. Siegel, Thalamic projections of the hippocampal formation: evidence for an alternate pathway involving the internal capsule. Brain Res, 1977. 134(1): p. 1-12.

29. Onteniente, B., et al., Dopamine-GABA interactions in the nucleus accumbens and lateral septum of the rat. Brain Res, 1987. 421(1-2): p. 391-6.

30. Freund, T.F. and M. Antal, GABA-containing neurons in the septum control inhibitory interneurons in the hippocampus. Nature, 1988. 336(6195): p. 170-3.

31. Toth, K., Z. Borhegyi, and T.F. Freund, Postsynaptic targets of GABAergic hippocampal neurons in the medial septum-diagonal band of broca complex. J Neurosci, 1993. 13(9): p. 3712-24.

32. Milner, T.A. and D.G. Amaral, Evidence for a ventral septal projection to the hippocampal formation of the rat. Exp Brain Res, 1984. 55(3): p. 579-85.

33. Gaykema, R.P., et al., Cortical projection patterns of the medial septum-diagonal band complex. J Comp Neurol, 1990. 293(1): p. 103-24.

34. Peterson, G.M., Differential projections to the hippocampus by neurons of the medial septum and vertical limb of the diagonal band. Brain Res, 1994. 646(1): p. 129-34.

35. Yoshida, K. and H. Oka, Topographical distribution of septohippocampal projections demonstrated by the PHA-L immunohistochemical method in rats. Neurosci Lett, 1990. 113(3): p. 247-52.

36. Senut, M.C., F. De Bilbao, and Y. Lamour, Medial septal neurons containing N-acetyl-aspartyl-glutamate-like immunoreactivity project to the hippocampal formation in the rat. Neurosci Lett, 1990. 113(1): p. 12-6.

37. van Groen, T. and J.M. Wyss, The postsubicular cortex in the rat: characterization of the fourth region of the subicular cortex and its connections. Brain Res, 1990. 529(1-2): p. 165-77.

38. Gaykema, R.P. and L. Zaborszky, Direct catecholaminergic-cholinergic interactions in the basal forebrain. II. Substantia nigra-ventral tegmental area projections to cholinergic neurons. J Comp Neurol, 1996. 374(4): p. 555-77.

39. Arluison, M. and P. Derer, Forebrain connections of the rat paraventricular thalamic nucleus as demonstrated using the carbocyanide dye DiI. Neurobiology (Bp), 1993. 1(4): p. 337-50.

40. Chen, S. and M. Bentivoglio, Nerve growth factor receptor-containing cholinergic neurons of the basal forebrain project to the thalamic reticular nucleus in the rat. Brain Res, 1993. 606(2): p. 207-12.

41. Geisler, S. and D.S. Zahm, Afferents of the ventral tegmental area in the rat-anatomical substratum for integrative functions. J Comp Neurol, 2005. 490(3): p. 270-94.

42. Kalen, P. and L. Wiklund, Projections from the medial septum and diagonal band of Broca to the dorsal and central superior raphe nuclei: a non-cholinergic pathway. Exp Brain Res, 1989. 75(2): p. 401-16.

43. Behzadi, G., et al., Afferents to the median raphe nucleus of the rat: retrograde cholera toxin and wheat germ conjugated horseradish peroxidase tracing, and selective D-[3H]aspartate labelling of possible excitatory amino acid inputs. Neuroscience, 1990. 37(1): p. 77-100.

44. Gonzalo-Ruiz, A. and L. Morte, Localization of amino acids, neuropeptides and cholinergic markers in neurons of the septum-diagonal band complex projecting to the retrosplenial granular cortex of the rat. Brain Res Bull, 2000. 52(6): p. 499-510.

45. Sesack, S.R., et al., Topographical organization of the efferent projections of the medial prefrontal cortex in the rat: an anterograde tract-tracing study with Phaseolus vulgaris leucoagglutinin. J Comp Neurol, 1989. 290(2): p. 213-42.

46. Gaykema, R.P., et al., Prefrontal cortical projections to the cholinergic neurons in the basal forebrain. J Comp Neurol, 1991. 303(4): p. 563-83.

47. Risold, P.Y., N.S. Canteras, and L.W. Swanson, Organization of projections from the anterior hypothalamic nucleus: a Phaseolus vulgaris-leucoagglutinin study in the rat. J Comp Neurol, 1994. 348(1): p. 1-40.

48. Segal, M. and S.C. Landis, Afferents to the septal area of the rat studied with the method of retrograde axonal transport of horseradish peroxidase. Brain Res, 1974. 82(2): p. 263-8.

49. Saper, C.B., L.W. Swanson, and W.M. Cowan, An autoradiographic study of the efferent connections of the lateral hypothalamic area in the rat. J Comp Neurol, 1979. 183(4): p. 689-706.

50. Goto, M., et al., Projections from the subfornical region of the lateral hypothalamic area. J Comp Neurol, 2005. 493(3): p. 412-38.

51. Swanson, L.W., An autoradiographic study of the efferent connections of the preoptic region in the rat. J Comp Neurol, 1976. 167(2): p. 227-56.

52. Vertes, R.P., et al., Ascending projections of the posterior nucleus of the hypothalamus: PHA-L analysis in the rat. J Comp Neurol, 1995. 359(1): p. 90-116.

53. Swanson, L.W. and H.G. Kuypers, The paraventricular nucleus of the hypothalamus: cytoarchitectonic subdivisions and organization of projections to the pituitary, dorsal vagal complex, and spinal cord as demonstrated by retrograde fluorescence double-labeling methods. J Comp Neurol, 1980. 194(3): p. 555-70.

54. Gartner, U., et al., Immunofluorescence and immunoelectron microscopic evidence for differences in myelination of GABAergic and cholinergic septohippocampal fibres. Int J Dev Neurosci, 2001. 19(3): p. 347-52.

55. Kiss, J., et al., The supramammillo-hippocampal and supramammillo-septal glutamatergic/aspartatergic projections in the rat: a combined [3H]D-aspartate autoradiographic and immunohistochemical study. Neuroscience, 2000. 97(4): p. 657-69.

56. Borhegyi, Z., et al., The supramammillary nucleus innervates cholinergic and GABAergic neurons in the medial septum-diagonal band of Broca complex. Neuroscience, 1998. 82(4): p. 1053-65.

57. Vertes, R.P., PHA-L analysis of projections from the supramammillary nucleus in the rat. J Comp Neurol, 1992. 326(4): p. 595-622.

58. Canteras, N.S., R.B. Simerly, and L.W. Swanson, Organization of projections from the medial nucleus of the amygdala: a PHAL study in the rat. J Comp Neurol, 1995. 360(2): p. 213-45.

59. Raisman, G., The connexions of the septum. Brain, 1966. 89(2): p. 317-48.

60. Gulyas, A.I., et al., Interneurons are the local targets of hippocampal inhibitory cells which project to the medial septum. Eur J Neurosci, 2003. 17(9): p. 1861-72.

61. Calderazzo, L., et al., Branched connections to the septum and to the entorhinal cortex from the hippocampus, amygdala, and diencephalon in the rat. Brain Res Bull, 1996. 40(4): p. 245-51.

62. Leranth, C., T. Deller, and G. Buzsaki, Intraseptal connections redefined: lack of a lateral septum to medial septum path. Brain Res, 1992. 583(1-2): p. 1-11.

63. Canteras, N.S. and L.W. Swanson, Projections of the ventral subiculum to the amygdala, septum, and hypothalamus: a PHAL anterograde tract-tracing study in the rat. J Comp Neurol, 1992. 324(2): p. 180-94.

64. Bokor, H., et al., Cellular architecture of the nucleus reuniens thalami and its putative aspartatergic/glutamatergic projection to the hippocampus and medial septum in the rat. Eur J Neurosci, 2002. 16(7): p. 1227-39.

65. Kohler, C., V. Chan-Palay, and H. Steinbusch, The distribution and origin of serotonin-containing fibers in the septal area: a combined immunohistochemical and fluorescent retrograde tracing study in the rat. J Comp Neurol, 1982. 209(1): p. 91-111.

66. Aznar, S., Z.X. Qian, and G.M. Knudsen, non-serotonergic dorsal and median raphe projection onto parvalbumin- and calbindin-containing neurons in hippocampus and septum. Neuroscience, 2004. 124(3): p. 573-81.

67. Leranth, C. and R.P. Vertes, Median raphe serotonergic innervation of medial septum/diagonal band of broca (MSDB) parvalbumin-containing neurons: possible involvement of the MSDB in the desynchronization of the hippocampal EEG. J Comp Neurol, 1999. 410(4): p. 586-98.

68. van de Kar, L.D. and S.A. Lorens, Differential serotonergic innervation of individual hypothalamic nuclei and other forebrain regions by the dorsal and median midbrain raphe nuclei. Brain Res, 1979. 162(1): p. 45-54.

69. Azmitia, E.C. and M. Segal, An autoradiographic analysis of the differential ascending projections of the dorsal and median raphe nuclei in the rat. J Comp Neurol, 1978. 179(3): p. 641-67.

70. McKenna, J.T. and R.P. Vertes, Collateral projections from the median raphe nucleus to the medial septum and hippocampus. Brain Res Bull, 2001. 54(6): p. 619-30.

71. Lindvall, O. and U. Stenevi, Dopamine and noradrenaline neurons projecting to the septal area in the rat. Cell Tissue Res, 1978. 190(3): p. 383-407.

72. Montone, K.T., B. Fass, and G.S. Hamill, Serotonergic and nonserotonergic projections from the rat interpeduncular nucleus to the septum, hippocampal formation and raphe: a combined immunocytochemical and fluorescent retrograde labelling study of neurons in the apical subnucleus. Brain Res Bull, 1988. 20(2): p. 233-40.

Triangular Septal Nucleus (efferents and afferents)

1. Swanson, L.W. and W.M. Cowan, The connections of the septal region in the rat. J Comp Neurol, 1979. 186(4): p. 621-55.

2. Kawaja, M.D., B.A. Flumerfelt, and A.W. Hrycyshyn, Synaptic organization of septal projections in the rat medial habenula: a wheat germ agglutinin-horseradish peroxidase and immunohistochemical study. Synapse, 1990. 6(1): p. 45-54.

3. Wilson, J.A. and M.D. Kawaja, Distribution of calretinin-immunoreactive septal axons in the normal and deafferented medial habenula of adult rats. J Comp Neurol, 1996. 374(4): p. 593-606.

4. Sperlagh, B., et al., The triangular septal nucleus as the major source of ATP release in the rat habenula: a combined neurochemical and morphological study. Neuroscience, 1998. 86(4): p. 1195-207.

5. Staines, W.A., et al., Distribution, morphology and habenular projections of adenosine deaminase-containing neurons in the septal area of rat. Brain Res, 1988. 455(1): p. 72-87.

6. Herkenham, M. and W.J. Nauta, Afferent connections of the habenular nuclei in the rat. A horseradish peroxidase study, with a note on the fiber-of-passage problem. J Comp Neurol, 1977. 173(1): p. 123-46.

7. Contestabile, A. and F. Fonnum, Cholinergic and GABAergic forebrain projections to the habenula and nucleus interpeduncularis: surgical and kainic acid lesions. Brain Res, 1983. 275(2): p. 287-97.

8. Fonnum, F. and A. Contestabile, Colchicine neurotoxicity demonstrates the cholinergic projection from the supracommissural septum to the habenula and the nucleus interpeduncularis in the rat. J Neurochem, 1984. 43(3): p. 881-4.

9. Li, Y.Q., et al., The sites of origin of dopaminergic afferent fibers to the lateral habenular nucleus in the rat. J Comp Neurol, 1993. 333(1): p. 118-33.

10. Freund, T.F., GABAergic septohippocampal neurons contain parvalbumin. Brain Res, 1989. 478(2): p. 375-81.

11. Raisman, G., The connexions of the septum. Brain, 1966. 89(2): p. 317-48.

12. Canteras, N.S. and L.W. Swanson, Projections of the ventral subiculum to the amygdala, septum, and hypothalamus: a PHAL anterograde tract-tracing study in the rat. J Comp Neurol, 1992. 324(2): p. 180-94.

13. Azmitia, E.C. and M. Segal, An autoradiographic analysis of the differential ascending projections of the dorsal and median raphe nuclei in the rat. J Comp Neurol, 1978. 179(3): p. 641-67.

Septofimbrial Nucleus (efferents and afferents)

1. Simerly, R.B. and L.W. Swanson, The organization of neural inputs to the medial preoptic nucleus of the rat. J Comp Neurol, 1986. 246(3): p. 312-42.

2. Kawaja, M.D., B.A. Flumerfelt, and A.W. Hrycyshyn, Synaptic organization of septal projections in the rat medial habenula: a wheat germ agglutinin-horseradish peroxidase and immunohistochemical study. Synapse, 1990. 6(1): p. 45-54.

3. Swanson, L.W. and W.M. Cowan, The connections of the septal region in the rat. J Comp Neurol, 1979. 186(4): p. 621-55.

4. Contestabile, A. and F. Fonnum, Cholinergic and GABAergic forebrain projections to the habenula and nucleus interpeduncularis: surgical and kainic acid lesions. Brain Res, 1983. 275(2): p. 287-97.

5. Herkenham, M. and W.J. Nauta, Afferent connections of the habenular nuclei in the rat. A horseradish peroxidase study, with a note on the fiber-of-passage problem. J Comp Neurol, 1977. 173(1): p. 123-46.

6. Staines, W.A., et al., Distribution, morphology and habenular projections of adenosine deaminase-containing neurons in the septal area of rat. Brain Res, 1988. 455(1): p. 72-87.

7. Sperlagh, B., et al., The triangular septal nucleus as the major source of ATP release in the rat habenula: a combined neurochemical and morphological study. Neuroscience, 1998. 86(4): p. 1195-207.

8. Wilson, J.A. and M.D. Kawaja, Distribution of calretinin-immunoreactive septal axons in the normal and deafferented medial habenula of adult rats. J Comp Neurol, 1996. 374(4): p. 593-606.

9. Kim, U. and S.Y. Chang, Dendritic morphology, local circuitry, and intrinsic electrophysiology of neurons in the rat medial and lateral habenular nuclei of the epithalamus. J Comp Neurol, 2005. 483(2): p. 236-50.

10. Li, Y.Q., et al., The sites of origin of dopaminergic afferent fibers to the lateral habenular nucleus in the rat. J Comp Neurol, 1993. 333(1): p. 118-33.

11. Fonnum, F. and A. Contestabile, Colchicine neurotoxicity demonstrates the cholinergic projection from the supracommissural septum to the habenula and the nucleus interpeduncularis in the rat. J Neurochem, 1984. 43(3): p. 881-4.

12. Freund, T.F. and M. Antal, GABA-containing neurons in the septum control inhibitory interneurons in the hippocampus. Nature, 1988. 336(6195): p. 170-3.

13. Ray, J.P., et al., Sources of presumptive glutamatergic/aspartatergic afferents to the mediodorsal nucleus of the thalamus in the rat. J Comp Neurol, 1992. 320(4): p. 435-56.

14. Geisler, S. and D.S. Zahm, Afferents of the ventral tegmental area in the rat-anatomical substratum for integrative functions. J Comp Neurol, 2005. 490(3): p. 270-94.

15. Risold, P.Y., N.S. Canteras, and L.W. Swanson, Organization of projections from the anterior hypothalamic nucleus: a Phaseolus vulgaris-leucoagglutinin study in the rat. J Comp Neurol, 1994. 348(1): p. 1-40.

16. Canteras, N.S., R.B. Simerly, and L.W. Swanson, Organization of projections from the medial nucleus of the amygdala: a PHAL study in the rat. J Comp Neurol, 1995. 360(2): p. 213-45.

17. Raisman, G., The connexions of the septum. Brain, 1966. 89(2): p. 317-48.

18. Raisman, G., W.M. Cowan, and T.P. Powell, An experimental analysis of the efferent projection of the hippocampus. Brain, 1966. 89(1): p. 83-108.

19. Canteras, N.S. and L.W. Swanson, Projections of the ventral subiculum to the amygdala, septum, and hypothalamus: a PHAL anterograde tract-tracing study in the rat. J Comp Neurol, 1992. 324(2): p. 180-94.

20. Vertes, R.P. and G.F. Martin, Autoradiographic analysis of ascending projections from the pontine and mesencephalic reticular formation and the median raphe nucleus in the rat. J Comp Neurol, 1988. 275(4): p. 511-41.

21. Lindvall, O. and U. Stenevi, Dopamine and noradrenaline neurons projecting to the septal area in the rat. Cell Tissue Res, 1978. 190(3): p. 383-407.

Nucleus of the Diagonal Band (efferents and afferents)

1. Gaykema, R.P., et al., Cortical projection patterns of the medial septum-diagonal band complex. J Comp Neurol, 1990. 293(1): p. 103-24.

2. Zaborszky, L., et al., Cholinergic and GABAergic afferents to the olfactory bulb in the rat with special emphasis on the projection neurons in the nucleus of the horizontal limb of the diagonal band. J Comp Neurol, 1986. 243(4): p. 488-509.

3. Okoyama, S., et al., Cholinergic divergent projections from rat basal forebrain to the hippocampus and olfactory bulb. Neurosci Lett, 1987. 83(1-2): p. 77-81.

4. Gracia-Llanes, F.J., et al., GABAergic basal forebrain afferents innervate selectively GABAergic targets in the main olfactory bulb. Neuroscience, 2010. 170(3): p. 913-22.

5. de Olmos, J., H. Hardy, and L. Heimer, The afferent connections of the main and the accessory olfactory bulb formations in the rat: an experimental HRP-study. J Comp Neurol, 1978. 181(2): p. 213-44.

6. Swanson, L.W. and W.M. Cowan, The connections of the septal region in the rat. J Comp Neurol, 1979. 186(4): p. 621-55.

7. Meibach, R.C. and A. Siegel, Efferent connections of the septal area in the rat: an analysis utilizing retrograde and anterograde transport methods. Brain Res, 1977. 119(1): p. 1-20.

8. Gonzalo-Ruiz, A., et al., Afferent projections to the mammillary complex of the rat, with special reference to those from surrounding hypothalamic regions. J Comp Neurol, 1992. 321(2): p. 277-99.

9. Shibata, H., Descending projections to the mammillary nuclei in the rat, as studied by retrograde and anterograde transport of wheat germ agglutinin-horseradish peroxidase. J Comp Neurol, 1989. 285(4): p. 436-52.

10. Abrahamson, E.E. and R.Y. Moore, The posterior hypothalamic area: chemoarchitecture and afferent connections. Brain Res, 2001. 889(1-2): p. 1-22.

11. Moga, M.M. and R.Y. Moore, Organization of neural inputs to the suprachiasmatic nucleus in the rat. J Comp Neurol, 1997. 389(3): p. 508-34.

12. Jhamandas, J.H., et al., Diagonal band projection towards the hypothalamic supraoptic nucleus: light and electron microscopic observations in the rat. J Comp Neurol, 1989. 282(1): p. 15-23.

13. Hayakawa, T., H. Ito, and K. Zyo, Neuroanatomical study of afferent projections to the supramammillary nucleus of the rat. Anat Embryol (Berl), 1993. 188(2): p. 139-48.

14. Kiss, J., et al., Possible glutamatergic/aspartatergic projections to the supramammillary nucleus and their origins in the rat studied by selective [(3)H]D-aspartate labelling and immunocytochemistry. Neuroscience, 2002. 111(3): p. 671-91.

15. Fahrbach, S.E., J.I. Morrell, and D.W. Pfaff, Studies of ventromedial hypothalamic afferents in the rat using three methods of HRP application. Exp Brain Res, 1989. 77(2): p. 221-33.

16. Ottersen, O.P., Afferent connections to the amygdaloid complex of the rat and cat: II. Afferents from the hypothalamus and the basal telencephalon. J Comp Neurol, 1980. 194(1): p. 267-89.

17. Kim, U. and S.Y. Chang, Dendritic morphology, local circuitry, and intrinsic electrophysiology of neurons in the rat medial and lateral habenular nuclei of the epithalamus. J Comp Neurol, 2005. 483(2): p. 236-50.

18. Li, Y.Q., et al., The sites of origin of dopaminergic afferent fibers to the lateral habenular nucleus in the rat. J Comp Neurol, 1993. 333(1): p. 118-33.

19. Herkenham, M. and W.J. Nauta, Afferent connections of the habenular nuclei in the rat. A horseradish peroxidase study, with a note on the fiber-of-passage problem. J Comp Neurol, 1977. 173(1): p. 123-46.

20. Qu, T., et al., Demonstration of direct input from the retina to the lateral habenular nucleus in the albino rat. Brain Res, 1996. 709(2): p. 251-58.

21. Contestabile, A. and F. Fonnum, Cholinergic and GABAergic forebrain projections to the habenula and nucleus interpeduncularis: surgical and kainic acid lesions. Brain Res, 1983. 275(2): p. 287-97.

22. Contestabile, A. and B.A. Flumerfelt, Afferent connections of the interpeduncular nucleus and the topographic organization of the habenulo-interpeduncular pathway: an HRP study in the rat. J Comp Neurol, 1981. 196(2): p. 253-70.

23. Yoshida, K. and H. Oka, Topographical projections from the medial septum-diagonal band complex to the hippocampus: a retrograde tracing study with multiple fluorescent dyes in rats. Neurosci Res, 1995. 21(3): p. 199-209.

24. Colom, L.V., et al., Characterization of medial septal glutamatergic neurons and their projection to the hippocampus. Synapse, 2005. 58(3): p. 151-64.

25. Pasquier, D.A. and F. Reinoso-Suarez, The topographic organization of hypothalamic and brain stem projections to the hippocampus. Brain Res Bull, 1978. 3(4): p. 373-89.

26. Nyakas, C., et al., Detailed projection patterns of septal and diagonal band efferents to the hippocampus in the rat with emphasis on innervation of CA1 and dentate gyrus. Brain Res Bull, 1987. 18(4): p. 533-45.

27. Miettinen, R. and T.F. Freund, Neuropeptide Y-containing interneurons in the hippocampus receive synaptic input from median raphe and GABAergic septal afferents. Neuropeptides, 1992. 22(3): p. 185-93.

28. Segal, M. and S. Landis, Afferents to the hippocampus of the rat studied with the method of retrograde transport of horseradish peroxidase. Brain Res, 1974. 78(1): p. 1-15.

29. Sakanaka, M., et al., Topographic organization of the projection from the forebrain subcortical areas to the hippocampal formation of the rat. Neurosci Lett, 1980. 20(3): p. 253-7.

30. Amaral, D.G. and J. Kurz, An analysis of the origins of the cholinergic and noncholinergic septal projections to the hippocampal formation of the rat. J Comp Neurol, 1985. 240(1): p. 37-59.

31. Kohler, C., V. Chan-Palay, and J.Y. Wu, Septal neurons containing glutamic acid decarboxylase immunoreactivity project to the hippocampal region in the rat brain. Anat Embryol (Berl), 1984. 169(1): p. 41-4.

32. Gaykema, R.P. and L. Zaborszky, Direct catecholaminergic-cholinergic interactions in the basal forebrain. II. Substantia nigra-ventral tegmental area projections to cholinergic neurons. J Comp Neurol, 1996. 374(4): p. 555-77.

33. Alonso, A. and C. Kohler, A study of the reciprocal connections between the septum and the entorhinal area using anterograde and retrograde axonal transport methods in the rat brain. J Comp Neurol, 1984. 225(3): p. 327-43.

34. Meibach, R.C. and A. Siegel, Thalamic projections of the hippocampal formation: evidence for an alternate pathway involving the internal capsule. Brain Res, 1977. 134(1): p. 1-12.

35. Meibach, R.C. and A. Siegel, Efferent connections of the hippocampal formation in the rat. Brain Res, 1977. 124(2): p. 197-224.

36. Segal, M., Afferents to the entorhinal cortex of the rat studied by the method of retrograde transport of horseradish peroxidase. Exp Neurol, 1977. 57(3): p. 750-65.

37. Beckstead, R.M., Afferent connections of the entorhinal area in the rat as demonstrated by retrograde cell-labeling with horseradish peroxidase. Brain Res, 1978. 152(2): p. 249-64.

38. Onteniente, B., et al., Dopamine-GABA interactions in the nucleus accumbens and lateral septum of the rat. Brain Res, 1987. 421(1-2): p. 391-6.

39. Freund, T.F. and M. Antal, GABA-containing neurons in the septum control inhibitory interneurons in the hippocampus. Nature, 1988. 336(6195): p. 170-3.

40. Toth, K., Z. Borhegyi, and T.F. Freund, Postsynaptic targets of GABAergic hippocampal neurons in the medial septum-diagonal band of broca complex. J Neurosci, 1993. 13(9): p. 3712-24.

41. Milner, T.A. and D.G. Amaral, Evidence for a ventral septal projection to the hippocampal formation of the rat. Exp Brain Res, 1984. 55(3): p. 579-85.

42. Peterson, G.M., Differential projections to the hippocampus by neurons of the medial septum and vertical limb of the diagonal band. Brain Res, 1994. 646(1): p. 129-34.

43. Yoshida, K. and H. Oka, Topographical distribution of septohippocampal projections demonstrated by the PHA-L immunohistochemical method in rats. Neurosci Lett, 1990. 113(3): p. 247-52.

44. Senut, M.C., F. De Bilbao, and Y. Lamour, Medial septal neurons containing N-acetyl-aspartyl-glutamate-like immunoreactivity project to the hippocampal formation in the rat. Neurosci Lett, 1990. 113(1): p. 12-6.

45. Deacon, T.W., et al., Afferent connections of the perirhinal cortex in the rat. J Comp Neurol, 1983. 220(2): p. 168-90.

46. van Groen, T. and J.M. Wyss, The postsubicular cortex in the rat: characterization of the fourth region of the subicular cortex and its connections. Brain Res, 1990. 529(1-2): p. 165-77.

47. Mogenson, G.J., et al., Ventral pallidum projections to mediodorsal nucleus of the thalamus: an anatomical and electrophysiological investigation in the rat. Brain Res, 1987. 404(1-2): p. 221-30.

48. Groenewegen, H.J., Organization of the afferent connections of the mediodorsal thalamic nucleus in the rat, related to the mediodorsal-prefrontal topography. Neuroscience, 1988. 24(2): p. 379-431.

49. Ray, J.P., et al., Sources of presumptive glutamatergic/aspartatergic afferents to the mediodorsal nucleus of the thalamus in the rat. J Comp Neurol, 1992. 320(4): p. 435-56.

50. Hallanger, A.E., et al., The origins of cholinergic and other subcortical afferents to the thalamus in the rat. J Comp Neurol, 1987. 262(1): p. 105-24.

51. Price, J.L. and B.M. Slotnick, Dual olfactory representation in the rat thalamus: an anatomical and electrophysiological study. J Comp Neurol, 1983. 215(1): p. 63-77.

52. Yoshida, A., J.O. Dostrovsky, and C.Y. Chiang, The afferent and efferent connections of the nucleus submedius in the rat. J Comp Neurol, 1992. 324(1): p. 115-33.

53. Herkenham, M., The connections of the nucleus reuniens thalami: evidence for a direct thalamo-hippocampal pathway in the rat. J Comp Neurol, 1978. 177(4): p. 589-610.

54. Cornwall, J., J.D. Cooper, and O.T. Phillipson, Projections to the rostral reticular thalamic nucleus in the rat. Exp Brain Res, 1990. 80(1): p. 157-71.

55. Chen, S. and M. Bentivoglio, Nerve growth factor receptor-containing cholinergic neurons of the basal forebrain project to the thalamic reticular nucleus in the rat. Brain Res, 1993. 606(2): p. 207-12.

56. Geisler, S. and D.S. Zahm, Afferents of the ventral tegmental area in the rat-anatomical substratum for integrative functions. J Comp Neurol, 2005. 490(3): p. 270-94.

57. Aghajanian, G.K. and R.Y. Wang, Habenular and other midbrain raphe afferents demonstrated by a modified retrograde tracing technique. Brain Res, 1977. 122(2): p. 229-42.

58. Kalen, P., G. Skagerberg, and O. Lindvall, Projections from the ventral tegmental area and mesencephalic raphe to the dorsal raphe nucleus in the rat. Evidence for a minor dopaminergic component. Exp Brain Res, 1988. 73(1): p. 69-77.

59. Sim, L.J. and S.A. Joseph, Opiocortin and catecholamine projections to raphe nuclei. Peptides, 1989. 10(5): p. 1019-25.

60. Behzadi, G., et al., Afferents to the median raphe nucleus of the rat: retrograde cholera toxin and wheat germ conjugated horseradish peroxidase tracing, and selective D-[3H]aspartate labelling of possible excitatory amino acid inputs. Neuroscience, 1990. 37(1): p. 77-100.

61. Kalen, P. and L. Wiklund, Projections from the medial septum and diagonal band of Broca to the dorsal and central superior raphe nuclei: a non-cholinergic pathway. Exp Brain Res, 1989. 75(2): p. 401-16.

62. Lee, H.S., M.A. Kim, and B.D. Waterhouse, Retrograde double-labeling study of common afferent projections to the dorsal raphe and the nuclear core of the locus coeruleus in the rat. J Comp Neurol, 2005. 481(2): p. 179-93.

63. Lamour, Y., P. Dutar, and A. Jobert, Cortical projections of the nucleus of the diagonal band of Broca and of the substantia innominata in the rat: an anatomical study using the anterograde transport of a conjugate of wheat germ agglutinin and horseradish peroxidase. Neuroscience, 1984. 12(2): p. 395-408.

64. Gottesfeld, Z. and D.M. Jacobowitz, Cholinergic projection of the diagonal band to the interpeduncular nucleus of the rat brain. Brain Res, 1978. 156(2): p. 329-32.

65. Gonzalo-Ruiz, A. and L. Morte, Localization of amino acids, neuropeptides and cholinergic markers in neurons of the septum-diagonal band complex projecting to the retrosplenial granular cortex of the rat. Brain Res Bull, 2000. 52(6): p. 499-510.

66. Gaykema, R.P., et al., Prefrontal cortical projections to the cholinergic neurons in the basal forebrain. J Comp Neurol, 1991. 303(4): p. 563-83.

67. Sesack, S.R., et al., Topographical organization of the efferent projections of the medial prefrontal cortex in the rat: an anterograde tract-tracing study with Phaseolus vulgaris leucoagglutinin. J Comp Neurol, 1989. 290(2): p. 213-42.

68. Dong, H.W. and L.W. Swanson, Projections from bed nuclei of the stria terminalis, posterior division: implications for cerebral hemisphere regulation of defensive and reproductive behaviors. J Comp Neurol, 2004. 471(4): p. 396-433.

69. Risold, P.Y., N.S. Canteras, and L.W. Swanson, Organization of projections from the anterior hypothalamic nucleus: a Phaseolus vulgaris-leucoagglutinin study in the rat. J Comp Neurol, 1994. 348(1): p. 1-40.

70. Conrad, L.C. and D.W. Pfaff, Efferents from medial basal forebrain and hypothalamus in the rat. II. An autoradiographic study of the anterior hypothalamus. J Comp Neurol, 1976. 169(2): p. 221-61.

71. Aarnisalo, A.A. and P. Panula, Neuropeptide FF-containing efferent projections from the medial hypothalamus of rat: a Phaseolus vulgaris leucoagglutinin study. Neuroscience, 1995. 65(1): p. 175-92.

72. Raisman, G., The connexions of the septum. Brain, 1966. 89(2): p. 317-48.

73. Berk, M.L. and J.A. Finkelstein, Efferent connections of the lateral hypothalamic area of the rat: an autoradiographic investigation. Brain Res Bull, 1982. 8(5): p. 511-26..

74. Berk, M.L. and J.A. Finkelstein, Efferent connections of the lateral hypothalamic area of the rat: an autoradiographic investigation. Brain Res Bull, 1982. 8(5): p. 511-26.

75. Swanson, L.W., An autoradiographic study of the efferent connections of the preoptic region in the rat. J Comp Neurol, 1976. 167(2): p. 227-56.

76. Gu, G.B. and R.B. Simerly, Projections of the sexually dimorphic anteroventral periventricular nucleus in the female rat. J Comp Neurol, 1997. 384(1): p. 142-64.

77. Vertes, R.P., et al., Ascending projections of the posterior nucleus of the hypothalamus: PHA-L analysis in the rat. J Comp Neurol, 1995. 359(1): p. 90-116.

78. Vertes, R.P., PHA-L analysis of projections from the supramammillary nucleus in the rat. J Comp Neurol, 1992. 326(4): p. 595-622.

79. Borhegyi, Z., et al., The supramammillary nucleus innervates cholinergic and GABAergic neurons in the medial septum-diagonal band of Broca complex. Neuroscience, 1998. 82(4): p. 1053-65.

80. Canteras, N.S., R.B. Simerly, and L.W. Swanson, Organization of projections from the medial nucleus of the amygdala: a PHAL study in the rat. J Comp Neurol, 1995. 360(2): p. 213-45.

81. Caffe, A.R., F.W. van Leeuwen, and P.G. Luiten, Vasopressin cells in the medial amygdala of the rat project to the lateral septum and ventral hippocampus. J Comp Neurol, 1987. 261(2): p. 237-52.

82. Araki, M., P.L. McGeer, and H. Kimura, The efferent projections of the rat lateral habenular nucleus revealed by the PHA-L anterograde tracing method. Brain Res, 1988. 441(1-2): p. 319-30.

83. Raisman, G., W.M. Cowan, and T.P. Powell, An experimental analysis of the efferent projection of the hippocampus. Brain, 1966. 89(1): p. 83-108.

84. Canteras, N.S. and L.W. Swanson, Projections of the ventral subiculum to the amygdala, septum, and hypothalamus: a PHAL anterograde tract-tracing study in the rat. J Comp Neurol, 1992. 324(2): p. 180-94.

85. Swanson, L.W., The projections of the ventral tegmental area and adjacent regions: a combined fluorescent retrograde tracer and immunofluorescence study in the rat. Brain Res Bull, 1982. 9(1-6): p. 321-53.

86. Beckstead, R.M., V.B. Domesick, and W.J. Nauta, Efferent connections of the substantia nigra and ventral tegmental area in the rat. Brain Res, 1979. 175(2): p. 191-217.

87. Kalivas, P.W., L. Jennes, and J.S. Miller, A catecholaminergic projection from the ventral tegmental area to the diagonal band of Broca: modulation by neurotensin. Brain Res, 1985. 326(2): p. 229-38.

88. Kalivas, P.W. and J.S. Miller, Neurotensin neurons in the ventral tegmental area project to the medial nucleus accumbens. Brain Res, 1984. 300(1): p. 157-60.

89. Kohler, C., V. Chan-Palay, and H. Steinbusch, The distribution and origin of serotonin-containing fibers in the septal area: a combined immunohistochemical and fluorescent retrograde tracing study in the rat. J Comp Neurol, 1982. 209(1): p. 91-111.

90. Aznar, S., Z.X. Qian, and G.M. Knudsen, non-serotonergic dorsal and median raphe projection onto parvalbumin- and calbindin-containing neurons in hippocampus and septum. Neuroscience, 2004. 124(3): p. 573-81.

91. Vertes, R.P., A PHA-L analysis of ascending projections of the dorsal raphe nucleus in the rat. J Comp Neurol, 1991. 313(4): p. 643-68.

92. Leranth, C. and R.P. Vertes, Median raphe serotonergic innervation of medial septum/diagonal band of broca (MSDB) parvalbumin-containing neurons: possible involvement of the MSDB in the desynchronization of the hippocampal EEG. J Comp Neurol, 1999. 410(4): p. 586-98.

93. Vertes, R.P. and G.F. Martin, Autoradiographic analysis of ascending projections from the pontine and mesencephalic reticular formation and the median raphe nucleus in the rat. J Comp Neurol, 1988. 275(4): p. 511-41.

94. Lindvall, O. and U. Stenevi, Dopamine and noradrenaline neurons projecting to the septal area in the rat. Cell Tissue Res, 1978. 190(3): p. 383-407.

95. Alden, M., J.M. Besson, and J.F. Bernard, Organization of the efferent projections from the pontine parabrachial area to the bed nucleus of the stria terminalis and neighboring regions: a PHA-L study in the rat. J Comp Neurol, 1994. 341(3): p. 289-314.

96. Krukoff, T.L., K.H. Harris, and J.H. Jhamandas, Efferent projections from the parabrachial nucleus demonstrated with the anterograde tracer Phaseolus vulgaris leucoagglutinin. Brain Res Bull, 1993. 30(1-2): p. 163-72.

97. Saper, C.B., Reciprocal parabrachial-cortical connections in the rat. Brain Res, 1982. 242(1): p. 33-40.

98. Saper, C.B. and A.D. Loewy, Efferent connections of the parabrachial nucleus in the rat. Brain Res, 1980. 197(2): p. 291-317.

99. Montone, K.T., B. Fass, and G.S. Hamill, Serotonergic and nonserotonergic projections from the rat interpeduncular nucleus to the septum, hippocampal formation and raphe: a combined immunocytochemical and fluorescent retrograde labelling study of neurons in the apical subnucleus. Brain Res Bull, 1988. 20(2): p. 233-40.

**Bed Nucleus of Stria Terminalis**

Intraregional connections

1. Dong, H.W. and L.W. Swanson, Projections from bed nuclei of the stria terminalis, dorsomedial nucleus: implications for cerebral hemisphere integration of neuroendocrine, autonomic, and drinking responses. J Comp Neurol, 2006. 494(1): p. 75-107.

2. Dong, H.W. and L.W. Swanson, Projections from bed nuclei of the stria terminalis, posterior division: implications for cerebral hemisphere regulation of defensive and reproductive behaviors. J Comp Neurol, 2004. 471(4): p. 396-433.

3. Dong, H.W. and L.W. Swanson, Projections from the rhomboid nucleus of the bed nuclei of the stria terminalis: implications for cerebral hemisphere regulation of ingestive behaviors. J Comp Neurol, 2003. 463(4): p. 434-72.

4. Dong, H.-W. and L.W. Swanson, Projections from bed nuclei of the stria terminalis, magnocellular nucleus: Implications for cerebral hemisphere regulation of micturition, defecation, and penile erection. The Journal of Comparative Neurology, 2006. 494(1): p. 108-141.

5. Gu, G., A. Cornea, and R.B. Simerly, Sexual differentiation of projections from the principal nucleus of the bed nuclei of the stria terminalis. J Comp Neurol, 2003. 460(4): p. 542-62.

6. Dong, H.W., et al., Basic organization of projections from the oval and fusiform nuclei of the bed nuclei of the stria terminalis in adult rat brain. J Comp Neurol, 2001. 436(4): p. 430-55.

7. Dong, H., G.D. Petrovich, and L.W. Swanson, Organization of projections from the juxtacapsular nucleus of the BST: a PHAL study in the rat. Brain Res, 2000. 859(1): p. 1-14.

Bed Nucleus of Stria Terminalis - transverse nucleus (efferents and afferents)

1. Dong, H.W. and L.W. Swanson, Projections from bed nuclei of the stria terminalis, posterior division: implications for cerebral hemisphere regulation of defensive and reproductive behaviors. J Comp Neurol, 2004. 471(4): p. 396-433.

2. Luiten, P.G. and P. Room, Interrelations between lateral, dorsomedial and ventromedial hypothalamic nuclei in the rat. An HRP study. Brain Res, 1980. 190(2): p. 321-32.

3. Thompson, R.H. and L.W. Swanson, Organization of inputs to the dorsomedial nucleus of the hypothalamus: a reexamination with Fluorogold and PHAL in the rat. Brain Res Brain Res Rev, 1998. 27(2): p. 89-118.

4. Poulin, J.F., et al., Enkephalinergic afferents of the centromedial amygdala in the rat. J Comp Neurol, 2006. 496(6): p. 859-76.

5. Sim, L.J. and S.A. Joseph, Arcuate nucleus projections to brainstem regions which modulate nociception. J Chem Neuroanat, 1991. 4(2): p. 97-109.

6. Goto, M., et al., Projections from the subfornical region of the lateral hypothalamic area. J Comp Neurol, 2005. 493(3): p. 412-38.

7. Gu, G.B. and R.B. Simerly, Projections of the sexually dimorphic anteroventral periventricular nucleus in the female rat. J Comp Neurol, 1997. 384(1): p. 142-64.

8. Canteras, N.S., R.B. Simerly, and L.W. Swanson, Projections of the ventral premammillary nucleus. J Comp Neurol, 1992. 324(2): p. 195-212.

9. Petrovich, G.D., P.Y. Risold, and L.W. Swanson, Organization of projections from the basomedial nucleus of the amygdala: a PHAL study in the rat. J Comp Neurol, 1996. 374(3): p. 387-420.

10. Canteras, N.S., R.B. Simerly, and L.W. Swanson, Organization of projections from the medial nucleus of the amygdala: a PHAL study in the rat. J Comp Neurol, 1995. 360(2): p. 213-45.

11. Canteras, N.S., R.B. Simerly, and L.W. Swanson, Connections of the posterior nucleus of the amygdala. J Comp Neurol, 1992. 324(2): p. 143-79.

Bed Nucleus of Stria Terminalis - interfascicular nucleus (efferents and afferents)

1. Dong, H.W. and L.W. Swanson, Projections from bed nuclei of the stria terminalis, posterior division: implications for cerebral hemisphere regulation of defensive and reproductive behaviors. J Comp Neurol, 2004. 471(4): p. 396-433.

2. Luiten, P.G. and P. Room, Interrelations between lateral, dorsomedial and ventromedial hypothalamic nuclei in the rat. An HRP study. Brain Res, 1980. 190(2): p. 321-32.

3. Thompson, R.H. and L.W. Swanson, Organization of inputs to the dorsomedial nucleus of the hypothalamus: a reexamination with Fluorogold and PHAL in the rat. Brain Res Brain Res Rev, 1998. 27(2): p. 89-118.

4. Comoli, E., E.R. Ribeiro-Barbosa, and N.S. Canteras, Afferent connections of the dorsal premammillary nucleus. J Comp Neurol, 2000. 423(1): p. 83-98.

5. Poulin, J.F., et al., Enkephalinergic afferents of the centromedial amygdala in the rat. J Comp Neurol, 2006. 496(6): p. 859-76.

6. Canteras, N.S., R.B. Simerly, and L.W. Swanson, Connections of the posterior nucleus of the amygdala. J Comp Neurol, 1992. 324(2): p. 143-79.

7. Risold, P.Y., N.S. Canteras, and L.W. Swanson, Organization of projections from the anterior hypothalamic nucleus: a Phaseolus vulgaris-leucoagglutinin study in the rat. J Comp Neurol, 1994. 348(1): p. 1-40.

8. Sim, L.J. and S.A. Joseph, Arcuate nucleus projections to brainstem regions which modulate nociception. J Chem Neuroanat, 1991. 4(2): p. 97-109.

9. Canteras, N.S., R.B. Simerly, and L.W. Swanson, Projections of the ventral premammillary nucleus. J Comp Neurol, 1992. 324(2): p. 195-212.

10. Gu, G.B. and R.B. Simerly, Projections of the sexually dimorphic anteroventral periventricular nucleus in the female rat. J Comp Neurol, 1997. 384(1): p. 142-64.

11. Goto, M., et al., Projections from the subfornical region of the lateral hypothalamic area. J Comp Neurol, 2005. 493(3): p. 412-38.

12. Canteras, N.S., R.B. Simerly, and L.W. Swanson, Organization of projections from the medial nucleus of the amygdala: a PHAL study in the rat. J Comp Neurol, 1995. 360(2): p. 213-45.

Bed Nucleus of Stria Terminalis – posteromedial (efferents and afferents)

1. Simerly, R.B. and L.W. Swanson, The organization of neural inputs to the medial preoptic nucleus of the rat. J Comp Neurol, 1986. 246(3): p. 312-42.

2. Simerly, R.B. and L.W. Swanson, Projections of the medial preoptic nucleus: a Phaseolus vulgaris leucoagglutinin anterograde tract-tracing study in the rat. J Comp Neurol, 1988. 270(2): p. 209-42.

3. Coolen, L.M., H.J. Peters, and J.G. Veening, Anatomical interrelationships of the medial preoptic area and other brain regions activated following male sexual behavior: a combined fos and tract-tracing study. J Comp Neurol, 1998. 397(3): p. 421-35.

4. Sim, L.J. and S.A. Joseph, Arcuate nucleus projections to brainstem regions which modulate nociception. J Chem Neuroanat, 1991. 4(2): p. 97-109.

5. Sun, N., L. Roberts, and M.D. Cassell, Rat central amygdaloid nucleus projections to the bed nucleus of the stria terminalis. Brain Res Bull, 1991. 27(5): p. 651-62.

6. Poulin, J.F., et al., Enkephalinergic afferents of the centromedial amygdala in the rat. J Comp Neurol, 2006. 496(6): p. 859-76.

7. Prewitt, C.M. and J.P. Herman, Anatomical interactions between the central amygdaloid nucleus and the hypothalamic paraventricular nucleus of the rat: a dual tract-tracing analysis. J Chem Neuroanat, 1998. 15(3): p. 173-85.

8. Bourgeais, L., C. Gauriau, and J.F. Bernard, Projections from the nociceptive area of the central nucleus of the amygdala to the forebrain: a PHA-L study in the rat. Eur J Neurosci, 2001. 14(2): p. 229-55.

9. Weller, K.L. and D.A. Smith, Afferent connections to the bed nucleus of the stria terminalis. Brain Res, 1982. 232(2): p. 255-70.

10. Alden, M., J.M. Besson, and J.F. Bernard, Organization of the efferent projections from the pontine parabrachial area to the bed nucleus of the stria terminalis and neighboring regions: a PHA-L study in the rat. J Comp Neurol, 1994. 341(3): p. 289-314.

Bed Nucleus of Stria Terminalis - principal nucleus (efferents and afferents)

1. Dong, H.W. and L.W. Swanson, Projections from bed nuclei of the stria terminalis, posterior division: implications for cerebral hemisphere regulation of defensive and reproductive behaviors. J Comp Neurol, 2004. 471(4): p. 396-433.

2. Gu, G., A. Cornea, and R.B. Simerly, Sexual differentiation of projections from the principal nucleus of the bed nuclei of the stria terminalis. J Comp Neurol, 2003. 460(4): p. 542-62.

3. Thompson, R.H. and L.W. Swanson, Organization of inputs to the dorsomedial nucleus of the hypothalamus: a reexamination with Fluorogold and PHAL in the rat. Brain Res Brain Res Rev, 1998. 27(2): p. 89-118.

4. Luiten, P.G. and P. Room, Interrelations between lateral, dorsomedial and ventromedial hypothalamic nuclei in the rat. An HRP study. Brain Res, 1980. 190(2): p. 321-32.

5. Abrahamson, E.E. and R.Y. Moore, The posterior hypothalamic area: chemoarchitecture and afferent connections. Brain Res, 2001. 889(1-2): p. 1-22.

6. Canteras, N.S., R.B. Simerly, and L.W. Swanson, Projections of the ventral premammillary nucleus. J Comp Neurol, 1992. 324(2): p. 195-212.

7. Comoli, E., E.R. Ribeiro-Barbosa, and N.S. Canteras, Afferent connections of the dorsal premammillary nucleus. J Comp Neurol, 2000. 423(1): p. 83-98.

8. Hutton, L.A., G. Gu, and R.B. Simerly, Development of a sexually dimorphic projection from the bed nuclei of the stria terminalis to the anteroventral periventricular nucleus in the rat. J Neurosci, 1998. 18(8): p. 3003-13.

9. Polston, E.K., G. Gu, and R.B. Simerly, Neurons in the principal nucleus of the bed nuclei of the stria terminalis provide a sexually dimorphic GABAergic input to the anteroventral periventricular nucleus of the hypothalamus. Neuroscience, 2004. 123(3): p. 793-803.

10. Poulin, J.F., et al., Enkephalinergic afferents of the centromedial amygdala in the rat. J Comp Neurol, 2006. 496(6): p. 859-76.

11. Gu, G.B. and R.B. Simerly, Projections of the sexually dimorphic anteroventral periventricular nucleus in the female rat. J Comp Neurol, 1997. 384(1): p. 142-64.

12. Sim, L.J. and S.A. Joseph, Arcuate nucleus projections to brainstem regions which modulate nociception. J Chem Neuroanat, 1991. 4(2): p. 97-109.

13. Petrovich, G.D., P.Y. Risold, and L.W. Swanson, Organization of projections from the basomedial nucleus of the amygdala: a PHAL study in the rat. J Comp Neurol, 1996. 374(3): p. 387-420.

14. Canteras, N.S., R.B. Simerly, and L.W. Swanson, Connections of the posterior nucleus of the amygdala. J Comp Neurol, 1992. 324(2): p. 143-79.

Bed Nucleus of Stria Terminalis - rhomboid nucleus (efferents and afferents)

1. Dong, H.W. and L.W. Swanson, Projections from the rhomboid nucleus of the bed nuclei of the stria terminalis: implications for cerebral hemisphere regulation of ingestive behaviors. J Comp Neurol, 2003. 463(4): p. 434-72.

2. Poulin, J.F., et al., Enkephalinergic afferents of the centromedial amygdala in the rat. J Comp Neurol, 2006. 496(6): p. 859-76.

3. Sim, L.J. and S.A. Joseph, Arcuate nucleus projections to brainstem regions which modulate nociception. J Chem Neuroanat, 1991. 4(2): p. 97-109.

Bed Nucleus of Stria Terminalis - ventral nucleus (efferents and afferents)

1. McDonald, A.J., et al., Cortical afferents to the extended amygdala. Ann N Y Acad Sci, 1999. 877: p. 309-38.

2. Dong, H.-W. and L.W. Swanson, Projections from bed nuclei of the stria terminalis, magnocellular nucleus: Implications for cerebral hemisphere regulation of micturition, defecation, and penile erection. The Journal of Comparative Neurology, 2006. 494(1): p. 108-141.

3. Dong, H.W. and L.W. Swanson, Projections from bed nuclei of the stria terminalis, anteromedial area: cerebral hemisphere integration of neuroendocrine, autonomic, and behavioral aspects of energy balance. J Comp Neurol, 2006. 494(1): p. 142-78.

4. Thompson, R.H. and L.W. Swanson, Organization of inputs to the dorsomedial nucleus of the hypothalamus: a reexamination with Fluorogold and PHAL in the rat. Brain Res Brain Res Rev, 1998. 27(2): p. 89-118.

5. Luiten, P.G. and P. Room, Interrelations between lateral, dorsomedial and ventromedial hypothalamic nuclei in the rat. An HRP study. Brain Res, 1980. 190(2): p. 321-32.

6. Comoli, E., E.R. Ribeiro-Barbosa, and N.S. Canteras, Afferent connections of the dorsal premammillary nucleus. J Comp Neurol, 2000. 423(1): p. 83-98.

7. Coolen, L.M., et al., Afferent connections of the parvocellular subparafascicular thalamic nucleus in the rat: evidence for functional subdivisions. J Comp Neurol, 2003. 463(2): p. 132-56.

8. Georges, F. and G. Aston-Jones, Activation of ventral tegmental area cells by the bed nucleus of the stria terminalis: a novel excitatory amino acid input to midbrain dopamine neurons. J Neurosci, 2002. 22(12): p. 5173-87.

9. Massi, L., et al., Cannabinoid receptors in the bed nucleus of the stria terminalis control cortical excitation of midbrain dopamine cells in vivo. J Neurosci, 2008. 28(42): p. 10496-508.

10. Gray, T.S. and D.J. Magnuson, Neuropeptide neuronal efferents from the bed nucleus of the stria terminalis and central amygdaloid nucleus to the dorsal vagal complex in the rat. J Comp Neurol, 1987. 262(3): p. 365-74.

11. Finn, P.D. and P. Yahr, Projection from the ventral bed nucleus of the stria terminalis to the retrorubral field in rats and the effects of cells in these areas on mating in male rats versus gerbils. Horm Behav, 2005. 47(2): p. 123-38.

12. ter Horst, G.J. and P.G. Luiten, The projections of the dorsomedial hypothalamic nucleus in the rat. Brain Res Bull, 1986. 16(2): p. 231-48.

13. Gu, G.B. and R.B. Simerly, Projections of the sexually dimorphic anteroventral periventricular nucleus in the female rat. J Comp Neurol, 1997. 384(1): p. 142-64.

14. Sim, L.J. and S.A. Joseph, Arcuate nucleus projections to brainstem regions which modulate nociception. J Chem Neuroanat, 1991. 4(2): p. 97-109.

15. Petrovich, G.D., P.Y. Risold, and L.W. Swanson, Organization of projections from the basomedial nucleus of the amygdala: a PHAL study in the rat. J Comp Neurol, 1996. 374(3): p. 387-420.

16. Rao, Z.R., et al., Origin of leucine-enkephalin fibers and their two main afferent pathways in the bed nucleus of the stria terminalis in the rat. Exp Brain Res, 1987. 65(2): p. 411-20.

17. McDonald, A.J., Projections of the intermediate subdivision of the central amygdaloid nucleus to the bed nucleus of the stria terminalis and medial diencephalon. Neurosci Lett, 1988. 85(3): p. 285-90.

18. Li, S. and G.J. Kirouac, Projections from the paraventricular nucleus of the thalamus to the forebrain, with special emphasis on the extended amygdala. J Comp Neurol, 2008. 506(2): p. 263-87.

Bed Nucleus of Stria Terminalis - anterodorsal area (efferents and afferents)

1. Luiten, P.G. and P. Room, Interrelations between lateral, dorsomedial and ventromedial hypothalamic nuclei in the rat. An HRP study. Brain Res, 1980. 190(2): p. 321-32.

2. Thompson, R.H. and L.W. Swanson, Organization of inputs to the dorsomedial nucleus of the hypothalamus: a reexamination with Fluorogold and PHAL in the rat. Brain Res Brain Res Rev, 1998. 27(2): p. 89-118.

3. Comoli, E., E.R. Ribeiro-Barbosa, and N.S. Canteras, Afferent connections of the dorsal premammillary nucleus. J Comp Neurol, 2000. 423(1): p. 83-98.

4. Canteras, N.S., R.B. Simerly, and L.W. Swanson, Connections of the posterior nucleus of the amygdala. J Comp Neurol, 1992. 324(2): p. 143-79.

5. Canteras, N.S., R.B. Simerly, and L.W. Swanson, Projections of the ventral premammillary nucleus. J Comp Neurol, 1992. 324(2): p. 195-212.

6. Sim, L.J. and S.A. Joseph, Arcuate nucleus projections to brainstem regions which modulate nociception. J Chem Neuroanat, 1991. 4(2): p. 97-109.

Bed Nucleus of Stria Terminalis - anterolateral area (efferents and afferents)

1. Dong, H.W. and L.W. Swanson, Organization of axonal projections from the anterolateral area of the bed nuclei of the stria terminalis. J Comp Neurol, 2004. 468(2): p. 277-98.

2. Sun, N. and M.D. Cassell, Intrinsic GABAergic neurons in the rat central extended amygdala. J Comp Neurol, 1993. 330(3): p. 381-404.

3. Dumont, E.C. and J.T. Williams, Noradrenaline triggers GABAA inhibition of bed nucleus of the stria terminalis neurons projecting to the ventral tegmental area. J Neurosci, 2004. 24(38): p. 8198-204.

4. Moga, M.M., C.B. Saper, and T.S. Gray, Bed nucleus of the stria terminalis: cytoarchitecture, immunohistochemistry, and projection to the parabrachial nucleus in the rat. J Comp Neurol, 1989. 283(3): p. 315-32.

5. Moga, M.M., et al., Organization of cortical, basal forebrain, and hypothalamic afferents to the parabrachial nucleus in the rat. J Comp Neurol, 1990. 295(4): p. 624-61.

6. Gray, T.S. and D.J. Magnuson, Peptide immunoreactive neurons in the amygdala and the bed nucleus of the stria terminalis project to the midbrain central gray in the rat. Peptides, 1992. 13(3): p. 451-60.

7. Weller, K.L. and D.A. Smith, Afferent connections to the bed nucleus of the stria terminalis. Brain Res, 1982. 232(2): p. 255-70.

8. Sun, N., L. Roberts, and M.D. Cassell, Rat central amygdaloid nucleus projections to the bed nucleus of the stria terminalis. Brain Res Bull, 1991. 27(5): p. 651-62.

9. Poulin, J.F., et al., Enkephalinergic afferents of the centromedial amygdala in the rat. J Comp Neurol, 2006. 496(6): p. 859-76.

10. Prewitt, C.M. and J.P. Herman, Anatomical interactions between the central amygdaloid nucleus and the hypothalamic paraventricular nucleus of the rat: a dual tract-tracing analysis. J Chem Neuroanat, 1998. 15(3): p. 173-85.

11. Bourgeais, L., C. Gauriau, and J.F. Bernard, Projections from the nociceptive area of the central nucleus of the amygdala to the forebrain: a PHA-L study in the rat. Eur J Neurosci, 2001. 14(2): p. 229-55.

12. Moga, M.M., R.P. Weis, and R.Y. Moore, Efferent projections of the paraventricular thalamic nucleus in the rat. J Comp Neurol, 1995. 359(2): p. 221-38.

13. Li, S. and G.J. Kirouac, Projections from the paraventricular nucleus of the thalamus to the forebrain, with special emphasis on the extended amygdala. J Comp Neurol, 2008. 506(2): p. 263-87.

14. Alden, M., J.M. Besson, and J.F. Bernard, Organization of the efferent projections from the pontine parabrachial area to the bed nucleus of the stria terminalis and neighboring regions: a PHA-L study in the rat. J Comp Neurol, 1994. 341(3): p. 289-314.

15. Kozicz, T. and A. Arimura, Axon terminals containing CGRP-immunoreactivity form synapses with CRF- and Met-enkephalin-immunopositive neurons in the laterodorsal division of the bed nucleus of the stria terminalis in the rat. Brain Res, 2001. 893(1-2): p. 11-20.

Bed Nucleus of the Stria Terminalis- anteromedial subnucleus (efferents and afferents)

1. Bokor, H., et al., Cellular architecture of the nucleus reuniens thalami and its putative aspartatergic/glutamatergic projection to the hippocampus and medial septum in the rat. Eur J Neurosci, 2002. 16(7): p. 1227-39.

2. Dong, H.W. and L.W. Swanson, Projections from bed nuclei of the stria terminalis, anteromedial area: cerebral hemisphere integration of neuroendocrine, autonomic, and behavioral aspects of energy balance. J Comp Neurol, 2006. 494(1): p. 142-78.

3. Sim, L.J. and S.A. Joseph, Arcuate nucleus projections to brainstem regions which modulate nociception. J Chem Neuroanat, 1991. 4(2): p. 97-109.

4. Sun, N., L. Roberts, and M.D. Cassell, Rat central amygdaloid nucleus projections to the bed nucleus of the stria terminalis. Brain Res Bull, 1991. 27(5): p. 651-62.

5. Prewitt, C.M. and J.P. Herman, Anatomical interactions between the central amygdaloid nucleus and the hypothalamic paraventricular nucleus of the rat: a dual tract-tracing analysis. J Chem Neuroanat, 1998. 15(3): p. 173-85.

6. Weller, K.L. and D.A. Smith, Afferent connections to the bed nucleus of the stria terminalis. Brain Res, 1982. 232(2): p. 255-70.

7. Poulin, J.F., et al., Enkephalinergic afferents of the centromedial amygdala in the rat. J Comp Neurol, 2006. 496(6): p. 859-76.

8. Bourgeais, L., C. Gauriau, and J.F. Bernard, Projections from the nociceptive area of the central nucleus of the amygdala to the forebrain: a PHA-L study in the rat. Eur J Neurosci, 2001. 14(2): p. 229-55.

9. McDonald, A.J., et al., Cortical afferents to the extended amygdala. Ann N Y Acad Sci, 1999. 877: p. 309-38.

10. Moga, M.M., R.P. Weis, and R.Y. Moore, Efferent projections of the paraventricular thalamic nucleus in the rat. J Comp Neurol, 1995. 359(2): p. 221-38.

11. Alden, M., J.M. Besson, and J.F. Bernard, Organization of the efferent projections from the pontine parabrachial area to the bed nucleus of the stria terminalis and neighboring regions: a PHA-L study in the rat. J Comp Neurol, 1994. 341(3): p. 289-314.

Bed Nucleus of the Stria Terminalis – anteroventral (efferents and afferents)

1. Luiten, P.G. and P. Room, Interrelations between lateral, dorsomedial and ventromedial hypothalamic nuclei in the rat. An HRP study. Brain Res, 1980. 190(2): p. 321-32.

2. Thompson, R.H. and L.W. Swanson, Organization of inputs to the dorsomedial nucleus of the hypothalamus: a reexamination with Fluorogold and PHAL in the rat. Brain Res Brain Res Rev, 1998. 27(2): p. 89-118.

3. Canteras, N.S., R.B. Simerly, and L.W. Swanson, Projections of the ventral premammillary nucleus. J Comp Neurol, 1992. 324(2): p. 195-212.

4. Sim, L.J. and S.A. Joseph, Arcuate nucleus projections to brainstem regions which modulate nociception. J Chem Neuroanat, 1991. 4(2): p. 97-109.

Bed Nucleus of the Stria Terminalis - dorsolateral nucleus (efferents and afferents)

1. Moga, M.M., et al., Organization of cortical, basal forebrain, and hypothalamic afferents to the parabrachial nucleus in the rat. J Comp Neurol, 1990. 295(4): p. 624-61.

2. Moga, M.M., C.B. Saper, and T.S. Gray, Bed nucleus of the stria terminalis: cytoarchitecture, immunohistochemistry, and projection to the parabrachial nucleus in the rat. J Comp Neurol, 1989. 283(3): p. 315-32.

3. Yasui, Y., et al., Autonomic responses and efferent pathways from the insular cortex in the rat. J Comp Neurol, 1991. 303(3): p. 355-74.

4. McDonald, A.J., et al., Cortical afferents to the extended amygdala. Ann N Y Acad Sci, 1999. 877: p. 309-38.

5. Sim, L.J. and S.A. Joseph, Arcuate nucleus projections to brainstem regions which modulate nociception. J Chem Neuroanat, 1991. 4(2): p. 97-109.

6. Prewitt, C.M. and J.P. Herman, Anatomical interactions between the central amygdaloid nucleus and the hypothalamic paraventricular nucleus of the rat: a dual tract-tracing analysis. J Chem Neuroanat, 1998. 15(3): p. 173-85.

7. Moga, M.M., R.P. Weis, and R.Y. Moore, Efferent projections of the paraventricular thalamic nucleus in the rat. J Comp Neurol, 1995. 359(2): p. 221-38.

8. Li, S. and G.J. Kirouac, Projections from the paraventricular nucleus of the thalamus to the forebrain, with special emphasis on the extended amygdala. J Comp Neurol, 2008. 506(2): p. 263-87.

9. Alden, M., J.M. Besson, and J.F. Bernard, Organization of the efferent projections from the pontine parabrachial area to the bed nucleus of the stria terminalis and neighboring regions: a PHA-L study in the rat. J Comp Neurol, 1994. 341(3): p. 289-314.

Bed Nucleus of the Stria Terminalis - dorsomedial nucleus (efferents and afferents)

1. Dong, H.W. and L.W. Swanson, Projections from bed nuclei of the stria terminalis, dorsomedial nucleus: implications for cerebral hemisphere integration of neuroendocrine, autonomic, and drinking responses. J Comp Neurol, 2006. 494(1): p. 75-107.

2. Dong, H.-W. and L.W. Swanson, Projections from bed nuclei of the stria terminalis, magnocellular nucleus: Implications for cerebral hemisphere regulation of micturition, defecation, and penile erection. The Journal of Comparative Neurology, 2006. 494(1): p. 108-141.

3. Dong, H.W. and L.W. Swanson, Projections from bed nuclei of the stria terminalis, anteromedial area: cerebral hemisphere integration of neuroendocrine, autonomic, and behavioral aspects of energy balance. J Comp Neurol, 2006. 494(1): p. 142-78.

4. Thompson, R.H. and L.W. Swanson, Organization of inputs to the dorsomedial nucleus of the hypothalamus: a reexamination with Fluorogold and PHAL in the rat. Brain Res Brain Res Rev, 1998. 27(2): p. 89-118.

5. Luiten, P.G. and P. Room, Interrelations between lateral, dorsomedial and ventromedial hypothalamic nuclei in the rat. An HRP study. Brain Res, 1980. 190(2): p. 321-32.

6. Canteras, N.S., R.B. Simerly, and L.W. Swanson, Connections of the posterior nucleus of the amygdala. J Comp Neurol, 1992. 324(2): p. 143-79.

7. Sim, L.J. and S.A. Joseph, Arcuate nucleus projections to brainstem regions which modulate nociception. J Chem Neuroanat, 1991. 4(2): p. 97-109.

Bed Nucleus of the Stria Terminalis- fusiform nucleus (efferents and afferents)

1. Dong, H.W., et al., Basic organization of projections from the oval and fusiform nuclei of the bed nuclei of the stria terminalis in adult rat brain. J Comp Neurol, 2001. 436(4): p. 430-55.

2. Dong, H.W. and L.W. Swanson, Projections from bed nuclei of the stria terminalis, anteromedial area: cerebral hemisphere integration of neuroendocrine, autonomic, and behavioral aspects of energy balance. J Comp Neurol, 2006. 494(1): p. 142-78.

3. Poulin, J.F., et al., Enkephalinergic afferents of the centromedial amygdala in the rat. J Comp Neurol, 2006. 496(6): p. 859-76.

4. Sim, L.J. and S.A. Joseph, Arcuate nucleus projections to brainstem regions which modulate nociception. J Chem Neuroanat, 1991. 4(2): p. 97-109.

5. Li, S. and G.J. Kirouac, Projections from the paraventricular nucleus of the thalamus to the forebrain, with special emphasis on the extended amygdala. J Comp Neurol, 2008. 506(2): p. 263-87.

Bed Nucleus of the Stria Terminalis- juxtacapsular nucleus (efferents and afferents)

1. Dong, H., G.D. Petrovich, and L.W. Swanson, Organization of projections from the juxtacapsular nucleus of the BST: a PHAL study in the rat. Brain Res, 2000. 859(1): p. 1-14.

2. Poulin, J.F., et al., Enkephalinergic afferents of the centromedial amygdala in the rat. J Comp Neurol, 2006. 496(6): p. 859-76.

3. McDonald, A.J., et al., Cortical afferents to the extended amygdala. Ann N Y Acad Sci, 1999. 877: p. 309-38.

4. Massi, L., et al., Cannabinoid receptors in the bed nucleus of the stria terminalis control cortical excitation of midbrain dopamine cells in vivo. J Neurosci, 2008. 28(42): p. 10496-508.

5. Sim, L.J. and S.A. Joseph, Arcuate nucleus projections to brainstem regions which modulate nociception. J Chem Neuroanat, 1991. 4(2): p. 97-109.

6. Petrovich, G.D., P.Y. Risold, and L.W. Swanson, Organization of projections from the basomedial nucleus of the amygdala: a PHAL study in the rat. J Comp Neurol, 1996. 374(3): p. 387-420.

7. Li, S. and G.J. Kirouac, Projections from the paraventricular nucleus of the thalamus to the forebrain, with special emphasis on the extended amygdala. J Comp Neurol, 2008. 506(2): p. 263-87.

Bed Nucleus of the Stria Terminalis - magnocellular nucleus (efferents and afferents)

1. Dong, H.-W. and L.W. Swanson, Projections from bed nuclei of the stria terminalis, magnocellular nucleus: Implications for cerebral hemisphere regulation of micturition, defecation, and penile erection. The Journal of Comparative Neurology, 2006. 494(1): p. 108-141.

2. Dong, H.W. and L.W. Swanson, Projections from bed nuclei of the stria terminalis, anteromedial area: cerebral hemisphere integration of neuroendocrine, autonomic, and behavioral aspects of energy balance. J Comp Neurol, 2006. 494(1): p. 142-78.

3. Finn, P.D. and P. Yahr, Projection from the ventral bed nucleus of the stria terminalis to the retrorubral field in rats and the effects of cells in these areas on mating in male rats versus gerbils. Horm Behav, 2005. 47(2): p. 123-38.

4. Sim, L.J. and S.A. Joseph, Arcuate nucleus projections to brainstem regions which modulate nociception. J Chem Neuroanat, 1991. 4(2): p. 97-109.

5. Petrovich, G.D., P.Y. Risold, and L.W. Swanson, Organization of projections from the basomedial nucleus of the amygdala: a PHAL study in the rat. J Comp Neurol, 1996. 374(3): p. 387-420.

Bed Nucleus of the Stria Terminalis - oval nucleus (efferents and afferents)

1. Dong, H.W., et al., Basic organization of projections from the oval and fusiform nuclei of the bed nuclei of the stria terminalis in adult rat brain. J Comp Neurol, 2001. 436(4): p. 430-55.

2. Poulin, J.F., et al., Enkephalinergic afferents of the centromedial amygdala in the rat. J Comp Neurol, 2006. 496(6): p. 859-76.

3. Geisler, S. and D.S. Zahm, Afferents of the ventral tegmental area in the rat-anatomical substratum for integrative functions. J Comp Neurol, 2005. 490(3): p. 270-94.

4. Sim, L.J. and S.A. Joseph, Arcuate nucleus projections to brainstem regions which modulate nociception. J Chem Neuroanat, 1991. 4(2): p. 97-109.

5. Petrovich, G.D., P.Y. Risold, and L.W. Swanson, Organization of projections from the basomedial nucleus of the amygdala: a PHAL study in the rat. J Comp Neurol, 1996. 374(3): p. 387-420.

Bed Nucleus of the Stria Terminalis - posterolateral nucleus (efferents and afferents)

1. Moga, M.M., et al., Organization of cortical, basal forebrain, and hypothalamic afferents to the parabrachial nucleus in the rat. J Comp Neurol, 1990. 295(4): p. 624-61.

2. Moga, M.M., C.B. Saper, and T.S. Gray, Bed nucleus of the stria terminalis: cytoarchitecture, immunohistochemistry, and projection to the parabrachial nucleus in the rat. J Comp Neurol, 1989. 283(3): p. 315-32.

3. Finn, P.D. and P. Yahr, Projection from the ventral bed nucleus of the stria terminalis to the retrorubral field in rats and the effects of cells in these areas on mating in male rats versus gerbils. Horm Behav, 2005. 47(2): p. 123-38.

4. McDonald, A.J., et al., Cortical afferents to the extended amygdala. Ann N Y Acad Sci, 1999. 877: p. 309-38.

5. Massi, L., et al., Cannabinoid receptors in the bed nucleus of the stria terminalis control cortical excitation of midbrain dopamine cells in vivo. J Neurosci, 2008. 28(42): p. 10496-508.

6. Sim, L.J. and S.A. Joseph, Arcuate nucleus projections to brainstem regions which modulate nociception. J Chem Neuroanat, 1991. 4(2): p. 97-109.

7. Weller, K.L. and D.A. Smith, Afferent connections to the bed nucleus of the stria terminalis. Brain Res, 1982. 232(2): p. 255-70.

8. Sun, N., L. Roberts, and M.D. Cassell, Rat central amygdaloid nucleus projections to the bed nucleus of the stria terminalis. Brain Res Bull, 1991. 27(5): p. 651-62.

9. Bourgeais, L., C. Gauriau, and J.F. Bernard, Projections from the nociceptive area of the central nucleus of the amygdala to the forebrain: a PHA-L study in the rat. Eur J Neurosci, 2001. 14(2): p. 229-55.

10. Prewitt, C.M. and J.P. Herman, Anatomical interactions between the central amygdaloid nucleus and the hypothalamic paraventricular nucleus of the rat: a dual tract-tracing analysis. J Chem Neuroanat, 1998. 15(3): p. 173-85.

11. Poulin, J.F., et al., Enkephalinergic afferents of the centromedial amygdala in the rat. J Comp Neurol, 2006. 496(6): p. 859-76.

12. McDonald, A.J., Somatostatinergic projections from the amygdala to the bed nucleus of the stria terminalis and medial preoptic-hypothalamic region. Neurosci Lett, 1987. 75(3): p. 271-7.

13. Alden, M., J.M. Besson, and J.F. Bernard, Organization of the efferent projections from the pontine parabrachial area to the bed nucleus of the stria terminalis and neighboring regions: a PHA-L study in the rat. J Comp Neurol, 1994. 341(3): p. 289-314.

14. Kozicz, T. and A. Arimura, Axon terminals containing CGRP-immunoreactivity form synapses with CRF- and Met-enkephalin-immunopositive neurons in the laterodorsal division of the bed nucleus of the stria terminalis in the rat. Brain Res, 2001. 893(1-2): p. 11-20.

Bed Nucleus of the Stria Terminalis – supracapsular nucleus (efferents and afferents)

1. Shammah-Lagnado, S.J., et al., Supracapsular bed nucleus of the stria terminalis contains central and medial extended amygdala elements: evidence from anterograde and retrograde tracing experiments in the rat. J Comp Neurol, 2000. 422(4): p. 533-55.

2. Moga, M.M., C.B. Saper, and T.S. Gray, Bed nucleus of the stria terminalis: cytoarchitecture, immunohistochemistry, and projection to the parabrachial nucleus in the rat. J Comp Neurol, 1989. 283(3): p. 315-32.

3. Sim, L.J. and S.A. Joseph, Arcuate nucleus projections to brainstem regions which modulate nociception. J Chem Neuroanat, 1991. 4(2): p. 97-109.

Bed Nucleus of the Stria Terminalis - ventrolateral nucleus (efferents and afferents)

1. Dumont, E.C. and J.T. Williams, Noradrenaline triggers GABAA inhibition of bed nucleus of the stria terminalis neurons projecting to the ventral tegmental area. J Neurosci, 2004. 24(38): p. 8198-204.

2. Moga, M.M., C.B. Saper, and T.S. Gray, Bed nucleus of the stria terminalis: cytoarchitecture, immunohistochemistry, and projection to the parabrachial nucleus in the rat. J Comp Neurol, 1989. 283(3): p. 315-32.

3. Moga, M.M., et al., Organization of cortical, basal forebrain, and hypothalamic afferents to the parabrachial nucleus in the rat. J Comp Neurol, 1990. 295(4): p. 624-61.

4. Gray, T.S. and D.J. Magnuson, Peptide immunoreactive neurons in the amygdala and the bed nucleus of the stria terminalis project to the midbrain central gray in the rat. Peptides, 1992. 13(3): p. 451-60.

5. Shin, J.W., J.C. Geerling, and A.D. Loewy, Inputs to the ventrolateral bed nucleus of the stria terminalis. J Comp Neurol, 2008. 511(5): p. 628-57.

6. McDonald, A.J., et al., Cortical afferents to the extended amygdala. Ann N Y Acad Sci, 1999. 877: p. 309-38.

7. Sim, L.J. and S.A. Joseph, Arcuate nucleus projections to brainstem regions which modulate nociception. J Chem Neuroanat, 1991. 4(2): p. 97-109.

8. Prewitt, C.M. and J.P. Herman, Anatomical interactions between the central amygdaloid nucleus and the hypothalamic paraventricular nucleus of the rat: a dual tract-tracing analysis. J Chem Neuroanat, 1998. 15(3): p. 173-85.

9. Bourgeais, L., C. Gauriau, and J.F. Bernard, Projections from the nociceptive area of the central nucleus of the amygdala to the forebrain: a PHA-L study in the rat. Eur J Neurosci, 2001. 14(2): p. 229-55.

10. Alden, M., J.M. Besson, and J.F. Bernard, Organization of the efferent projections from the pontine parabrachial area to the bed nucleus of the stria terminalis and neighboring regions: a PHA-L study in the rat. J Comp Neurol, 1994. 341(3): p. 289-314.

11. Kozicz, T. and A. Arimura, Axon terminals containing CGRP-immunoreactivity form synapses with CRF- and Met-enkephalin-immunopositive neurons in the laterodorsal division of the bed nucleus of the stria terminalis in the rat. Brain Res, 2001. 893(1-2): p. 11-20.

Bed Nucleus of the Stria Terminalis - ventromedial nucleus (efferents and afferents)

1. Moga, M.M., C.B. Saper, and T.S. Gray, Bed nucleus of the stria terminalis: cytoarchitecture, immunohistochemistry, and projection to the parabrachial nucleus in the rat. J Comp Neurol, 1989. 283(3): p. 315-32.

2. Sim, L.J. and S.A. Joseph, Arcuate nucleus projections to brainstem regions which modulate nociception. J Chem Neuroanat, 1991. 4(2): p. 97-109.

3. Bourgeais, L., C. Gauriau, and J.F. Bernard, Projections from the nociceptive area of the central nucleus of the amygdala to the forebrain: a PHA-L study in the rat. Eur J Neurosci, 2001. 14(2): p. 229-55.

4. Prewitt, C.M. and J.P. Herman, Anatomical interactions between the central amygdaloid nucleus and the hypothalamic paraventricular nucleus of the rat: a dual tract-tracing analysis. J Chem Neuroanat, 1998. 15(3): p. 173-85.

5. Alden, M., J.M. Besson, and J.F. Bernard, Organization of the efferent projections from the pontine parabrachial area to the bed nucleus of the stria terminalis and neighboring regions: a PHA-L study in the rat. J Comp Neurol, 1994. 341(3): p. 289-314.

Bed Nucleus of the Stria Terminalis - preoptic nucleus (efferents and afferents)

1. Moga, M.M., et al., Organization of cortical, basal forebrain, and hypothalamic afferents to the parabrachial nucleus in the rat. J Comp Neurol, 1990. 295(4): p. 624-61.

2. Moga, M.M., C.B. Saper, and T.S. Gray, Bed nucleus of the stria terminalis: cytoarchitecture, immunohistochemistry, and projection to the parabrachial nucleus in the rat. J Comp Neurol, 1989. 283(3): p. 315-32.

3. Sim, L.J. and S.A. Joseph, Arcuate nucleus projections to brainstem regions which modulate nociception. J Chem Neuroanat, 1991. 4(2): p. 97-109.

**Globus Pallidus**

Intraregional connections

1. Fink-Jensen, A. and J.D. Mikkelsen, A direct neuronal projection from the entopeduncular nucleus to the globus pallidus. A PHA-L anterograde tracing study in the rat. Brain Res, 1991. 542(1): p. 175-9.

2. Kincaid, A.E., et al., Evidence for a projection from the globus pallidus to the entopeduncular nucleus in the rat. Neurosci Lett, 1991. 128(1): p. 121-5.

3. Smith, Y. and J.P. Bolam, Neurons of the substantia nigra reticulata receive a dense GABA-containing input from the globus pallidus in the rat. Brain Res, 1989. 493(1): p. 160-7.

4. Smith, Y., J.P. Bolam, and M. Von Krosigk, Topographical and Synaptic Organization of the GABA-Containing Pallidosubthalamic Projection in the Rat. Eur J Neurosci, 1990. 2(6): p. 500-511.

5. Bevan, M.D., N.P. Clarke, and J.P. Bolam, Synaptic integration of functionally diverse pallidal information in the entopeduncular nucleus and subthalamic nucleus in the rat. J Neurosci, 1997. 17(1): p. 308-24.

6. Bolam, J.P. and Y. Smith, The striatum and the globus pallidus send convergent synaptic inputs onto single cells in the entopeduncular nucleus of the rat: a double anterograde labelling study combined with postembedding immunocytochemistry for GABA. J Comp Neurol, 1992. 321(3): p. 456-76.

Globus pallidus (efferents and afferents)

1. Nakashima, M., et al., An anterograde and retrograde tract-tracing study on the projections from the thalamic gustatory area in the rat: distribution of neurons projecting to the insular cortex and amygdaloid complex. Neurosci Res, 2000. 36(4): p. 297-309.

2. Bevan, M.D., N.P. Clarke, and J.P. Bolam, Synaptic integration of functionally diverse pallidal information in the entopeduncular nucleus and subthalamic nucleus in the rat. J Neurosci, 1997. 17(1): p. 308-24.

3. Brog, J.S., et al., The patterns of afferent innervation of the core and shell in the "accumbens" part of the rat ventral striatum: immunohistochemical detection of retrogradely transported fluoro-gold. J Comp Neurol, 1993. 338(2): p. 255-78.

4. Bevan, M.D., et al., Selective innervation of neostriatal interneurons by a subclass of neuron in the globus pallidus of the rat. J Neurosci, 1998. 18(22): p. 9438-52.

5. Kincaid, A.E., et al., Evidence for a projection from the globus pallidus to the entopeduncular nucleus in the rat. Neurosci Lett, 1991. 128(1): p. 121-5.

6. Staines, W.A. and H.C. Fibiger, Collateral projections of neurons of the rat globus pallidus to the striatum and substantia nigra. Exp Brain Res, 1984. 56(2): p. 217-20.

7. Hattori, T., H.C. Fibiger, and P.L. McGeer, Demonstration of a pallido-nigral projection innervating dopaminergic neurons. J Comp Neurol, 1975. 162(4): p. 487-504.

8. Shammah-Lagnado, S.J., G.F. Alheid, and L. Heimer, Efferent connections of the caudal part of the globus pallidus in the rat. J Comp Neurol, 1996. 376(3): p. 489-507.

9. Ottersen, O.P., Afferent connections to the amygdaloid complex of the rat and cat: II. Afferents from the hypothalamus and the basal telencephalon. J Comp Neurol, 1980. 194(1): p. 267-89.

10. Mogenson, G.J., et al., Ventral pallidum projections to mediodorsal nucleus of the thalamus: an anatomical and electrophysiological investigation in the rat. Brain Res, 1987. 404(1-2): p. 221-30.

11. Groenewegen, H.J., Organization of the afferent connections of the mediodorsal thalamic nucleus in the rat, related to the mediodorsal-prefrontal topography. Neuroscience, 1988. 24(2): p. 379-431.

12. Ray, J.P., et al., Sources of presumptive glutamatergic/aspartatergic afferents to the mediodorsal nucleus of the thalamus in the rat. J Comp Neurol, 1992. 320(4): p. 435-56.

13. Ray, J.P. and J.L. Price, The organization of the thalamocortical connections of the mediodorsal thalamic nucleus in the rat, related to the ventral forebrain-prefrontal cortex topography. J Comp Neurol, 1992. 323(2): p. 167-97.

14. Hallanger, A.E., et al., The origins of cholinergic and other subcortical afferents to the thalamus in the rat. J Comp Neurol, 1987. 262(1): p. 105-24.

15. Churchill, L., D.S. Zahm, and P.W. Kalivas, The mediodorsal nucleus of the thalamus in rats--I. forebrain gabaergic innervation. Neuroscience, 1996. 70(1): p. 93-102.

16. Sugimoto, T. and T. Hattori, Direct projections from the globus pallidus to the paraventricular nucleus of the thalamus in the rat. Brain Res, 1984. 323(1): p. 188-92.

17. Smith, Y., J.P. Bolam, and M. Von Krosigk, Topographical and Synaptic Organization of the GABA-Containing Pallidosubthalamic Projection in the Rat. Eur J Neurosci, 1990. 2(6): p. 500-511.

18. Smith, Y., J.P. Bolam, and M. Von Krosigk, Topographical and Synaptic Organization of the GABA-Containing Pallidosubthalamic Projection in the Rat. Eur J Neurosci, 1990. 2(6): p. 500-511..

19. Govindaiah, G., et al., Regulation of inhibitory synapses by presynaptic D(4) dopamine receptors in thalamus. J Neurophysiol, 2010. 104(5): p. 2757-65.

20. Gasca-Martinez, D., et al., Dopamine inhibits GABA transmission from the globus pallidus to the thalamic reticular nucleus via presynaptic D4 receptors. Neuroscience, 2010. 169(4): p. 1672-81.

21. Coolen, L.M., et al., Afferent connections of the parvocellular subparafascicular thalamic nucleus in the rat: evidence for functional subdivisions. J Comp Neurol, 2003. 463(2): p. 132-56.

22. Herkenham, M., The afferent and efferent connections of the ventromedial thalamic nucleus in the rat. J Comp Neurol, 1979. 183(3): p. 487-517.

23. Kita, H. and S.T. Kitai, The morphology of globus pallidus projection neurons in the rat: an intracellular staining study. Brain Res, 1994. 636(2): p. 308-19.

24. van der Kooy, D., et al., The pallido-subthalamic projection in rat: anatomical and biochemical studies. Brain Res, 1981. 204(2): p. 253-68.

25. Cooper, A.J. and I.M. Stanford, Calbindin D-28k positive projection neurones and calretinin positive interneurones of the rat globus pallidus. Brain Res, 2002. 929(2): p. 243-51.

26. Smith, Y. and J.P. Bolam, Neurons of the substantia nigra reticulata receive a dense GABA-containing input from the globus pallidus in the rat. Brain Res, 1989. 493(1): p. 160-7.

27. Bell, K., L. Churchill, and P.W. Kalivas, GABAergic projection from the ventral pallidum and globus pallidus to the subthalamic nucleus. Synapse, 1995. 20(1): p. 10-8.

28. Haber, S.N., et al., Efferent connections of the ventral pallidum: evidence of a dual striato pallidofugal pathway. J Comp Neurol, 1985. 235(3): p. 322-35.

29. Schmued, L., et al., Collateralization and GAD immunoreactivity of descending pallidal efferents. Brain Res, 1989. 487(1): p. 131-42.

30. Carter, D.A. and H.C. Fibiger, The projections of the entopeduncular nucleus and globus pallidus in rat as demonstrated by autoradiography and horseradish peroxidase histochemistry. J Comp Neurol, 1978. 177(1): p. 113-23.

31. Moriizumi, T. and T. Hattori, Separate neuronal populations of the rat globus pallidus projecting to the subthalamic nucleus, auditory cortex and pedunculopontine tegmental area. Neuroscience, 1992. 46(3): p. 701-10.

32. Hassani, O.K., et al., Evidence for a dopaminergic innervation of the subthalamic nucleus in the rat. Brain Res, 1997. 749(1): p. 88-94.

33. Canteras, N.S., et al., Afferent connections of the subthalamic nucleus: a combined retrograde and anterograde horseradish peroxidase study in the rat. Brain Res, 1990. 513(1): p. 43-59.

34. Jessell, T.M., et al., Topographic projections of substance P and GABA pathways in the striato- and pallido-nigral system: a biochemical and immunohistochemical study. Brain Res, 1978. 152(3): p. 487-98.

35. Smith, Y. and J.P. Bolam, The output neurones and the dopaminergic neurones of the substantia nigra receive a GABA-containing input from the globus pallidus in the rat. J Comp Neurol, 1990. 296(1): p. 47-64.

36. Araki, M., P.L. McGeer, and E.G. McGeer, Striatonigral and pallidonigral pathways studied by a combination of retrograde horseradish peroxidase tracing and a pharmacohistochemical method for gamma-aminobutyric acid transaminase. Brain Res, 1985. 331(1): p. 17-24.

37. Bunney, B.S. and G.K. Aghajanian, The precise localization of nigral afferents in the rat as determined by a retrograde tracing technique. Brain Res, 1976. 117(3): p. 423-35.

38. Bunney, B.S. and G.K. Aghajanian, The precise localization of nigral afferents in the rat as determined by a retrograde tracing technique. Brain Res, 1976. 117(3): p. 423-35.

39. Edstrom, J.P. and J.W. Phillis, A cholinergic projection from the globus pallidus to cerebral cortex. Brain Res, 1980. 189(2): p. 524-9.

40. Moriizumi, T. and T. Hattori, Pallidotectal projection to the inferior colliculus of the rat. Exp Brain Res, 1991. 87(1): p. 223-6.

41. Staines, W.A., S. Atmadja, and H.C. Fibiger, Demonstration of a pallidostriatal pathway by retrograde transport of HRP-labeled lectin. Brain Res, 1981. 206(2): p. 446-50.

42. Kita, H. and T. Kita, Number, origins, and chemical types of rat pallidostriatal projection neurons. J Comp Neurol, 2001. 437(4): p. 438-48.

43. Rajakumar, N., K. Elisevich, and B.A. Flumerfelt, The pallidostriatal projection in the rat: a recurrent inhibitory loop? Brain Res, 1994. 651(1-2): p. 332-6.

44. Walker, R.H., G.W. Arbuthnott, and A.K. Wright, Electrophysiological and anatomical observations concerning the pallidostriatal pathway in the rat. Exp Brain Res, 1989. 74(2): p. 303-10.

45. Gaykema, R.P., et al., Prefrontal cortical projections to the cholinergic neurons in the basal forebrain. J Comp Neurol, 1991. 303(4): p. 563-83.

46. Heimer, L., et al., Specificity in the projection patterns of accumbal core and shell in the rat. Neuroscience, 1991. 41(1): p. 89-125.

47. Conrad, L.C. and D.W. Pfaff, Autoradiographic tracing of nucleus accumbens efferents in the rat. Brain Res, 1976. 113(3): p. 589-96.

48. Usuda, I., K. Tanaka, and T. Chiba, Efferent projections of the nucleus accumbens in the rat with special reference to subdivision of the nucleus: biotinylated dextran amine study. Brain Res, 1998. 797(1): p. 73-93.

49. Jones, D.L. and G.J. Mogenson, Nucleus accumbens to globus pallidus GABA projection subserving ambulatory activity. Am J Physiol, 1980. 238(1): p. R65-9.

50. Mogenson, G.J., L.W. Swanson, and M. Wu, Neural projections from nucleus accumbens to globus pallidus, substantia innominata, and lateral preoptic-lateral hypothalamic area: an anatomical and electrophysiological investigation in the rat. J Neurosci, 1983. 3(1): p. 189-202.

51. Dray, A. and N.R. Oakley, Projections from nucleus accumbens to globus pallidus and substantia nigra in the rat. Experientia, 1978. 34(1): p. 68-70.

52. Williams, D.J., A.R. Crossman, and P. Slater, The efferent projections of the nucleus accumbens in the rat. Brain Res, 1977. 130(2): p. 217-27.

53. Zahm, D.S. and L. Heimer, Specificity in the efferent projections of the nucleus accumbens in the rat: comparison of the rostral pole projection patterns with those of the core and shell. J Comp Neurol, 1993. 327(2): p. 220-32.

54. Del Fiacco, M., G. Paxinos, and A.C. Cuello, Neostriatal enkephalin-immunoreactive neurones project to the globus pallidus. Brain Res, 1982. 231(1): p. 1-17.

55. Kita, H., Parvalbumin-immunopositive neurons in rat globus pallidus: a light and electron microscopic study. Brain Res, 1994. 657(1-2): p. 31-41.

56. Chang, H.T., C.J. Wilson, and S.T. Kitai, Single neostriatal efferent axons in the globus pallidus: a light and electron microscopic study. Science, 1981. 213(4510): p. 915-8.

57. Kincaid, A.E., et al., The globus pallidus receives a projection from the parafascicular nucleus in the rat. Brain Res, 1991. 553(1): p. 18-26.

58. Wu, Y., S. Richard, and A. Parent, The organization of the striatal output system: a single-cell juxtacellular labeling study in the rat. Neurosci Res, 2000. 38(1): p. 49-62.

59. Brann, M.R. and P.C. Emson, Microiontophoretic injection of fluorescent tracer combined with simultaneous immunofluorescent histochemistry for the demonstration of efferents from the caudate-putamen projecting to the globus pallidus. Neurosci Lett, 1980. 16(1): p. 61-5.

60. Tallaksen-Greene, S.J. and R.L. Albin, Localization of AMPA-selective excitatory amino acid receptor subunits in identified populations of striatal neurons. Neuroscience, 1994. 61(3): p. 509-19.

61. Bourgeais, L., C. Gauriau, and J.F. Bernard, Projections from the nociceptive area of the central nucleus of the amygdala to the forebrain: a PHA-L study in the rat. Eur J Neurosci, 2001. 14(2): p. 229-55.

62. Kohler, C., et al., The cytoarchitecture, histochemistry and projections of the tuberomammillary nucleus in the rat. Neuroscience, 1985. 16(1): p. 85-110.

63. Yasukawa, T., et al., Rat intralaminar thalamic nuclei projections to the globus pallidus: a biotinylated dextran amine anterograde tracing study. J Comp Neurol, 2004. 471(2): p. 153-67.

64. Vertes, R.P., W.B. Hoover, and J.J. Rodriguez, Projections of the central medial nucleus of the thalamus in the rat: node in cortical, striatal and limbic forebrain circuitry. Neuroscience, 2012. 219: p. 120-36.

65. Moriizumi, T. and T. Hattori, Ultrastructural morphology of projections from the medial geniculate nucleus and its adjacent region to the basal ganglia. Brain Res Bull, 1992. 29(2): p. 193-8.

66. Vertes, R.P. and W.B. Hoover, Projections of the paraventricular and paratenial nuclei of the dorsal midline thalamus in the rat. J Comp Neurol, 2008. 508(2): p. 212-37.

67. Ohtake, T. and H. Yamada, Efferent connections of the nucleus reuniens and the rhomboid nucleus in the rat: an anterograde PHA-L tracing study. Neurosci Res, 1989. 6(6): p. 556-68.

68. Takada, M., Widespread dopaminergic projections of the subparafascicular thalamic nucleus in the rat. Brain Res Bull, 1993. 32(3): p. 301-9.

69. Ricardo, J.A., Efferent connections of the subthalamic region in the rat. I. The subthalamic nucleus of Luys. Brain Res, 1980. 202(2): p. 257-71.

70. Kita, H. and S.T. Kitai, Efferent projections of the subthalamic nucleus in the rat: light and electron microscopic analysis with the PHA-L method. J Comp Neurol, 1987. 260(3): p. 435-52.

71. Groenewegen, H.J. and H.W. Berendse, Connections of the subthalamic nucleus with ventral striatopallidal parts of the basal ganglia in the rat. J Comp Neurol, 1990. 294(4): p. 607-22.

72. Rouzaire-Dubois, B., et al., Microiontophoretic studies on the nature of the neurotransmitter in the subthalamo-entopeduncular pathway of the rat. Brain Res, 1983. 271(1): p. 11-20.

73. Bevan, M.D., J.P. Bolam, and A.R. Crossman, Convergent synaptic input from the neostriatum and the subthalamus onto identified nigrothalamic neurons in the rat. Eur J Neurosci, 1994. 6(3): p. 320-34.

74. Lindvall, O. and A. Bjorklund, Dopaminergic innervation of the globus pallidus by collaterals from the nigrostriatal pathway. Brain Res, 1979. 172(1): p. 169-73.

75. Oleshko, N.N., et al., [Divergence of axon collaterals of substantia nigra neurons in the forebrain of the rat: double labeling with fluorochromes and horseradish peroxidase]. Neirofiziologiia, 1983. 15(5): p. 517-26.

76. Vertes, R.P., A PHA-L analysis of ascending projections of the dorsal raphe nucleus in the rat. J Comp Neurol, 1991. 313(4): p. 643-68.

77. Azmitia, E.C. and M. Segal, An autoradiographic analysis of the differential ascending projections of the dorsal and median raphe nuclei in the rat. J Comp Neurol, 1978. 179(3): p. 641-67.

78. Alden, M., J.M. Besson, and J.F. Bernard, Organization of the efferent projections from the pontine parabrachial area to the bed nucleus of the stria terminalis and neighboring regions: a PHA-L study in the rat. J Comp Neurol, 1994. 341(3): p. 289-314.

79. Bernard, J.F., M. Alden, and J.M. Besson, The organization of the efferent projections from the pontine parabrachial area to the amygdaloid complex: a Phaseolus vulgaris leucoagglutinin (PHA-L) study in the rat. J Comp Neurol, 1993. 329(2): p. 201-29.

80. Naito, A. and H. Kita, The cortico-pallidal projection in the rat: an anterograde tracing study with biotinylated dextran amine. Brain Res, 1994. 653(1-2): p. 251-7.

81. Nagy, J.I., D.A. Carter, and H.C. Fibiger, Anterior striatal projections to the globus pallidus, entopeduncular nucleus and substantia nigra in the rat: the GABA connection. Brain Res, 1978. 158(1): p. 15-29.

82. Cuello, A.C. and G. Paxinos, Evidence for a long Leu-enkephalin striopallidal pathway in rat brain. Nature, 1978. 271(5641): p. 178-80.

83. Gross, A., et al., Differential localization of GABA(A) receptor subunits in relation to rat striatopallidal and pallidopallidal synapses. Eur J Neurosci, 2011. 33(5): p. 868-78.

Ventral pallidum (efferents and afferents)

1. Zaborszky, L., et al., Cholinergic and GABAergic afferents to the olfactory bulb in the rat with special emphasis on the projection neurons in the nucleus of the horizontal limb of the diagonal band. J Comp Neurol, 1986. 243(4): p. 488-509.

2. Mayo, W., et al., Cortical cholinergic projections from the basal forebrain of the rat, with special reference to the prefrontal cortex innervation. Neurosci Lett, 1984. 47(2): p. 149-54.

3. Churchill, L., et al., GABAA receptors containing alpha 1 and beta 2 subunits are mainly localized on neurons in the ventral pallidum. Synapse, 1991. 8(2): p. 75-85.

4. Brog, J.S., et al., The patterns of afferent innervation of the core and shell in the "accumbens" part of the rat ventral striatum: immunohistochemical detection of retrogradely transported fluoro-gold. J Comp Neurol, 1993. 338(2): p. 255-78.

5. Churchill, L. and P.W. Kalivas, A topographically organized gamma-aminobutyric acid projection from the ventral pallidum to the nucleus accumbens in the rat. J Comp Neurol, 1994. 345(4): p. 579-95.

6. Staines, W.A. and H.C. Fibiger, Collateral projections of neurons of the rat globus pallidus to the striatum and substantia nigra. Exp Brain Res, 1984. 56(2): p. 217-20.

7. Ottersen, O.P., Afferent connections to the amygdaloid complex of the rat and cat: II. Afferents from the hypothalamus and the basal telencephalon. J Comp Neurol, 1980. 194(1): p. 267-89.

8. Li, Y.Q., et al., The sites of origin of dopaminergic afferent fibers to the lateral habenular nucleus in the rat. J Comp Neurol, 1993. 333(1): p. 118-33.

9. Mogenson, G.J., et al., Ventral pallidum projections to mediodorsal nucleus of the thalamus: an anatomical and electrophysiological investigation in the rat. Brain Res, 1987. 404(1-2): p. 221-30.

10. Groenewegen, H.J., Organization of the afferent connections of the mediodorsal thalamic nucleus in the rat, related to the mediodorsal-prefrontal topography. Neuroscience, 1988. 24(2): p. 379-431.

11. Ray, J.P., et al., Sources of presumptive glutamatergic/aspartatergic afferents to the mediodorsal nucleus of the thalamus in the rat. J Comp Neurol, 1992. 320(4): p. 435-56.

12. Kuroda, M. and J.L. Price, Synaptic organization of projections from basal forebrain structures to the mediodorsal thalamic nucleus of the rat. J Comp Neurol, 1991. 303(4): p. 513-33.

13. Churchill, L., D.S. Zahm, and P.W. Kalivas, The mediodorsal nucleus of the thalamus in rats--I. forebrain gabaergic innervation. Neuroscience, 1996. 70(1): p. 93-102.

14. Chen, S. and H.S. Su, Afferent connections of the thalamic paraventricular and parataenial nuclei in the rat--a retrograde tracing study with iontophoretic application of Fluoro-Gold. Brain Res, 1990. 522(1): p. 1-6.

15. Cornwall, J., J.D. Cooper, and O.T. Phillipson, Projections to the rostral reticular thalamic nucleus in the rat. Exp Brain Res, 1990. 80(1): p. 157-71.

16. Hallanger, A.E., et al., The origins of cholinergic and other subcortical afferents to the thalamus in the rat. J Comp Neurol, 1987. 262(1): p. 105-24.

17. Groenewegen, H.J., Y. Galis-de Graaf, and W.J. Smeets, Integration and segregation of limbic cortico-striatal loops at the thalamic level: an experimental tracing study in rats. J Chem Neuroanat, 1999. 16(3): p. 167-85.

18. Canteras, N.S., et al., Afferent connections of the subthalamic nucleus: a combined retrograde and anterograde horseradish peroxidase study in the rat. Brain Res, 1990. 513(1): p. 43-59.

19. Bell, K., L. Churchill, and P.W. Kalivas, GABAergic projection from the ventral pallidum and globus pallidus to the subthalamic nucleus. Synapse, 1995. 20(1): p. 10-8.

20. Klitenick, M.A., et al., Topography and functional role of dopaminergic projections from the ventral mesencephalic tegmentum to the ventral pallidum. Neuroscience, 1992. 50(2): p. 371-86.

21. Takada, M. and T. Hattori, Organization of ventral tegmental area cells projecting to the occipital cortex and forebrain in the rat. Brain Res, 1987. 418(1): p. 27-33.

22. Gervasoni, D., et al., Role and origin of the GABAergic innervation of dorsal raphe serotonergic neurons. J Neurosci, 2000. 20(11): p. 4217-25.

23. Behzadi, G., et al., Afferents to the median raphe nucleus of the rat: retrograde cholera toxin and wheat germ conjugated horseradish peroxidase tracing, and selective D-[3H]aspartate labelling of possible excitatory amino acid inputs. Neuroscience, 1990. 37(1): p. 77-100.

24. Haber, S.N., et al., Efferent connections of the ventral pallidum: evidence of a dual striato pallidofugal pathway. J Comp Neurol, 1985. 235(3): p. 322-35.

25. Araki, M., P.L. McGeer, and H. Kimura, The efferent projections of the rat lateral habenular nucleus revealed by the PHA-L anterograde tracing method. Brain Res, 1988. 441(1-2): p. 319-30.

26. Gaykema, R.P., et al., Prefrontal cortical projections to the cholinergic neurons in the basal forebrain. J Comp Neurol, 1991. 303(4): p. 563-83.

27. Sesack, S.R., et al., Topographical organization of the efferent projections of the medial prefrontal cortex in the rat: an anterograde tract-tracing study with Phaseolus vulgaris leucoagglutinin. J Comp Neurol, 1989. 290(2): p. 213-42.

28. Kuo, H. and H.T. Chang, Ventral pallido-striatal pathway in the rat brain: a light and electron microscopic study. J Comp Neurol, 1992. 321(4): p. 626-36.

29. Usuda, I., K. Tanaka, and T. Chiba, Efferent projections of the nucleus accumbens in the rat with special reference to subdivision of the nucleus: biotinylated dextran amine study. Brain Res, 1998. 797(1): p. 73-93.

30. Heimer, L., et al., Specificity in the projection patterns of accumbal core and shell in the rat. Neuroscience, 1991. 41(1): p. 89-125.

31. Zahm, D.S. and L. Heimer, Specificity in the efferent projections of the nucleus accumbens in the rat: comparison of the rostral pole projection patterns with those of the core and shell. J Comp Neurol, 1993. 327(2): p. 220-32.

32. Zhou, L., T. Furuta, and T. Kaneko, Chemical organization of projection neurons in the rat accumbens nucleus and olfactory tubercle. Neuroscience, 2003. 120(3): p. 783-798.

33. Lu, X.Y., M.B. Ghasemzadeh, and P.W. Kalivas, Expression of D1 receptor, D2 receptor, substance P and enkephalin messenger RNAs in the neurons projecting from the nucleus accumbens. Neuroscience, 1998. 82(3): p. 767-80.

34. Lu, X.Y., M.B. Ghasemzadeh, and P.W. Kalivas, Expression of glutamate receptor subunit/subtype messenger RNAS for NMDAR1, GLuR1, GLuR2 and mGLuR5 by accumbal projection neurons. Brain Res Mol Brain Res, 1999. 63(2): p. 287-96.

35. Walaas, I. and F. Fonnum, Biochemical evidence for glutamate as a transmitter in hippocampal efferents to the basal forebrain and hypothalamus in the rat brain. Neuroscience, 1980. 5(10): p. 1691-8.

36. Kalivas, P.W., L. Churchill, and M.A. Klitenick, GABA and enkephalin projection from the nucleus accumbens and ventral pallidum to the ventral tegmental area. Neuroscience, 1993. 57(4): p. 1047-60.

37. Bevan, M.D., N.P. Clarke, and J.P. Bolam, Synaptic integration of functionally diverse pallidal information in the entopeduncular nucleus and subthalamic nucleus in the rat. J Neurosci, 1997. 17(1): p. 308-24.

38. Mascagni, F. and A.J. McDonald, Parvalbumin-immunoreactive neurons and GABAergic neurons of the basal forebrain project to the rat basolateral amygdala. Neuroscience, 2009. 160(4): p. 805-12.

39. Young, W.S., 3rd, G.F. Alheid, and L. Heimer, The ventral pallidal projection to the mediodorsal thalamus: a study with fluorescent retrograde tracers and immunohistofluorescence. J Neurosci, 1984. 4(6): p. 1626-38.

40. Ricardo, J.A., Efferent connections of the subthalamic region in the rat. I. The subthalamic nucleus of Luys. Brain Res, 1980. 202(2): p. 257-71.

41. Kita, H. and S.T. Kitai, Efferent projections of the subthalamic nucleus in the rat: light and electron microscopic analysis with the PHA-L method. J Comp Neurol, 1987. 260(3): p. 435-52.

42. Groenewegen, H.J. and H.W. Berendse, Connections of the subthalamic nucleus with ventral striatopallidal parts of the basal ganglia in the rat. J Comp Neurol, 1990. 294(4): p. 607-22.

43. Rouzaire-Dubois, B., et al., Microiontophoretic studies on the nature of the neurotransmitter in the subthalamo-entopeduncular pathway of the rat. Brain Res, 1983. 271(1): p. 11-20.

44. Takada, M., et al., Two separate neuronal populations of the rat subthalamic nucleus project to the basal ganglia and pedunculopontine tegmental region. Brain Res, 1988. 442(1): p. 72-80.

45. Del-Fava, F., et al., Efferent connections of the rostral linear nucleus of the ventral tegmental area in the rat. Neuroscience, 2007. 145(3): p. 1059-76.

46. Hjelmstad, G.O., et al., Opioid modulation of ventral pallidal afferents to ventral tegmental area neurons. J Neurosci, 2013. 33(15): p. 6454-9.

47. Vertes, R.P., A PHA-L analysis of ascending projections of the dorsal raphe nucleus in the rat. J Comp Neurol, 1991. 313(4): p. 643-68.

48. Walker, R.H., G.W. Arbuthnott, and A.K. Wright, Electrophysiological and anatomical observations concerning the pallidostriatal pathway in the rat. Exp Brain Res, 1989. 74(2): p. 303-10.

Entopeduncular nucleus (efferents and afferents)

1. Takada, M., et al., Direct projections from the entopeduncular nucleus to the lower brainstem in the rat. J Comp Neurol, 1994. 342(3): p. 409-29.

2. Nagy, J.I., et al., Evidence for a GABA-containing projection from the entopeduncular nucleus to the lateral habenula in the rat. Brain Res, 1978. 145(2): p. 360-4.

3. Vincent, S.R. and J.C. Brown, Somatostatin immunoreactivity in the entopeduncular projection to the lateral habenula in the rat. Neurosci Lett, 1986. 68(2): p. 160-4.

4. Schmued, L., et al., Collateralization and GAD immunoreactivity of descending pallidal efferents. Brain Res, 1989. 487(1): p. 131-42.

5. Carter, D.A. and H.C. Fibiger, The projections of the entopeduncular nucleus and globus pallidus in rat as demonstrated by autoradiography and horseradish peroxidase histochemistry. J Comp Neurol, 1978. 177(1): p. 113-23.

6. Kim, U. and S.Y. Chang, Dendritic morphology, local circuitry, and intrinsic electrophysiology of neurons in the rat medial and lateral habenular nuclei of the epithalamus. J Comp Neurol, 2005. 483(2): p. 236-50.

7. Li, Y.Q., et al., The sites of origin of dopaminergic afferent fibers to the lateral habenular nucleus in the rat. J Comp Neurol, 1993. 333(1): p. 118-33.

8. Herkenham, M. and W.J. Nauta, Afferent connections of the habenular nuclei in the rat. A horseradish peroxidase study, with a note on the fiber-of-passage problem. J Comp Neurol, 1977. 173(1): p. 123-46.

9. Qu, T., et al., Demonstration of direct input from the retina to the lateral habenular nucleus in the albino rat. Brain Res, 1996. 709(2): p. 251-58.

10. Araki, M., P.L. McGeer, and E.G. McGeer, Retrograde HRP tracing combined with a pharmacohistochemical method for GABA transaminase for the identification of presumptive GABAergic projections to the habenula. Brain Res, 1984. 304(2): p. 271-7.

11. Ray, J.P., et al., Sources of presumptive glutamatergic/aspartatergic afferents to the mediodorsal nucleus of the thalamus in the rat. J Comp Neurol, 1992. 320(4): p. 435-56.

12. Hallanger, A.E., et al., The origins of cholinergic and other subcortical afferents to the thalamus in the rat. J Comp Neurol, 1987. 262(1): p. 105-24.

13. Kha, H.T., et al., Study of projections from the entopeduncular nucleus to the thalamus of the rat. J Comp Neurol, 2000. 426(3): p. 366-77.

14. Sugimoto, T. and T. Hattori, Direct projections from the globus pallidus to the paraventricular nucleus of the thalamus in the rat. Brain Res, 1984. 323(1): p. 188-92.

15. Herkenham, M., The afferent and efferent connections of the ventromedial thalamic nucleus in the rat. J Comp Neurol, 1979. 183(3): p. 487-517.

16. Finkelstein, D.I., A.K. Reeves, and M.K. Horne, An electron microscopic tracer study of the projections from entopeduncular nucleus to the ventrolateral nucleus of the rat. Neurosci Lett, 1996. 211(1): p. 33-6.

17. Rouzaire-Dubois, B., et al., Microiontophoretic studies on the nature of the neurotransmitter in the subthalamo-entopeduncular pathway of the rat. Brain Res, 1983. 271(1): p. 11-20.

18. Hay-Schmidt, A. and J.D. Mikkelsen, Demonstration of a neuronal projection from the entopeduncular nucleus to the substantia nigra of the rat. Brain Res, 1992. 576(2): p. 343-7.

19. Jackson, A. and A.R. Crossman, Basal ganglia and other afferent projections to the peribrachial region in the rat: a study using retrograde and anterograde transport of horseradish peroxidase. Neuroscience, 1981. 6(8): p. 1537-49.

20. Conrad, L.C. and D.W. Pfaff, Autoradiographic tracing of nucleus accumbens efferents in the rat. Brain Res, 1976. 113(3): p. 589-96.

21. Heimer, L., et al., Specificity in the projection patterns of accumbal core and shell in the rat. Neuroscience, 1991. 41(1): p. 89-125.

22. Fink-Jensen, A. and J.D. Mikkelsen, The striato-entopeduncular pathway in the rat. A retrograde transport study with wheatgerm-agglutinin-horseradish peroxidase. Brain Res, 1989. 476(1): p. 194-8.

23. Wu, Y., S. Richard, and A. Parent, The organization of the striatal output system: a single-cell juxtacellular labeling study in the rat. Neurosci Res, 2000. 38(1): p. 49-62.

24. Ricardo, J.A., Efferent connections of the subthalamic region in the rat. I. The subthalamic nucleus of Luys. Brain Res, 1980. 202(2): p. 257-71.

25. Kita, H. and S.T. Kitai, Efferent projections of the subthalamic nucleus in the rat: light and electron microscopic analysis with the PHA-L method. J Comp Neurol, 1987. 260(3): p. 435-52.

26. Chang, H.T., C.J. Wilson, and S.T. Kitai, A Golgi study of rat neostriatal neurons: light microscopic analysis. J Comp Neurol, 1982. 208(2): p. 107-26.

27. Nakano, K., Neural circuits and topographic organization of the basal ganglia and related regions. Brain Dev, 2000. 22 Suppl 1: p. S5-16.

28. Groenewegen, H.J. and H.W. Berendse, Connections of the subthalamic nucleus with ventral striatopallidal parts of the basal ganglia in the rat. J Comp Neurol, 1990. 294(4): p. 607-22.

29. Bevan, M.D., A.R. Crossman, and J.P. Bolam, Neurons projecting from the entopeduncular nucleus to the thalamus receive convergent synaptic inputs from the subthalamic nucleus and the neostriatum in the rat. Brain Res, 1994. 659(1-2): p. 99-109.

30. Arbuthnott, G.W., et al., Distribution and synaptic contacts of the cortical terminals arising from neurons in the rat ventromedial thalamic nucleus. Neuroscience, 1990. 38(1): p. 47-60.

31. Nagy, J.I., D.A. Carter, and H.C. Fibiger, Anterior striatal projections to the globus pallidus, entopeduncular nucleus and substantia nigra in the rat: the GABA connection. Brain Res, 1978. 158(1): p. 15-29.

32. Bolam, J.P. and Y. Smith, The striatum and the globus pallidus send convergent synaptic inputs onto single cells in the entopeduncular nucleus of the rat: a double anterograde labelling study combined with postembedding immunocytochemistry for GABA. J Comp Neurol, 1992. 321(3): p. 456-76.

**Hypothalamus**

Intraregional connections

1. Swanson, L.W., An autoradiographic study of the efferent connections of the preoptic region in the rat. J Comp Neurol, 1976. 167(2): p. 227-56.

2. Vertes, R.P., et al., Ascending projections of the posterior nucleus of the hypothalamus: PHA-L analysis in the rat. J Comp Neurol, 1995. 359(1): p. 90-116.

3. Zaborszky, L. and G.B. Makara, Intrahypothalamic connections: an electron microscopic study in the rat. Exp Brain Res, 1979. 34(2): p. 201-15.

4. Canteras, N.S., R.B. Simerly, and L.W. Swanson, Projections of the ventral premammillary nucleus. J Comp Neurol, 1992. 324(2): p. 195-212.

5. Canteras, N.S. and L.W. Swanson, The dorsal premammillary nucleus: an unusual component of the mammillary body. Proc Natl Acad Sci U S A, 1992. 89(21): p. 10089-93.

6. Staines, W.A., et al., The hypothalamus receives major projections from the tuberomammillary nucleus in rat. Neurosci Lett, 1987. 76(3): p. 257-62.

7. Sim, L.J. and S.A. Joseph, Arcuate nucleus projections to brainstem regions which modulate nociception. J Chem Neuroanat, 1991. 4(2): p. 97-109.

8. ter Horst, G.J. and P.G. Luiten, The projections of the dorsomedial hypothalamic nucleus in the rat. Brain Res Bull, 1986. 16(2): p. 231-48.

9. Aarnisalo, A.A. and P. Panula, Neuropeptide FF-containing efferent projections from the medial hypothalamus of rat: a Phaseolus vulgaris leucoagglutinin study. Neuroscience, 1995. 65(1): p. 175-92.

10. Berk, M.L. and J.A. Finkelstein, Efferent connections of the lateral hypothalamic area of the rat: an autoradiographic investigation. Brain Res Bull, 1982. 8(5): p. 511-26.

11. Goto, M., et al., Projections from the subfornical region of the lateral hypothalamic area. J Comp Neurol, 2005. 493(3): p. 412-38.

12. Saper, C.B., L.W. Swanson, and W.M. Cowan, An autoradiographic study of the efferent connections of the lateral hypothalamic area in the rat. J Comp Neurol, 1979. 183(4): p. 689-706.

13. Gu, G.B. and R.B. Simerly, Projections of the sexually dimorphic anteroventral periventricular nucleus in the female rat. J Comp Neurol, 1997. 384(1): p. 142-64.

14. Saper, C.B., L.W. Swanson, and W.M. Cowan, The efferent connections of the ventromedial nucleus of the hypothalamus of the rat. J Comp Neurol, 1976. 169(4): p. 409-42.

15. Risold, P.Y., N.S. Canteras, and L.W. Swanson, Organization of projections from the anterior hypothalamic nucleus: a Phaseolus vulgaris-leucoagglutinin study in the rat. J Comp Neurol, 1994. 348(1): p. 1-40.

16. Conrad, L.C. and D.W. Pfaff, Efferents from medial basal forebrain and hypothalamus in the rat. II. An autoradiographic study of the anterior hypothalamus. J Comp Neurol, 1976. 169(2): p. 221-61.

17. Vertes, R.P., PHA-L analysis of projections from the supramammillary nucleus in the rat. J Comp Neurol, 1992. 326(4): p. 595-622.

18. Saper, C.B. and D. Levisohn, Afferent connections of the median preoptic nucleus in the rat: anatomical evidence for a cardiovascular integrative mechanism in the anteroventral third ventricular (AV3V) region. Brain Res, 1983. 288(1-2): p. 21-31.

19. Swanson, L.W. and H.G. Kuypers, The paraventricular nucleus of the hypothalamus: cytoarchitectonic subdivisions and organization of projections to the pituitary, dorsal vagal complex, and spinal cord as demonstrated by retrograde fluorescence double-labeling methods. J Comp Neurol, 1980. 194(3): p. 555-70.

20. Simerly, R.B. and L.W. Swanson, The organization of neural inputs to the medial preoptic nucleus of the rat. J Comp Neurol, 1986. 246(3): p. 312-42.

21. Coolen, L.M., H.J. Peters, and J.G. Veening, Anatomical interrelationships of the medial preoptic area and other brain regions activated following male sexual behavior: a combined fos and tract-tracing study. J Comp Neurol, 1998. 397(3): p. 421-35.

22. Watts, A.G., L.W. Swanson, and G. Sanchez-Watts, Efferent projections of the suprachiasmatic nucleus: I. Studies using anterograde transport of Phaseolus vulgaris leucoagglutinin in the rat. J Comp Neurol, 1987. 258(2): p. 204-29.

23. Leak, R.K. and R.Y. Moore, Topographic organization of suprachiasmatic nucleus projection neurons. J Comp Neurol, 2001. 433(3): p. 312-34.

24. Hoffman, G.E., et al., The hypothalamic ventromedial nucleus sends a met-enkephalin projection to the preoptic area's periventricular zone in the female rat. Brain Res Mol Brain Res, 1996. 36(2): p. 201-10.

25. Csaki, A., et al., Localization of glutamatergic/aspartatergic neurons projecting to the hypothalamic paraventricular nucleus studied by retrograde transport of [3H]D-aspartate autoradiography. Neuroscience, 2000. 101(3): p. 637-55.

26. Champagne, D., J. Beaulieu, and G. Drolet, CRFergic innervation of the paraventricular nucleus of the rat hypothalamus: a tract-tracing study. J Neuroendocrinol, 1998. 10(2): p. 119-31.

27. Sawchenko, P.E. and L.W. Swanson, The organization of forebrain afferents to the paraventricular and supraoptic nuclei of the rat. J Comp Neurol, 1983. 218(2): p. 121-44.

28. Beaulieu, J., D. Champagne, and G. Drolet, Enkephalin innervation of the paraventricular nucleus of the hypothalamus: distribution of fibers and origins of input. J Chem Neuroanat, 1996. 10(2): p. 79-92.

29. Simerly, R.B. and L.W. Swanson, Projections of the medial preoptic nucleus: a Phaseolus vulgaris leucoagglutinin anterograde tract-tracing study in the rat. J Comp Neurol, 1988. 270(2): p. 209-42.

30. Ter Horst, G.J. and P.G. Luiten, Phaseolus vulgaris leuco-agglutinin tracing of intrahypothalamic connections of the lateral, ventromedial, dorsomedial and paraventricular hypothalamic nuclei in the rat. Brain Res Bull, 1987. 18(2): p. 191-203.

31. Watts, A.G. and L.W. Swanson, Efferent projections of the suprachiasmatic nucleus: II. Studies using retrograde transport of fluorescent dyes and simultaneous peptide immunohistochemistry in the rat. J Comp Neurol, 1987. 258(2): p. 230-52.

32. Magoul, R., et al., Direct and indirect enkephalinergic synaptic inputs to the rat arcuate nucleus studied by combination of retrograde tracing and immunocytochemistry. Neuroscience, 1993. 55(4): p. 1055-66.

33. Campbell, R.E., K.L. Grove, and M.S. Smith, Distribution of corticotropin releasing hormone receptor immunoreactivity in the rat hypothalamus: coexpression in neuropeptide Y and dopamine neurons in the arcuate nucleus. Brain Research, 2003. 973(2): p. 223-232.

34. Li, C., P. Chen, and M.S. Smith, Corticotropin releasing hormone neurons in the paraventricular nucleus are direct targets for neuropeptide Y neurons in the arcuate nucleus: an anterograde tracing study. Brain Res, 2000. 854(1-2): p. 122-9.

35. Jhanwar-Uniyal, M., et al., Neuropeptide Y projection from arcuate nucleus to parvocellular division of paraventricular nucleus: specific relation to the ingestion of carbohydrate. Brain Res, 1993. 631(1): p. 97-106.

36. Abrahamson, E.E. and R.Y. Moore, The posterior hypothalamic area: chemoarchitecture and afferent connections. Brain Res, 2001. 889(1-2): p. 1-22.

37. Comoli, E., E.R. Ribeiro-Barbosa, and N.S. Canteras, Afferent connections of the dorsal premammillary nucleus. J Comp Neurol, 2000. 423(1): p. 83-98.

38. Moga, M.M. and R.Y. Moore, Organization of neural inputs to the suprachiasmatic nucleus in the rat. J Comp Neurol, 1997. 389(3): p. 508-34.

39. Luiten, P.G. and P. Room, Interrelations between lateral, dorsomedial and ventromedial hypothalamic nuclei in the rat. An HRP study. Brain Res, 1980. 190(2): p. 321-32.

40. Thompson, R.H. and L.W. Swanson, Organization of inputs to the dorsomedial nucleus of the hypothalamus: a reexamination with Fluorogold and PHAL in the rat. Brain Res Brain Res Rev, 1998. 27(2): p. 89-118.

41. Alamilla, J. and R. Aguilar-Roblero, Glutamate and GABA neurotransmission from the paraventricular thalamus to the suprachiasmatic nuclei in the rat. J Biol Rhythms, 2010. 25(1): p. 28-36.

42. Alamilla, J. and R. Aguilar-Roblero, Glutamate and GABA neurotransmission from the paraventricular thalamus to the suprachiasmatic nuclei in the rat. J Biol Rhythms, 2010. 25(1): p. 28-36.

43. Swanson, L.W. and W.M. Cowan, The efferent connections of the suprachiasmatic nucleus of the hypothalamus. J Comp Neurol, 1975. 160(1): p. 1-12.

44. Ter Horst, G.J., et al., Ascending projections from the solitary tract nucleus to the hypothalamus. A Phaseolus vulgaris lectin tracing study in the rat. Neuroscience, 1989. 31(3): p. 785-97.

45. Ter Horst, G.J., et al., Projections from the rostral parvocellular reticular formation to pontine and medullary nuclei in the rat: involvement in autonomic regulation and orofacial motor control. Neuroscience, 1991. 40(3): p. 735-58.

46. Millhouse, O.E., The organization of the ventromedial hypothalamic nucleus. Brain Res, 1973. 55(1): p. 71-87.

47. Shibata, H., Descending projections to the mammillary nuclei in the rat, as studied by retrograde and anterograde transport of wheat germ agglutinin-horseradish peroxidase. J Comp Neurol, 1989. 285(4): p. 436-52.

48. Gonzalo-Ruiz, A., et al., Afferent projections to the mammillary complex of the rat, with special reference to those from surrounding hypothalamic regions. J Comp Neurol, 1992. 321(2): p. 277-99.

49. Shibata, H., Ascending projections to the mammillary nuclei in the rat: a study using retrograde and anterograde transport of wheat germ agglutinin conjugated to horseradish peroxidase. J Comp Neurol, 1987. 264(2): p. 205-15.

50. Villalobos, J. and A. Ferssiwi, The differential descending projections from the anterior, central and posterior regions of the lateral hypothalamic area: an autoradiographic study. Neurosci Lett, 1987. 81(1-2): p. 95-9.

51. Gonzalo-Ruiz, A., et al., A dopaminergic projection to the rat mammillary nuclei demonstrated by retrograde transport of wheat germ agglutinin-horseradish peroxidase and tyrosine hydroxylase immunohistochemistry. J Comp Neurol, 1992. 321(2): p. 300-11.

52. Hayakawa, T., H. Ito, and K. Zyo, Neuroanatomical study of afferent projections to the supramammillary nucleus of the rat. Anat Embryol (Berl), 1993. 188(2): p. 139-48.

53. Kiss, J., et al., Possible glutamatergic/aspartatergic projections to the supramammillary nucleus and their origins in the rat studied by selective [(3)H]D-aspartate labelling and immunocytochemistry. Neuroscience, 2002. 111(3): p. 671-91.

54. Cui, L.N., K. Saeb-Parsy, and R.E. Dyball, Neurones in the supraoptic nucleus of the rat are regulated by a projection from the suprachiasmatic nucleus. J Physiol, 1997. 502 ( Pt 1): p. 149-59.

55. Ludwig, M. and G. Leng, GABAergic projection from the arcuate nucleus to the supraoptic nucleus in the rat. Neurosci Lett, 2000. 281(2-3): p. 195-7.

56. Fahrbach, S.E., J.I. Morrell, and D.W. Pfaff, Studies of ventromedial hypothalamic afferents in the rat using three methods of HRP application. Exp Brain Res, 1989. 77(2): p. 221-33.

Hypothalamus - anterior hypothalamic nucleus (efferents and afferents)

1. Risold, P.Y., N.S. Canteras, and L.W. Swanson, Organization of projections from the anterior hypothalamic nucleus: a Phaseolus vulgaris-leucoagglutinin study in the rat. J Comp Neurol, 1994. 348(1): p. 1-40.

2. Conrad, L.C. and D.W. Pfaff, Efferents from medial basal forebrain and hypothalamus in the rat. II. An autoradiographic study of the anterior hypothalamus. J Comp Neurol, 1976. 169(2): p. 221-61.

3. Watts, A.G. and L.W. Swanson, Efferent projections of the suprachiasmatic nucleus: II. Studies using retrograde transport of fluorescent dyes and simultaneous peptide immunohistochemistry in the rat. J Comp Neurol, 1987. 258(2): p. 230-52.

4. Sakanaka, M., et al., Corticotropin releasing factor-containing afferents to the lateral septum of the rat brain. J Comp Neurol, 1988. 270(3): p. 404-15, 396-7.

5. Renaud, L.P. and D.A. Hopkins, Amygdala afferents from the mediobasal hypothalamus: an electrophysiological and neuroanatomical study in the rat. Brain Res, 1977. 121(2): p. 201-13.

6. Cornwall, J. and O.T. Phillipson, Afferent projections to the dorsal thalamus of the rat as shown by retrograde lectin transport. II. The midline nuclei. Brain Res Bull, 1988. 21(2): p. 147-61.

7. Swanson, L.W. and W.M. Cowan, The efferent connections of the suprachiasmatic nucleus of the hypothalamus. J Comp Neurol, 1975. 160(1): p. 1-12.

8. Herkenham, M., The connections of the nucleus reuniens thalami: evidence for a direct thalamo-hippocampal pathway in the rat. J Comp Neurol, 1978. 177(4): p. 589-610.

9. McKenna, J.T. and R.P. Vertes, Afferent projections to nucleus reuniens of the thalamus. J Comp Neurol, 2004. 480(2): p. 115-42.

10. Geisler, S. and D.S. Zahm, Afferents of the ventral tegmental area in the rat-anatomical substratum for integrative functions. J Comp Neurol, 2005. 490(3): p. 270-94.

11. Greenwell, T.N., et al., Endomorphin-1 and -2 immunoreactive cells in the hypothalamus are labeled by fluoro-gold injections to the ventral tegmental area. J Comp Neurol, 2002. 454(3): p. 320-8.

12. Peschanski, M. and J.M. Besson, Diencephalic connections of the raphe nuclei of the rat brainstem: an anatomical study with reference to the somatosensory system. J Comp Neurol, 1984. 224(4): p. 509-34.

13. Luppi, P.H., et al., Afferent projections to the rat locus coeruleus demonstrated by retrograde and anterograde tracing with cholera-toxin B subunit and Phaseolus vulgaris leucoagglutinin. Neuroscience, 1995. 65(1): p. 119-60.

14. Takagishi, M. and T. Chiba, Efferent projections of the infralimbic (area 25) region of the medial prefrontal cortex in the rat: an anterograde tracer PHA-L study. Brain Res, 1991. 566(1-2): p. 26-39.

15. Conrad, L.C. and D.W. Pfaff, Autoradiographic tracing of nucleus accumbens efferents in the rat. Brain Res, 1976. 113(3): p. 589-96.

16. Varoqueaux, F. and P. Poulain, Projections of the mediolateral part of the lateral septum to the hypothalamus, revealed by Fos expression and axonal tracing in rats. Anat Embryol (Berl), 1999. 199(3): p. 249-63.

17. Dong, H.W. and L.W. Swanson, Projections from bed nuclei of the stria terminalis, anteromedial area: cerebral hemisphere integration of neuroendocrine, autonomic, and behavioral aspects of energy balance. J Comp Neurol, 2006. 494(1): p. 142-78.

18. Dong, H.W. and L.W. Swanson, Projections from bed nuclei of the stria terminalis, dorsomedial nucleus: implications for cerebral hemisphere integration of neuroendocrine, autonomic, and drinking responses. J Comp Neurol, 2006. 494(1): p. 75-107.

19. Dong, H.-W. and L.W. Swanson, Projections from bed nuclei of the stria terminalis, magnocellular nucleus: Implications for cerebral hemisphere regulation of micturition, defecation, and penile erection. The Journal of Comparative Neurology, 2006. 494(1): p. 108-141.

20. Dong, H.W. and L.W. Swanson, Projections from bed nuclei of the stria terminalis, posterior division: implications for cerebral hemisphere regulation of defensive and reproductive behaviors. J Comp Neurol, 2004. 471(4): p. 396-433.

21. Shammah-Lagnado, S.J., et al., Supracapsular bed nucleus of the stria terminalis contains central and medial extended amygdala elements: evidence from anterograde and retrograde tracing experiments in the rat. J Comp Neurol, 2000. 422(4): p. 533-55.

22. Petrovich, G.D., P.Y. Risold, and L.W. Swanson, Organization of projections from the basomedial nucleus of the amygdala: a PHAL study in the rat. J Comp Neurol, 1996. 374(3): p. 387-420.

23. Prewitt, C.M. and J.P. Herman, Anatomical interactions between the central amygdaloid nucleus and the hypothalamic paraventricular nucleus of the rat: a dual tract-tracing analysis. J Chem Neuroanat, 1998. 15(3): p. 173-85.

24. Canteras, N.S., R.B. Simerly, and L.W. Swanson, Organization of projections from the medial nucleus of the amygdala: a PHAL study in the rat. J Comp Neurol, 1995. 360(2): p. 213-45.

25. Moga, M.M. and R.Y. Moore, Organization of neural inputs to the suprachiasmatic nucleus in the rat. J Comp Neurol, 1997. 389(3): p. 508-34.

26. Canteras, N.S. and L.W. Swanson, Projections of the ventral subiculum to the amygdala, septum, and hypothalamus: a PHAL anterograde tract-tracing study in the rat. J Comp Neurol, 1992. 324(2): p. 180-94.

27. Donovan, M.K. and J.M. Wyss, Evidence for some collateralization between cortical and diencephalic efferent axons of the rat subicular cortex. Brain Res, 1983. 259(2): p. 181-92.

28. Kishi, T., et al., Topographical organization of projections from the subiculum to the hypothalamus in the rat. J Comp Neurol, 2000. 419(2): p. 205-22.

29. Ohtake, T. and H. Yamada, Efferent connections of the nucleus reuniens and the rhomboid nucleus in the rat: an anterograde PHA-L tracing study. Neurosci Res, 1989. 6(6): p. 556-68.

30. Azmitia, E.C. and M. Segal, An autoradiographic analysis of the differential ascending projections of the dorsal and median raphe nuclei in the rat. J Comp Neurol, 1978. 179(3): p. 641-67.

31. Alden, M., J.M. Besson, and J.F. Bernard, Organization of the efferent projections from the pontine parabrachial area to the bed nucleus of the stria terminalis and neighboring regions: a PHA-L study in the rat. J Comp Neurol, 1994. 341(3): p. 289-314.

32. Bester, H., J.M. Besson, and J.F. Bernard, Organization of efferent projections from the parabrachial area to the hypothalamus: a Phaseolus vulgaris-leucoagglutinin study in the rat. J Comp Neurol, 1997. 383(3): p. 245-81.

Hypothalamus - lateral hypothalamus (efferents and afferents)

1. de Olmos, J., H. Hardy, and L. Heimer, The afferent connections of the main and the accessory olfactory bulb formations in the rat: an experimental HRP-study. J Comp Neurol, 1978. 181(2): p. 213-44.

2. Jasmin, L., et al., Rostral agranular insular cortex and pain areas of the central nervous system: a tract-tracing study in the rat. J Comp Neurol, 2004. 468(3): p. 425-40.

3. Shi, C.J. and M.D. Cassell, Cortical, thalamic, and amygdaloid connections of the anterior and posterior insular cortices. J Comp Neurol, 1998. 399(4): p. 440-68.

4. Saper, C.B., Convergence of autonomic and limbic connections in the insular cortex of the rat. J Comp Neurol, 1982. 210(2): p. 163-73.

5. Brog, J.S., et al., The patterns of afferent innervation of the core and shell in the "accumbens" part of the rat ventral striatum: immunohistochemical detection of retrogradely transported fluoro-gold. J Comp Neurol, 1993. 338(2): p. 255-78.

6. Saper, C.B., L.W. Swanson, and W.M. Cowan, An autoradiographic study of the efferent connections of the lateral hypothalamic area in the rat. J Comp Neurol, 1979. 183(4): p. 689-706.

7. Berk, M.L. and J.A. Finkelstein, Efferent connections of the lateral hypothalamic area of the rat: an autoradiographic investigation. Brain Res Bull, 1982. 8(5): p. 511-26.

8. Goto, M., et al., Projections from the subfornical region of the lateral hypothalamic area. J Comp Neurol, 2005. 493(3): p. 412-38.

9. Varoqueaux, F. and P. Poulain, Projections of the mediolateral part of the lateral septum to the hypothalamus, revealed by Fos expression and axonal tracing in rats. Anat Embryol (Berl), 1999. 199(3): p. 249-63.

10. Meibach, R.C. and A. Siegel, Efferent connections of the septal area in the rat: an analysis utilizing retrograde and anterograde transport methods. Brain Res, 1977. 119(1): p. 1-20.

11. Segal, M. and S.C. Landis, Afferents to the septal area of the rat studied with the method of retrograde axonal transport of horseradish peroxidase. Brain Res, 1974. 82(2): p. 263-8.

12. Haber, S.N., et al., Efferent connections of the ventral pallidum: evidence of a dual striato pallidofugal pathway. J Comp Neurol, 1985. 235(3): p. 322-35.

13. Bevan, M.D., N.P. Clarke, and J.P. Bolam, Synaptic integration of functionally diverse pallidal information in the entopeduncular nucleus and subthalamic nucleus in the rat. J Neurosci, 1997. 17(1): p. 308-24.

14. Renaud, L.P. and D.A. Hopkins, Amygdala afferents from the mediobasal hypothalamus: an electrophysiological and neuroanatomical study in the rat. Brain Res, 1977. 121(2): p. 201-13.

15. Ottersen, O.P., Afferent connections to the amygdaloid complex of the rat and cat: II. Afferents from the hypothalamus and the basal telencephalon. J Comp Neurol, 1980. 194(1): p. 267-89.

16. Canteras, N.S., R.B. Simerly, and L.W. Swanson, Connections of the posterior nucleus of the amygdala. J Comp Neurol, 1992. 324(2): p. 143-79.

17. Kim, U. and S.Y. Chang, Dendritic morphology, local circuitry, and intrinsic electrophysiology of neurons in the rat medial and lateral habenular nuclei of the epithalamus. J Comp Neurol, 2005. 483(2): p. 236-50.

18. Li, Y.Q., et al., The sites of origin of dopaminergic afferent fibers to the lateral habenular nucleus in the rat. J Comp Neurol, 1993. 333(1): p. 118-33.

19. Herkenham, M. and W.J. Nauta, Afferent connections of the habenular nuclei in the rat. A horseradish peroxidase study, with a note on the fiber-of-passage problem. J Comp Neurol, 1977. 173(1): p. 123-46.

20. Qu, T., et al., Demonstration of direct input from the retina to the lateral habenular nucleus in the albino rat. Brain Res, 1996. 709(2): p. 251-58.

21. Araki, M., P.L. McGeer, and E.G. McGeer, Retrograde HRP tracing combined with a pharmacohistochemical method for GABA transaminase for the identification of presumptive GABAergic projections to the habenula. Brain Res, 1984. 304(2): p. 271-7.

22. Poller, W.C., et al., A glutamatergic projection from the lateral hypothalamus targets Ventral Tegmental Area -projecting neurons in the lateral habenula of the rat. Brain Res, 2013. 1507: p. 45-60.

23. Shinoda, K. and M. Tohyama, Analysis of the habenulopetal enkephalinergic system in the rat brain: an immunohistochemical study. J Comp Neurol, 1987. 255(4): p. 483-96.

24. Sakanaka, M., et al., Topographic organization of the projection from the forebrain subcortical areas to the hippocampal formation of the rat. Neurosci Lett, 1980. 20(3): p. 253-7.

25. Pasquier, D.A. and F. Reinoso-Suarez, Direct projections from hypothalamus to hippocampus in the rat demonstrated by retrograde transport of horseradish peroxidase. Brain Res, 1976. 108(1): p. 165-9.

26. Calderazzo, L., et al., Branched connections to the septum and to the entorhinal cortex from the hippocampus, amygdala, and diencephalon in the rat. Brain Res Bull, 1996. 40(4): p. 245-51.

27. Kohler, C., L. Haglund, and L.W. Swanson, A diffuse alpha MSH-immunoreactive projection to the hippocampus and spinal cord from individual neurons in the lateral hypothalamic area and zona incerta. J Comp Neurol, 1984. 223(4): p. 501-14.

28. Cornwall, J. and O.T. Phillipson, Afferent projections to the dorsal thalamus of the rat as shown by retrograde lectin transport. II. The midline nuclei. Brain Res Bull, 1988. 21(2): p. 147-61.

29. Groenewegen, H.J., Organization of the afferent connections of the mediodorsal thalamic nucleus in the rat, related to the mediodorsal-prefrontal topography. Neuroscience, 1988. 24(2): p. 379-431.

30. Ray, J.P., et al., Sources of presumptive glutamatergic/aspartatergic afferents to the mediodorsal nucleus of the thalamus in the rat. J Comp Neurol, 1992. 320(4): p. 435-56.

31. Hallanger, A.E., et al., The origins of cholinergic and other subcortical afferents to the thalamus in the rat. J Comp Neurol, 1987. 262(1): p. 105-24.

32. Yoshida, A., J.O. Dostrovsky, and C.Y. Chiang, The afferent and efferent connections of the nucleus submedius in the rat. J Comp Neurol, 1992. 324(1): p. 115-33.

33. Otake, K., D.A. Ruggiero, and Y. Nakamura, Adrenergic innervation of forebrain neurons that project to the paraventricular thalamic nucleus in the rat. Brain Res, 1995. 697(1-2): p. 17-26.

34. Arluison, M. and P. Derer, Forebrain connections of the rat paraventricular thalamic nucleus as demonstrated using the carbocyanide dye DiI. Neurobiology (Bp), 1993. 1(4): p. 337-50.

35. Sugimoto, T. and T. Hattori, Direct projections from the globus pallidus to the paraventricular nucleus of the thalamus in the rat. Brain Res, 1984. 323(1): p. 188-92.

36. McKenna, J.T. and R.P. Vertes, Afferent projections to nucleus reuniens of the thalamus. J Comp Neurol, 2004. 480(2): p. 115-42.

37. Coolen, L.M., et al., Afferent connections of the parvocellular subparafascicular thalamic nucleus in the rat: evidence for functional subdivisions. J Comp Neurol, 2003. 463(2): p. 132-56.

38. Villalobos, J. and A. Ferssiwi, The differential descending projections from the anterior, central and posterior regions of the lateral hypothalamic area: an autoradiographic study. Neurosci Lett, 1987. 81(1-2): p. 95-9.

39. Greenwell, T.N., et al., Endomorphin-1 and -2 immunoreactive cells in the hypothalamus are labeled by fluoro-gold injections to the ventral tegmental area. J Comp Neurol, 2002. 454(3): p. 320-8.

40. Geisler, S. and D.S. Zahm, Afferents of the ventral tegmental area in the rat-anatomical substratum for integrative functions. J Comp Neurol, 2005. 490(3): p. 270-94.

41. Fadel, J. and A.Y. Deutch, Anatomical substrates of orexin-dopamine interactions: lateral hypothalamic projections to the ventral tegmental area. Neuroscience, 2002. 111(2): p. 379-87.

42. Peschanski, M. and J.M. Besson, Diencephalic connections of the raphe nuclei of the rat brainstem: an anatomical study with reference to the somatosensory system. J Comp Neurol, 1984. 224(4): p. 509-34.

43. Lee, H.S., et al., Glutamatergic afferent projections to the dorsal raphe nucleus of the rat. Brain Research, 2003. 963(1-2): p. 57-71.

44. Sim, L.J. and S.A. Joseph, Opiocortin and catecholamine projections to raphe nuclei. Peptides, 1989. 10(5): p. 1019-25.

45. Gervasoni, D., et al., Role and origin of the GABAergic innervation of dorsal raphe serotonergic neurons. J Neurosci, 2000. 20(11): p. 4217-25.

46. Behzadi, G., et al., Afferents to the median raphe nucleus of the rat: retrograde cholera toxin and wheat germ conjugated horseradish peroxidase tracing, and selective D-[3H]aspartate labelling of possible excitatory amino acid inputs. Neuroscience, 1990. 37(1): p. 77-100.

47. Hermann, D.M., et al., Afferent projections to the rat nuclei raphe magnus, raphe pallidus and reticularis gigantocellularis pars alpha demonstrated by iontophoretic application of choleratoxin (subunit b). J Chem Neuroanat, 1997. 13(1): p. 1-21.

48. Lee, H.S., M.A. Kim, and B.D. Waterhouse, Retrograde double-labeling study of common afferent projections to the dorsal raphe and the nuclear core of the locus coeruleus in the rat. J Comp Neurol, 2005. 481(2): p. 179-93.

49. Luppi, P.H., et al., Afferent projections to the rat locus coeruleus demonstrated by retrograde and anterograde tracing with cholera-toxin B subunit and Phaseolus vulgaris leucoagglutinin. Neuroscience, 1995. 65(1): p. 119-60.

50. Milner, T.A. and V.M. Pickel, Ultrastructural localization and afferent sources of substance P in the rat parabrachial region. Neuroscience, 1986. 17(3): p. 687-707.

51. Moga, M.M., C.B. Saper, and T.S. Gray, Neuropeptide organization of the hypothalamic projection to the parabrachial nucleus in the rat. J Comp Neurol, 1990. 295(4): p. 662-82.

52. Moga, M.M., et al., Organization of cortical, basal forebrain, and hypothalamic afferents to the parabrachial nucleus in the rat. J Comp Neurol, 1990. 295(4): p. 624-61.

53. Hosoya, Y. and M. Matsushita, Brainstem projections from the lateral hypothalamic area in the rat, as studied with autoradiography. Neurosci Lett, 1981. 24(2): p. 111-6.

54. Touzani, K., A. Ferssiwi, and L. Velley, Localization of lateral hypothalamic neurons projecting to the medial part of the parabrachial area of the rat. Neurosci Lett, 1990. 114(1): p. 17-21.

55. Hosoya, Y., et al., Direct projection from the dorsal hypothalamic area to the nucleus raphe pallidus: a study using anterograde transport with Phaseolus vulgaris leucoagglutinin in the rat. Exp Brain Res, 1989. 75(1): p. 40-6.

56. Saper, C.B., H. Akil, and S.J. Watson, Lateral hypothalamic innervation of the cerebral cortex: immunoreactive staining for a peptide resembling but immunochemically distinct from pituitary/arcuate alpha-melanocyte stimulating hormone. Brain Res Bull, 1986. 16(1): p. 107-20.

57. Saper, C.B., et al., Direct hypothalamo-autonomic connections. Brain Res, 1976. 117(2): p. 305-12.

58. Haring, J.H. and J.N. Davis, Acetylcholinesterase neurons in the lateral hypothalamus project to the spinal cord. Brain Res, 1983. 268(2): p. 275-83.

59. Luiten, P.G. and P. Room, Interrelations between lateral, dorsomedial and ventromedial hypothalamic nuclei in the rat. An HRP study. Brain Res, 1980. 190(2): p. 321-32.

60. Hurley, K.M., et al., Efferent projections of the infralimbic cortex of the rat. J Comp Neurol, 1991. 308(2): p. 249-76.

61. Wouterlood, F.G., et al., Projection from the prefrontal cortex to histaminergic cell groups in the posterior hypothalamic region of the rat. Anterograde tracing with Phaseolus vulgaris leucoagglutinin combined with immunocytochemistry of histidine decarboxylase. Brain Res, 1987. 406(1-2): p. 330-6.

62. Sesack, S.R., et al., Topographical organization of the efferent projections of the medial prefrontal cortex in the rat: an anterograde tract-tracing study with Phaseolus vulgaris leucoagglutinin. J Comp Neurol, 1989. 290(2): p. 213-42.

63. Yasui, Y., et al., Autonomic responses and efferent pathways from the insular cortex in the rat. J Comp Neurol, 1991. 303(3): p. 355-74.

64. Usuda, I., K. Tanaka, and T. Chiba, Efferent projections of the nucleus accumbens in the rat with special reference to subdivision of the nucleus: biotinylated dextran amine study. Brain Res, 1998. 797(1): p. 73-93.

65. Mogenson, G.J., L.W. Swanson, and M. Wu, Neural projections from nucleus accumbens to globus pallidus, substantia innominata, and lateral preoptic-lateral hypothalamic area: an anatomical and electrophysiological investigation in the rat. J Neurosci, 1983. 3(1): p. 189-202.

66. Williams, D.J., A.R. Crossman, and P. Slater, The efferent projections of the nucleus accumbens in the rat. Brain Res, 1977. 130(2): p. 217-27.

67. Swanson, L.W. and W.M. Cowan, The connections of the septal region in the rat. J Comp Neurol, 1979. 186(4): p. 621-55.

68. Dong, H.W. and L.W. Swanson, Organization of axonal projections from the anterolateral area of the bed nuclei of the stria terminalis. J Comp Neurol, 2004. 468(2): p. 277-98.

69. Dong, H.W. and L.W. Swanson, Projections from bed nuclei of the stria terminalis, anteromedial area: cerebral hemisphere integration of neuroendocrine, autonomic, and behavioral aspects of energy balance. J Comp Neurol, 2006. 494(1): p. 142-78.

70. Dong, H.W. and L.W. Swanson, Projections from bed nuclei of the stria terminalis, dorsomedial nucleus: implications for cerebral hemisphere integration of neuroendocrine, autonomic, and drinking responses. J Comp Neurol, 2006. 494(1): p. 75-107.

71. Dong, H.-W. and L.W. Swanson, Projections from bed nuclei of the stria terminalis, magnocellular nucleus: Implications for cerebral hemisphere regulation of micturition, defecation, and penile erection. The Journal of Comparative Neurology, 2006. 494(1): p. 108-141.

72. Dong, H.W., et al., Basic organization of projections from the oval and fusiform nuclei of the bed nuclei of the stria terminalis in adult rat brain. J Comp Neurol, 2001. 436(4): p. 430-55.

73. Dong, H.W. and L.W. Swanson, Projections from bed nuclei of the stria terminalis, posterior division: implications for cerebral hemisphere regulation of defensive and reproductive behaviors. J Comp Neurol, 2004. 471(4): p. 396-433.

74. Shammah-Lagnado, S.J., et al., Supracapsular bed nucleus of the stria terminalis contains central and medial extended amygdala elements: evidence from anterograde and retrograde tracing experiments in the rat. J Comp Neurol, 2000. 422(4): p. 533-55.

75. Dong, H.W. and L.W. Swanson, Projections from the rhomboid nucleus of the bed nuclei of the stria terminalis: implications for cerebral hemisphere regulation of ingestive behaviors. J Comp Neurol, 2003. 463(4): p. 434-72.

76. Petrovich, G.D., P.Y. Risold, and L.W. Swanson, Organization of projections from the basomedial nucleus of the amygdala: a PHAL study in the rat. J Comp Neurol, 1996. 374(3): p. 387-420.

77. Zahm, D.S., et al., Direct comparison of projections from the central amygdaloid region and nucleus accumbens shell. Eur J Neurosci, 1999. 11(4): p. 1119-26.

78. Krettek, J.E. and J.L. Price, Amygdaloid projections to subcortical structures within the basal forebrain and brainstem in the rat and cat. J Comp Neurol, 1978. 178(2): p. 225-54.

79. Sakanaka, M., T. Shibasaki, and K. Lederis, Distribution and efferent projections of corticotropin-releasing factor-like immunoreactivity in the rat amygdaloid complex. Brain Res, 1986. 382(2): p. 213-38.

80. Dafny, N., et al., Lateral hypothalamus: site involved in pain modulation. Neuroscience, 1996. 70(2): p. 449-60.

81. Herkenham, M. and W.J. Nauta, Efferent connections of the habenular nuclei in the rat. J Comp Neurol, 1979. 187(1): p. 19-47.

82. Araki, M., P.L. McGeer, and H. Kimura, The efferent projections of the rat lateral habenular nucleus revealed by the PHA-L anterograde tracing method. Brain Res, 1988. 441(1-2): p. 319-30.

83. McIntyre, D.C., M.E. Kelly, and W.A. Staines, Efferent projections of the anterior perirhinal cortex in the rat. J Comp Neurol, 1996. 369(2): p. 302-18.

84. Canteras, N.S. and L.W. Swanson, Projections of the ventral subiculum to the amygdala, septum, and hypothalamus: a PHAL anterograde tract-tracing study in the rat. J Comp Neurol, 1992. 324(2): p. 180-94.

85. Groenewegen, H.J., et al., Organization of the projections from the subiculum to the ventral striatum in the rat. A study using anterograde transport of Phaseolus vulgaris leucoagglutinin. Neuroscience, 1987. 23(1): p. 103-20.

86. Mikkelsen, J.D., A neuronal projection from the lateral geniculate nucleus to the lateral hypothalamus of the rat demonstrated with Phaseolus vulgaris leucoagglutinin tracing. Neurosci Lett, 1990. 116(1-2): p. 58-63.

87. Vertes, R.P. and W.B. Hoover, Projections of the paraventricular and paratenial nuclei of the dorsal midline thalamus in the rat. J Comp Neurol, 2008. 508(2): p. 212-37.

88. Ohtake, T. and H. Yamada, Efferent connections of the nucleus reuniens and the rhomboid nucleus in the rat: an anterograde PHA-L tracing study. Neurosci Res, 1989. 6(6): p. 556-68.

89. Wouterlood, F.G., E. Saldana, and M.P. Witter, Projection from the nucleus reuniens thalami to the hippocampal region: light and electron microscopic tracing study in the rat with the anterograde tracer Phaseolus vulgaris-leucoagglutinin. J Comp Neurol, 1990. 296(2): p. 179-203.

90. Barone, F.C., J.T. Cheng, and M.J. Wayner, GABA inhibition of lateral hypothalamic neurons: role of reticular thalamic afferents. Brain Res Bull, 1994. 33(6): p. 699-708.

91. Ricardo, J.A., Efferent connections of the subthalamic region in the rat. I. The subthalamic nucleus of Luys. Brain Res, 1980. 202(2): p. 257-71.

92. Del-Fava, F., et al., Efferent connections of the rostral linear nucleus of the ventral tegmental area in the rat. Neuroscience, 2007. 145(3): p. 1059-76.

93. Azmitia, E.C. and M. Segal, An autoradiographic analysis of the differential ascending projections of the dorsal and median raphe nuclei in the rat. J Comp Neurol, 1978. 179(3): p. 641-67.

94. Vertes, R.P., A PHA-L analysis of ascending projections of the dorsal raphe nucleus in the rat. J Comp Neurol, 1991. 313(4): p. 643-68.

95. van de Kar, L.D. and S.A. Lorens, Differential serotonergic innervation of individual hypothalamic nuclei and other forebrain regions by the dorsal and median midbrain raphe nuclei. Brain Res, 1979. 162(1): p. 45-54.

96. Bobillier, P., et al., The efferent connections of the nucleus raphe centralis superior in the rat as revealed by radioautography. Brain Res, 1979. 166(1): p. 1-8.

97. Vertes, R.P. and G.F. Martin, Autoradiographic analysis of ascending projections from the pontine and mesencephalic reticular formation and the median raphe nucleus in the rat. J Comp Neurol, 1988. 275(4): p. 511-41.

98. Sim, L.J. and S.A. Joseph, Efferent projections of the nucleus raphe magnus. Brain Res Bull, 1992. 28(5): p. 679-82.

99. Saper, C.B. and A.D. Loewy, Efferent connections of the parabrachial nucleus in the rat. Brain Res, 1980. 197(2): p. 291-317.

100. Bester, H., J.M. Besson, and J.F. Bernard, Organization of efferent projections from the parabrachial area to the hypothalamus: a Phaseolus vulgaris-leucoagglutinin study in the rat. J Comp Neurol, 1997. 383(3): p. 245-81.

101. Ter Horst, G.J., et al., Ascending projections from the solitary tract nucleus to the hypothalamus. A Phaseolus vulgaris lectin tracing study in the rat. Neuroscience, 1989. 31(3): p. 785-97.

102. Riley, J.N., J.P. Card, and R.Y. Moore, A retinal projection to the lateral hypothalamus in the rat. Cell Tissue Res, 1981. 214(2): p. 257-69.

103. Leak, R.K. and R.Y. Moore, Identification of retinal ganglion cells projecting to the lateral hypothalamic area of the rat. Brain Res, 1997. 770(1-2): p. 105-14.

Hypothalamus - lateral preoptic nucleus (efferents and afferents)

1. Swanson, L.W., An autoradiographic study of the efferent connections of the preoptic region in the rat. J Comp Neurol, 1976. 167(2): p. 227-56.

2. Li, Y.Q., et al., The sites of origin of dopaminergic afferent fibers to the lateral habenular nucleus in the rat. J Comp Neurol, 1993. 333(1): p. 118-33.

3. Herkenham, M. and W.J. Nauta, Afferent connections of the habenular nuclei in the rat. A horseradish peroxidase study, with a note on the fiber-of-passage problem. J Comp Neurol, 1977. 173(1): p. 123-46.

4. Qu, T., et al., Demonstration of direct input from the retina to the lateral habenular nucleus in the albino rat. Brain Res, 1996. 709(2): p. 251-58.

5. Araki, M., P.L. McGeer, and E.G. McGeer, Retrograde HRP tracing combined with a pharmacohistochemical method for GABA transaminase for the identification of presumptive GABAergic projections to the habenula. Brain Res, 1984. 304(2): p. 271-7.

6. Poller, W.C., et al., A glutamatergic projection from the lateral hypothalamus targets Ventral Tegmental Area -projecting neurons in the lateral habenula of the rat. Brain Res, 2013. 1507: p. 45-60.

7. Shinoda, K. and M. Tohyama, Analysis of the habenulopetal enkephalinergic system in the rat brain: an immunohistochemical study. J Comp Neurol, 1987. 255(4): p. 483-96.

8. Kowski, A.B., et al., Differential projections from subfields in the lateral preoptic area to the lateral habenular complex of the rat. J Comp Neurol, 2008. 507(4): p. 1465-78.

9. Kim, U. and S.Y. Chang, Dendritic morphology, local circuitry, and intrinsic electrophysiology of neurons in the rat medial and lateral habenular nuclei of the epithalamus. J Comp Neurol, 2005. 483(2): p. 236-50.

10. Groenewegen, H.J., Organization of the afferent connections of the mediodorsal thalamic nucleus in the rat, related to the mediodorsal-prefrontal topography. Neuroscience, 1988. 24(2): p. 379-431.

11. Churchill, L., D.S. Zahm, and P.W. Kalivas, The mediodorsal nucleus of the thalamus in rats--I. forebrain gabaergic innervation. Neuroscience, 1996. 70(1): p. 93-102.

12. McKenna, J.T. and R.P. Vertes, Afferent projections to nucleus reuniens of the thalamus. J Comp Neurol, 2004. 480(2): p. 115-42.

13. Herkenham, M., The connections of the nucleus reuniens thalami: evidence for a direct thalamo-hippocampal pathway in the rat. J Comp Neurol, 1978. 177(4): p. 589-610.

14. Coolen, L.M., et al., Afferent connections of the parvocellular subparafascicular thalamic nucleus in the rat: evidence for functional subdivisions. J Comp Neurol, 2003. 463(2): p. 132-56.

15. Aghajanian, G.K. and R.Y. Wang, Habenular and other midbrain raphe afferents demonstrated by a modified retrograde tracing technique. Brain Res, 1977. 122(2): p. 229-42.

16. Gervasoni, D., et al., Role and origin of the GABAergic innervation of dorsal raphe serotonergic neurons. J Neurosci, 2000. 20(11): p. 4217-25.

17. Sim, L.J. and S.A. Joseph, Opiocortin and catecholamine projections to raphe nuclei. Peptides, 1989. 10(5): p. 1019-25.

18. Behzadi, G., et al., Afferents to the median raphe nucleus of the rat: retrograde cholera toxin and wheat germ conjugated horseradish peroxidase tracing, and selective D-[3H]aspartate labelling of possible excitatory amino acid inputs. Neuroscience, 1990. 37(1): p. 77-100.

19. Hermann, D.M., et al., Afferent projections to the rat nuclei raphe magnus, raphe pallidus and reticularis gigantocellularis pars alpha demonstrated by iontophoretic application of choleratoxin (subunit b). J Chem Neuroanat, 1997. 13(1): p. 1-21.

20. Geisler, S. and D.S. Zahm, Afferents of the ventral tegmental area in the rat-anatomical substratum for integrative functions. J Comp Neurol, 2005. 490(3): p. 270-94.

21. Cedarbaum, J.M. and G.K. Aghajanian, Afferent projections to the rat locus coeruleus as determined by a retrograde tracing technique. J Comp Neurol, 1978. 178(1): p. 1-16.

22. Lee, H.S., M.A. Kim, and B.D. Waterhouse, Retrograde double-labeling study of common afferent projections to the dorsal raphe and the nuclear core of the locus coeruleus in the rat. J Comp Neurol, 2005. 481(2): p. 179-93.

23. Moga, M.M., C.B. Saper, and T.S. Gray, Neuropeptide organization of the hypothalamic projection to the parabrachial nucleus in the rat. J Comp Neurol, 1990. 295(4): p. 662-82.

24. Swanson, L.W., et al., Evidence for a projection from the lateral preoptic area and substantia innominata to the 'mesencephalic locomotor region' in the rat. Brain Res, 1984. 295(1): p. 161-78.

25. Takagishi, M. and T. Chiba, Efferent projections of the infralimbic (area 25) region of the medial prefrontal cortex in the rat: an anterograde tracer PHA-L study. Brain Res, 1991. 566(1-2): p. 26-39.

26. Sesack, S.R., et al., Topographical organization of the efferent projections of the medial prefrontal cortex in the rat: an anterograde tract-tracing study with Phaseolus vulgaris leucoagglutinin. J Comp Neurol, 1989. 290(2): p. 213-42.

27. Gaykema, R.P., et al., Prefrontal cortical projections to the cholinergic neurons in the basal forebrain. J Comp Neurol, 1991. 303(4): p. 563-83.

28. Mogenson, G.J., L.W. Swanson, and M. Wu, Neural projections from nucleus accumbens to globus pallidus, substantia innominata, and lateral preoptic-lateral hypothalamic area: an anatomical and electrophysiological investigation in the rat. J Neurosci, 1983. 3(1): p. 189-202.

29. Dray, A. and N.R. Oakley, Projections from nucleus accumbens to globus pallidus and substantia nigra in the rat. Experientia, 1978. 34(1): p. 68-70.

30. Williams, D.J., A.R. Crossman, and P. Slater, The efferent projections of the nucleus accumbens in the rat. Brain Res, 1977. 130(2): p. 217-27.

31. Zahm, D.S. and L. Heimer, Specificity in the efferent projections of the nucleus accumbens in the rat: comparison of the rostral pole projection patterns with those of the core and shell. J Comp Neurol, 1993. 327(2): p. 220-32.

32. Swanson, L.W. and W.M. Cowan, The connections of the septal region in the rat. J Comp Neurol, 1979. 186(4): p. 621-55.

33. Meibach, R.C. and A. Siegel, Efferent connections of the septal area in the rat: an analysis utilizing retrograde and anterograde transport methods. Brain Res, 1977. 119(1): p. 1-20.

34. Dong, H.W. and L.W. Swanson, Organization of axonal projections from the anterolateral area of the bed nuclei of the stria terminalis. J Comp Neurol, 2004. 468(2): p. 277-98.

35. Dong, H.W. and L.W. Swanson, Projections from bed nuclei of the stria terminalis, anteromedial area: cerebral hemisphere integration of neuroendocrine, autonomic, and behavioral aspects of energy balance. J Comp Neurol, 2006. 494(1): p. 142-78.

36. Dong, H.W. and L.W. Swanson, Projections from bed nuclei of the stria terminalis, dorsomedial nucleus: implications for cerebral hemisphere integration of neuroendocrine, autonomic, and drinking responses. J Comp Neurol, 2006. 494(1): p. 75-107.

37. Prewitt, C.M. and J.P. Herman, Anatomical interactions between the central amygdaloid nucleus and the hypothalamic paraventricular nucleus of the rat: a dual tract-tracing analysis. J Chem Neuroanat, 1998. 15(3): p. 173-85.

38. Canteras, N.S., R.B. Simerly, and L.W. Swanson, Organization of projections from the medial nucleus of the amygdala: a PHAL study in the rat. J Comp Neurol, 1995. 360(2): p. 213-45.

39. Groenewegen, H.J., et al., Organization of the projections from the subiculum to the ventral striatum in the rat. A study using anterograde transport of Phaseolus vulgaris leucoagglutinin. Neuroscience, 1987. 23(1): p. 103-20.

40. Araki, M., P.L. McGeer, and H. Kimura, The efferent projections of the rat lateral habenular nucleus revealed by the PHA-L anterograde tracing method. Brain Res, 1988. 441(1-2): p. 319-30.

41. Herkenham, M. and W.J. Nauta, Efferent connections of the habenular nuclei in the rat. J Comp Neurol, 1979. 187(1): p. 19-47.

42. Ohtake, T. and H. Yamada, Efferent connections of the nucleus reuniens and the rhomboid nucleus in the rat: an anterograde PHA-L tracing study. Neurosci Res, 1989. 6(6): p. 556-68.

43. Vertes, R.P., A PHA-L analysis of ascending projections of the dorsal raphe nucleus in the rat. J Comp Neurol, 1991. 313(4): p. 643-68.

44. Vertes, R.P. and G.F. Martin, Autoradiographic analysis of ascending projections from the pontine and mesencephalic reticular formation and the median raphe nucleus in the rat. J Comp Neurol, 1988. 275(4): p. 511-41.

45. Bobillier, P., et al., The efferent connections of the nucleus raphe centralis superior in the rat as revealed by radioautography. Brain Res, 1979. 166(1): p. 1-8.

46. Saper, C.B. and A.D. Loewy, Efferent connections of the parabrachial nucleus in the rat. Brain Res, 1980. 197(2): p. 291-317.

47. Krukoff, T.L., K.H. Harris, and J.H. Jhamandas, Efferent projections from the parabrachial nucleus demonstrated with the anterograde tracer Phaseolus vulgaris leucoagglutinin. Brain Res Bull, 1993. 30(1-2): p. 163-72.

48. Bester, H., J.M. Besson, and J.F. Bernard, Organization of efferent projections from the parabrachial area to the hypothalamus: a Phaseolus vulgaris-leucoagglutinin study in the rat. J Comp Neurol, 1997. 383(3): p. 245-81.

Hypothalamus - mammillary body (efferents and afferents)

1. Gerfen, C.R. and R.M. Clavier, Neural inputs to the prefrontal agranular insular cortex in the rat: horseradish peroxidase study. Brain Res Bull, 1979. 4(3): p. 347-53.

2. Swanson, L.W., P.E. Sawchenko, and W.M. Cowan, Evidence for collateral projections by neurons in Ammon's horn, the dentate gyrus, and the subiculum: a multiple retrograde labeling study in the rat. J Neurosci, 1981. 1(5): p. 548-59.

3. Gonzalo-Ruiz, A., L. Morte, and J.M. Sanz, Glutamate/aspartate and leu-enkephalin immunoreactivity in mammillothalamic projection neurons of the rat. Brain Res Bull, 1998. 47(6): p. 565-74.

4. Seki, M. and K. Zyo, Anterior thalamic afferents from the mamillary body and the limbic cortex in the rat. J Comp Neurol, 1984. 229(2): p. 242-56.

5. Spreafico, R., G. Battaglia, and C. Frassoni, The reticular thalamic nucleus (RTN) of the rat: cytoarchitectural, Golgi, immunocytochemical, and horseradish peroxidase study. J Comp Neurol, 1991. 304(3): p. 478-90.

6. Shibata, H., Topographic organization of subcortical projections to the anterior thalamic nuclei in the rat. J Comp Neurol, 1992. 323(1): p. 117-27.

7. Peschanski, M. and J.M. Besson, Diencephalic connections of the raphe nuclei of the rat brainstem: an anatomical study with reference to the somatosensory system. J Comp Neurol, 1984. 224(4): p. 509-34.

8. Allen, G.V. and D.A. Hopkins, Convergent prefrontal cortex and mamillary body projections to the medial pontine nuclei: a light and electron microscopic study in the rat. J Comp Neurol, 1998. 398(3): p. 347-58.

9. Allen, G.V. and D.A. Hopkins, Topography and synaptology of mamillary body projections to the mesencephalon and pons in the rat. J Comp Neurol, 1990. 301(2): p. 214-31.

10. Cruce, J.A., An autoradiographic study of the descending connections of the mammillary nuclei of the rat. J Comp Neurol, 1977. 176(4): p. 631-44.

11. Saper, C.B., et al., Direct hypothalamo-autonomic connections. Brain Res, 1976. 117(2): p. 305-12.

12. Allen, G.V. and D.A. Hopkins, Mamillary body in the rat: topography and synaptology of projections from the subicular complex, prefrontal cortex, and midbrain tegmentum. J Comp Neurol, 1989. 286(3): p. 311-36.

13. Gonzalo-Ruiz, A., et al., Afferent projections to the mammillary complex of the rat, with special reference to those from surrounding hypothalamic regions. J Comp Neurol, 1992. 321(2): p. 277-99.

14. Shibata, H., Descending projections to the mammillary nuclei in the rat, as studied by retrograde and anterograde transport of wheat germ agglutinin-horseradish peroxidase. J Comp Neurol, 1989. 285(4): p. 436-52.

15. Takagishi, M. and T. Chiba, Efferent projections of the infralimbic (area 25) region of the medial prefrontal cortex in the rat: an anterograde tracer PHA-L study. Brain Res, 1991. 566(1-2): p. 26-39.

16. Wouterlood, F.G., et al., Projection from the prefrontal cortex to histaminergic cell groups in the posterior hypothalamic region of the rat. Anterograde tracing with Phaseolus vulgaris leucoagglutinin combined with immunocytochemistry of histidine decarboxylase. Brain Res, 1987. 406(1-2): p. 330-6.

17. Sesack, S.R., et al., Topographical organization of the efferent projections of the medial prefrontal cortex in the rat: an anterograde tract-tracing study with Phaseolus vulgaris leucoagglutinin. J Comp Neurol, 1989. 290(2): p. 213-42.

18. Swanson, L.W. and W.M. Cowan, The connections of the septal region in the rat. J Comp Neurol, 1979. 186(4): p. 621-55.

19. Meibach, R.C. and A. Siegel, Efferent connections of the septal area in the rat: an analysis utilizing retrograde and anterograde transport methods. Brain Res, 1977. 119(1): p. 1-20.

20. Varoqueaux, F. and P. Poulain, Projections of the mediolateral part of the lateral septum to the hypothalamus, revealed by Fos expression and axonal tracing in rats. Anat Embryol (Berl), 1999. 199(3): p. 249-63.

21. Petrovich, G.D., P.Y. Risold, and L.W. Swanson, Organization of projections from the basomedial nucleus of the amygdala: a PHAL study in the rat. J Comp Neurol, 1996. 374(3): p. 387-420.

22. Raisman, G., W.M. Cowan, and T.P. Powell, An experimental analysis of the efferent projection of the hippocampus. Brain, 1966. 89(1): p. 83-108.

23. Shibata, H., A direct projection from the entorhinal cortex to the mammillary nuclei in the rat. Neurosci Lett, 1988. 90(1-2): p. 6-10.

24. Donovan, M.K. and J.M. Wyss, Evidence for some collateralization between cortical and diencephalic efferent axons of the rat subicular cortex. Brain Res, 1983. 259(2): p. 181-92.

25. van Groen, T. and J.M. Wyss, The postsubicular cortex in the rat: characterization of the fourth region of the subicular cortex and its connections. Brain Res, 1990. 529(1-2): p. 165-77.

26. Canteras, N.S. and L.W. Swanson, Projections of the ventral subiculum to the amygdala, septum, and hypothalamus: a PHAL anterograde tract-tracing study in the rat. J Comp Neurol, 1992. 324(2): p. 180-94.

27. Groenewegen, H.J., et al., Organization of the projections from the subiculum to the ventral striatum in the rat. A study using anterograde transport of Phaseolus vulgaris leucoagglutinin. Neuroscience, 1987. 23(1): p. 103-20.

28. Witter, M.P., R.H. Ostendorf, and H.J. Groenewegen, Heterogeneity in the Dorsal Subiculum of the Rat. Distinct Neuronal Zones Project to Different Cortical and Subcortical Targets. Eur J Neurosci, 1990. 2(8): p. 718-725.

29. Handelmann, G.E., et al., Extra-hippocampal projections of CCK neurons of the hippocampus and subiculum. Peptides, 1983. 4(3): p. 331-4.

30. Swanson, L.W. and W.M. Cowan, Hippocampo-hypothalamic connections: origin in subicular cortex, not ammon's horn. Science, 1975. 189(4199): p. 303-4.

31. Azmitia, E.C. and M. Segal, An autoradiographic analysis of the differential ascending projections of the dorsal and median raphe nuclei in the rat. J Comp Neurol, 1978. 179(3): p. 641-67.

32. Shibata, H., Ascending projections to the mammillary nuclei in the rat: a study using retrograde and anterograde transport of wheat germ agglutinin conjugated to horseradish peroxidase. J Comp Neurol, 1987. 264(2): p. 205-15.

33. Vertes, R.P. and G.F. Martin, Autoradiographic analysis of ascending projections from the pontine and mesencephalic reticular formation and the median raphe nucleus in the rat. J Comp Neurol, 1988. 275(4): p. 511-41.

34. Vertes, R.P., W.J. Fortin, and A.M. Crane, Projections of the median raphe nucleus in the rat. J Comp Neurol, 1999. 407(4): p. 555-82.

35. Loizou, L.A., Projections of the nucleus locus coeruleus in the albino rat. Brain Res, 1969. 15(2): p. 563-6.

Hypothalamus - periventricular hypothalamic nucleus (efferents and afferents)

1. Aarnisalo, A.A. and P. Panula, Neuropeptide FF-containing efferent projections from the medial hypothalamus of rat: a Phaseolus vulgaris leucoagglutinin study. Neuroscience, 1995. 65(1): p. 175-92.

2. Gu, G.B. and R.B. Simerly, Projections of the sexually dimorphic anteroventral periventricular nucleus in the female rat. J Comp Neurol, 1997. 384(1): p. 142-64.

3. Segal, M. and S.C. Landis, Afferents to the septal area of the rat studied with the method of retrograde axonal transport of horseradish peroxidase. Brain Res, 1974. 82(2): p. 263-8.

4. Renaud, L.P. and D.A. Hopkins, Amygdala afferents from the mediobasal hypothalamus: an electrophysiological and neuroanatomical study in the rat. Brain Res, 1977. 121(2): p. 201-13.

5. Canteras, N.S., R.B. Simerly, and L.W. Swanson, Connections of the posterior nucleus of the amygdala. J Comp Neurol, 1992. 324(2): p. 143-79.

6. Li, Y.Q., et al., The sites of origin of dopaminergic afferent fibers to the lateral habenular nucleus in the rat. J Comp Neurol, 1993. 333(1): p. 118-33.

7. Cornwall, J. and O.T. Phillipson, Afferent projections to the dorsal thalamus of the rat as shown by retrograde lectin transport. II. The midline nuclei. Brain Res Bull, 1988. 21(2): p. 147-61.

8. Cornwall, J. and O.T. Phillipson, Afferent projections to the parafascicular thalamic nucleus of the rat, as shown by the retrograde transport of wheat germ agglutinin. Brain Res Bull, 1988. 20(2): p. 139-50.

9. Arluison, M. and P. Derer, Forebrain connections of the rat paraventricular thalamic nucleus as demonstrated using the carbocyanide dye DiI. Neurobiology (Bp), 1993. 1(4): p. 337-50.

10. McKenna, J.T. and R.P. Vertes, Afferent projections to nucleus reuniens of the thalamus. J Comp Neurol, 2004. 480(2): p. 115-42.

11. Herkenham, M., The connections of the nucleus reuniens thalami: evidence for a direct thalamo-hippocampal pathway in the rat. J Comp Neurol, 1978. 177(4): p. 589-610.

12. Coolen, L.M., et al., Afferent connections of the parvocellular subparafascicular thalamic nucleus in the rat: evidence for functional subdivisions. J Comp Neurol, 2003. 463(2): p. 132-56.

13. Herkenham, M., The afferent and efferent connections of the ventromedial thalamic nucleus in the rat. J Comp Neurol, 1979. 183(3): p. 487-517.

14. Greenwell, T.N., et al., Endomorphin-1 and -2 immunoreactive cells in the hypothalamus are labeled by fluoro-gold injections to the ventral tegmental area. J Comp Neurol, 2002. 454(3): p. 320-8.

15. Hermann, D.M., et al., Afferent projections to the rat nuclei raphe magnus, raphe pallidus and reticularis gigantocellularis pars alpha demonstrated by iontophoretic application of choleratoxin (subunit b). J Chem Neuroanat, 1997. 13(1): p. 1-21.

16. Moga, M.M., C.B. Saper, and T.S. Gray, Neuropeptide organization of the hypothalamic projection to the parabrachial nucleus in the rat. J Comp Neurol, 1990. 295(4): p. 662-82.

17. Meibach, R.C. and A. Siegel, Efferent connections of the septal area in the rat: an analysis utilizing retrograde and anterograde transport methods. Brain Res, 1977. 119(1): p. 1-20.

18. Dong, H.W. and L.W. Swanson, Projections from bed nuclei of the stria terminalis, anteromedial area: cerebral hemisphere integration of neuroendocrine, autonomic, and behavioral aspects of energy balance. J Comp Neurol, 2006. 494(1): p. 142-78.

19. Dong, H.W. and L.W. Swanson, Projections from bed nuclei of the stria terminalis, dorsomedial nucleus: implications for cerebral hemisphere integration of neuroendocrine, autonomic, and drinking responses. J Comp Neurol, 2006. 494(1): p. 75-107.

20. Dong, H.W., et al., Basic organization of projections from the oval and fusiform nuclei of the bed nuclei of the stria terminalis in adult rat brain. J Comp Neurol, 2001. 436(4): p. 430-55.

21. Dong, H.W. and L.W. Swanson, Projections from bed nuclei of the stria terminalis, posterior division: implications for cerebral hemisphere regulation of defensive and reproductive behaviors. J Comp Neurol, 2004. 471(4): p. 396-433.

22. Dong, H.-W. and L.W. Swanson, Projections from bed nuclei of the stria terminalis, magnocellular nucleus: Implications for cerebral hemisphere regulation of micturition, defecation, and penile erection. The Journal of Comparative Neurology, 2006. 494(1): p. 108-141.

23. Hutton, L.A., G. Gu, and R.B. Simerly, Development of a sexually dimorphic projection from the bed nuclei of the stria terminalis to the anteroventral periventricular nucleus in the rat. J Neurosci, 1998. 18(8): p. 3003-13.

24. Gu, G., A. Cornea, and R.B. Simerly, Sexual differentiation of projections from the principal nucleus of the bed nuclei of the stria terminalis. J Comp Neurol, 2003. 460(4): p. 542-62.

25. Polston, E.K., G. Gu, and R.B. Simerly, Neurons in the principal nucleus of the bed nuclei of the stria terminalis provide a sexually dimorphic GABAergic input to the anteroventral periventricular nucleus of the hypothalamus. Neuroscience, 2004. 123(3): p. 793-803.

26. Dong, H.W. and L.W. Swanson, Projections from the rhomboid nucleus of the bed nuclei of the stria terminalis: implications for cerebral hemisphere regulation of ingestive behaviors. J Comp Neurol, 2003. 463(4): p. 434-72.

27. Canteras, N.S., R.B. Simerly, and L.W. Swanson, Organization of projections from the medial nucleus of the amygdala: a PHAL study in the rat. J Comp Neurol, 1995. 360(2): p. 213-45.

28. Prewitt, C.M. and J.P. Herman, Anatomical interactions between the central amygdaloid nucleus and the hypothalamic paraventricular nucleus of the rat: a dual tract-tracing analysis. J Chem Neuroanat, 1998. 15(3): p. 173-85.

29. Moga, M.M. and R.Y. Moore, Organization of neural inputs to the suprachiasmatic nucleus in the rat. J Comp Neurol, 1997. 389(3): p. 508-34.

30. Canteras, N.S. and L.W. Swanson, Projections of the ventral subiculum to the amygdala, septum, and hypothalamus: a PHAL anterograde tract-tracing study in the rat. J Comp Neurol, 1992. 324(2): p. 180-94.

31. Kishi, T., et al., Topographical organization of projections from the subiculum to the hypothalamus in the rat. J Comp Neurol, 2000. 419(2): p. 205-22.

32. Azmitia, E.C. and M. Segal, An autoradiographic analysis of the differential ascending projections of the dorsal and median raphe nuclei in the rat. J Comp Neurol, 1978. 179(3): p. 641-67.

33. Bobillier, P., et al., The efferent connections of the nucleus raphe centralis superior in the rat as revealed by radioautography. Brain Res, 1979. 166(1): p. 1-8.

34. Loizou, L.A., Projections of the nucleus locus coeruleus in the albino rat. Brain Res, 1969. 15(2): p. 563-6.

35. Bester, H., J.M. Besson, and J.F. Bernard, Organization of efferent projections from the parabrachial area to the hypothalamus: a Phaseolus vulgaris-leucoagglutinin study in the rat. J Comp Neurol, 1997. 383(3): p. 245-81.

36. Alden, M., J.M. Besson, and J.F. Bernard, Organization of the efferent projections from the pontine parabrachial area to the bed nucleus of the stria terminalis and neighboring regions: a PHA-L study in the rat. J Comp Neurol, 1994. 341(3): p. 289-314.

Hypothalamus - posterior nucleus of hypothalamus (efferents and afferents)

1. de Olmos, J., H. Hardy, and L. Heimer, The afferent connections of the main and the accessory olfactory bulb formations in the rat: an experimental HRP-study. J Comp Neurol, 1978. 181(2): p. 213-44.

2. Vertes, R.P., et al., Ascending projections of the posterior nucleus of the hypothalamus: PHA-L analysis in the rat. J Comp Neurol, 1995. 359(1): p. 90-116.

3. Shin, J.W., J.C. Geerling, and A.D. Loewy, Inputs to the ventrolateral bed nucleus of the stria terminalis. J Comp Neurol, 2008. 511(5): p. 628-57.

4. Li, Y.Q., et al., The sites of origin of dopaminergic afferent fibers to the lateral habenular nucleus in the rat. J Comp Neurol, 1993. 333(1): p. 118-33.

5. Sakanaka, M., et al., Topographic organization of the projection from the forebrain subcortical areas to the hippocampal formation of the rat. Neurosci Lett, 1980. 20(3): p. 253-7.

6. Cornwall, J. and O.T. Phillipson, Afferent projections to the dorsal thalamus of the rat as shown by retrograde lectin transport. II. The midline nuclei. Brain Res Bull, 1988. 21(2): p. 147-61.

7. Herkenham, M., The connections of the nucleus reuniens thalami: evidence for a direct thalamo-hippocampal pathway in the rat. J Comp Neurol, 1978. 177(4): p. 589-610.

8. McKenna, J.T. and R.P. Vertes, Afferent projections to nucleus reuniens of the thalamus. J Comp Neurol, 2004. 480(2): p. 115-42.

9. Geisler, S. and D.S. Zahm, Afferents of the ventral tegmental area in the rat-anatomical substratum for integrative functions. J Comp Neurol, 2005. 490(3): p. 270-94.

10. Peschanski, M. and J.M. Besson, Diencephalic connections of the raphe nuclei of the rat brainstem: an anatomical study with reference to the somatosensory system. J Comp Neurol, 1984. 224(4): p. 509-34.

11. Sim, L.J. and S.A. Joseph, Opiocortin and catecholamine projections to raphe nuclei. Peptides, 1989. 10(5): p. 1019-25.

12. Gervasoni, D., et al., Role and origin of the GABAergic innervation of dorsal raphe serotonergic neurons. J Neurosci, 2000. 20(11): p. 4217-25.

13. Luppi, P.H., et al., Afferent projections to the rat locus coeruleus demonstrated by retrograde and anterograde tracing with cholera-toxin B subunit and Phaseolus vulgaris leucoagglutinin. Neuroscience, 1995. 65(1): p. 119-60.

14. Abrahamson, E.E. and R.Y. Moore, The posterior hypothalamic area: chemoarchitecture and afferent connections. Brain Res, 2001. 889(1-2): p. 1-22.

15. Takagishi, M. and T. Chiba, Efferent projections of the infralimbic (area 25) region of the medial prefrontal cortex in the rat: an anterograde tracer PHA-L study. Brain Res, 1991. 566(1-2): p. 26-39.

16. Sesack, S.R., et al., Topographical organization of the efferent projections of the medial prefrontal cortex in the rat: an anterograde tract-tracing study with Phaseolus vulgaris leucoagglutinin. J Comp Neurol, 1989. 290(2): p. 213-42.

17. Dong, H.W. and L.W. Swanson, Organization of axonal projections from the anterolateral area of the bed nuclei of the stria terminalis. J Comp Neurol, 2004. 468(2): p. 277-98.

18. Dong, H.W. and L.W. Swanson, Projections from bed nuclei of the stria terminalis, dorsomedial nucleus: implications for cerebral hemisphere integration of neuroendocrine, autonomic, and drinking responses. J Comp Neurol, 2006. 494(1): p. 75-107.

19. Dong, H.-W. and L.W. Swanson, Projections from bed nuclei of the stria terminalis, magnocellular nucleus: Implications for cerebral hemisphere regulation of micturition, defecation, and penile erection. The Journal of Comparative Neurology, 2006. 494(1): p. 108-141.

20. Dong, H.W. and L.W. Swanson, Projections from bed nuclei of the stria terminalis, anteromedial area: cerebral hemisphere integration of neuroendocrine, autonomic, and behavioral aspects of energy balance. J Comp Neurol, 2006. 494(1): p. 142-78.

21. Dong, H.W. and L.W. Swanson, Projections from bed nuclei of the stria terminalis, posterior division: implications for cerebral hemisphere regulation of defensive and reproductive behaviors. J Comp Neurol, 2004. 471(4): p. 396-433.

22. Bourgeais, L., C. Gauriau, and J.F. Bernard, Projections from the nociceptive area of the central nucleus of the amygdala to the forebrain: a PHA-L study in the rat. Eur J Neurosci, 2001. 14(2): p. 229-55.

23. Chiba, T., Collateral projection from the amygdalo--hippocampal transition area and CA1 to the hypothalamus and medial prefrontal cortex in the rat. Neurosci Res, 2000. 38(4): p. 373-83.

24. Canteras, N.S. and L.W. Swanson, Projections of the ventral subiculum to the amygdala, septum, and hypothalamus: a PHAL anterograde tract-tracing study in the rat. J Comp Neurol, 1992. 324(2): p. 180-94.

25. Kishi, T., et al., Topographical organization of projections from the subiculum to the hypothalamus in the rat. J Comp Neurol, 2000. 419(2): p. 205-22.

26. Beckstead, R.M., V.B. Domesick, and W.J. Nauta, Efferent connections of the substantia nigra and ventral tegmental area in the rat. Brain Res, 1979. 175(2): p. 191-217.

27. Azmitia, E.C. and M. Segal, An autoradiographic analysis of the differential ascending projections of the dorsal and median raphe nuclei in the rat. J Comp Neurol, 1978. 179(3): p. 641-67.

28. Bobillier, P., et al., The efferent connections of the nucleus raphe centralis superior in the rat as revealed by radioautography. Brain Res, 1979. 166(1): p. 1-8.

29. Vertes, R.P. and G.F. Martin, Autoradiographic analysis of ascending projections from the pontine and mesencephalic reticular formation and the median raphe nucleus in the rat. J Comp Neurol, 1988. 275(4): p. 511-41.

30. Loizou, L.A., Projections of the nucleus locus coeruleus in the albino rat. Brain Res, 1969. 15(2): p. 563-6.

Hypothalamus - premammillary nuclei (ventral and dorsal) (efferents and afferents)

1. de Olmos, J., H. Hardy, and L. Heimer, The afferent connections of the main and the accessory olfactory bulb formations in the rat: an experimental HRP-study. J Comp Neurol, 1978. 181(2): p. 213-44.

2. Sarter, M. and H.J. Markowitsch, Collateral innervation of the medial and lateral prefrontal cortex by amygdaloid, thalamic, and brain-stem neurons. J Comp Neurol, 1984. 224(3): p. 445-60.

3. Canteras, N.S., R.B. Simerly, and L.W. Swanson, Projections of the ventral premammillary nucleus. J Comp Neurol, 1992. 324(2): p. 195-212.

4. Ottersen, O.P., Afferent connections to the amygdaloid complex of the rat and cat: II. Afferents from the hypothalamus and the basal telencephalon. J Comp Neurol, 1980. 194(1): p. 267-89.

5. Veening, J.G., Subcortical afferents of the amygdaloid complex in the rat: an HRP study. Neurosci Lett, 1978. 8(3): p. 197-202.

6. Canteras, N.S., R.B. Simerly, and L.W. Swanson, Connections of the posterior nucleus of the amygdala. J Comp Neurol, 1992. 324(2): p. 143-79.

7. Canteras, N.S. and L.W. Swanson, The dorsal premammillary nucleus: an unusual component of the mammillary body. Proc Natl Acad Sci U S A, 1992. 89(21): p. 10089-93.

8. Herkenham, M., The connections of the nucleus reuniens thalami: evidence for a direct thalamo-hippocampal pathway in the rat. J Comp Neurol, 1978. 177(4): p. 589-610.

9. McKenna, J.T. and R.P. Vertes, Afferent projections to nucleus reuniens of the thalamus. J Comp Neurol, 2004. 480(2): p. 115-42.

10. Geisler, S. and D.S. Zahm, Afferents of the ventral tegmental area in the rat-anatomical substratum for integrative functions. J Comp Neurol, 2005. 490(3): p. 270-94.

11. Comoli, E., E.R. Ribeiro-Barbosa, and N.S. Canteras, Afferent connections of the dorsal premammillary nucleus. J Comp Neurol, 2000. 423(1): p. 83-98.

12. Sesack, S.R., et al., Topographical organization of the efferent projections of the medial prefrontal cortex in the rat: an anterograde tract-tracing study with Phaseolus vulgaris leucoagglutinin. J Comp Neurol, 1989. 290(2): p. 213-42.

13. Dong, H.W. and L.W. Swanson, Projections from bed nuclei of the stria terminalis, posterior division: implications for cerebral hemisphere regulation of defensive and reproductive behaviors. J Comp Neurol, 2004. 471(4): p. 396-433.

14. Dong, H.-W. and L.W. Swanson, Projections from bed nuclei of the stria terminalis, magnocellular nucleus: Implications for cerebral hemisphere regulation of micturition, defecation, and penile erection. The Journal of Comparative Neurology, 2006. 494(1): p. 108-141.

15. Krettek, J.E. and J.L. Price, Amygdaloid projections to subcortical structures within the basal forebrain and brainstem in the rat and cat. J Comp Neurol, 1978. 178(2): p. 225-54.

16. Canteras, N.S., R.B. Simerly, and L.W. Swanson, Organization of projections from the medial nucleus of the amygdala: a PHAL study in the rat. J Comp Neurol, 1995. 360(2): p. 213-45.

17. Chiba, T., Collateral projection from the amygdalo--hippocampal transition area and CA1 to the hypothalamus and medial prefrontal cortex in the rat. Neurosci Res, 2000. 38(4): p. 373-83.

18. McIntyre, D.C., M.E. Kelly, and W.A. Staines, Efferent projections of the anterior perirhinal cortex in the rat. J Comp Neurol, 1996. 369(2): p. 302-18.

19. Canteras, N.S. and L.W. Swanson, Projections of the ventral subiculum to the amygdala, septum, and hypothalamus: a PHAL anterograde tract-tracing study in the rat. J Comp Neurol, 1992. 324(2): p. 180-94.

20. Kishi, T., et al., Topographical organization of projections from the subiculum to the hypothalamus in the rat. J Comp Neurol, 2000. 419(2): p. 205-22.

21. Loizou, L.A., Projections of the nucleus locus coeruleus in the albino rat. Brain Res, 1969. 15(2): p. 563-6.

Hypothalamus - suprachiasmatic nucleus (efferents and afferents)

1. Sylvester, C.M., K.E. Krout, and A.D. Loewy, Suprachiasmatic nucleus projection to the medial prefrontal cortex: a viral transneuronal tracing study. Neuroscience, 2002. 114(4): p. 1071-80.

2. Watts, A.G. and L.W. Swanson, Efferent projections of the suprachiasmatic nucleus: II. Studies using retrograde transport of fluorescent dyes and simultaneous peptide immunohistochemistry in the rat. J Comp Neurol, 1987. 258(2): p. 230-52.

3. Leak, R.K. and R.Y. Moore, Topographic organization of suprachiasmatic nucleus projection neurons. J Comp Neurol, 2001. 433(3): p. 312-34.

4. Watts, A.G., L.W. Swanson, and G. Sanchez-Watts, Efferent projections of the suprachiasmatic nucleus: I. Studies using anterograde transport of Phaseolus vulgaris leucoagglutinin in the rat. J Comp Neurol, 1987. 258(2): p. 204-29.

5. Chen, S. and H.S. Su, Afferent connections of the thalamic paraventricular and parataenial nuclei in the rat--a retrograde tracing study with iontophoretic application of Fluoro-Gold. Brain Res, 1990. 522(1): p. 1-6.

6. Otake, K., D.A. Ruggiero, and Y. Nakamura, Adrenergic innervation of forebrain neurons that project to the paraventricular thalamic nucleus in the rat. Brain Res, 1995. 697(1-2): p. 17-26.

7. Sim, L.J. and S.A. Joseph, Opiocortin and catecholamine projections to raphe nuclei. Peptides, 1989. 10(5): p. 1019-25.

8. Swanson, L.W. and W.M. Cowan, The efferent connections of the suprachiasmatic nucleus of the hypothalamus. J Comp Neurol, 1975. 160(1): p. 1-12.

9. Moga, M.M. and R.Y. Moore, Organization of neural inputs to the suprachiasmatic nucleus in the rat. J Comp Neurol, 1997. 389(3): p. 508-34.

10. Hurley, K.M., et al., Efferent projections of the infralimbic cortex of the rat. J Comp Neurol, 1991. 308(2): p. 249-76.

11. Staiger, J.F. and F.G. Wouterlood, Efferent projections from the lateral septal nucleus to the anterior hypothalamus in the rat: a study combining Phaseolus vulgaris-leucoagglutinin tracing with vasopressin immunocytochemistry. Cell Tissue Res, 1990. 261(1): p. 17-23.

12. Dong, H.W. and L.W. Swanson, Projections from bed nuclei of the stria terminalis, dorsomedial nucleus: implications for cerebral hemisphere integration of neuroendocrine, autonomic, and drinking responses. J Comp Neurol, 2006. 494(1): p. 75-107.

13. Dong, H.-W. and L.W. Swanson, Projections from bed nuclei of the stria terminalis, magnocellular nucleus: Implications for cerebral hemisphere regulation of micturition, defecation, and penile erection. The Journal of Comparative Neurology, 2006. 494(1): p. 108-141.

14. Dong, H.W. and L.W. Swanson, Projections from bed nuclei of the stria terminalis, anteromedial area: cerebral hemisphere integration of neuroendocrine, autonomic, and behavioral aspects of energy balance. J Comp Neurol, 2006. 494(1): p. 142-78.

15. Canteras, N.S., R.B. Simerly, and L.W. Swanson, Organization of projections from the medial nucleus of the amygdala: a PHAL study in the rat. J Comp Neurol, 1995. 360(2): p. 213-45.

16. Raisman, G., W.M. Cowan, and T.P. Powell, An experimental analysis of the efferent projection of the hippocampus. Brain, 1966. 89(1): p. 83-108.

17. Kishi, T., et al., Topographical organization of projections from the subiculum to the hypothalamus in the rat. J Comp Neurol, 2000. 419(2): p. 205-22.

18. Canteras, N.S. and L.W. Swanson, Projections of the ventral subiculum to the amygdala, septum, and hypothalamus: a PHAL anterograde tract-tracing study in the rat. J Comp Neurol, 1992. 324(2): p. 180-94.

19. Ribak, C.E. and A. Peters, An autoradiographic study of the projections from the lateral geniculate body of the rat. Brain Res, 1975. 92(3): p. 341-68.

20. Alamilla, J. and R. Aguilar-Roblero, Glutamate and GABA neurotransmission from the paraventricular thalamus to the suprachiasmatic nuclei in the rat. J Biol Rhythms, 2010. 25(1): p. 28-36.

21. Moga, M.M., R.P. Weis, and R.Y. Moore, Efferent projections of the paraventricular thalamic nucleus in the rat. J Comp Neurol, 1995. 359(2): p. 221-38.

22. Herkenham, M., The connections of the nucleus reuniens thalami: evidence for a direct thalamo-hippocampal pathway in the rat. J Comp Neurol, 1978. 177(4): p. 589-610.

23. Azmitia, E.C. and M. Segal, An autoradiographic analysis of the differential ascending projections of the dorsal and median raphe nuclei in the rat. J Comp Neurol, 1978. 179(3): p. 641-67.

24. Hay-Schmidt, A., et al., Projections from the raphe nuclei to the suprachiasmatic nucleus of the rat. Journal of Chemical Neuroanatomy, 2003. 25(4): p. 293-310.

25. Bobillier, P., et al., The efferent connections of the nucleus raphe centralis superior in the rat as revealed by radioautography. Brain Res, 1979. 166(1): p. 1-8.

26. Vertes, R.P. and G.F. Martin, Autoradiographic analysis of ascending projections from the pontine and mesencephalic reticular formation and the median raphe nucleus in the rat. J Comp Neurol, 1988. 275(4): p. 511-41.

27. van de Kar, L.D. and S.A. Lorens, Differential serotonergic innervation of individual hypothalamic nuclei and other forebrain regions by the dorsal and median midbrain raphe nuclei. Brain Res, 1979. 162(1): p. 45-54.

28. Ter Horst, G.J., et al., Ascending projections from the solitary tract nucleus to the hypothalamus. A Phaseolus vulgaris lectin tracing study in the rat. Neuroscience, 1989. 31(3): p. 785-97.

29. Moore, R.Y. and N.J. Lenn, A retinohypothalamic projection in the rat. J Comp Neurol, 1972. 146(1): p. 1-14.

Hypothalamus - supraoptic nucleus (efferents and afferents)

1. Sim, L.J. and S.A. Joseph, Opiocortin and catecholamine projections to raphe nuclei. Peptides, 1989. 10(5): p. 1019-25.

2. Smithson, K.G., M.L. Weiss, and G.I. Hatton, Supraoptic nucleus afferents from the accessory olfactory bulb: evidence from anterograde and retrograde tract tracing in the rat. Brain Res Bull, 1992. 29(2): p. 209-20.

3. Smithson, K.G., M.L. Weiss, and G.I. Hatton, Supraoptic nucleus afferents from the main olfactory bulb--I. Anatomical evidence from anterograde and retrograde tracers in rat. Neuroscience, 1989. 31(2): p. 277-87.

4. Jhamandas, J.H., et al., Diagonal band projection towards the hypothalamic supraoptic nucleus: light and electron microscopic observations in the rat. J Comp Neurol, 1989. 282(1): p. 15-23.

5. Staiger, J.F. and F.G. Wouterlood, Efferent projections from the lateral septal nucleus to the anterior hypothalamus in the rat: a study combining Phaseolus vulgaris-leucoagglutinin tracing with vasopressin immunocytochemistry. Cell Tissue Res, 1990. 261(1): p. 17-23.

6. Dong, H.W. and L.W. Swanson, Organization of axonal projections from the anterolateral area of the bed nuclei of the stria terminalis. J Comp Neurol, 2004. 468(2): p. 277-98.

7. Dong, H.W. and L.W. Swanson, Projections from bed nuclei of the stria terminalis, anteromedial area: cerebral hemisphere integration of neuroendocrine, autonomic, and behavioral aspects of energy balance. J Comp Neurol, 2006. 494(1): p. 142-78.

8. Dong, H.W. and L.W. Swanson, Projections from bed nuclei of the stria terminalis, dorsomedial nucleus: implications for cerebral hemisphere integration of neuroendocrine, autonomic, and drinking responses. J Comp Neurol, 2006. 494(1): p. 75-107.

9. Dong, H.-W. and L.W. Swanson, Projections from bed nuclei of the stria terminalis, magnocellular nucleus: Implications for cerebral hemisphere regulation of micturition, defecation, and penile erection. The Journal of Comparative Neurology, 2006. 494(1): p. 108-141.

10. Canteras, N.S. and L.W. Swanson, Projections of the ventral subiculum to the amygdala, septum, and hypothalamus: a PHAL anterograde tract-tracing study in the rat. J Comp Neurol, 1992. 324(2): p. 180-94.

11. Iijima, K. and T. Ogawa, Demonstration of projections from locus coeruleus to supraoptic nucleus by the HRP method with special reference to cell types in the rat. Arch Histol Jpn, 1980. 43(5): p. 411-21.

12. Jhamandas, J.H., K.H. Harris, and T.L. Krukoff, Parabrachial nucleus projection towards the hypothalamic supraoptic nucleus: electrophysiological and anatomical observations in the rat. J Comp Neurol, 1991. 308(1): p. 42-50.

13. Krukoff, T.L., K.H. Harris, and J.H. Jhamandas, Efferent projections from the parabrachial nucleus demonstrated with the anterograde tracer Phaseolus vulgaris leucoagglutinin. Brain Res Bull, 1993. 30(1-2): p. 163-72.

14. Day, T.A. and J.R. Sibbald, Direct catecholaminergic projection from nucleus tractus solitarii to supraoptic nucleus. Brain Res, 1988. 454(1-2): p. 387-92.

Hypothalamus - arcuate nucleus (efferents and afferents)

1. Sim, L.J. and S.A. Joseph, Arcuate nucleus projections to brainstem regions which modulate nociception. J Chem Neuroanat, 1991. 4(2): p. 97-109.

2. Renaud, L.P. and D.A. Hopkins, Amygdala afferents from the mediobasal hypothalamus: an electrophysiological and neuroanatomical study in the rat. Brain Res, 1977. 121(2): p. 201-13.

3. Ottersen, O.P., Afferent connections to the amygdaloid complex of the rat and cat: II. Afferents from the hypothalamus and the basal telencephalon. J Comp Neurol, 1980. 194(1): p. 267-89.

4. Cornwall, J. and O.T. Phillipson, Afferent projections to the dorsal thalamus of the rat as shown by retrograde lectin transport. II. The midline nuclei. Brain Res Bull, 1988. 21(2): p. 147-61.

5. Otake, K., D.A. Ruggiero, and Y. Nakamura, Adrenergic innervation of forebrain neurons that project to the paraventricular thalamic nucleus in the rat. Brain Res, 1995. 697(1-2): p. 17-26.

6. McKenna, J.T. and R.P. Vertes, Afferent projections to nucleus reuniens of the thalamus. J Comp Neurol, 2004. 480(2): p. 115-42.

7. Greenwell, T.N., et al., Endomorphin-1 and -2 immunoreactive cells in the hypothalamus are labeled by fluoro-gold injections to the ventral tegmental area. J Comp Neurol, 2002. 454(3): p. 320-8.

8. Yoon, Y.S., J.S. Lee, and H.S. Lee, Retrograde study of CART- or NPY-neuronal projection from the hypothalamic arcuate nucleus to the dorsal raphe and/or the locus coeruleus in the rat. Brain Res, 2013. 1519: p. 40-52.

9. Lee, H.S., et al., Glutamatergic afferent projections to the dorsal raphe nucleus of the rat. Brain Research, 2003. 963(1-2): p. 57-71.

10. Sim, L.J. and S.A. Joseph, Opiocortin and catecholamine projections to raphe nuclei. Peptides, 1989. 10(5): p. 1019-25.

11. Luppi, P.H., et al., Afferent projections to the rat locus coeruleus demonstrated by retrograde and anterograde tracing with cholera-toxin B subunit and Phaseolus vulgaris leucoagglutinin. Neuroscience, 1995. 65(1): p. 119-60.

12. Lee, H.S., M.A. Kim, and B.D. Waterhouse, Retrograde double-labeling study of common afferent projections to the dorsal raphe and the nuclear core of the locus coeruleus in the rat. J Comp Neurol, 2005. 481(2): p. 179-93.

13. Magoul, R., et al., Direct and indirect enkephalinergic synaptic inputs to the rat arcuate nucleus studied by combination of retrograde tracing and immunocytochemistry. Neuroscience, 1993. 55(4): p. 1055-66.

14. Dong, H.W. and L.W. Swanson, Projections from bed nuclei of the stria terminalis, anteromedial area: cerebral hemisphere integration of neuroendocrine, autonomic, and behavioral aspects of energy balance. J Comp Neurol, 2006. 494(1): p. 142-78.

15. Dong, H.W. and L.W. Swanson, Projections from bed nuclei of the stria terminalis, dorsomedial nucleus: implications for cerebral hemisphere integration of neuroendocrine, autonomic, and drinking responses. J Comp Neurol, 2006. 494(1): p. 75-107.

16. Dong, H.-W. and L.W. Swanson, Projections from bed nuclei of the stria terminalis, magnocellular nucleus: Implications for cerebral hemisphere regulation of micturition, defecation, and penile erection. The Journal of Comparative Neurology, 2006. 494(1): p. 108-141.

17. Gu, G., A. Cornea, and R.B. Simerly, Sexual differentiation of projections from the principal nucleus of the bed nuclei of the stria terminalis. J Comp Neurol, 2003. 460(4): p. 542-62.

18. Dong, H.W. and L.W. Swanson, Projections from bed nuclei of the stria terminalis, posterior division: implications for cerebral hemisphere regulation of defensive and reproductive behaviors. J Comp Neurol, 2004. 471(4): p. 396-433.

19. Canteras, N.S., R.B. Simerly, and L.W. Swanson, Organization of projections from the medial nucleus of the amygdala: a PHAL study in the rat. J Comp Neurol, 1995. 360(2): p. 213-45.

20. Kishi, T., et al., Topographical organization of projections from the subiculum to the hypothalamus in the rat. J Comp Neurol, 2000. 419(2): p. 205-22.

21. Moga, M.M. and R.Y. Moore, Organization of neural inputs to the suprachiasmatic nucleus in the rat. J Comp Neurol, 1997. 389(3): p. 508-34.

22. Canteras, N.S. and L.W. Swanson, Projections of the ventral subiculum to the amygdala, septum, and hypothalamus: a PHAL anterograde tract-tracing study in the rat. J Comp Neurol, 1992. 324(2): p. 180-94.

23. Handelmann, G.E., et al., Extra-hippocampal projections of CCK neurons of the hippocampus and subiculum. Peptides, 1983. 4(3): p. 331-4.

24. Vertes, R.P. and W.B. Hoover, Projections of the paraventricular and paratenial nuclei of the dorsal midline thalamus in the rat. J Comp Neurol, 2008. 508(2): p. 212-37.

25. Azmitia, E.C. and M. Segal, An autoradiographic analysis of the differential ascending projections of the dorsal and median raphe nuclei in the rat. J Comp Neurol, 1978. 179(3): p. 641-67.

26. van de Kar, L.D. and S.A. Lorens, Differential serotonergic innervation of individual hypothalamic nuclei and other forebrain regions by the dorsal and median midbrain raphe nuclei. Brain Res, 1979. 162(1): p. 45-54.

27. Loizou, L.A., Projections of the nucleus locus coeruleus in the albino rat. Brain Res, 1969. 15(2): p. 563-6.

28. Saper, C.B. and A.D. Loewy, Efferent connections of the parabrachial nucleus in the rat. Brain Res, 1980. 197(2): p. 291-317.

29. Ter Horst, G.J., et al., Ascending projections from the solitary tract nucleus to the hypothalamus. A Phaseolus vulgaris lectin tracing study in the rat. Neuroscience, 1989. 31(3): p. 785-97.

Hypothalamus - dorsomedial nucleus of hypothalamus (efferents and afferents)

1. Aarnisalo, A.A. and P. Panula, Neuropeptide FF-containing efferent projections from the medial hypothalamus of rat: a Phaseolus vulgaris leucoagglutinin study. Neuroscience, 1995. 65(1): p. 175-92.

2. ter Horst, G.J. and P.G. Luiten, The projections of the dorsomedial hypothalamic nucleus in the rat. Brain Res Bull, 1986. 16(2): p. 231-48.

3. Shin, J.W., J.C. Geerling, and A.D. Loewy, Inputs to the ventrolateral bed nucleus of the stria terminalis. J Comp Neurol, 2008. 511(5): p. 628-57.

4. Renaud, L.P. and D.A. Hopkins, Amygdala afferents from the mediobasal hypothalamus: an electrophysiological and neuroanatomical study in the rat. Brain Res, 1977. 121(2): p. 201-13.

5. Canteras, N.S., R.B. Simerly, and L.W. Swanson, Connections of the posterior nucleus of the amygdala. J Comp Neurol, 1992. 324(2): p. 143-79.

6. Li, Y.Q., et al., The sites of origin of dopaminergic afferent fibers to the lateral habenular nucleus in the rat. J Comp Neurol, 1993. 333(1): p. 118-33.

7. Sakanaka, M., et al., Topographic organization of the projection from the forebrain subcortical areas to the hippocampal formation of the rat. Neurosci Lett, 1980. 20(3): p. 253-7.

8. Calderazzo, L., et al., Branched connections to the septum and to the entorhinal cortex from the hippocampus, amygdala, and diencephalon in the rat. Brain Res Bull, 1996. 40(4): p. 245-51.

9. Groenewegen, H.J., Organization of the afferent connections of the mediodorsal thalamic nucleus in the rat, related to the mediodorsal-prefrontal topography. Neuroscience, 1988. 24(2): p. 379-431.

10. Otake, K., Cholecystokinin and substance P immunoreactive projections to the paraventricular thalamic nucleus in the rat. Neurosci Res, 2005. 51(4): p. 383-94.

11. Otake, K., D.A. Ruggiero, and Y. Nakamura, Adrenergic innervation of forebrain neurons that project to the paraventricular thalamic nucleus in the rat. Brain Res, 1995. 697(1-2): p. 17-26.

12. Coolen, L.M., et al., Afferent connections of the parvocellular subparafascicular thalamic nucleus in the rat: evidence for functional subdivisions. J Comp Neurol, 2003. 463(2): p. 132-56.

13. Geisler, S. and D.S. Zahm, Afferents of the ventral tegmental area in the rat-anatomical substratum for integrative functions. J Comp Neurol, 2005. 490(3): p. 270-94.

14. Greenwell, T.N., et al., Endomorphin-1 and -2 immunoreactive cells in the hypothalamus are labeled by fluoro-gold injections to the ventral tegmental area. J Comp Neurol, 2002. 454(3): p. 320-8.

15. Peschanski, M. and J.M. Besson, Diencephalic connections of the raphe nuclei of the rat brainstem: an anatomical study with reference to the somatosensory system. J Comp Neurol, 1984. 224(4): p. 509-34.

16. Sim, L.J. and S.A. Joseph, Opiocortin and catecholamine projections to raphe nuclei. Peptides, 1989. 10(5): p. 1019-25.

17. Behzadi, G., et al., Afferents to the median raphe nucleus of the rat: retrograde cholera toxin and wheat germ conjugated horseradish peroxidase tracing, and selective D-[3H]aspartate labelling of possible excitatory amino acid inputs. Neuroscience, 1990. 37(1): p. 77-100.

18. Hermann, D.M., et al., Afferent projections to the rat nuclei raphe magnus, raphe pallidus and reticularis gigantocellularis pars alpha demonstrated by iontophoretic application of choleratoxin (subunit b). J Chem Neuroanat, 1997. 13(1): p. 1-21.

19. Cedarbaum, J.M. and G.K. Aghajanian, Afferent projections to the rat locus coeruleus as determined by a retrograde tracing technique. J Comp Neurol, 1978. 178(1): p. 1-16.

20. Moga, M.M., C.B. Saper, and T.S. Gray, Neuropeptide organization of the hypothalamic projection to the parabrachial nucleus in the rat. J Comp Neurol, 1990. 295(4): p. 662-82.

21. Moga, M.M., et al., Organization of cortical, basal forebrain, and hypothalamic afferents to the parabrachial nucleus in the rat. J Comp Neurol, 1990. 295(4): p. 624-61.

22. Milner, T.A. and V.M. Pickel, Ultrastructural localization and afferent sources of substance P in the rat parabrachial region. Neuroscience, 1986. 17(3): p. 687-707.

23. Thompson, R.H. and L.W. Swanson, Organization of inputs to the dorsomedial nucleus of the hypothalamus: a reexamination with Fluorogold and PHAL in the rat. Brain Res Brain Res Rev, 1998. 27(2): p. 89-118.

24. Hurley, K.M., et al., Efferent projections of the infralimbic cortex of the rat. J Comp Neurol, 1991. 308(2): p. 249-76.

25. Sesack, S.R., et al., Topographical organization of the efferent projections of the medial prefrontal cortex in the rat: an anterograde tract-tracing study with Phaseolus vulgaris leucoagglutinin. J Comp Neurol, 1989. 290(2): p. 213-42.

26. Conrad, L.C. and D.W. Pfaff, Autoradiographic tracing of nucleus accumbens efferents in the rat. Brain Res, 1976. 113(3): p. 589-96.

27. Usuda, I., K. Tanaka, and T. Chiba, Efferent projections of the nucleus accumbens in the rat with special reference to subdivision of the nucleus: biotinylated dextran amine study. Brain Res, 1998. 797(1): p. 73-93.

28. Varoqueaux, F. and P. Poulain, Projections of the mediolateral part of the lateral septum to the hypothalamus, revealed by Fos expression and axonal tracing in rats. Anat Embryol (Berl), 1999. 199(3): p. 249-63.

29. Luiten, P.G. and P. Room, Interrelations between lateral, dorsomedial and ventromedial hypothalamic nuclei in the rat. An HRP study. Brain Res, 1980. 190(2): p. 321-32.

30. Dong, H.W. and L.W. Swanson, Organization of axonal projections from the anterolateral area of the bed nuclei of the stria terminalis. J Comp Neurol, 2004. 468(2): p. 277-98.

31. Dong, H.W. and L.W. Swanson, Projections from bed nuclei of the stria terminalis, anteromedial area: cerebral hemisphere integration of neuroendocrine, autonomic, and behavioral aspects of energy balance. J Comp Neurol, 2006. 494(1): p. 142-78.

32. Dong, H.W. and L.W. Swanson, Projections from bed nuclei of the stria terminalis, dorsomedial nucleus: implications for cerebral hemisphere integration of neuroendocrine, autonomic, and drinking responses. J Comp Neurol, 2006. 494(1): p. 75-107.

33. Dong, H.-W. and L.W. Swanson, Projections from bed nuclei of the stria terminalis, magnocellular nucleus: Implications for cerebral hemisphere regulation of micturition, defecation, and penile erection. The Journal of Comparative Neurology, 2006. 494(1): p. 108-141.

34. Dong, H.W., et al., Basic organization of projections from the oval and fusiform nuclei of the bed nuclei of the stria terminalis in adult rat brain. J Comp Neurol, 2001. 436(4): p. 430-55.

35. Dong, H.W. and L.W. Swanson, Projections from bed nuclei of the stria terminalis, posterior division: implications for cerebral hemisphere regulation of defensive and reproductive behaviors. J Comp Neurol, 2004. 471(4): p. 396-433.

36. Petrovich, G.D., P.Y. Risold, and L.W. Swanson, Organization of projections from the basomedial nucleus of the amygdala: a PHAL study in the rat. J Comp Neurol, 1996. 374(3): p. 387-420.

37. Prewitt, C.M. and J.P. Herman, Anatomical interactions between the central amygdaloid nucleus and the hypothalamic paraventricular nucleus of the rat: a dual tract-tracing analysis. J Chem Neuroanat, 1998. 15(3): p. 173-85.

38. Herkenham, M. and W.J. Nauta, Efferent connections of the habenular nuclei in the rat. J Comp Neurol, 1979. 187(1): p. 19-47.

39. Canteras, N.S. and L.W. Swanson, Projections of the ventral subiculum to the amygdala, septum, and hypothalamus: a PHAL anterograde tract-tracing study in the rat. J Comp Neurol, 1992. 324(2): p. 180-94.

40. Kishi, T., et al., Topographical organization of projections from the subiculum to the hypothalamus in the rat. J Comp Neurol, 2000. 419(2): p. 205-22.

41. Vertes, R.P. and W.B. Hoover, Projections of the paraventricular and paratenial nuclei of the dorsal midline thalamus in the rat. J Comp Neurol, 2008. 508(2): p. 212-37.

42. Moga, M.M., R.P. Weis, and R.Y. Moore, Efferent projections of the paraventricular thalamic nucleus in the rat. J Comp Neurol, 1995. 359(2): p. 221-38.

43. Arluison, M. and P. Derer, Forebrain connections of the rat paraventricular thalamic nucleus as demonstrated using the carbocyanide dye DiI. Neurobiology (Bp), 1993. 1(4): p. 337-50.

44. Ohtake, T. and H. Yamada, Efferent connections of the nucleus reuniens and the rhomboid nucleus in the rat: an anterograde PHA-L tracing study. Neurosci Res, 1989. 6(6): p. 556-68.

45. Azmitia, E.C. and M. Segal, An autoradiographic analysis of the differential ascending projections of the dorsal and median raphe nuclei in the rat. J Comp Neurol, 1978. 179(3): p. 641-67.

46. Bobillier, P., et al., The efferent connections of the nucleus raphe centralis superior in the rat as revealed by radioautography. Brain Res, 1979. 166(1): p. 1-8.

47. Saper, C.B. and A.D. Loewy, Efferent connections of the parabrachial nucleus in the rat. Brain Res, 1980. 197(2): p. 291-317.

48. Krukoff, T.L., K.H. Harris, and J.H. Jhamandas, Efferent projections from the parabrachial nucleus demonstrated with the anterograde tracer Phaseolus vulgaris leucoagglutinin. Brain Res Bull, 1993. 30(1-2): p. 163-72.

49. Bester, H., J.M. Besson, and J.F. Bernard, Organization of efferent projections from the parabrachial area to the hypothalamus: a Phaseolus vulgaris-leucoagglutinin study in the rat. J Comp Neurol, 1997. 383(3): p. 245-81.

50. Ter Horst, G.J., et al., Ascending projections from the solitary tract nucleus to the hypothalamus. A Phaseolus vulgaris lectin tracing study in the rat. Neuroscience, 1989. 31(3): p. 785-97.

Hypothalamus - medial preoptic nucleus (efferents and afferents)

1. Simerly, R.B. and L.W. Swanson, Projections of the medial preoptic nucleus: a Phaseolus vulgaris leucoagglutinin anterograde tract-tracing study in the rat. J Comp Neurol, 1988. 270(2): p. 209-42.

2. Watts, A.G. and L.W. Swanson, Efferent projections of the suprachiasmatic nucleus: II. Studies using retrograde transport of fluorescent dyes and simultaneous peptide immunohistochemistry in the rat. J Comp Neurol, 1987. 258(2): p. 230-52.

3. Coolen, L.M., H.J. Peters, and J.G. Veening, Anatomical interrelationships of the medial preoptic area and other brain regions activated following male sexual behavior: a combined fos and tract-tracing study. J Comp Neurol, 1998. 397(3): p. 421-35.

4. Shin, J.W., J.C. Geerling, and A.D. Loewy, Inputs to the ventrolateral bed nucleus of the stria terminalis. J Comp Neurol, 2008. 511(5): p. 628-57.

5. Ottersen, O.P., Afferent connections to the amygdaloid complex of the rat and cat: II. Afferents from the hypothalamus and the basal telencephalon. J Comp Neurol, 1980. 194(1): p. 267-89.

6. Canteras, N.S., R.B. Simerly, and L.W. Swanson, Connections of the posterior nucleus of the amygdala. J Comp Neurol, 1992. 324(2): p. 143-79.

7. Segal, M., Afferents to the entorhinal cortex of the rat studied by the method of retrograde transport of horseradish peroxidase. Exp Neurol, 1977. 57(3): p. 750-65.

8. Cornwall, J. and O.T. Phillipson, Afferent projections to the dorsal thalamus of the rat as shown by retrograde lectin transport. II. The midline nuclei. Brain Res Bull, 1988. 21(2): p. 147-61.

9. Chen, S. and H.S. Su, Afferent connections of the thalamic paraventricular and parataenial nuclei in the rat--a retrograde tracing study with iontophoretic application of Fluoro-Gold. Brain Res, 1990. 522(1): p. 1-6.

10. Arluison, M. and P. Derer, Forebrain connections of the rat paraventricular thalamic nucleus as demonstrated using the carbocyanide dye DiI. Neurobiology (Bp), 1993. 1(4): p. 337-50.

11. Otake, K., D.A. Ruggiero, and Y. Nakamura, Adrenergic innervation of forebrain neurons that project to the paraventricular thalamic nucleus in the rat. Brain Res, 1995. 697(1-2): p. 17-26.

12. Herkenham, M., The connections of the nucleus reuniens thalami: evidence for a direct thalamo-hippocampal pathway in the rat. J Comp Neurol, 1978. 177(4): p. 589-610.

13. McKenna, J.T. and R.P. Vertes, Afferent projections to nucleus reuniens of the thalamus. J Comp Neurol, 2004. 480(2): p. 115-42.

14. Coolen, L.M., et al., Afferent connections of the parvocellular subparafascicular thalamic nucleus in the rat: evidence for functional subdivisions. J Comp Neurol, 2003. 463(2): p. 132-56.

15. Geisler, S. and D.S. Zahm, Afferents of the ventral tegmental area in the rat-anatomical substratum for integrative functions. J Comp Neurol, 2005. 490(3): p. 270-94.

16. Sim, L.J. and S.A. Joseph, Opiocortin and catecholamine projections to raphe nuclei. Peptides, 1989. 10(5): p. 1019-25.

17. Aghajanian, G.K. and R.Y. Wang, Habenular and other midbrain raphe afferents demonstrated by a modified retrograde tracing technique. Brain Res, 1977. 122(2): p. 229-42.

18. Gervasoni, D., et al., Role and origin of the GABAergic innervation of dorsal raphe serotonergic neurons. J Neurosci, 2000. 20(11): p. 4217-25.

19. Behzadi, G., et al., Afferents to the median raphe nucleus of the rat: retrograde cholera toxin and wheat germ conjugated horseradish peroxidase tracing, and selective D-[3H]aspartate labelling of possible excitatory amino acid inputs. Neuroscience, 1990. 37(1): p. 77-100.

20. Murphy, A.Z., et al., The organization of preoptic-medullary circuits in the male rat: evidence for interconnectivity of neural structures involved in reproductive behavior, antinociception and cardiovascular regulation. Neuroscience, 1999. 91(3): p. 1103-16.

21. Hermann, D.M., et al., Afferent projections to the rat nuclei raphe magnus, raphe pallidus and reticularis gigantocellularis pars alpha demonstrated by iontophoretic application of choleratoxin (subunit b). J Chem Neuroanat, 1997. 13(1): p. 1-21.

22. Rizvi, T.A., et al., Preoptic projections to Barrington's nucleus and the pericoerulear region: architecture and terminal organization. J Comp Neurol, 1994. 347(1): p. 1-24.

23. Luppi, P.H., et al., Afferent projections to the rat locus coeruleus demonstrated by retrograde and anterograde tracing with cholera-toxin B subunit and Phaseolus vulgaris leucoagglutinin. Neuroscience, 1995. 65(1): p. 119-60.

24. Lee, H.S., M.A. Kim, and B.D. Waterhouse, Retrograde double-labeling study of common afferent projections to the dorsal raphe and the nuclear core of the locus coeruleus in the rat. J Comp Neurol, 2005. 481(2): p. 179-93.

25. Cedarbaum, J.M. and G.K. Aghajanian, Afferent projections to the rat locus coeruleus as determined by a retrograde tracing technique. J Comp Neurol, 1978. 178(1): p. 1-16.

26. Moga, M.M., C.B. Saper, and T.S. Gray, Neuropeptide organization of the hypothalamic projection to the parabrachial nucleus in the rat. J Comp Neurol, 1990. 295(4): p. 662-82.

27. Moga, M.M., et al., Organization of cortical, basal forebrain, and hypothalamic afferents to the parabrachial nucleus in the rat. J Comp Neurol, 1990. 295(4): p. 624-61.

28. Swanson, L.W., An autoradiographic study of the efferent connections of the preoptic region in the rat. J Comp Neurol, 1976. 167(2): p. 227-56.

29. Simerly, R.B. and L.W. Swanson, The organization of neural inputs to the medial preoptic nucleus of the rat. J Comp Neurol, 1986. 246(3): p. 312-42.

30. Hurley, K.M., et al., Efferent projections of the infralimbic cortex of the rat. J Comp Neurol, 1991. 308(2): p. 249-76.

31. Swanson, L.W. and W.M. Cowan, The connections of the septal region in the rat. J Comp Neurol, 1979. 186(4): p. 621-55.

32. Varoqueaux, F. and P. Poulain, Projections of the mediolateral part of the lateral septum to the hypothalamus, revealed by Fos expression and axonal tracing in rats. Anat Embryol (Berl), 1999. 199(3): p. 249-63.

33. Dong, H.W. and L.W. Swanson, Organization of axonal projections from the anterolateral area of the bed nuclei of the stria terminalis. J Comp Neurol, 2004. 468(2): p. 277-98.

34. Dong, H.W. and L.W. Swanson, Projections from bed nuclei of the stria terminalis, anteromedial area: cerebral hemisphere integration of neuroendocrine, autonomic, and behavioral aspects of energy balance. J Comp Neurol, 2006. 494(1): p. 142-78.

35. Dong, H.W. and L.W. Swanson, Projections from bed nuclei of the stria terminalis, dorsomedial nucleus: implications for cerebral hemisphere integration of neuroendocrine, autonomic, and drinking responses. J Comp Neurol, 2006. 494(1): p. 75-107.

36. Dong, H.-W. and L.W. Swanson, Projections from bed nuclei of the stria terminalis, magnocellular nucleus: Implications for cerebral hemisphere regulation of micturition, defecation, and penile erection. The Journal of Comparative Neurology, 2006. 494(1): p. 108-141.

37. Dong, H.W. and L.W. Swanson, Projections from bed nuclei of the stria terminalis, posterior division: implications for cerebral hemisphere regulation of defensive and reproductive behaviors. J Comp Neurol, 2004. 471(4): p. 396-433.

38. Gu, G., A. Cornea, and R.B. Simerly, Sexual differentiation of projections from the principal nucleus of the bed nuclei of the stria terminalis. J Comp Neurol, 2003. 460(4): p. 542-62.

39. Petrovich, G.D., P.Y. Risold, and L.W. Swanson, Organization of projections from the basomedial nucleus of the amygdala: a PHAL study in the rat. J Comp Neurol, 1996. 374(3): p. 387-420.

40. McDonald, A.J., Somatostatinergic projections from the amygdala to the bed nucleus of the stria terminalis and medial preoptic-hypothalamic region. Neurosci Lett, 1987. 75(3): p. 271-7.

41. Canteras, N.S., R.B. Simerly, and L.W. Swanson, Organization of projections from the medial nucleus of the amygdala: a PHAL study in the rat. J Comp Neurol, 1995. 360(2): p. 213-45.

42. Kishi, T., et al., Topographical organization of projections from the subiculum to the hypothalamus in the rat. J Comp Neurol, 2000. 419(2): p. 205-22.

43. Luo, A.H. and G. Aston-Jones, Circuit projection from suprachiasmatic nucleus to ventral tegmental area: a novel circadian output pathway. Eur J Neurosci, 2009. 29(4): p. 748-60.

44. Simerly, R.B., L.W. Swanson, and R.A. Gorski, The cells of origin of a sexually dimorphic serotonergic input to the medial preoptic nucleus of the rat. Brain Res, 1984. 324(1): p. 185-9.

45. Vertes, R.P., A PHA-L analysis of ascending projections of the dorsal raphe nucleus in the rat. J Comp Neurol, 1991. 313(4): p. 643-68.

46. Vertes, R.P. and G.F. Martin, Autoradiographic analysis of ascending projections from the pontine and mesencephalic reticular formation and the median raphe nucleus in the rat. J Comp Neurol, 1988. 275(4): p. 511-41.

47. van de Kar, L.D. and S.A. Lorens, Differential serotonergic innervation of individual hypothalamic nuclei and other forebrain regions by the dorsal and median midbrain raphe nuclei. Brain Res, 1979. 162(1): p. 45-54.

48. Saper, C.B. and D. Levisohn, Afferent connections of the median preoptic nucleus in the rat: anatomical evidence for a cardiovascular integrative mechanism in the anteroventral third ventricular (AV3V) region. Brain Res, 1983. 288(1-2): p. 21-31.

49. Bester, H., J.M. Besson, and J.F. Bernard, Organization of efferent projections from the parabrachial area to the hypothalamus: a Phaseolus vulgaris-leucoagglutinin study in the rat. J Comp Neurol, 1997. 383(3): p. 245-81.

50. Krukoff, T.L., K.H. Harris, and J.H. Jhamandas, Efferent projections from the parabrachial nucleus demonstrated with the anterograde tracer Phaseolus vulgaris leucoagglutinin. Brain Res Bull, 1993. 30(1-2): p. 163-72.

51. Ter Horst, G.J., et al., Ascending projections from the solitary tract nucleus to the hypothalamus. A Phaseolus vulgaris lectin tracing study in the rat. Neuroscience, 1989. 31(3): p. 785-97.

Hypothalamus - paraventricular nucleus of hypothalamus (efferents and afferents)

1. Watts, A.G. and L.W. Swanson, Efferent projections of the suprachiasmatic nucleus: II. Studies using retrograde transport of fluorescent dyes and simultaneous peptide immunohistochemistry in the rat. J Comp Neurol, 1987. 258(2): p. 230-52.

2. Sakanaka, M. and S. Magari, Reassessment of enkephalin (ENK)-containing afferents to the rat lateral septum with reference to the fine structures of septal ENK fibers. Brain Res, 1989. 479(2): p. 205-16.

3. Swanson, L.W. and H.G. Kuypers, The paraventricular nucleus of the hypothalamus: cytoarchitectonic subdivisions and organization of projections to the pituitary, dorsal vagal complex, and spinal cord as demonstrated by retrograde fluorescence double-labeling methods. J Comp Neurol, 1980. 194(3): p. 555-70.

4. Ottersen, O.P., Afferent connections to the amygdaloid complex of the rat and cat: II. Afferents from the hypothalamus and the basal telencephalon. J Comp Neurol, 1980. 194(1): p. 267-89.

5. Conrad, L.C. and D.W. Pfaff, Efferents from medial basal forebrain and hypothalamus in the rat. II. An autoradiographic study of the anterior hypothalamus. J Comp Neurol, 1976. 169(2): p. 221-61.

6. Coolen, L.M., et al., Afferent connections of the parvocellular subparafascicular thalamic nucleus in the rat: evidence for functional subdivisions. J Comp Neurol, 2003. 463(2): p. 132-56.

7. Geisler, S. and D.S. Zahm, Afferents of the ventral tegmental area in the rat-anatomical substratum for integrative functions. J Comp Neurol, 2005. 490(3): p. 270-94.

8. Rodaros, D., et al., Corticotropin-releasing factor projections from limbic forebrain and paraventricular nucleus of the hypothalamus to the region of the ventral tegmental area. Neuroscience, 2007. 150(1): p. 8-13.

9. Gervasoni, D., et al., Role and origin of the GABAergic innervation of dorsal raphe serotonergic neurons. J Neurosci, 2000. 20(11): p. 4217-25.

10. Sim, L.J. and S.A. Joseph, Opiocortin and catecholamine projections to raphe nuclei. Peptides, 1989. 10(5): p. 1019-25.

11. Peschanski, M. and J.M. Besson, Diencephalic connections of the raphe nuclei of the rat brainstem: an anatomical study with reference to the somatosensory system. J Comp Neurol, 1984. 224(4): p. 509-34.

12. Hermann, D.M., et al., Afferent projections to the rat nuclei raphe magnus, raphe pallidus and reticularis gigantocellularis pars alpha demonstrated by iontophoretic application of choleratoxin (subunit b). J Chem Neuroanat, 1997. 13(1): p. 1-21.

13. Luppi, P.H., et al., Afferent projections to the rat locus coeruleus demonstrated by retrograde and anterograde tracing with cholera-toxin B subunit and Phaseolus vulgaris leucoagglutinin. Neuroscience, 1995. 65(1): p. 119-60.

14. Cedarbaum, J.M. and G.K. Aghajanian, Afferent projections to the rat locus coeruleus as determined by a retrograde tracing technique. J Comp Neurol, 1978. 178(1): p. 1-16.

15. Lechner, S.M. and R.J. Valentino, Glucocorticoid receptor-immunoreactivity in corticotrophin-releasing factor afferents to the locus coeruleus. Brain Res, 1999. 816(1): p. 17-28.

16. Valentino, R.J., et al., Corticotropin-releasing factor innervation of the locus coeruleus region: distribution of fibers and sources of input. Neuroscience, 1992. 48(3): p. 689-705.

17. Reyes, B.A., et al., Hypothalamic projections to locus coeruleus neurons in rat brain. Eur J Neurosci, 2005. 22(1): p. 93-106.

18. Moga, M.M., C.B. Saper, and T.S. Gray, Neuropeptide organization of the hypothalamic projection to the parabrachial nucleus in the rat. J Comp Neurol, 1990. 295(4): p. 662-82.

19. Moga, M.M., et al., Organization of cortical, basal forebrain, and hypothalamic afferents to the parabrachial nucleus in the rat. J Comp Neurol, 1990. 295(4): p. 624-61.

20. Milner, T.A. and V.M. Pickel, Ultrastructural localization and afferent sources of substance P in the rat parabrachial region. Neuroscience, 1986. 17(3): p. 687-707.

21. Milner, T.A., T.H. Joh, and V.M. Pickel, Tyrosine hydroxylase in the rat parabrachial region: ultrastructural localization and extrinsic sources of immunoreactivity. J Neurosci, 1986. 6(9): p. 2585-603.

22. Saper, C.B., et al., Direct hypothalamo-autonomic connections. Brain Res, 1976. 117(2): p. 305-12.

23. Guevara-Aguilar, R., et al., Olfactory and visceral projections to the paraventricular nucleus. Brain Res Bull, 1988. 20(6): p. 799-801.

24. Takagishi, M. and T. Chiba, Efferent projections of the infralimbic (area 25) region of the medial prefrontal cortex in the rat: an anterograde tracer PHA-L study. Brain Res, 1991. 566(1-2): p. 26-39.

25. Beaulieu, J., D. Champagne, and G. Drolet, Enkephalin innervation of the paraventricular nucleus of the hypothalamus: distribution of fibers and origins of input. J Chem Neuroanat, 1996. 10(2): p. 79-92.

26. Csaki, A., et al., Localization of glutamatergic/aspartatergic neurons projecting to the hypothalamic paraventricular nucleus studied by retrograde transport of [3H]D-aspartate autoradiography. Neuroscience, 2000. 101(3): p. 637-55.

27. Rutherford, J.G., An investigation of a possible direct projection from the medial nucleus of the cerebellum to the paraventricular nucleus of the hypothalamus in the rat: a study using retrograde WGA-HRP and Fluoro-Gold tracing techniques. Anat Embryol (Berl), 1995. 192(3): p. 229-38.

28. Staiger, J.F. and F.G. Wouterlood, Efferent projections from the lateral septal nucleus to the anterior hypothalamus in the rat: a study combining Phaseolus vulgaris-leucoagglutinin tracing with vasopressin immunocytochemistry. Cell Tissue Res, 1990. 261(1): p. 17-23.

29. Sawchenko, P.E. and L.W. Swanson, The organization of forebrain afferents to the paraventricular and supraoptic nuclei of the rat. J Comp Neurol, 1983. 218(2): p. 121-44.

30. Champagne, D., J. Beaulieu, and G. Drolet, CRFergic innervation of the paraventricular nucleus of the rat hypothalamus: a tract-tracing study. J Neuroendocrinol, 1998. 10(2): p. 119-31.

31. Dong, H.W. and L.W. Swanson, Organization of axonal projections from the anterolateral area of the bed nuclei of the stria terminalis. J Comp Neurol, 2004. 468(2): p. 277-98.

32. Dong, H.W. and L.W. Swanson, Projections from bed nuclei of the stria terminalis, anteromedial area: cerebral hemisphere integration of neuroendocrine, autonomic, and behavioral aspects of energy balance. J Comp Neurol, 2006. 494(1): p. 142-78.

33. Dong, H.W. and L.W. Swanson, Projections from bed nuclei of the stria terminalis, dorsomedial nucleus: implications for cerebral hemisphere integration of neuroendocrine, autonomic, and drinking responses. J Comp Neurol, 2006. 494(1): p. 75-107.

34. Dong, H.-W. and L.W. Swanson, Projections from bed nuclei of the stria terminalis, magnocellular nucleus: Implications for cerebral hemisphere regulation of micturition, defecation, and penile erection. The Journal of Comparative Neurology, 2006. 494(1): p. 108-141.

35. Dong, H.W., et al., Basic organization of projections from the oval and fusiform nuclei of the bed nuclei of the stria terminalis in adult rat brain. J Comp Neurol, 2001. 436(4): p. 430-55.

36. Dong, H.W. and L.W. Swanson, Projections from bed nuclei of the stria terminalis, posterior division: implications for cerebral hemisphere regulation of defensive and reproductive behaviors. J Comp Neurol, 2004. 471(4): p. 396-433.

37. Gu, G., A. Cornea, and R.B. Simerly, Sexual differentiation of projections from the principal nucleus of the bed nuclei of the stria terminalis. J Comp Neurol, 2003. 460(4): p. 542-62.

38. Dong, H.W. and L.W. Swanson, Projections from the rhomboid nucleus of the bed nuclei of the stria terminalis: implications for cerebral hemisphere regulation of ingestive behaviors. J Comp Neurol, 2003. 463(4): p. 434-72.

39. Petrovich, G.D., P.Y. Risold, and L.W. Swanson, Organization of projections from the basomedial nucleus of the amygdala: a PHAL study in the rat. J Comp Neurol, 1996. 374(3): p. 387-420.

40. Prewitt, C.M. and J.P. Herman, Anatomical interactions between the central amygdaloid nucleus and the hypothalamic paraventricular nucleus of the rat: a dual tract-tracing analysis. J Chem Neuroanat, 1998. 15(3): p. 173-85.

41. Zahm, D.S., et al., Direct comparison of projections from the central amygdaloid region and nucleus accumbens shell. Eur J Neurosci, 1999. 11(4): p. 1119-26.

42. Canteras, N.S., R.B. Simerly, and L.W. Swanson, Organization of projections from the medial nucleus of the amygdala: a PHAL study in the rat. J Comp Neurol, 1995. 360(2): p. 213-45.

43. Canteras, N.S. and L.W. Swanson, Projections of the ventral subiculum to the amygdala, septum, and hypothalamus: a PHAL anterograde tract-tracing study in the rat. J Comp Neurol, 1992. 324(2): p. 180-94.

44. Kishi, T., et al., Topographical organization of projections from the subiculum to the hypothalamus in the rat. J Comp Neurol, 2000. 419(2): p. 205-22.

45. Ohtake, T. and H. Yamada, Efferent connections of the nucleus reuniens and the rhomboid nucleus in the rat: an anterograde PHA-L tracing study. Neurosci Res, 1989. 6(6): p. 556-68.

46. Wouterlood, F.G., E. Saldana, and M.P. Witter, Projection from the nucleus reuniens thalami to the hippocampal region: light and electron microscopic tracing study in the rat with the anterograde tracer Phaseolus vulgaris-leucoagglutinin. J Comp Neurol, 1990. 296(2): p. 179-203.

47. Petrov, T., T.L. Krukoff, and J.H. Jhamandas, The hypothalamic paraventricular and lateral parabrachial nuclei receive collaterals from raphe nucleus neurons: a combined double retrograde and immunocytochemical study. J Comp Neurol, 1992. 318(1): p. 18-26.

48. Vertes, R.P. and G.F. Martin, Autoradiographic analysis of ascending projections from the pontine and mesencephalic reticular formation and the median raphe nucleus in the rat. J Comp Neurol, 1988. 275(4): p. 511-41.

49. Azmitia, E.C. and M. Segal, An autoradiographic analysis of the differential ascending projections of the dorsal and median raphe nuclei in the rat. J Comp Neurol, 1978. 179(3): p. 641-67.

50. Cunningham, E.T., Jr. and P.E. Sawchenko, Anatomical specificity of noradrenergic inputs to the paraventricular and supraoptic nuclei of the rat hypothalamus. J Comp Neurol, 1988. 274(1): p. 60-76.

51. Bester, H., J.M. Besson, and J.F. Bernard, Organization of efferent projections from the parabrachial area to the hypothalamus: a Phaseolus vulgaris-leucoagglutinin study in the rat. J Comp Neurol, 1997. 383(3): p. 245-81.

52. Krukoff, T.L., K.H. Harris, and J.H. Jhamandas, Efferent projections from the parabrachial nucleus demonstrated with the anterograde tracer Phaseolus vulgaris leucoagglutinin. Brain Res Bull, 1993. 30(1-2): p. 163-72.

53. Saper, C.B. and A.D. Loewy, Efferent connections of the parabrachial nucleus in the rat. Brain Res, 1980. 197(2): p. 291-317.

54. Cunningham, E.T., Jr., M.C. Bohn, and P.E. Sawchenko, Organization of adrenergic inputs to the paraventricular and supraoptic nuclei of the hypothalamus in the rat. J Comp Neurol, 1990. 292(4): p. 651-67.

55. Ter Horst, G.J., et al., Ascending projections from the solitary tract nucleus to the hypothalamus. A Phaseolus vulgaris lectin tracing study in the rat. Neuroscience, 1989. 31(3): p. 785-97.

Hypothalamus - supramammillary nucleus (efferents and afferents)

1. Saper, C.B., Convergence of autonomic and limbic connections in the insular cortex of the rat. J Comp Neurol, 1982. 210(2): p. 163-73.

2. Vertes, R.P., PHA-L analysis of projections from the supramammillary nucleus in the rat. J Comp Neurol, 1992. 326(4): p. 595-622.

3. Borhegyi, Z., et al., The supramammillary nucleus innervates cholinergic and GABAergic neurons in the medial septum-diagonal band of Broca complex. Neuroscience, 1998. 82(4): p. 1053-65.

4. Kiss, J., et al., The supramammillo-hippocampal and supramammillo-septal glutamatergic/aspartatergic projections in the rat: a combined [3H]D-aspartate autoradiographic and immunohistochemical study. Neuroscience, 2000. 97(4): p. 657-69.

5. Gartner, U., et al., Immunofluorescence and immunoelectron microscopic evidence for differences in myelination of GABAergic and cholinergic septohippocampal fibres. Int J Dev Neurosci, 2001. 19(3): p. 347-52.

6. Ottersen, O.P., Afferent connections to the amygdaloid complex of the rat and cat: II. Afferents from the hypothalamus and the basal telencephalon. J Comp Neurol, 1980. 194(1): p. 267-89.

7. Haglund, L., L.W. Swanson, and C. Kohler, The projection of the supramammillary nucleus to the hippocampal formation: an immunohistochemical and anterograde transport study with the lectin PHA-L in the rat. J Comp Neurol, 1984. 229(2): p. 171-85.

8. Magloczky, Z., L. Acsady, and T.F. Freund, Principal cells are the postsynaptic targets of supramammillary afferents in the hippocampus of the rat. Hippocampus, 1994. 4(3): p. 322-34.

9. Segal, M. and S. Landis, Afferents to the hippocampus of the rat studied with the method of retrograde transport of horseradish peroxidase. Brain Res, 1974. 78(1): p. 1-15.

10. Sakanaka, M., et al., Topographic organization of the projection from the forebrain subcortical areas to the hippocampal formation of the rat. Neurosci Lett, 1980. 20(3): p. 253-7.

11. Pasquier, D.A. and F. Reinoso-Suarez, The topographic organization of hypothalamic and brain stem projections to the hippocampus. Brain Res Bull, 1978. 3(4): p. 373-89.

12. Wyss, J.M., L.W. Swanson, and W.M. Cowan, Evidence for an input to the molecular layer and the stratum granulosum of the dentate gyrus from the supramammillary region of the hypothalamus. Anat Embryol (Berl), 1979. 156(2): p. 165-76.

13. Calderazzo, L., et al., Branched connections to the septum and to the entorhinal cortex from the hippocampus, amygdala, and diencephalon in the rat. Brain Res Bull, 1996. 40(4): p. 245-51.

14. Deacon, T.W., et al., Afferent connections of the perirhinal cortex in the rat. J Comp Neurol, 1983. 220(2): p. 168-90.

15. van Groen, T. and J.M. Wyss, The postsubicular cortex in the rat: characterization of the fourth region of the subicular cortex and its connections. Brain Res, 1990. 529(1-2): p. 165-77.

16. Cornwall, J. and O.T. Phillipson, Afferent projections to the dorsal thalamus of the rat as shown by retrograde lectin transport. II. The midline nuclei. Brain Res Bull, 1988. 21(2): p. 147-61.

17. Groenewegen, H.J., Organization of the afferent connections of the mediodorsal thalamic nucleus in the rat, related to the mediodorsal-prefrontal topography. Neuroscience, 1988. 24(2): p. 379-431.

18. Ray, J.P., et al., Sources of presumptive glutamatergic/aspartatergic afferents to the mediodorsal nucleus of the thalamus in the rat. J Comp Neurol, 1992. 320(4): p. 435-56.

19. McKenna, J.T. and R.P. Vertes, Afferent projections to nucleus reuniens of the thalamus. J Comp Neurol, 2004. 480(2): p. 115-42.

20. Herkenham, M., The connections of the nucleus reuniens thalami: evidence for a direct thalamo-hippocampal pathway in the rat. J Comp Neurol, 1978. 177(4): p. 589-610.

21. Geisler, S. and D.S. Zahm, Afferents of the ventral tegmental area in the rat-anatomical substratum for integrative functions. J Comp Neurol, 2005. 490(3): p. 270-94.

22. Peschanski, M. and J.M. Besson, Diencephalic connections of the raphe nuclei of the rat brainstem: an anatomical study with reference to the somatosensory system. J Comp Neurol, 1984. 224(4): p. 509-34.

23. Hayakawa, T., H. Ito, and K. Zyo, Neuroanatomical study of afferent projections to the supramammillary nucleus of the rat. Anat Embryol (Berl), 1993. 188(2): p. 139-48.

24. Hurley, K.M., et al., Efferent projections of the infralimbic cortex of the rat. J Comp Neurol, 1991. 308(2): p. 249-76.

25. Takagishi, M. and T. Chiba, Efferent projections of the infralimbic (area 25) region of the medial prefrontal cortex in the rat: an anterograde tracer PHA-L study. Brain Res, 1991. 566(1-2): p. 26-39.

26. Conrad, L.C. and D.W. Pfaff, Autoradiographic tracing of nucleus accumbens efferents in the rat. Brain Res, 1976. 113(3): p. 589-96.

27. Kiss, J., et al., Possible glutamatergic/aspartatergic projections to the supramammillary nucleus and their origins in the rat studied by selective [(3)H]D-aspartate labelling and immunocytochemistry. Neuroscience, 2002. 111(3): p. 671-91.

28. Borhegyi, Z. and T.F. Freund, Dual projection from the medial septum to the supramammillary nucleus in the rat. Brain Res Bull, 1998. 46(5): p. 453-9.

29. Dong, H.W. and L.W. Swanson, Organization of axonal projections from the anterolateral area of the bed nuclei of the stria terminalis. J Comp Neurol, 2004. 468(2): p. 277-98.

30. Dong, H.W. and L.W. Swanson, Projections from bed nuclei of the stria terminalis, anteromedial area: cerebral hemisphere integration of neuroendocrine, autonomic, and behavioral aspects of energy balance. J Comp Neurol, 2006. 494(1): p. 142-78.

31. Dong, H.W. and L.W. Swanson, Projections from bed nuclei of the stria terminalis, dorsomedial nucleus: implications for cerebral hemisphere integration of neuroendocrine, autonomic, and drinking responses. J Comp Neurol, 2006. 494(1): p. 75-107.

32. Dong, H.-W. and L.W. Swanson, Projections from bed nuclei of the stria terminalis, magnocellular nucleus: Implications for cerebral hemisphere regulation of micturition, defecation, and penile erection. The Journal of Comparative Neurology, 2006. 494(1): p. 108-141.

33. Dong, H.W., et al., Basic organization of projections from the oval and fusiform nuclei of the bed nuclei of the stria terminalis in adult rat brain. J Comp Neurol, 2001. 436(4): p. 430-55.

34. Gu, G., A. Cornea, and R.B. Simerly, Sexual differentiation of projections from the principal nucleus of the bed nuclei of the stria terminalis. J Comp Neurol, 2003. 460(4): p. 542-62.

35. Dong, H.W. and L.W. Swanson, Projections from bed nuclei of the stria terminalis, posterior division: implications for cerebral hemisphere regulation of defensive and reproductive behaviors. J Comp Neurol, 2004. 471(4): p. 396-433.

36. Petrovich, G.D., P.Y. Risold, and L.W. Swanson, Organization of projections from the basomedial nucleus of the amygdala: a PHAL study in the rat. J Comp Neurol, 1996. 374(3): p. 387-420.

37. Canteras, N.S. and L.W. Swanson, Projections of the ventral subiculum to the amygdala, septum, and hypothalamus: a PHAL anterograde tract-tracing study in the rat. J Comp Neurol, 1992. 324(2): p. 180-94.

38. Kishi, T., et al., Topographical organization of projections from the subiculum to the hypothalamus in the rat. J Comp Neurol, 2000. 419(2): p. 205-22.

39. Donovan, M.K. and J.M. Wyss, Evidence for some collateralization between cortical and diencephalic efferent axons of the rat subicular cortex. Brain Res, 1983. 259(2): p. 181-92.

40. Vertes, R.P., A PHA-L analysis of ascending projections of the dorsal raphe nucleus in the rat. J Comp Neurol, 1991. 313(4): p. 643-68.

41. Bobillier, P., et al., The efferent connections of the nucleus raphe centralis superior in the rat as revealed by radioautography. Brain Res, 1979. 166(1): p. 1-8.

42. Vertes, R.P. and G.F. Martin, Autoradiographic analysis of ascending projections from the pontine and mesencephalic reticular formation and the median raphe nucleus in the rat. J Comp Neurol, 1988. 275(4): p. 511-41.

43. Vertes, R.P., W.J. Fortin, and A.M. Crane, Projections of the median raphe nucleus in the rat. J Comp Neurol, 1999. 407(4): p. 555-82.

Hypothalamus - tuberomammillary nucleus (efferents and afferents)

1. Kohler, C., et al., The cytoarchitecture, histochemistry and projections of the tuberomammillary nucleus in the rat. Neuroscience, 1985. 16(1): p. 85-110.

2. Zaborszky, L., et al., Cholinergic and GABAergic afferents to the olfactory bulb in the rat with special emphasis on the projection neurons in the nucleus of the horizontal limb of the diagonal band. J Comp Neurol, 1986. 243(4): p. 488-509.

3. Staines, W.A., et al., The hypothalamus receives major projections from the tuberomammillary nucleus in rat. Neurosci Lett, 1987. 76(3): p. 257-62.

4. Saper, C.B., Convergence of autonomic and limbic connections in the insular cortex of the rat. J Comp Neurol, 1982. 210(2): p. 163-73.

5. Lee, S.B., B.J. Chang, and H.S. Lee, Organization of histamine-immunoreactive, tuberomammillary neurons projecting to the dorsal tier of the substantia nigra compacta in the rat. Brain Res, 2008. 1203: p. 79-88.

6. Geisler, S. and D.S. Zahm, Afferents of the ventral tegmental area in the rat-anatomical substratum for integrative functions. J Comp Neurol, 2005. 490(3): p. 270-94.

7. Greenwell, T.N., et al., Endomorphin-1 and -2 immunoreactive cells in the hypothalamus are labeled by fluoro-gold injections to the ventral tegmental area. J Comp Neurol, 2002. 454(3): p. 320-8.

8. Lee, H.S., B.Y. Lee, and B.D. Waterhouse, Retrograde study of projections from the tuberomammillary nucleus to the dorsal raphe and the locus coeruleus in the rat. Brain Res, 2005. 1043(1-2): p. 65-75.

9. Gervasoni, D., et al., Role and origin of the GABAergic innervation of dorsal raphe serotonergic neurons. J Neurosci, 2000. 20(11): p. 4217-25.

10. Luppi, P.H., et al., Afferent projections to the rat locus coeruleus demonstrated by retrograde and anterograde tracing with cholera-toxin B subunit and Phaseolus vulgaris leucoagglutinin. Neuroscience, 1995. 65(1): p. 119-60.

11. Lee, H.S., M.A. Kim, and B.D. Waterhouse, Retrograde double-labeling study of common afferent projections to the dorsal raphe and the nuclear core of the locus coeruleus in the rat. J Comp Neurol, 2005. 481(2): p. 179-93.

12. Dietrichs, E., L. Wiklund, and D.E. Haines, The hypothalamo-cerebellar projection in the rat: origin and transmitter. Arch Ital Biol, 1992. 130(3): p. 203-11.

13. Hong, E.Y. and H.S. Lee, Retrograde study of projections from the tuberomammillary nucleus to the mesopontine cholinergic complex in the rat. Brain Res, 2011. 1383: p. 169-78.

14. Takada, M., Z.K. Li, and T. Hattori, A direct projection from the tuberomammillary nucleus to the spinal cord in the rat. Neurosci Lett, 1987. 79(3): p. 257-62.

15. Gaykema, R.P., et al., Prefrontal cortical projections to the cholinergic neurons in the basal forebrain. J Comp Neurol, 1991. 303(4): p. 563-83.

16. Dong, H.W. and L.W. Swanson, Projections from bed nuclei of the stria terminalis, anteromedial area: cerebral hemisphere integration of neuroendocrine, autonomic, and behavioral aspects of energy balance. J Comp Neurol, 2006. 494(1): p. 142-78.

17. Dong, H.W. and L.W. Swanson, Projections from bed nuclei of the stria terminalis, dorsomedial nucleus: implications for cerebral hemisphere integration of neuroendocrine, autonomic, and drinking responses. J Comp Neurol, 2006. 494(1): p. 75-107.

18. Dong, H.-W. and L.W. Swanson, Projections from bed nuclei of the stria terminalis, magnocellular nucleus: Implications for cerebral hemisphere regulation of micturition, defecation, and penile erection. The Journal of Comparative Neurology, 2006. 494(1): p. 108-141.

19. Canteras, N.S. and L.W. Swanson, Projections of the ventral subiculum to the amygdala, septum, and hypothalamus: a PHAL anterograde tract-tracing study in the rat. J Comp Neurol, 1992. 324(2): p. 180-94.

20. Kishi, T., et al., Topographical organization of projections from the subiculum to the hypothalamus in the rat. J Comp Neurol, 2000. 419(2): p. 205-22.

Hypothalamus – ventromedial nucleus of hypothalamus (efferents and afferents)

1. Aarnisalo, A.A. and P. Panula, Neuropeptide FF-containing efferent projections from the medial hypothalamus of rat: a Phaseolus vulgaris leucoagglutinin study. Neuroscience, 1995. 65(1): p. 175-92.

2. Conrad, L.C. and D.W. Pfaff, Efferents from medial basal forebrain and hypothalamus in the rat. II. An autoradiographic study of the anterior hypothalamus. J Comp Neurol, 1976. 169(2): p. 221-61.

3. Saper, C.B., L.W. Swanson, and W.M. Cowan, The efferent connections of the ventromedial nucleus of the hypothalamus of the rat. J Comp Neurol, 1976. 169(4): p. 409-42.

4. Hoffman, G.E., et al., The hypothalamic ventromedial nucleus sends a met-enkephalin projection to the preoptic area's periventricular zone in the female rat. Brain Res Mol Brain Res, 1996. 36(2): p. 201-10.

5. Ottersen, O.P., Afferent connections to the amygdaloid complex of the rat and cat: II. Afferents from the hypothalamus and the basal telencephalon. J Comp Neurol, 1980. 194(1): p. 267-89.

6. Renaud, L.P. and D.A. Hopkins, Amygdala afferents from the mediobasal hypothalamus: an electrophysiological and neuroanatomical study in the rat. Brain Res, 1977. 121(2): p. 201-13.

7. Sakanaka, M., T. Shibasaki, and K. Lederis, Distribution and efferent projections of corticotropin-releasing factor-like immunoreactivity in the rat amygdaloid complex. Brain Res, 1986. 382(2): p. 213-38.

8. Calderazzo, L., et al., Branched connections to the septum and to the entorhinal cortex from the hippocampus, amygdala, and diencephalon in the rat. Brain Res Bull, 1996. 40(4): p. 245-51.

9. Cornwall, J. and O.T. Phillipson, Afferent projections to the dorsal thalamus of the rat as shown by retrograde lectin transport. II. The midline nuclei. Brain Res Bull, 1988. 21(2): p. 147-61.

10. Herkenham, M., The connections of the nucleus reuniens thalami: evidence for a direct thalamo-hippocampal pathway in the rat. J Comp Neurol, 1978. 177(4): p. 589-610.

11. McKenna, J.T. and R.P. Vertes, Afferent projections to nucleus reuniens of the thalamus. J Comp Neurol, 2004. 480(2): p. 115-42.

12. Coolen, L.M., et al., Afferent connections of the parvocellular subparafascicular thalamic nucleus in the rat: evidence for functional subdivisions. J Comp Neurol, 2003. 463(2): p. 132-56.

13. Geisler, S. and D.S. Zahm, Afferents of the ventral tegmental area in the rat-anatomical substratum for integrative functions. J Comp Neurol, 2005. 490(3): p. 270-94.

14. Greenwell, T.N., et al., Endomorphin-1 and -2 immunoreactive cells in the hypothalamus are labeled by fluoro-gold injections to the ventral tegmental area. J Comp Neurol, 2002. 454(3): p. 320-8.

15. Luppi, P.H., et al., Afferent projections to the rat locus coeruleus demonstrated by retrograde and anterograde tracing with cholera-toxin B subunit and Phaseolus vulgaris leucoagglutinin. Neuroscience, 1995. 65(1): p. 119-60.

16. Milner, T.A. and V.M. Pickel, Ultrastructural localization and afferent sources of substance P in the rat parabrachial region. Neuroscience, 1986. 17(3): p. 687-707.

17. Moga, M.M., C.B. Saper, and T.S. Gray, Neuropeptide organization of the hypothalamic projection to the parabrachial nucleus in the rat. J Comp Neurol, 1990. 295(4): p. 662-82.

18. Zaborszky, L. and G.B. Makara, Intrahypothalamic connections: an electron microscopic study in the rat. Exp Brain Res, 1979. 34(2): p. 201-15.

19. Swanson, L.W. and H.G. Kuypers, A direct projection from the ventromedial nucleus and retrochiasmatic area of the hypothalamus to the medulla and spinal cord of the rat. Neurosci Lett, 1980. 17(3): p. 307-12.

20. Millhouse, O.E., The organization of the ventromedial hypothalamic nucleus. Brain Res, 1973. 55(1): p. 71-87.

21. Fahrbach, S.E., J.I. Morrell, and D.W. Pfaff, Studies of ventromedial hypothalamic afferents in the rat using three methods of HRP application. Exp Brain Res, 1989. 77(2): p. 221-33.

22. Luiten, P.G. and P. Room, Interrelations between lateral, dorsomedial and ventromedial hypothalamic nuclei in the rat. An HRP study. Brain Res, 1980. 190(2): p. 321-32.

23. Dong, H.W. and L.W. Swanson, Projections from bed nuclei of the stria terminalis, posterior division: implications for cerebral hemisphere regulation of defensive and reproductive behaviors. J Comp Neurol, 2004. 471(4): p. 396-433.

24. Shammah-Lagnado, S.J., et al., Supracapsular bed nucleus of the stria terminalis contains central and medial extended amygdala elements: evidence from anterograde and retrograde tracing experiments in the rat. J Comp Neurol, 2000. 422(4): p. 533-55.

25. Petrovich, G.D., P.Y. Risold, and L.W. Swanson, Organization of projections from the basomedial nucleus of the amygdala: a PHAL study in the rat. J Comp Neurol, 1996. 374(3): p. 387-420.

26. Krettek, J.E. and J.L. Price, Amygdaloid projections to subcortical structures within the basal forebrain and brainstem in the rat and cat. J Comp Neurol, 1978. 178(2): p. 225-54.

27. Canteras, N.S., R.B. Simerly, and L.W. Swanson, Organization of projections from the medial nucleus of the amygdala: a PHAL study in the rat. J Comp Neurol, 1995. 360(2): p. 213-45.

28. Canteras, N.S., R.B. Simerly, and L.W. Swanson, Connections of the posterior nucleus of the amygdala. J Comp Neurol, 1992. 324(2): p. 143-79.

29. Chiba, T., Collateral projection from the amygdalo--hippocampal transition area and CA1 to the hypothalamus and medial prefrontal cortex in the rat. Neurosci Res, 2000. 38(4): p. 373-83.

30. Donovan, M.K. and J.M. Wyss, Evidence for some collateralization between cortical and diencephalic efferent axons of the rat subicular cortex. Brain Res, 1983. 259(2): p. 181-92.

31. Canteras, N.S. and L.W. Swanson, Projections of the ventral subiculum to the amygdala, septum, and hypothalamus: a PHAL anterograde tract-tracing study in the rat. J Comp Neurol, 1992. 324(2): p. 180-94.

32. Kishi, T., et al., Topographical organization of projections from the subiculum to the hypothalamus in the rat. J Comp Neurol, 2000. 419(2): p. 205-22.

33. Handelmann, G.E., et al., Extra-hippocampal projections of CCK neurons of the hippocampus and subiculum. Peptides, 1983. 4(3): p. 331-4.

34. LeDoux, J.E., D.A. Ruggiero, and D.J. Reis, Projections to the subcortical forebrain from anatomically defined regions of the medial geniculate body in the rat. J Comp Neurol, 1985. 242(2): p. 182-213.

35. Moga, M.M., R.P. Weis, and R.Y. Moore, Efferent projections of the paraventricular thalamic nucleus in the rat. J Comp Neurol, 1995. 359(2): p. 221-38.

36. Ohtake, T. and H. Yamada, Efferent connections of the nucleus reuniens and the rhomboid nucleus in the rat: an anterograde PHA-L tracing study. Neurosci Res, 1989. 6(6): p. 556-68.

37. Peschanski, M. and J.M. Besson, Diencephalic connections of the raphe nuclei of the rat brainstem: an anatomical study with reference to the somatosensory system. J Comp Neurol, 1984. 224(4): p. 509-34.

38. Bester, H., J.M. Besson, and J.F. Bernard, Organization of efferent projections from the parabrachial area to the hypothalamus: a Phaseolus vulgaris-leucoagglutinin study in the rat. J Comp Neurol, 1997. 383(3): p. 245-81.

39. Fulwiler, C.E. and C.B. Saper, Cholecystokinin-immunoreactive innervation of the ventromedial hypothalamus in the rat: possible substrate for autonomic regulation of feeding. Neurosci Lett, 1985. 53(3): p. 289-96.

40. Krukoff, T.L., K.H. Harris, and J.H. Jhamandas, Efferent projections from the parabrachial nucleus demonstrated with the anterograde tracer Phaseolus vulgaris leucoagglutinin. Brain Res Bull, 1993. 30(1-2): p. 163-72.

41. Saper, C.B. and A.D. Loewy, Efferent connections of the parabrachial nucleus in the rat. Brain Res, 1980. 197(2): p. 291-317.

42. Ter Horst, G.J., et al., Ascending projections from the solitary tract nucleus to the hypothalamus. A Phaseolus vulgaris lectin tracing study in the rat. Neuroscience, 1989. 31(3): p. 785-97.

**Amygdala**

Intraregional connections

1. Jolkkonen, E. and A. Pitkanen, Intrinsic connections of the rat amygdaloid complex: projections originating in the central nucleus. J Comp Neurol, 1998. 395(1): p. 53-72.

2. Canteras, N.S., R.B. Simerly, and L.W. Swanson, Organization of projections from the medial nucleus of the amygdala: a PHAL study in the rat. J Comp Neurol, 1995. 360(2): p. 213-45.

3. Pitkanen, A., et al., Intrinsic connections of the rat amygdaloid complex: projections originating in the lateral nucleus. J Comp Neurol, 1995. 356(2): p. 288-310.

4. Ottersen, O.P., Connections of the amygdala of the rat. IV: Corticoamygdaloid and intraamygdaloid connections as studied with axonal transport of horseradish peroxidase. J Comp Neurol, 1982. 205(1): p. 30-48.

5. Krettek, J.E. and J.L. Price, A description of the amygdaloid complex in the rat and cat with observations on intra-amygdaloid axonal connections. J Comp Neurol, 1978. 178(2): p. 255-80.

6. Petrovich, G.D., P.Y. Risold, and L.W. Swanson, Organization of projections from the basomedial nucleus of the amygdala: a PHAL study in the rat. J Comp Neurol, 1996. 374(3): p. 387-420.

7. Savander, V., et al., Intrinsic connections of the rat amygdaloid complex: projections originating in the basal nucleus. J Comp Neurol, 1995. 361(2): p. 345-68.

8. Kemppainen, S., E. Jolkkonen, and A. Pitkanen, Projections from the posterior cortical nucleus of the amygdala to the hippocampal formation and parahippocampal region in rat. Hippocampus, 2002. 12(6): p. 735-55.

9. Ottersen, O.P., Afferent connections to the amygdaloid complex of the rat and cat: II. Afferents from the hypothalamus and the basal telencephalon. J Comp Neurol, 1980. 194(1): p. 267-89.

10. Canteras, N.S., R.B. Simerly, and L.W. Swanson, Connections of the posterior nucleus of the amygdala. J Comp Neurol, 1992. 324(2): p. 143-79.

11. Savander, V., et al., Lateral nucleus of the rat amygdala is reciprocally connected with basal and accessory basal nuclei: a light and electron microscopic study. Neuroscience, 1997. 77(3): p. 767-81.

12. Stefanacci, L., et al., Projections from the lateral nucleus to the basal nucleus of the amygdala: a light and electron microscopic PHA-L study in the rat. J Comp Neurol, 1992. 323(4): p. 586-601.

13. Bourgeais, L., C. Gauriau, and J.F. Bernard, Projections from the nociceptive area of the central nucleus of the amygdala to the forebrain: a PHA-L study in the rat. Eur J Neurosci, 2001. 14(2): p. 229-55.

Amygdala - basal nucleus of the accessory olfactory tract (efferents and afferents)

1. Takagishi, M. and T. Chiba, Efferent projections of the infralimbic (area 25) region of the medial prefrontal cortex in the rat: an anterograde tracer PHA-L study. Brain Res, 1991. 566(1-2): p. 26-39.

2. Ottersen, O.P., Connections of the amygdala of the rat. IV: Corticoamygdaloid and intraamygdaloid connections as studied with axonal transport of horseradish peroxidase. J Comp Neurol, 1982. 205(1): p. 30-48.

3. Shi, C.J. and M.D. Cassell, Cortical, thalamic, and amygdaloid connections of the anterior and posterior insular cortices. J Comp Neurol, 1998. 399(4): p. 440-68.

4. Turner, B.H. and M. Herkenham, Thalamoamygdaloid projections in the rat: a test of the amygdala's role in sensory processing. J Comp Neurol, 1991. 313(2): p. 295-325.

Amygdala - medial nucleus of amygdala (efferents and afferents)

1. Canteras, N.S., R.B. Simerly, and L.W. Swanson, Organization of projections from the medial nucleus of the amygdala: a PHAL study in the rat. J Comp Neurol, 1995. 360(2): p. 213-45.

2. Raisman, G., An experimental study of the projection of the amygdala to the accessory olfactory bulb and its relationship to the concept of a dual olfactory system. Exp Brain Res, 1972. 14(4): p. 395-408.

3. de Olmos, J., H. Hardy, and L. Heimer, The afferent connections of the main and the accessory olfactory bulb formations in the rat: an experimental HRP-study. J Comp Neurol, 1978. 181(2): p. 213-44.

4. Jasmin, L., et al., Rostral agranular insular cortex and pain areas of the central nervous system: a tract-tracing study in the rat. J Comp Neurol, 2004. 468(3): p. 425-40.

5. Shi, C.J. and M.D. Cassell, Cortical, thalamic, and amygdaloid connections of the anterior and posterior insular cortices. J Comp Neurol, 1998. 399(4): p. 440-68.

6. Christie, M.J., et al., Excitatory amino acid projections to the nucleus accumbens septi in the rat: a retrograde transport study utilizing D[3H]aspartate and [3H]GABA. Neuroscience, 1987. 22(2): p. 425-39.

7. Caffe, A.R., F.W. van Leeuwen, and P.G. Luiten, Vasopressin cells in the medial amygdala of the rat project to the lateral septum and ventral hippocampus. J Comp Neurol, 1987. 261(2): p. 237-52.

8. Prewitt, C.M. and J.P. Herman, Anatomical interactions between the central amygdaloid nucleus and the hypothalamic paraventricular nucleus of the rat: a dual tract-tracing analysis. J Chem Neuroanat, 1998. 15(3): p. 173-85.

9. Beltramino, C.A., et al., Amygdaloid input to transiently tyrosine hydroxylase immunoreactive neurons in the bed nucleus of the stria terminalis of the rat. Brain Res, 1996. 706(1): p. 37-46.

10. Krettek, J.E. and J.L. Price, Amygdaloid projections to subcortical structures within the basal forebrain and brainstem in the rat and cat. J Comp Neurol, 1978. 178(2): p. 225-54.

11. McDonald, A.J., Somatostatinergic projections from the amygdala to the bed nucleus of the stria terminalis and medial preoptic-hypothalamic region. Neurosci Lett, 1987. 75(3): p. 271-7.

12. Shammah-Lagnado, S.J., et al., Supracapsular bed nucleus of the stria terminalis contains central and medial extended amygdala elements: evidence from anterograde and retrograde tracing experiments in the rat. J Comp Neurol, 2000. 422(4): p. 533-55.

13. Shin, J.W., J.C. Geerling, and A.D. Loewy, Inputs to the ventrolateral bed nucleus of the stria terminalis. J Comp Neurol, 2008. 511(5): p. 628-57.

14. Magoul, R., et al., Direct and indirect enkephalinergic synaptic inputs to the rat arcuate nucleus studied by combination of retrograde tracing and immunocytochemistry. Neuroscience, 1993. 55(4): p. 1055-66.

15. Luiten, P.G. and P. Room, Interrelations between lateral, dorsomedial and ventromedial hypothalamic nuclei in the rat. An HRP study. Brain Res, 1980. 190(2): p. 321-32.

16. Coolen, L.M., H.J. Peters, and J.G. Veening, Anatomical interrelationships of the medial preoptic area and other brain regions activated following male sexual behavior: a combined fos and tract-tracing study. J Comp Neurol, 1998. 397(3): p. 421-35.

17. Simerly, R.B. and L.W. Swanson, The organization of neural inputs to the medial preoptic nucleus of the rat. J Comp Neurol, 1986. 246(3): p. 312-42.

18. Abrahamson, E.E. and R.Y. Moore, The posterior hypothalamic area: chemoarchitecture and afferent connections. Brain Res, 2001. 889(1-2): p. 1-22.

19. Comoli, E., E.R. Ribeiro-Barbosa, and N.S. Canteras, Afferent connections of the dorsal premammillary nucleus. J Comp Neurol, 2000. 423(1): p. 83-98.

20. Csaki, A., et al., Localization of glutamatergic/aspartatergic neurons projecting to the hypothalamic paraventricular nucleus studied by retrograde transport of [3H]D-aspartate autoradiography. Neuroscience, 2000. 101(3): p. 637-55.

21. Rutherford, J.G., An investigation of a possible direct projection from the medial nucleus of the cerebellum to the paraventricular nucleus of the hypothalamus in the rat: a study using retrograde WGA-HRP and Fluoro-Gold tracing techniques. Anat Embryol (Berl), 1995. 192(3): p. 229-38.

22. Fahrbach, S.E., J.I. Morrell, and D.W. Pfaff, Studies of ventromedial hypothalamic afferents in the rat using three methods of HRP application. Exp Brain Res, 1989. 77(2): p. 221-33.

23. Calderazzo, L., et al., Branched connections to the septum and to the entorhinal cortex from the hippocampus, amygdala, and diencephalon in the rat. Brain Res Bull, 1996. 40(4): p. 245-51.

24. Meibach, R.C. and A. Siegel, Thalamic projections of the hippocampal formation: evidence for an alternate pathway involving the internal capsule. Brain Res, 1977. 134(1): p. 1-12.

25. Ray, J.P., et al., Sources of presumptive glutamatergic/aspartatergic afferents to the mediodorsal nucleus of the thalamus in the rat. J Comp Neurol, 1992. 320(4): p. 435-56.

26. Chen, S. and H.S. Su, Afferent connections of the thalamic paraventricular and parataenial nuclei in the rat--a retrograde tracing study with iontophoretic application of Fluoro-Gold. Brain Res, 1990. 522(1): p. 1-6.

27. Herkenham, M., The connections of the nucleus reuniens thalami: evidence for a direct thalamo-hippocampal pathway in the rat. J Comp Neurol, 1978. 177(4): p. 589-610.

28. McKenna, J.T. and R.P. Vertes, Afferent projections to nucleus reuniens of the thalamus. J Comp Neurol, 2004. 480(2): p. 115-42.

29. Geisler, S. and D.S. Zahm, Afferents of the ventral tegmental area in the rat-anatomical substratum for integrative functions. J Comp Neurol, 2005. 490(3): p. 270-94.

30. Peyron, C., et al., Forebrain afferents to the rat dorsal raphe nucleus demonstrated by retrograde and anterograde tracing methods. Neuroscience, 1998. 82(2): p. 443-68.

31. Sim, L.J. and S.A. Joseph, Opiocortin and catecholamine projections to raphe nuclei. Peptides, 1989. 10(5): p. 1019-25.

32. Hermann, D.M., et al., Afferent projections to the rat nuclei raphe magnus, raphe pallidus and reticularis gigantocellularis pars alpha demonstrated by iontophoretic application of choleratoxin (subunit b). J Chem Neuroanat, 1997. 13(1): p. 1-21.

33. Milner, T.A. and V.M. Pickel, Ultrastructural localization and afferent sources of substance P in the rat parabrachial region. Neuroscience, 1986. 17(3): p. 687-707.

34. Hurley, K.M., et al., Efferent projections of the infralimbic cortex of the rat. J Comp Neurol, 1991. 308(2): p. 249-76.

35. Takagishi, M. and T. Chiba, Efferent projections of the infralimbic (area 25) region of the medial prefrontal cortex in the rat: an anterograde tracer PHA-L study. Brain Res, 1991. 566(1-2): p. 26-39.

36. McDonald, A.J., F. Mascagni, and L. Guo, Projections of the medial and lateral prefrontal cortices to the amygdala: a Phaseolus vulgaris leucoagglutinin study in the rat. Neuroscience, 1996. 71(1): p. 55-75.

37. Cassell, M.D. and D.J. Wright, Topography of projections from the medial prefrontal cortex to the amygdala in the rat. Brain Res Bull, 1986. 17(3): p. 321-33.

38. Ottersen, O.P., Connections of the amygdala of the rat. IV: Corticoamygdaloid and intraamygdaloid connections as studied with axonal transport of horseradish peroxidase. J Comp Neurol, 1982. 205(1): p. 30-48.

39. McDonald, A.J., et al., Cortical afferents to the extended amygdala. Ann N Y Acad Sci, 1999. 877: p. 309-38.

40. Ottersen, O.P., Afferent connections to the amygdaloid complex of the rat and cat: II. Afferents from the hypothalamus and the basal telencephalon. J Comp Neurol, 1980. 194(1): p. 267-89.

41. Dong, H.W. and L.W. Swanson, Projections from bed nuclei of the stria terminalis, anteromedial area: cerebral hemisphere integration of neuroendocrine, autonomic, and behavioral aspects of energy balance. J Comp Neurol, 2006. 494(1): p. 142-78.

42. Dong, H.W. and L.W. Swanson, Projections from bed nuclei of the stria terminalis, dorsomedial nucleus: implications for cerebral hemisphere integration of neuroendocrine, autonomic, and drinking responses. J Comp Neurol, 2006. 494(1): p. 75-107.

43. Poulin, J.F., et al., Enkephalinergic afferents of the centromedial amygdala in the rat. J Comp Neurol, 2006. 496(6): p. 859-76.

44. Dong, H.W. and L.W. Swanson, Projections from bed nuclei of the stria terminalis, posterior division: implications for cerebral hemisphere regulation of defensive and reproductive behaviors. J Comp Neurol, 2004. 471(4): p. 396-433.

45. Gu, G., A. Cornea, and R.B. Simerly, Sexual differentiation of projections from the principal nucleus of the bed nuclei of the stria terminalis. J Comp Neurol, 2003. 460(4): p. 542-62.

46. Risold, P.Y., N.S. Canteras, and L.W. Swanson, Organization of projections from the anterior hypothalamic nucleus: a Phaseolus vulgaris-leucoagglutinin study in the rat. J Comp Neurol, 1994. 348(1): p. 1-40.

47. Conrad, L.C. and D.W. Pfaff, Efferents from medial basal forebrain and hypothalamus in the rat. I. An autoradiographic study of the medial preoptic area. J Comp Neurol, 1976. 169(2): p. 185-219.

48. Sim, L.J. and S.A. Joseph, Arcuate nucleus projections to brainstem regions which modulate nociception. J Chem Neuroanat, 1991. 4(2): p. 97-109.

49. Aarnisalo, A.A. and P. Panula, Neuropeptide FF-containing efferent projections from the medial hypothalamus of rat: a Phaseolus vulgaris leucoagglutinin study. Neuroscience, 1995. 65(1): p. 175-92.

50. Goto, M., et al., Projections from the subfornical region of the lateral hypothalamic area. J Comp Neurol, 2005. 493(3): p. 412-38.

51. Swanson, L.W., An autoradiographic study of the efferent connections of the preoptic region in the rat. J Comp Neurol, 1976. 167(2): p. 227-56.

52. Vertes, R.P., et al., Ascending projections of the posterior nucleus of the hypothalamus: PHA-L analysis in the rat. J Comp Neurol, 1995. 359(1): p. 90-116.

53. Veening, J.G., Subcortical afferents of the amygdaloid complex in the rat: an HRP study. Neurosci Lett, 1978. 8(3): p. 197-202.

54. Canteras, N.S., R.B. Simerly, and L.W. Swanson, Projections of the ventral premammillary nucleus. J Comp Neurol, 1992. 324(2): p. 195-212.

55. Conrad, L.C. and D.W. Pfaff, Efferents from medial basal forebrain and hypothalamus in the rat. II. An autoradiographic study of the anterior hypothalamus. J Comp Neurol, 1976. 169(2): p. 221-61.

56. Vertes, R.P., PHA-L analysis of projections from the supramammillary nucleus in the rat. J Comp Neurol, 1992. 326(4): p. 595-622.

57. Saper, C.B., L.W. Swanson, and W.M. Cowan, The efferent connections of the ventromedial nucleus of the hypothalamus of the rat. J Comp Neurol, 1976. 169(4): p. 409-42.

58. McDonald, A.J. and F. Mascagni, Projections of the lateral entorhinal cortex to the amygdala: a Phaseolus vulgaris leucoagglutinin study in the rat. Neuroscience, 1997. 77(2): p. 445-59.

59. McIntyre, D.C., M.E. Kelly, and W.A. Staines, Efferent projections of the anterior perirhinal cortex in the rat. J Comp Neurol, 1996. 369(2): p. 302-18.

60. LeDoux, J.E., C. Farb, and D.A. Ruggiero, Topographic organization of neurons in the acoustic thalamus that project to the amygdala. J Neurosci, 1990. 10(4): p. 1043-54.

61. Yasui, Y., C.B. Saper, and D.F. Cechetto, Calcitonin gene-related peptide (CGRP) immunoreactive projections from the thalamus to the striatum and amygdala in the rat. J Comp Neurol, 1991. 308(2): p. 293-310.

62. Vertes, R.P., W.B. Hoover, and J.J. Rodriguez, Projections of the central medial nucleus of the thalamus in the rat: node in cortical, striatal and limbic forebrain circuitry. Neuroscience, 2012. 219: p. 120-36.

63. Tsumori, T., et al., Synaptic organization of GABAergic projections from the substantia nigra pars reticulata and the reticular thalamic nucleus to the parafascicular thalamic nucleus in the rat. Brain Res, 2002. 957(2): p. 231-41.

64. Vertes, R.P. and W.B. Hoover, Projections of the paraventricular and paratenial nuclei of the dorsal midline thalamus in the rat. J Comp Neurol, 2008. 508(2): p. 212-37.

65. Turner, B.H. and M. Herkenham, Thalamoamygdaloid projections in the rat: a test of the amygdala's role in sensory processing. J Comp Neurol, 1991. 313(2): p. 295-325.

66. Moga, M.M., R.P. Weis, and R.Y. Moore, Efferent projections of the paraventricular thalamic nucleus in the rat. J Comp Neurol, 1995. 359(2): p. 221-38.

67. Ohtake, T. and H. Yamada, Efferent connections of the nucleus reuniens and the rhomboid nucleus in the rat: an anterograde PHA-L tracing study. Neurosci Res, 1989. 6(6): p. 556-68.

68. Loughlin, S.E. and J.H. Fallon, Dopaminergic and non-dopaminergic projections to amygdala from substantia nigra and ventral tegmental area. Brain Res, 1983. 262(2): p. 334-8.

69. Haglund, L., et al., Forebrain projections of the ventral tegmentum as studied by axonal transport of [3H]dopamine in the rat. Neurosci Lett, 1979. 12(2-3): p. 301-6.

70. Swanson, L.W., The projections of the ventral tegmental area and adjacent regions: a combined fluorescent retrograde tracer and immunofluorescence study in the rat. Brain Res Bull, 1982. 9(1-6): p. 321-53.

71. Beckstead, R.M., V.B. Domesick, and W.J. Nauta, Efferent connections of the substantia nigra and ventral tegmental area in the rat. Brain Res, 1979. 175(2): p. 191-217.

72. Ma, Q.P., et al., Serotonergic projections from the nucleus raphe dorsalis to the amygdala in the rat. Neurosci Lett, 1991. 134(1): p. 21-4.

73. Vertes, R.P., A PHA-L analysis of ascending projections of the dorsal raphe nucleus in the rat. J Comp Neurol, 1991. 313(4): p. 643-68.

74. Azmitia, E.C. and M. Segal, An autoradiographic analysis of the differential ascending projections of the dorsal and median raphe nuclei in the rat. J Comp Neurol, 1978. 179(3): p. 641-67.

75. Vertes, R.P. and G.F. Martin, Autoradiographic analysis of ascending projections from the pontine and mesencephalic reticular formation and the median raphe nucleus in the rat. J Comp Neurol, 1988. 275(4): p. 511-41.

76. Krukoff, T.L., K.H. Harris, and J.H. Jhamandas, Efferent projections from the parabrachial nucleus demonstrated with the anterograde tracer Phaseolus vulgaris leucoagglutinin. Brain Res Bull, 1993. 30(1-2): p. 163-72.

77. Voshart, K. and D. van der Kooy, The organization of the efferent projections of the parabrachial nucleus of the forebrain in the rat: a retrograde fluorescent double-labeling study. Brain Res, 1981. 212(2): p. 271-86.

78. Krettek, J.E. and J.L. Price, A description of the amygdaloid complex in the rat and cat with observations on intra-amygdaloid axonal connections. J Comp Neurol, 1978. 178(2): p. 255-80.

79. Roder, S. and J. Ciriello, Innervation of the amygdaloid complex by catecholaminergic cell groups of the ventrolateral medulla. J Comp Neurol, 1993. 332(1): p. 105-22.

Amygdala - accessory basal nucleus (efferents and afferents)

1. Brog, J.S., et al., The patterns of afferent innervation of the core and shell in the "accumbens" part of the rat ventral striatum: immunohistochemical detection of retrogradely transported fluoro-gold. J Comp Neurol, 1993. 338(2): p. 255-78.

2. Ray, J.P., et al., Sources of presumptive glutamatergic/aspartatergic afferents to the mediodorsal nucleus of the thalamus in the rat. J Comp Neurol, 1992. 320(4): p. 435-56.

3. Ray, J.P. and J.L. Price, The organization of the thalamocortical connections of the mediodorsal thalamic nucleus in the rat, related to the ventral forebrain-prefrontal cortex topography. J Comp Neurol, 1992. 323(2): p. 167-97.

4. Majak, K., et al., Projections from the amygdaloid complex to the claustrum and the endopiriform nucleus: a Phaseolus vulgaris leucoagglutinin study in the rat. J Comp Neurol, 2002. 451(3): p. 236-49.

5. McDonald, A.J., F. Mascagni, and L. Guo, Projections of the medial and lateral prefrontal cortices to the amygdala: a Phaseolus vulgaris leucoagglutinin study in the rat. Neuroscience, 1996. 71(1): p. 55-75.

6. Takagishi, M. and T. Chiba, Efferent projections of the infralimbic (area 25) region of the medial prefrontal cortex in the rat: an anterograde tracer PHA-L study. Brain Res, 1991. 566(1-2): p. 26-39.

7. Ottersen, O.P., Connections of the amygdala of the rat. IV: Corticoamygdaloid and intraamygdaloid connections as studied with axonal transport of horseradish peroxidase. J Comp Neurol, 1982. 205(1): p. 30-48.

8. Shi, C.J. and M.D. Cassell, Cortical, thalamic, and amygdaloid connections of the anterior and posterior insular cortices. J Comp Neurol, 1998. 399(4): p. 440-68.

9. McDonald, A.J. and F. Mascagni, Projections of the lateral entorhinal cortex to the amygdala: a Phaseolus vulgaris leucoagglutinin study in the rat. Neuroscience, 1997. 77(2): p. 445-59.

10. Turner, B.H. and M. Herkenham, Thalamoamygdaloid projections in the rat: a test of the amygdala's role in sensory processing. J Comp Neurol, 1991. 313(2): p. 295-325.

11. Vertes, R.P. and W.B. Hoover, Projections of the paraventricular and paratenial nuclei of the dorsal midline thalamus in the rat. J Comp Neurol, 2008. 508(2): p. 212-37.

12. Ottersen, O.P., Afferent connections to the amygdaloid complex of the rat and cat: II. Afferents from the hypothalamus and the basal telencephalon. J Comp Neurol, 1980. 194(1): p. 267-89.

Amygdala - basal nucleus (efferents and afferents)

1. Brog, J.S., et al., The patterns of afferent innervation of the core and shell in the "accumbens" part of the rat ventral striatum: immunohistochemical detection of retrogradely transported fluoro-gold. J Comp Neurol, 1993. 338(2): p. 255-78.

2. Beckstead, R.M., Afferent connections of the entorhinal area in the rat as demonstrated by retrograde cell-labeling with horseradish peroxidase. Brain Res, 1978. 152(2): p. 249-64.

3. Krettek, J.E. and J.L. Price, Projections from the amygdala to the perirhinal and entorhinal cortices and the subiculum. Brain Res, 1974. 71(1): p. 150-4.

4. Ray, J.P., et al., Sources of presumptive glutamatergic/aspartatergic afferents to the mediodorsal nucleus of the thalamus in the rat. J Comp Neurol, 1992. 320(4): p. 435-56.

5. Kuroda, M. and J.L. Price, Synaptic organization of projections from basal forebrain structures to the mediodorsal thalamic nucleus of the rat. J Comp Neurol, 1991. 303(4): p. 513-33.

6. Oda, S., Ultrastructure and distribution of corticothalamic fiber terminals from the posterior cingulate cortex and the presubiculum to the anteroventral thalamic nucleus of the rat. Brain Res Bull, 1997. 42(6): p. 485-91.

7. Herkenham, M., The afferent and efferent connections of the ventromedial thalamic nucleus in the rat. J Comp Neurol, 1979. 183(3): p. 487-517.

8. Savander, V., et al., Intrinsic connections of the rat amygdaloid complex: projections originating in the basal nucleus. J Comp Neurol, 1995. 361(2): p. 345-68.

9. Majak, K., et al., Projections from the amygdaloid complex to the claustrum and the endopiriform nucleus: a Phaseolus vulgaris leucoagglutinin study in the rat. J Comp Neurol, 2002. 451(3): p. 236-49.
[truncated: 528,020 more chars]
