# Supplementary material for: A multiscale cerebral neurochemical connectome of the rat brain
Source: PLoS Biol. 2017 Jul 3;15(7):e2002612. doi: 10.1371/journal.pbio.2002612 (PMC5507471; doi:10.1371/journal.pbio.2002612)
Supplement: S2 Text — (DOCX) [file pbio.2002612.s029.docx]

**Supplementary Information**

**A Multi-Scale Cerebral Neurochemical Connectome of the Rat Brain**

Hamid R. Noori^1,2,3,*^, Judith Schöttler^1^, Maria Ercsey-Ravasz^4,5^, Alejandro Cosa-Linan^1^, Melinda Varga^6^, Zoltan Toroczkai^6^, Rainer Spanagel^1^

^1^ Institute of Psychopharmacology, Central Institute of Mental Health, Medical Faculty Mannheim, University of Heidelberg, Mannheim, Germany

^2^ Max Planck Institute for Biological Cybernetics, Tübingen, Germany

^3^ Institut des Hautes Etudes Scientifiques, Bures-sur-Yvette, France

^4^ Faculty of Physics, Babes-Bolyai University, Cluj-Napoca, Romania

^5^ Romanian Institute of Science and Technology, Cluj-Napoca, Romania

^6^ Physics Department and the Interdisciplinary Center for Network Science and Applications, University of Notre Dame, Notre Dame, IN 46556, USA

1. Keywords

2. Generation of receptor density distribution maps

3. ChemNetDB – Neurochemical connectivity matrices

4. List of abbreviations (chemicals)

5. List of abbreviations (brain regions)

1. **Keywords**

The online portal of the National Library of Medicine (<http://www.ncbi.nlm.nih.gov/pubmed/>) including PubMed, PubMed Central and MEDLINE was used as the platform for literature research. A systematic screening of the original research articles published until February 2015 was performed based on the 1,750 keyword combinations:

*morphology (AND) brain region/subregion (AND) rat; cytoarchitecture (AND) brain region/subregion (AND) rat; projection neurons (AND) brain region/subregion (AND) rat; projection (AND) brain region/subregion (AND) rat; interneurons (AND) brain region/subregion (AND) rat; glutamate receptors (AND) brain region/subregion (AND) rat; GABA receptors (AND) brain region/subregion (AND) rat; dopamine receptors (AND) brain region/subregion (AND) rat; 5-HT receptors (AND) brain region/subregion (AND) rat; cholinergic receptors (AND) brain region/subregion (AND) rat; opioid receptors (AND) brain region/subregion (AND) rat; cannabinoid receptors (AND) brain region/subregion (AND) rat; enkephalin receptors (AND) brain region/subregion (AND) rat; β-endorphin receptors (AND) brain region/subregion (AND) rat.*

Thereby, the brain regions/subregions were defined as: *Main olfactory bulb; Accessory olfactory bulb; Infralimbic cortex; Prelimbic cortex; Anterior cingulate cortex; Lateral prefrontal cortex; Insular cortex, pars anterior; Insular cortex, pars posterior; Nucleus accumbens, shell; Nucleus accubens, core; Caudate Putamen; Lateral septal nucleus; Medial septal nucleus; Nucleus of the diagonal band (horizontal and vertical limb); Septofimbrial nucleus; Triangular septal nucleus; Bed nucleus of the stria terminalis, transverse nucleus; Bed nucleus of the stria terminalis, dorsomedial nucleus; Bed nucleus of the stria terminalis, dorsolateral nucleus; Bed nucleus of the stria terminalis, juxtacapsular nucleus; Bed nucleus of the stria terminalis, supracapsular nucleus; Bed nucleus of the stria terminalis, magnocellular nucleus; Bed nucleus of the stria terminalis, principal nucleus; Bed nucleus of the stria terminalis, ventrolateral nucleus; Bed nucleus of the stria terminalis, ventromedial nucleus; Bed nucleus of the stria terminalis, anterolateral nucleus; Bed nucleus of the stria terminalis, fusiform nucleus; Bed nucleus of the stria terminalis, interfascicular nucleus; Bed nucleus of the stria terminalis, rhomboid nucleus; Bed nucleus of the stria terminalis, anteromedial nucleus; Bed nucleus of the stria terminalis, anterodorsal area; Bed nucleus of the stria terminalis, anteroventral area; Bed nucleus of the stria terminalis, ventral nucleus; Bed nucleus of the stria terminalis, oval nucleus; Bed nucleus of the stria terminalis, posteromedial nucleus; Bed nucleus of the stria terminalus, posterolateral nucleus; Bed nucleus of the stria terminalis, preoptic nucleus; Globus pallidus; Ventral pallidum; Entopeduncular nucleus; Anterior hypothalamic nucleus; Lateral preoptic nucleus; Medial preoptic nucleus; Paraventricular hypothalamic nucleus; Posterior hypothalamic nucleus; Premammillary nucleus (ventral and dorsal); Suprachiasmatic nucleus; Tuberomammillary nucleus; Arcuate nucleus; Dorsomedial hypothalamic nucleus; Lateral hypothalamus; Mammillary nucleus (medial and lateral); Periventricular hypothalamic nucleus; Supramammillary nucleus; Supraoptic nucleus; Ventromedial hypothalamic nucleus; Central nucleus of amygdala; Medial nucleus of amygdala; Lateral nucleus of amygdala; Basolateral nucleus of amygdala; Basomedial nucleus of amygdala; Basal nucleus of amygdala; Accessory basal nucleus of amygdala; Cortical nuclei of amygdala (anterior and posterior); Posterior nucleus of amygdala; Nucleus of the lateral olfactory tract; Basal nucleus of the accessory olfactory tract; Medial Habenula; Lateral Habenula; Presubiculum; Postsubiculum; Parasubiculum; Subiculum; Entorhinal cortex; Dentate Gyrus; CA1; CA2; CA3; Perirhinal cortex; Postrhinal cortex; Anteromedial nucleus of thalamus; Anteroventral nucleus of thalamus; Central lateral nucleus of thalamus; Central medial nucleus of thalamus; Intermediodorsal nucleus of thalamus; Lateral geniculate nucleus of thalamus; Medial geniculate nucleus of thalamus; Nucleus reuniens; Paracentral nucleus of thalamus; Parafascicular nucleus of thalamus; Parataenial nucleus of thalamus; Paraventricular nucleus of thalamus; Reticular nucleus of thalamus; Ventral lateral nucleus of thalamus; Ventral medial nucleus of thalamus; Anterodorsal nucleus of thalamus; Interanteromedial nucleus of thalamus; Laterodorsal nucleus of thalamus; Lateroposterior nucleus of thalamus; Mediodorsal nucleus of thalamus; Rhomboid nucleus of thalamus; Subparafascicular nucleus of thalamus; Posterior thalamus; Nucleus submedius; Anterolateral Nucleus Of The Thalamus; Subthalamic Nucleus; Substantia nigra, pars reticulate; Substantia nigra, pars compacta; Substantia nigra, pars lateralis; Rostral linear nucleus; Caudal linear nucleus; Paranigral nucleus; Parabrachial nucleus pigmentosus; Interfascicular nucleus; Dorsal raphe nucleus; Medial raphe nucleus; Nucleus raphe magnus; Nucleus raphe pontis; Nucleus raphe pallidus; Nucleus raphe obscurus; locus coeruleus; Lateral parabarchial nucleus; Medial parabrachial nucleus; Kölliker-Fuse nucleus; Pontine nuclei.* Furthermore, the keywords were varied by the anatomical positioning terms and their combinations (e.g. dorsal, lateral and dorsolateral) in order to increase the robustness of the data mining step with respect to anatomical nomenclature.

In addition, the reference sections of identified papers as well as review and meta-analysis articles were then screened for further relevant citations. Reviewers, in pairs, independently screened titles and abstracts of articles and reviewed the full text of any title or abstract deemed potentially eligible by either reviewer. Reviewers resolved disagreements by discussion. Among these studies, only peer-reviewed original research articles in English language were chosen for data mining.

1. **Generation of receptor density distribution maps**

In addition to the confounds of the data mining procedure for ChemNetDB (supplementary data A) which utilized in-situ hybridization on the rat brain, Pubmed was screened with respect to the following keywords: (*mGluR1-mGluR7, Kainate, AMPA, NMDA) (AND/OR) mRNA (AND) distribution (AND) rat; (D1-D5) (AND/OR) mRNA (AND) distribution (AND) rat; CB (AND/OR) mRNA (AND) distribution (AND) rat; Opioid (AND/OR) mRNA (AND) distribution (AND) rat; (GABA A-B) (AND/OR) mRNA (AND) distribution (AND) rat; (5-HT1-7) (AND/OR) mRNA (AND) distribution (AND) rat; beta adrenergic mRNA (AND) distribution (AND) rat; alpha adrenergic mRNA (AND) distribution (AND) rat; muscarinic acetylcholine receptor mRNA (AND) distribution (AND) rat; nicotinic acetylcholine receptor mRNA (AND) distribution (AND) rat.*

Out of 1,843 mRNA expression level and receptor binding studies, data were extracted from 103 original research articles that fulfilled the selection criteria (see Methods section). mRNA molecules transfer DNA-information to ribosomes, where it translates into a chain of amino acids/peptides. As every receptor consists of these components, the mRNA distribution provide a measures for the localization of receptors and receptors subtypes in the brain. The presence of corresponding mRNA transcript is an indicator for the presence of particular receptors, although the exact correlation between mRNA and protein depends on the biological samples under consideration (Maier et al., 2009^[[1]](#footnote-1)^). Therefore, the mRNA distribution results are only used as binary support of receptor binding studies. Nonetheless, the extracted data was often qualitative and for each brain area was classified in the following form:

| Sign | Denotation |
| --- | --- |
| No sign | No information |
| X | present |
| - | Not detected |
| + | very low/slight/scarce/scattered |
| ++ | low/weak |
| +++ | moderat;e |
| ++++ | dense/high/intense/strong |
| +++++ | very dense/very intense/most/very strong |

The outcome of the data mining process is presented in tables SDB.1-4 of supplementary data B. Similar to a weighted meta-analysis, the qualitiative reports for each brain region were averaged. Hereby, the number of animals used in an experiment was considered as the weight of the respective experiment. This procedure translates the qualitative observations into normalized values for receptor distribution densities within the neurochemical connectome. The conjunction of these local, network node-specific normalized values leads to brain-wide distribution maps for gluatamtergic (mGluR1-7, Kainate1-2, AMPA, NMDAR1-R2(A-D)), monoaminergic (D1-D5, 5-HT1-7(A-C), β1-2, α1-2(A-D)), cholinergic (M1-M5, nAChRα(2-6)β(2-3)), GABAergic (GABAAα(1-6)β(2-3)γ(1-3)δ, GABAB1(a,b,p), GABAB2), opioid (μ, δ, κ, ORL1) and cannabinoid receptors. To illustrate these receptor distribution maps, the stereotaxic atlas of rat brain (Paxinos and Watson, 2013^[[2]](#footnote-2)^) was projected on sagittal magnetic resonance imaging templates of the rat brain encompassing the 125 brain areas (sagittal planes at 0.4, 1.4, 2.4 and 3.9 mm) and the normalized values were assigned to the respective areas on the atlas (S2-12 Fig) which were identified within the database. The higher the normalized values, the higher is the color density in the relevant brain region.

1. **ChemNetDB - Neurochemical connectivity matrices**

The connectivity matrices are presented from rostral to caudal. The coronal brain slides associated with specific Bregma levels were used to estimate Euclidian distances between different brain regions (figure SD1).


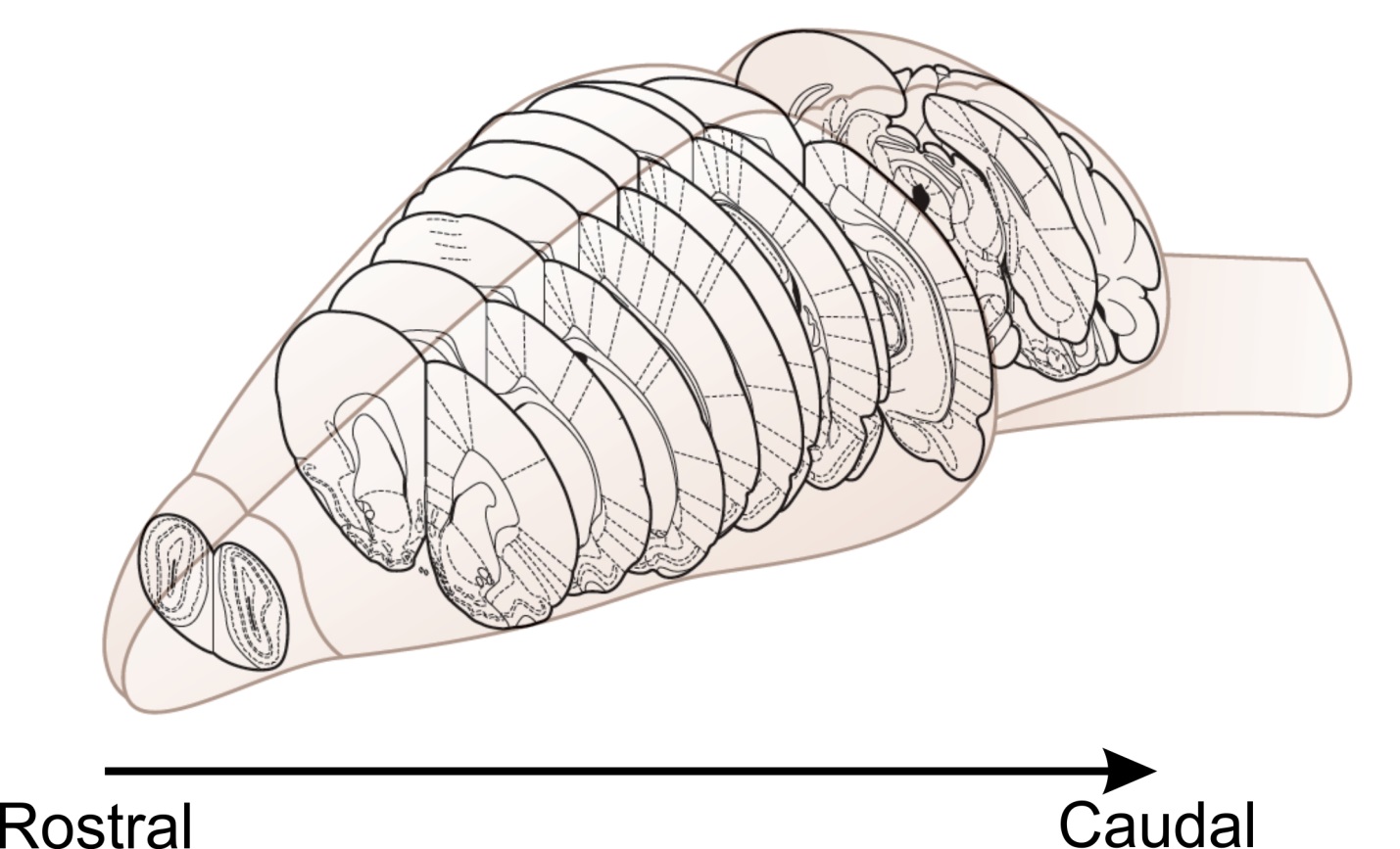


**S2 Figure 1.** The connectivity matrices are presented from rostral to caudal and the illustrated Bregma levels were used to obtain the distances between different brain regions.

For each brain region, four types of tables are presented. The blue tables provide the meta-data for each large-scale brain area, including its subregions and their abbreviations. Violet tables present the intraregional connectivity of the brain area. Green and orange tables present the efferent and afferent interregional connectivity matrices, respectively. Each table includes a hierarchical (based on network coarsening results) listing of the involved brain regions, abbreviations, transmitters and respective references. For completeness, the database also includes chemical information even if it was provided in form of a negation e.g. in some cases the transmitter is denoted as non-Ach or non-5HT etc. This means that the studies observed connections of non-cholinergic or non-serotoningeric nature but could not obtain what transmitter was involved.

The connectome and its spatial localization with respect to the rat brain is illustrated in figure SD2. In the designation of large-scale brain regions, we used the nomenklature which was introduced by Noori and colleagues (Noori et al., 2012). The terminology does not influence the network analytic results, as no assumptions on specific clustering properties were made based on the nomenklature. If consistent topological data was missing for a brain region, we did not present the existing connectivity in separate tables but rather included the existing information in the other tables in the section ‘other regions’ and if possible, were taken into consideration for mathematical analysis. The cerebral neurochemical connectome is of dynamical nature and evolving. It is safe to assume that with increasing number of anatomical studies, the missing links will be completed and the ‘other regions’ are integrated into the main connectome.


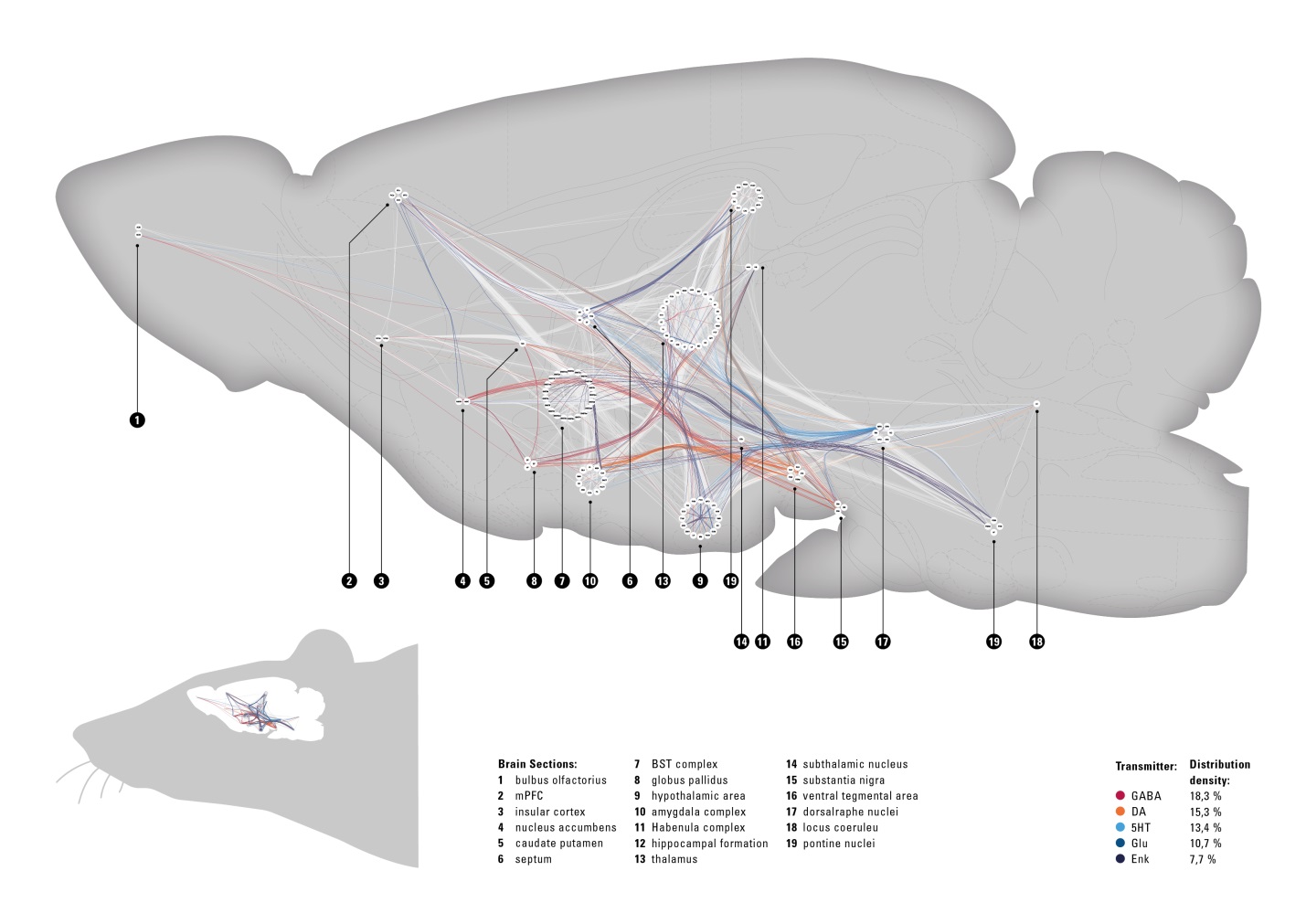


**S2 Figure 2.** The connectome in relation to the rat brain. Hierarchical edge-bundling algorithm in Cytoscape was used to visualize the adjacency of neuronal connections. The most prevalent transmitter are presented as colored edges. GABAergic neuronal connections (red) constitute the dominant chemical components at the intraregional scale (18.36% distribution density). In contrast, dopamine (orange), serotonin (light blue), glutamate (dark blue) and encephalin (black) represent the majority of chemical co-localizations of axonal projections at meso- and macro-scale.

It is noteworthy, that neuroVIISAS, BAMS (as well as BAMS2) and also hippocampome etc. provide databases that describe parts of the rat connectome. neuroVIISAS by Schmitt and Eipert (2012) is an evolving neuroinformatics tool that currently contains information on 106 regions and 1566 connections for the rat connectome. Thereby, data from neonatal, young and adult rats are included. Thus, inclusion of connectivity data of animals of different ages may lead to inconsistencies. Schmitt and colleagues in two excellent works also describe the connectivity of amygdala (2012) and basal ganglia (2016). ChemNetDB contains all 106 regions of neuroVIISAS as well as the local connectomes of amygdala and basal ganglia and therefore represents a superordinate structure. In addition, it further differs from neuroVIISAS in three main aspects:

1) ChemNetDB provides a consistent connectome of the adult rat brain. This is as critical, since it is well known that the connectomes are age-dependent dynamical structures and inclusion of data from animals of different age may lead to severe topological inconsistencies;

2) A major advantage of ChemNetDB is the fact that it includes data on neurotransmitters and neuropeptides. None of the existing databases and connectome approaches have ever addressed this aspect;

3) The nomenclature issues are not resolved in neuroVIISAS and therefore this database contains (in its current form) numerous redundancies. Using machine learning methods as described in the methods section, we have resolved this issue.

BAMS2 and BAMS (Bota et al., 2012) are good databases for rat CNS connectomes. The most recent version is a macroconnectome that contains 496 regions. However, only 15% of the connections are at this stage extracted from the literature, with the exception of cerebral cortex, BNST and amygdala. In addition to the above mentioned difference regarding the inclusion of neurochemicals in the connectomes, three main differences are substantial:

1) ChemNetDB is a multiscale connectome, i.e. it contains meso- and macroconnectomes;

2) the extraction of connectivity data for the 281 brain regions is complete in ChemNetDB;

3) A closer look at BAMS reveals that in contrast to ChemNetDB (which mostly contains cerebral nuclei >85%), a large proportion of areas in BAMS relate to cerebrospinal trunk and only approximately 25% refers to cerebral nuclei (see Figures 1 &2 Bota et al., 2012 Front Neuroinform).

In summary, the significant advance over the existing databases is that 1) ChemNetDB is currently the most comprehensive ***multi-scale*** database that contains previous databases as subsystems 2) it integrates neurochemical information in a consistent and validated manner and 3) it is consistent with respect to age of the animals and nomenclature.

# **List of abbreviations (Chemicals)**

| 5-HT | Serotonin/5-hydroxytryptamine |
| --- | --- |
| Ach | Acetylcholine |
| ADA | Adenosine |
| AMPA | α-amino-3-hydroxy-5-methyl-4-isoxazolepropionic acid |
| ASP | Aspartate |
| C | Catecholaminergic |
| CB | Cannabinoid |
| CCK | Cholecystokinin |
| CGRP | Calcitonin gene related peptide |
| CRF | Corticotropin releasing factor |
| ENK | Enkephalin |
| EX | Excitatory transmitter |
| GABA | Gamma-aminobutyric acid |
| GAD | Glutamic acid decarboxylase |
| Glu | Glutamate |
| NE | Norepinephrine |
| NGF | Nerve growth factor |
| NMDA | N-methyl-D-aspartate |
| NOS | Nitric oxid synthase |
| NPY | Neuropeptide Y |
| NT | Neurotensin |
| SP | Substance P |
| SS | Somatostatin |
| TH | Tyrosine hydroxylase |
| VAS | Vasopressin |
| VGluT | Vesicular glutamate transporter |
| VIP | Vasoactive intestinal peptide |
| β-END | β-Endorphin |
|  |  |

# **List of abbreviations (Brain regions)**

| \| MOB \| Main olfactory bulb \| \| --- \| --- \| \| AOB \| Accessory olfactory bulb \| \| IfCo \| Infralimbic cortex \| \| PrCo \| Prelimbic cortex \| \| ACC \| Anterior cingulate cortex \| \| lPFC \| Lateral prefrontal cortex \| \| InCoa \| Insular cortex, pars anterior \| \| InCop \| Insular cortex, pars posterior \| \| AcbSh \| Nucleus accumbens, shell \| \| AcbC \| Nucleus accubens, core \| \| Cpu \| Caudate Putamen \| \| LS \| Lateral septal nucleus \| \| MS \| Medial septal nucleus \| \| DB \| Nucleus of the diagonal band \| \| SF \| Septofimbrial nucleus \| \| TRS \| Triangular septal nucleus \| \| BNSTtr \| Bed nucleus of the stria terminalis, transverse nucleus \| \| BNSTdm \| Bed nucleus of the stria terminalis, dorsomedial nucleus \| \| BNSTdl \| Bed nucleus of the stria terminalis, dorsolateral nucleus \| \| BNSTjc \| Bed nucleus of the stria terminalis, juxtacapsular nucleus \| \| BNSTsc \| Bed nucleus of the stria terminalis, supracapsular nucleus \| \| BNSTmc \| Bed nucleus of the stria terminalis, magnocellular nucleus \| \| BNSTpr \| Bed nucleus of the stria terminalis, principal nucleus \| \| BNSTvl \| Bed nucleus of the stria terminalis, ventrolateral nucleus \| \| BNSTvm \| Bed nucleus of the stria terminalis, ventromedial nucleus \| \| BNSTal \| Bed nucleus of the stria terminalis, anterolateral nucleus \| \| BNSTfu \| Bed nucleus of the stria terminalis, fusiform nucleus \| \| BNSTif \| Bed nucleus of the stria terminalis, interfascicular nucleus \| \| BNSTrh \| Bed nucleus of the stria terminalis, rhomboid nucleus \| \| BNSTam \| Bed nucleus of the stria terminalis, anteromedial nucleus \| \| BNSTad \| Bed nucleus of the stria terminalis, anterodorsal area \| \| BNSTav \| Bed nucleus of the stria terminalis, anteroventral area \| \| BNSTv \| Bed nucleus of the stria terminalis, ventral nucleus \| \| BNSTov \| Bed nucleus of the stria terminalis, oval nucleus \| \| BNSTpm \| Bed nucleus of the stria terminalis, posteromedial nucleus \| \| BNSTpl \| Bed nucleus of the stria terminalus, posterolateral nucleus \| \| BNSTpo \| Bed nucleus of the stria terminalis, preoptic nucleus \| \| GP \| Globus pallidus \| \| VP \| Ventral pallidum \| \| EP \| Entopeduncular nucleus \| \| AHN \| Anterior hypothalamic nucleus \| \| LPO \| Lateral preoptic nucleus \| \| MPO \| Medial preoptic nucleus \| \| PVH \| Paraventricular hypothalamic nucleus \| \| PHN \| Posterior hypothalamic nucleus \| \| PM \| Premammillary nucleus (ventral and dorsal) \| \| SCH \| Suprachiasmatic nucleus \| \| TUM \| Tuberomammillary nucleus \| \| ARC \| Arcuate nucleus \| \| DMH \| Dorsomedial hypothalamic nucleus \| \| LH \| Lateral hypothalamus \| \| MM \| Mammillary nucleus ( medial and lateral) \| \| PeVH \| Periventricular hypothalamic nucleus \| \| SUM \| Supramammillary nucleus \| \| SO \| Supraoptic nucleus \| \| VMH \| Ventromedial hypothalamic nucleus \| \| CeA \| Central nucleus of amygdala \| \| MEA \| Medial nucleus of amygdala \| \| LA \| Lateral nucleus of amygdala \| \| BLA \| Basolateral nucleus of amygdala \| \| BMA \| Basomedial nucleus of amygdala \| \| BA \| Basal nucleus of amygdala \| \| ABA \| Accessory basal nucleus of amygdala \| \| COA \| Cortical nuclei of amygdala (anterior and posterior) \| \| PA \| Posterior nucleus of amygdala \| \| NLOT \| Nucleus of the lateral olfactory tract \| \| BAOT \| Basal nucleus of the accessory olfactory tract \| \| mHb \| Medial Habenula \| \| lHb \| Lateral Habenula \| \| PRE \| Presubiculum \| \| POST \| Postsubiculum \| \| PARA \| Parasubiculum \| \| SUB \| Subiculum \| \| ENT \| Entorhinal cortex \| \| DG \| Dentate Gyrus \| \| CA1 \| CA1 \| \| CA2 \| CA2 \| \| CA3 \| CA3 \| \| phCo \| Perirhinal cortex \| \| PosCo \| Postrhinal cortex \| \| AM \| Anteromedial nucleus of thalamus \| \| AV \| Anteroventral nucleus of thalamus \| \| CL \| Central lateral nucleus of thalamus \| \| CM \| Central medial nucleus of thalamus \| \| IMD \| Intermediodorsal nucleus of thalamus \| \| LGN \| Lateral geniculate nucleus of thalamus \| \| MGN \| Medial geniculate nucleus of thalamus \| \| RE \| Nucleus reuniens \| \| PCN \| Paracentral nucleus of thalamus \| \| PF \| Parafascicular nucleus of thalamus \| \| PT \| Parataenial nucleus of thalamus \| \| PVT \| Paraventricular nucleus of thalamus \| \| RT \| Reticular nucleus of thalamus \| \| VL \| Ventral lateral nucleus of thalamus \| \| VM \| Ventral medial nucleus of thalamus \| \| AD \| Anterodorsal nucleus of thalamus \| \| IAM \| Interanteromedial nucleus of thalamus \| \| LD \| Laterodorsal nucleus of thalamus \| \| LP \| Lateroposterior nucleus of thalamus \| \| MD \| Mediodorsal nucleus of thalamus \| \| RH \| Rhomboid nucleus of thalamus \| \| SPF \| Subparafascicular nucleus of thalamus \| \| POT \| Posterior thalamus \| \| NSM \| Nucleus submedius \| \| AL \| Anterolateral Nucleus Of The Thalamus \| \| STh \| Subthalamic Nucleus \| \| SNr \| Substantia nigra, pars reticulata \| \| SNc \| Substantia nigra, pars compacta \| \| SNl \| Substantia nigra, pars lateralis \| \| RLN \| Rostral linear nucleus \| \| CLN \| Caudal linear nucleus \| \| PNN \| Paranigral nucleus \| \| PBNp \| Parabrachial nucleus pigmentosus \| \| IFN \| Interfascicular nucleus \| \| DRN \| Dorsal raphe nucleus \| \| MRN \| Medial raphe nucleus \| \| RM \| Nucleus raphe magnus \| \| RPO \| Nucleus raphe pontis \| \| RPA \| Nucleus raphe pallidus \| \| RO \| Nucleus raphe obscurus \| \| LC \| locus coeruleus \| \| PBNl \| Lateral parabarchial nucleus \| \| PBNm \| Medial parabrachial nucleus \| \| KF \| Kölliker-Fuse nucleus \| \| PON \| Pontine nuclei \| |  |
| --- | --- | --- | --- | --- | --- | --- | --- | --- | --- | --- | --- | --- | --- | --- | --- | --- | --- | --- | --- | --- | --- | --- | --- | --- | --- | --- | --- | --- | --- | --- | --- | --- | --- | --- | --- | --- | --- | --- | --- | --- | --- | --- | --- | --- | --- | --- | --- | --- | --- | --- | --- | --- | --- | --- | --- | --- | --- | --- | --- | --- | --- | --- | --- | --- | --- | --- | --- | --- | --- | --- | --- | --- | --- | --- | --- | --- | --- | --- | --- | --- | --- | --- | --- | --- | --- | --- | --- | --- | --- | --- | --- | --- | --- | --- | --- | --- | --- | --- | --- | --- | --- | --- | --- | --- | --- | --- | --- | --- | --- | --- | --- | --- | --- | --- | --- | --- | --- | --- | --- | --- | --- | --- | --- | --- | --- | --- | --- | --- | --- | --- | --- | --- | --- | --- | --- | --- | --- | --- | --- | --- | --- | --- | --- | --- | --- | --- | --- | --- | --- | --- | --- | --- | --- | --- | --- | --- | --- | --- | --- | --- | --- | --- | --- | --- | --- | --- | --- | --- | --- | --- | --- | --- | --- | --- | --- | --- | --- | --- | --- | --- | --- | --- | --- | --- | --- | --- | --- | --- | --- | --- | --- | --- | --- | --- | --- | --- | --- | --- | --- | --- | --- | --- | --- | --- | --- | --- | --- | --- | --- | --- | --- | --- | --- | --- | --- | --- | --- | --- | --- | --- | --- | --- | --- | --- | --- | --- | --- | --- | --- | --- | --- | --- | --- | --- | --- | --- | --- | --- | --- | --- | --- | --- | --- | --- | --- | --- | --- | --- | --- | --- | --- |

1. Maier, T., Güell, M. & Serrano, L. Correlation of mRNA and protein in complex biological samples. *FEBS Letters*. **583**, 3966-3973 (2009). [↑](#footnote-ref-1)
2. Paxinos, G. & Watson, C. The rat brain in stereotaxic coordinates. *Academic Press*. **7th ed.** (2013). [↑](#footnote-ref-2)
